# The ultrastructural and proteomic analysis of mitochondria‐associated endoplasmic reticulum membrane in the midbrain of a Parkinson's disease mouse model

**DOI:** 10.1111/acel.14436

**Published:** 2024-11-29

**Authors:** Jin Liu, Yi Liu, Chao Gao, Hong Pan, Pei Huang, Yuyan Tan, Shengdi Chen

**Affiliations:** ^1^ Department of Neurology and Institute of Neurology, Ruijin Hospital Shanghai Jiao Tong University School of Medicine Shanghai People's Republic of China; ^2^ Department of Neurology, the Second Affiliated Hospital, Zhejiang University School of Medicine Zhejiang University Hangzhou People's Republic of China; ^3^ Lab for Translational Research of Neurodegenerative Diseases, Shanghai Institute for Advanced Immunochemical Studies (SIAIS) Shanghai Tech University Shanghai People's Republic of China

**Keywords:** 1‐methyl‐4‐phenyl‐1,2,3,6‐tetrahydropyridine, mitochondria‐associated endoplasmic reticulum membrane, Parkinson's disease, proteomics, ultrastructure

## Abstract

Recent studies indicated that the dysregulation of mitochondria‐associated endoplasmic reticulum membrane (MAM) could be a significant hub in the pathogenesis of Parkinson's disease (PD). However, little has been known about how MAM altered in PD. This study was aimed to observe morphological changes and analyze proteomic profiles of MAM in 1‐methyl‐4‐phenyl‐1,2,3,6‐tetrahydropyridine (MPTP)‐induced PD mouse models. In MPTP‐treated mice, transmission electron microscopy was applied for MAM ultrastructural visualization. Nano ultra‐high performance liquid chromatography‐tandem mass spectrum and bioinformatic analysis were adopted to obtain underlying molecular data of MAM fractions. The loosened, shortened and reduced MAM tethering was found in substantia nigral neurons from MPTP‐treated mice. In midbrain MAM proteomics, 158 differentially expressed proteins (DEPs) were identified between two groups. Specific DEPs were validated by western blot and exhibited significantly statistical changes, aligning with proteomic results. Bioinformatic analysis indicated that membrane, cytoplasm and cell projection were three major localizations for DEPs. Biological processes including metabolism, lipid transport, and immunological and apoptotic signaling pathways were greatly affected. For consensus MAM proteins, the enriched pathway analysis revealed the potential relationship between neurodegenerative diseases and MAM. Several biological processes such as peroxisome function and clathrin‐mediated endocytosis, were clustered, which provided additional insights into the fundamental molecular pathways associated with MAM. In our study, we demonstrated disrupted ER‐mitochondria contacts in an MPTP‐induced PD mouse model. The underlying signatures of MAM were revealed by proteomics and bioinformatic analysis, providing valuable insights into its potential role in PD pathogenesis.

## Graphical abstract

In MPTP‐induced PD mouse models, transmission electron microscopy was applied for MAM ultrastructural visualization. Nano ultra‐high performance liquid chromatography‐tandem mass spectrum and bioinformatic analysis were adopted to obtain the underlying molecular data of MAM fractions. Our findings showed that there was loosened, shortened and reduced MAM tethering in substantia nigral neurons from MPTP‐treated mice. In midbrain MAM fractions, specific proteins were validated by western blot and exhibited significantly statistical changes, aligning with proteomic results. The underlying signatures of MAM were revealed by proteomics and bioinformatic analysis, providing valuable insights into its potential role in PD pathogenesis.

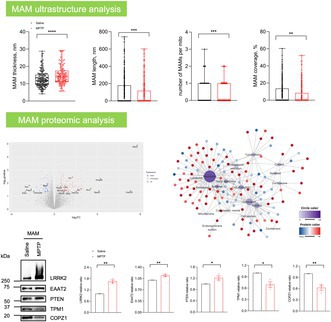

**Abbreviations**
6‐OHDA: 6‐hydroxydopamine
AD: Alzheimer's disease
ALS: Amyotrophic Lateral Sclerosis
ATP: adenosine triphosphate
BCA: bicinchoninic acid
BP: biological process
BSA: bovine serum albumin
CC: cellular component
CNX: calnexin
COPZ1: coatomer protein complex subunit zeta 1
Cox IV: cytochrome‐c‐oxidase subunit 4
DEPs: differentially expressed proteins
DNA: deoxyribonucleic acid
EAAT2: excitatory amino acid transporter 2
ER: endoplasmic reticulum
ERAD: endoplasmic reticulum‐associated degradation
FAIMS: field asymmetric ion mobility spectrometry
FC: fold change
GAPDH: glyceraldehyde‐3‐phosphate dehydrogenase
GFAP: glial fibrillary acidic protein
GO: gene ontology
GSEA: gene set enrichment analysis
HD: Huntington's disease
HPA: Human protein atlas
HRP: horseradish peroxidase
IACUC: Institutional animal care and use committees
IHC: immunohistochemistry
iPSCs: induced pluripotent stem cells
iRT: indexed retention time
KEGG: Kyoto encyclopedia of genes and genomes
LRRK2: leucine‐rich repeat kinase 2
MAM: mitochondria‐associated ER membrane
MCSs: membrane contact sites
MERCs: mitochondria‐ER contact sites
MF: molecular function
MPTP: 1‐methyl‐4‐phenyl‐1,2,3,6‐tetrahydropyridine
MRB: mitochondria resuspending buffer
NDs: neurodegenerative diseases
NES: normalized enrichment score
OMM: mitochondrial outer membrane
OXPHOS: oxidative phosphorylation
PBS: phosphate‐buffered saline
PCA: principal component analysis
PD: Parkinson's disease
PINK1: PTEN‐induced putative kinase 1
PLS‐DA: partial least squares discriminant analysis
PPI: protein–protein interaction
PTEN: phosphatase and tensin homolog
RIPA: radio immunoprecipitation assay
ROS: reactive oxygen species
SDS‐PAGE: sodium dodecyl sulfate polyacrylamide gel electrophoresis
SEM: standard error of mean
Sig‐1R: sigma‐1 receptor
SN: substantia nigra
TCA: tricarboxylic acid
TEM: transmission electron microscopy
TFA: trifluoroacetic acid
TH: tyrosine hydroxylase
TMT: tandem mass tag
TPM1: tropomyosin 1
UHPLC–MS/MS: ultra‐high performance liquid chromatography‐tandem mass spectrum
VDAC1: voltage dependent anion channel 1
WB: western blot
WT: wild type

## 1 INTRODUCTION

Parkinson's disease (PD) is one of the most common movement disorders. Up to now, the exact etiology and pathogenesis of PD still remain largely elusive, bringing great challenges in disease prevention and targeted therapy (Bloem et al., 2021). Multiple risk factors such as ageing, environmental factors and genetic backgrounds, have been reported to be related to the occurrence of PD. Experimental results have found that mitochondrial dysregulations are characteristic features of ageing process. PD‐associated neurotoxins, including rotenone and 1‐methyl‐4‐phenyl‐1,2,3,6‐tetrahydropyridine (MPTP), mainly interfered with mitochondrial bioactivities. Additionally, proteins encoded by PD‐related genes, including PTEN‐induced putative kinase 1 (PINK1), Parkin and DJ‐1, were widely involved in mitochondria homeostasis. In both idiopathic and monogenic PD cases, mitochondria‐mediated biological processes (BP), for example, bioenergy generation, calcium flow and mitophagy, were extensively impaired. Research findings have highlighted that the destruction of mitochondrial complex I could directly lead to parkinsonian phenotypes (Gonzalez‐Rodriguez et al., 2021). These evidences fully underscore the pivotal role of mitochondrial stability in the pathogenesis of PD.

Mitochondria‐associated endoplasmic reticulum (ER) membrane (MAM), as one of the widely‐existed membrane contact sites (MCSs), is a specialized ultrastructural contact formed by ER membrane and outer mitochondria membrane (OMM) within a distance less than 30 nm (Csordás et al., 2006; Liu et al., 2019; Lombardi & Elrod, 2017). Under physiological conditions, MAM serves as the key element for maintaining structural and functional balances of both ER and mitochondria (Liu & Zhu, 2017). A number of vital biological processes such as calcium exchange, phospholipid exchange, mitochondrial fusion and fission, mitophagy, oxidative stress, and bioenergy production, are finely regulated by this sub‐organelle component (Filadi et al., 2017). Recent studies indicated that the dysregulation of MAM could be a significant hub in the pathogenesis of PD (Markovinovic et al., 2022). The major altered pathways during PD development, for example, oxidative stress, calcium imbalance and mitochondrial abnormality, were largely overlapped with MAM bio‐activities. In addition, several PD‐related proteins such as α‐synuclein (Guardia‐Laguarta et al., 2014; Paillusson et al., 2017), Parkin (Grossmann et al., 2023), PINK1 (Grossmann et al., 2023) and DJ‐1 (Liu et al., 2019; Parrado‐Fernandez et al., 2018), were demonstrated to regulate MAM assembly and function in genetic PD models. However, sporadic cases and general PD models were less studied in this field. A basic recognitions of MAM alterations from PD mouse model is also lacking.

In this study, we adopted the subacute MPTP‐treated PD mouse model. The ultrastructural alterations of MAM were observed in substantia nigral and striatal neurons. Quantitative proteomics and bioinformatic analysis were also applied to reveal the underlying features of midbrain MAM fractions in MPTP‐treated mice. We speculated that there were disrupted MAM morphological changes and dysregulated MAM biological performances in MPTP‐induced PD mouse models.

## 2 EXPERIMENTAL PROCEDURES

### 2.1 Experimental animals

Nine‐week‐old male wild type (WT) C57BL/6J mice were purchased from Beijing Vital River Laboratory Animal Technology Co., Ltd. (Shanghai Branch, P.R. China). Animals were housed under standard conditions in terms of density, humidity, temperature, daily light/dark cycles, water, diet, and so forth. After one‐week's acclimatization, mice were randomly assigned to MPTP group or saline group, weighted, and intraperitoneally injected with a dose of 30 mg/kg MPTP (Sigma‐Aldrich) or equal converted volume of saline once a day for five consecutive days. Specific experimental operations basically followed the introduction of the previously published protocol (Jackson‐Lewis & Przedborski, 2007). Two‐day behavioral trainings began on the seventh day after the final dose. Behavioral tests were performed on the ninth day. On the tenth day after the end of drug injection, mice were sacrificed for corresponding experimental analysis. Animal associated experiments were approved by Ruijin Hospital Ethics Committee and Shanghai Tech University Institutional Animal Care and Use Committee (IACUC No. 20220503001). All animal related operations were conducted in accordance with the regulations of Shanghai Jiao Tong University School of Medicine Animal care and Use Program and regulations of committee and laboratory animal department of Shanghai Tech University.

### 2.2 Pole test

Pole test has been widely applied for evaluating movement coordination abilities in mice. Firstly, A small platform was placed and fixed on the top of pole. The bottom was smoothly laid in a cage with bedding materials. The mice were placed on the platform with their heads upwards. The time when they turned downwards, and when they descended to the bottom of the pole were recorded. The mice were trained for three times a day with an interval of at least 1 h. After 2 days' training, performance results were collected and analyzed.

### 2.3 Western blot (WB)

Isolated brain tissues were lysed with radio immunoprecipitation assay (RIPA) lysis buffer (Beyotime Biotechnology). Organelle and subcellular organelle fractions were lysed with 1× cell lysis buffer (Cell Signaling Technology). Protease inhibitor cocktail (Cell Signaling Technology), and phenylmethanesulfonyl fluoride (Beyotime Biotechnology) were both added to the corresponding lysate. Concentration of protein samples was evaluated by bicinchoninic acid (BCA) protein assay kit (Thermo Fisher Scientific). Samples were then boiled with loading buffer (Beyotime Biotechnology) at 95°C for 5 min, separated by molecular weights using sodium dodecyl sulfate polyacrylamide gel electrophoresis (SDS‐PAGE) methods (Epizyme Biotech), and transferred to polyvinylidene fluoride membranes (Millipore). The 5% bovine serum albumin (BSA) solution was applied for blocking. Primary antibodies used in this study included that anti‐tyrosine hydroxylase (TH) rabbit mAb (1:2000, Cat. 58844, Cell Signaling Technology), anti‐glial fibrillary acidic portein (GFAP) rabbit mAb (1:2000, Cat. 80788, Cell Signaling Technology), anti‐glyceraldehyde‐3‐phosphate dehydrogenase (GAPDH) rabbit mAb (1:2000, Cat. 5174, Cell Signaling Technology), anti‐cytochrome‐c‐oxidase subunit 4 (Cox IV) rabbit Ab (1:2000, Cat. 5844, Cell Signaling Technology), anti‐sigma‐1 receptor (Sig‐1R) rabbit mAb (1:2000, Cat. 61994, Cell Signaling Technology), anti‐phosphatase and tensin homolog (PTEN) rabbit mAb (1:2000, Cat. 9188, Cell Signaling Technology), anti‐calreticulin rabbit mAb (1:2000, Cat. 92516, Abcam), anti‐calnexin (CNX) rabbit pAb (1:2000, Cat. ADI‐SPA‐860, Enzo Life Sciences), anti‐voltage dependent anion channel 1 (VDAC1) rabbit pAb (1:2000, Cat. 55259‐1‐AP, Proteintech), anti‐leucine‐rich repeat kinase 2 (LRRK2) rabbit mAb (1:1000, Cat. R380823, Zen‐bioscience), anti‐excitatory amino acid transporter 2 (EAAT2) rabbit mAb (1:1000, Cat. R381853, Zen‐bioscience), anti‐tropomyosin‐1 (TPM1) rabbit mAb (1:1000, Cat. R383145, Zen‐bioscience), and anti‐coatomer protein complex subunit zeta 1 (COPZ1) rabbit pAb (1:1000, Cat. 824631, Zen‐bioscience). Secondary antibodies with horseradish peroxidase (HRP) conjugation (1:5000) were used for incubation. Both primary and secondary antibody treatments were followed by tris‐buffered saline and tween‐20 (Sangon Biotech) washing for several times. The strips were detected by mixed immobilon western HRP substrates (Millipore), using the ChemiDoc MP gel imaging system (Bio‐rad). Image quantification was conducted by ImageJ software. Analytical data was calculated through GraphPad Prism8 software.

### 2.4 Immunohistochemistry (IHC)

Whole mouse brains were processed successively by perfusion with cold saline, fixation with 4% paraformaldehyde (Beyotime Biotechnology), dehydration with 30% sucrose solution, and cryostat sectioning by 25 μm in thickness. Brain sections were firstly treated with 3% hydrogen peroxide for 10 min, and washed several times by phosphate‐buffered saline (PBS). The 1% BSA and 0.3% Triton X‐100 solutions were used for blocking. Anti‐TH rabbit mAb (1:200, Cat. 58844, Cell Signaling Technology) was then added overnight at 4°C. After incubation of anti‐rabbit secondary antibodies with HRP conjugation (1:300) and washing procedures several times, the section stains were developed by diaminobenzidine horseradish peroxidase color development kit (Servicebio), and scanned.

### 2.5 Transmission electron microscopy (TEM)

Three mice in each group were anesthetized and perfused by cold PBS and fresh 2.5% glutaraldehyde fixative through left ventricle ordinally. Substantia nigra (SN) and striatum tissues were carefully isolated by the authors and stored in 2.5% glutaraldehyde fixative at 4°C overnight. Samples were then transferred to electron microscopic core facility of Basic Medical Sciences in Shanghai Jiao Tong University School of Medicine for further processing and photographing (Li et al., 2021). The operating staff was blinded to sample groups. When sections were performed by ultramicrotome EM UC7 (Leica) and stained by lead citrate, TEM machine H‐7650 (Hitachi) was applied for photographing. Neurons were undifferentiated pictured by operating staff without intervention from the authors. MAM was a specialized tight ultrastructural contact formed by ER membrane and outer mitochondria membrane within a distance less than 30 nm (Csordás et al., 2006; Liu et al., 2019; Lombardi & Elrod, 2017). The parameters of every MAM included that the shortest vertical distance between ER and OMM as MAM thickness, ER length apposed to OMM within 30 nm thickness as MAM length, MAM number existed per mitochondria, and coverage percentage of mitochondria surface forming close contacts with ER as MAM coverage. Based on MAM thickness, MAM was divided into three subgroups as super tight type (<10 nm), tight type (10–20 nm) and loose type (20–30 nm) (Csordás et al., 2006). Based on MAM length, MAM was divided into three subgroups as short type (<200 nm), medium type (200–400 nm) and long type (>400 nm) (Liu et al., 2019). Mitochondrial related parameters were mitochondrial area, length, aspect ratio (major/minor axis of mitochondria), and cristae score (Crabtree et al., 2023). All visible mitochondria and associated ER membranes in TEM images were measured by ImageJ software in an un‐blinded condition. Analytical data were calculated by the authors through GraphPad Prism8 software.

### 2.6 Isolation of MAM fractions

Cell fractionation and organelle isolation from mouse midbrains were conducted by the differential centrifugation methods (Wieckowski et al., 2009). Specific experimental procedures could be referred in this well‐established protocol (Wieckowski et al., 2009). In our studies, three independent MAM biological samples were prepared for both saline and MPTP‐treated mice, and each MAM biological sample was extracted from pooled eight independent midbrain tissues within one group. Fresh mouse midbrain tissues were first washed by IB‐1 buffer (225 mM mannitol, 75 mM sucrose, 0.5% BSA, 0.5 mM EGTA, and 30 mM Tris–HCl pH 7.4), gently grinded with a proper glass homogenizer, and resuspended by IB‐1 buffer. The homogenate was centrifuged at least five times at 740 *g* for 5 min, and then at 9000 *g* for 10 min. The supernatant, which was obtained by centrifugation of 9000 *g* speed, consisted of ER, cytosolic fractions, lysosome, and so forth. The pellet, which was obtained by centrifugation of 9000 *g* speed, was called crude mitochondria and consisted of pure mitochondria and MAM fraction. For the supernatant component, which was obtained with centrifugal speed of 9000 *g*, further isolation was performed by centrifugation at 20000 *g* for 30 min (the resultant pellet containing lysosome and plasma membrane fractions was removed), and then at 100000 *g* for 1 h (the resultant pellet was reserved as pure ER fraction). The crude mitochondria pellet, which was prepared by primary centrifugation of 9000 *g* speed, was resuspended by IB‐2 buffer (225 mM mannitol, 75 mM sucrose, 0.5% BSA, and 30 mM Tris–HCl pH 7.4), and centrifuged at 10000 *g* for 10 min. After removing the supernatant, pellet fraction was then resuspended by IB‐3 buffer (225 mM mannitol, 75 mM sucrose, and 30 mM Tris–HCl pH 7.4), and centrifuged again at 10000 *g* for 10 min. Next, percoll medium (225 mM mannitol, 25 mM HEPES pH 7.4, 1 mM EGTA, and 30% percoll vol/vol) with a volume of 2.5 mL was prepared, and added in a 3 mL polycarbonate tube. The crude mitochondria pellet, which was obtained by previous centrifugation at 10000 *g* speed, was further resuspended in mitochondria resuspending buffer (MRB, 250 mM mannitol, 5 mM HEPES pH 7.4, and 0.5 mM EGTA), gently added to the top of percoll medium in the 3 mL polycarbonate tube, and centrifuged at 95000 g for 30 min (SW 60 Ti rotor, Beckman coulter). After centrifugation of 95000 *g* speed, purified mitochondria component, as a dense band at the bottom of the polycarbonate tube, was diluted in MRB and centrifuged three times at 20000 *g* for 10 min (the resultant pellet was reserved as pure mitochondria fraction). MAM fraction, as a cloudy white band in the middle part after centrifugation of 95000 *g* speed, was removed, diluted by MRB, and centrifuged at 6300 *g* for 10 min. The supernatant obtaining from centrifugal speed of 6300 *g*, was diluted and centrifuged twice at 100000 *g* for 1 h to extract purified MAM pellet. All solutions were placed on ice. Centrifugation procedures above were set to operate at 4°C. The pipette tips needed to be cut to prevent mechanical damage for organelle components. Subcellular fractions from the two groups were standardized to equal protein concentrations, and validated with specific protein markers by western blot approach.

### 2.7 Nano‐UHPLC–MS/MS analysis

Three independent MAM biological samples for both MPTP and control groups were transferred to mass spectrum core facility of basic medical sciences in Shanghai Jiao Tong University School of Medicine for further processing. Firstly, MAM fractions were suspended in a buffer with 2% SDS and 50 mM DTT, and grinded for 20 min. Before boiling at 100°C for 5 min, protein concentration was tested for MAM samples. As shown in the Supplementary Figure (Figure S1a), there were variations in MAM protein concentrations. For two MAM samples with rather higher protein concentrations in each group, associated proteomics of these samples was tested twice (Saline group, 2–16 (1, 2), 2–17 (1, 2); MPTP group, 2–19 (1, 2), 2–20 (1, 2)) (Figure S1b). For MAM samples with relatively lower protein concentrations, proteomics was tested once (Saline group, 2–15 (1); MPTP group, 2–18 (1)) (Figure S1b). We also combined the three separate MAM fractions of each group into a single sample, and conducted proteomic testing three times, labeling them as mix‐1, mix‐2, and mix‐3 in both saline and MPTP groups (Figure S1b). Therefore, there were altogether eight MAM proteomic testing results from three biological samples and five technical replicates for each group.

Samples were then alkylated (200 mM iodoacetamide) in the dark for 1 h, precipitated at −20°C overnight by addition of a five‐time volume of acetone, resolved and digested at 37°C overnight again in sequencing grade modified trypsin buffer (Promega), and centrifuged at 14000 g for 20 min. The tryptic peptides were further purified by treatment of 1% trifluoroacetic acid (TFA), eluted with buffer (0.1% TFA, and 50 ~ 70% acetonitrile), lyophilized by means of SpeedVac (ThermoSavant), and resuspended in buffer (1% formic acid, and 5% acetonitrile). The indexed retention time (iRT) peptides (Biognosys) were then spiked into samples following instructions. After re‐dissolution by solvent A (0.1% formic acid in water), cleaved peptides were analyzed by Orbitrap Exploris 480 mass spectrometer, with field asymmetric ion mobility spectrometry (FAIMS) technique coupled in EASY nLC 1200 system (Thermo Fisher Scientific). Peptide sample with a volume of 2 μL was added onto analytical column, and separated by a stepwise increased concentration of buffer B (80% acetonitrile, and 0.1% formic acid) along with maintained flow rate (250 nL/min), temperature (55°C) and voltage (2 kV) settings. Mass spectrometer was operated in an independent acquisition mode with hybrid data strategy. Parameters of the survey scan included a 120,000 resolution, a maximum injection time of 20 ms, and normalized AGC target of 3e6. During MS2 acquisition process, a variety of isolation window widths were performed such as 10 m/z (mass range from 408 *m*/*z*—795 *m*/*z* with 43 windows), 20 *m*/*z* (mass range from 795 *m*/*z* to 985 *m*/*z* with 11 windows), 30 *m*/*z* (mass range from 350 *m*/*z* to 408 *m*/*z* with 2 windows), and 50 *m*/*z* (mass range from 985 *m*/*z* to 1200 *m*/*z* with 4 windows). Parameters of a full scan followed by 20 windows were a 30,000 resolution, normalized collision energy (27, 30, and 33), and AGC target of 1e6. For MS/MS scans and survey scans, compensation voltages (45, and 65 V) were both chosen. Dynamic iRT was selected as retention time prediction type. Missing two specialized cleavages from specific trypsin enzyme was allowed. Carbamidomethyl of cysteine was chosen as a fixed modification, and oxidation of methionine as a variable modification. Ideal window was dynamically defined based on iRT calibration and gradient stability. Data extraction was determined by Spectronaut 14, which was based on the extensive mass calibration. Mass tolerances for precursor ions and fragment ions were also automatically calculated and calibrated by the software Spectronaut 14 (Biognosys) through machine deep learning methods. Other parameters were 1% *Q* value cutoff on precursors and proteins levels, mutated decoy generation which adopted a random number of amino acid position swamps (min = 2, max = length/2), and global normalization strategy. Major group quantities were calculated by the average top 3 filtered peptides passing 1% *Q* value cutoff. The mouse Uniprot fasta database (53,099 entries, downloaded on November 04, 2018) was applied for searching.

After initial data distribution analysis by partial least squares discriminant methods (PLS‐DA) (R version 4.2.2, mixOmics.plotIndiv 6.3.2), three sets of data from each group were excluded due to dispersed distribution, as these results might introduce potential data bias (Figure S1b,c). For saline group, the excluded samples were Saline 2–25, mix‐1, and mix‐2. For MPTP group, the excluded samples were MPTP 2–18, 2–20 (2), and mix‐1. Selected samples were followed by a red tick in the flow diagram (Figure S1b), and were circled by a black box in the PLS‐DA figure (Figure S1c). A final 5 versus 5 design was then selected for subsequent MAM proteomic analysis. Considering that each biological sample contained a mixture of eight independent midbrain tissues, the limitations of such a small sample size was partially mitigated. For quantitative statistics, not available (NA) was set for obviously undetected proteins. If one protein was detected in less than two testing samples for each group, this protein was viewed as non‐detected and the corresponding protein information from this group was discarded. Shapiro–Wilk methods were applied for normal distribution analysis. After unpaired two‐tailed *t* test analysis of converted log_2_ value, *p* value was acquired and corrected by Benjamini and Hochberg methods. The differentially expressed proteins (DEPs) were selected for *p* value being less than 0.05, and fold change (FC) being more than 1.2. Basic analysis included venn diagram (Jvenn), principal component analysis (PCA) (stats), PLS‐DA (mixOmics.plotIndiv 6.3.2), protein complex information (CORUM database), and protein subcellular information (Human protein atlas database, and Uniprot database). For DEPs and consensus MAM proteins, another analysis approaches were volcano plots (ggplot2 3.4.1), clustering heatmap (pheatmap 1.0.12), gene ontology (GO), Kyoto encyclopedia of genes and genomes (KEGG), gene set enrichment analysis (GSEA) (ClusterProfiler), and protein–protein interaction (PPI) analysis (STRING).

### 2.8 Protein complex analysis

Protein complex entries were retrieved by mouse CORUM database (https://mips.helmholtz‐muenchen.de/corum/) (856 entries, downloaded on January 08, 2023). We compared our midbrain MAM proteomics data with 856 downloaded protein complexes. A complex was considered as a potential MAM protein complex if all its constituent proteins were detected in our MAM proteomics analysis. There were altogether 180 protein complexes meeting the demand above, summarized as supplementary materials.

### 2.9 Subcellular location analysis

Protein localization analysis was searched by Human Protein Atlas (HPA) database (https://www.proteinatlas.org) and Uniprot database (https://www.uniprot.org) respectively. For proteins with a single location, results were presented as exclusive outcomes. Entire position data of all identified proteins were marked as combined results. For DEPs, Cytoscape was applied to draw correlation diagram about proteins and corresponding subcellular locations. The depth of large purple circle was positively depended on attached protein number. The smaller circle represented every significant protein, of which red color meant up‐regulation, and blue color meant down‐regulation.

### 2.10 Functional enrichment analysis

GO analysis was conducted by Bioconductor (org.Mm.eg.db). Biological process, molecular function (MF), and cell component (CC) were three parts for GO analysis. KEGG analysis was performed by database (https://www.genome.jp/kegg). GSEA was performed by R studio (ClusterProfiler). For GSEA methods, MPTP‐treated group was set to compare with controls. The positive value of normalized enrichment score (NES) meant that the specific gene set or biological pathway was up‐regulated in MPTP‐treated group in comparison to controls. The negative value of NES meant that the specific gene set or biological pathway was down‐regulated in MPTP‐treated group in comparison to controls. Associated bubble graph and bar graph of NES were conducted by R studio (ggplot2 3.4.1). The main top enriched entries with *p* value being less than 0.05 were exhibited as bubble graph unless specifically illustrated. The main top enriched pathways with *p* value being less than 0.05 and NES being larger than 1 were included for bar graph unless specifically illustrated.

### 2.11 Protein–protein interaction analysis

The data of detected consensus MAM proteins was analyzed by PPI network (https://string‐db.org/), and first clustered by obtained KEGG results (https://biit.cs.ut.ee/gprofiler/). For BP results, these terms were manually annotated into specific functional group, and visually enriched by K‐means clustering analysis.

### 2.12 Statistical analysis

GraphPad Prism8 software was applied for statistical analysis. Numerical variables were presented as forms of mean ± standard error of mean (SEM). Shapiro–Wilk methods were applied for normal distribution analysis. Unpaired *t*‐test (two‐sided) methods were used unless otherwise mentioned. The cutoff of *p* value was 0.05. Denotation of the significances were * *p* < 0.05, ** *p* < 0.01, *** *p* < 0.001, and **** *p* < 0.0001.

## 3 RESULTS

### 3.1 Validation of an MPTP‐treated PD mouse model

**FIGURE 1:**
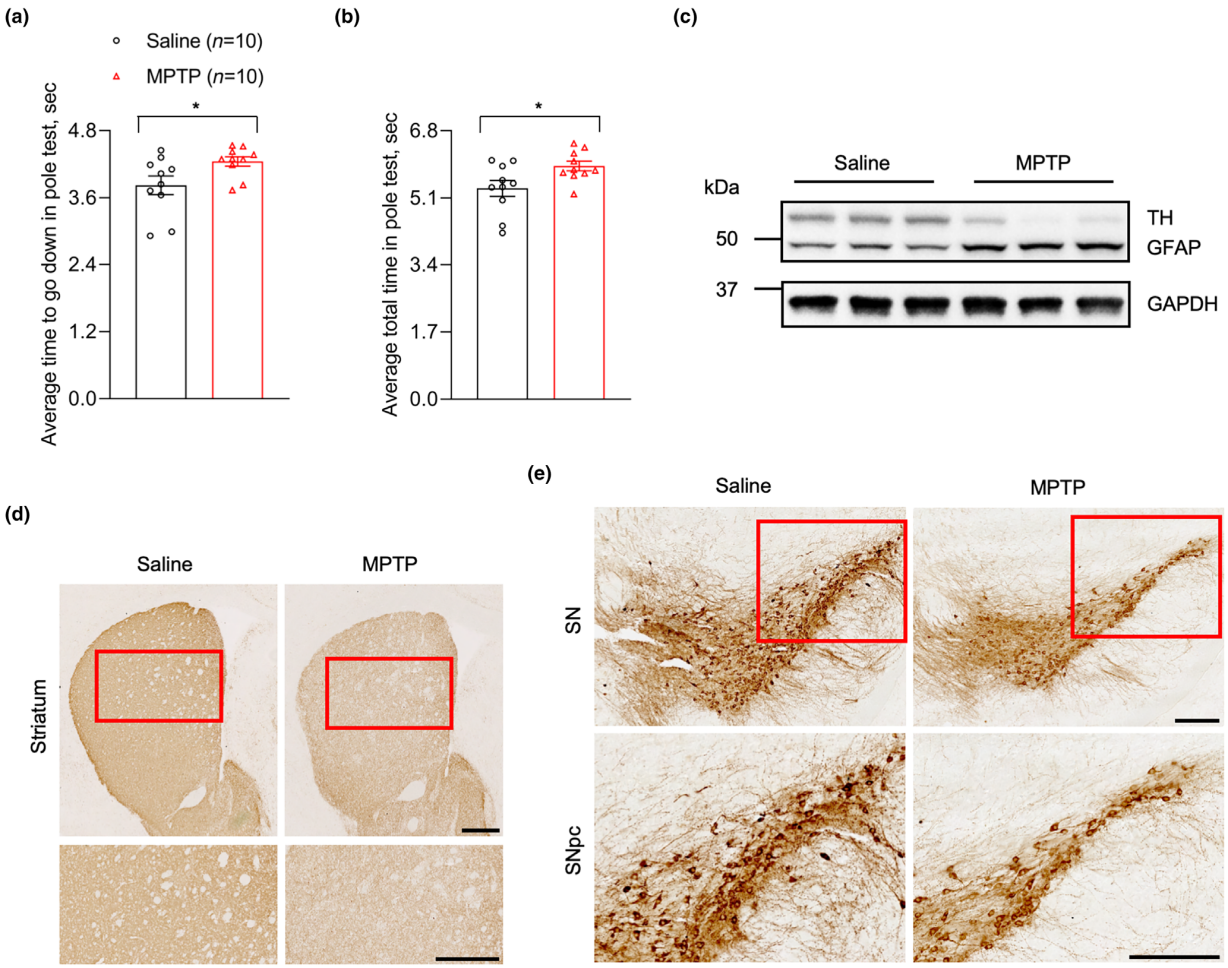
MPTP‐treated mice exhibited decreased motor abilities and the loss of dopaminergic neurons. (a, b) The statistical analysis of the average time from heads downwards to the bottom of the pole (a), and average time of entire recording (b) in controls and MPTP‐treated mice (*n* = 10 per group). (c) The representative expression levels of TH and GFAP proteins from striatum tissues in controls and MPTP‐treated mice by WB approach (*n* = 3 per group). (d, e) Representative IHC graphs of TH staining in striatum (d) and SN (e) sites in controls and MPTP‐treated mice (Scale bar, 500 and 200 μm, respectively). The measurement data were presented as the form of means ± SEM, unpaired two‐tailed *t* test; *p < 0.05.

The subacute MPTP‐treated PD mouse model (Jackson‐Lewis & Przedborski, 2007) was applied in this study. In pole test, MPTP‐treated mice exhibited poorer movement coordination abilities than controls, reflecting in longer time to climb down the pole and finish the whole process (Figure 1a,b). In aspects of pathology, western blot analysis of TH and GFAP markers confirmed the loss of TH‐positive dopaminergic neurons and the proliferation of astrocytes in MPTP‐induced PD mouse models, respectively (Figure 1c). In situ TH immunohistochemical staining further clearly displayed the worse survival status of dopaminergic neurons in both striatum and SN sites from MPTP‐treated mice (Figure 1d,e).

### 3.2 Impaired MAM ultrastructure in MPTP‐treated mice

**FIGURE 2:**
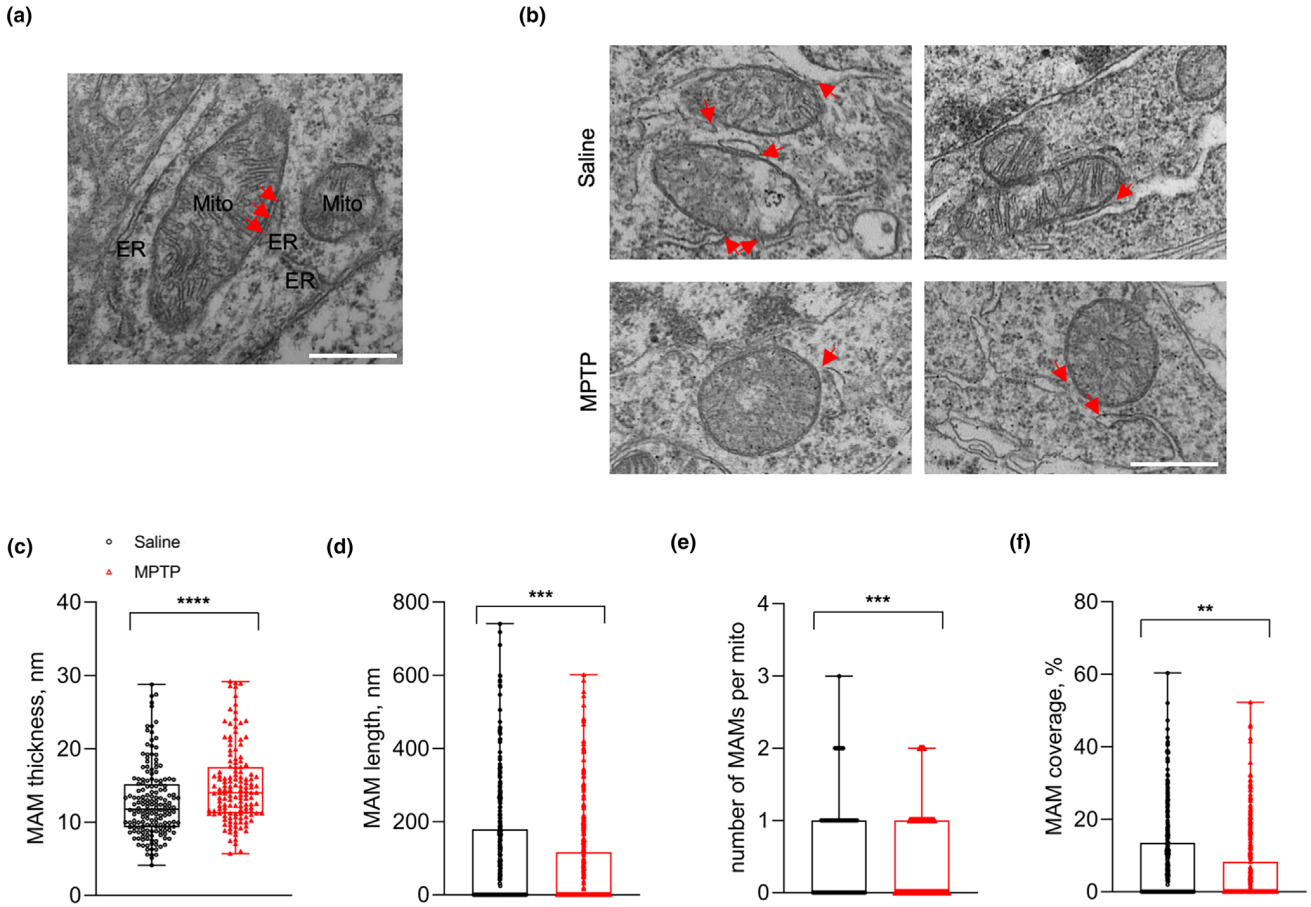
The disrupted MAM ultrastructure from SN neurons in MPTP‐treated mice. (a) Representative TEM image of MAM morphologies in SN neurons from saline mice, and the red arrow indicated MAM formation (Scale bar, 500 nm). (b) Representative TEM images of MAM morphologies in SN neurons from controls and MPTP‐treated mice (*n* = 3 per group), and the red arrow indicated MAM formation (Scale bar, 500 nm). (c–f) Quantitative analysis of average MAM thickness as the shortest vertical distance between ER and mitochondrial outer membrane (c), average MAM length as ER length which apposed to mitochondrial outer membrane within 30 nm thickness (d), average MAM number existed per mitochondria (e), and average MAM coverage percentage as coverage percentage of mitochondria surface forming close contacts with ER (f) between controls (mitochondria *n* = 307) and MPTP‐treated mice (mitochondria *n* = 353) (mouse *n* = 3 per group). The measurement data were presented as the form of means ± SEM, unpaired two‐tailed *t* test; **p < 0.01, ***p < 0.001, ****p < 0.0001.

MAM was a specialized ultrastructural contact formed by ER membrane and mitochondrial outer membrane within a distance less than 30 nm. In plenty of literatures, mitochondria‐ER contact sites (MERCs) are often utilized to depict the structural aspect, while MAM is more focused on the biochemical aspects under specific disease conditions (Janikiewicz et al., 2018; Liu & Zhu, 2017; Simmen & Herrera‐Cruz, 2018). Therefore, MAM was separately chosen to represent such a contact site in our study. To better understand such ultrastructure, MAM was marked with red arrows in a higher magnification of corresponding formation areas (Figure 2*a*). We used transmission electron microscopy to observe MAM morphology in controls and MPTP‐treated mice, and related parameters were analyzed (Table S1). In SN neurons, ER‐mitochondria tethering was significantly loosened and shortened from MPTP‐treated mice when compared with controls (Figure 2b–d). MAM number and coverage percentage were also reduced in MPTP group (Figure 2e,f). In addition, mitochondrial area and length were not significantly different between two groups (Figure S2a,b). The lower value of aspect ratio and cristae score in MPTP‐treated mice suggested a certain degree of structural abnormality of mitochondria (Figure S2c,d). While in striatal neurons, the number of MAMs per mitochondria and coverage percentage of MAM were mainly affected in MPTP‐treated mice (Figure S3a–d, and Table S1). The above evidences indicated that the MAM ultrastructure was disrupted in both SN and striatal neurons in MPTP‐induced PD mouse models.

### 3.3 Quantitative proteomic analysis in midbrain MAM fractions

**FIGURE 3:**
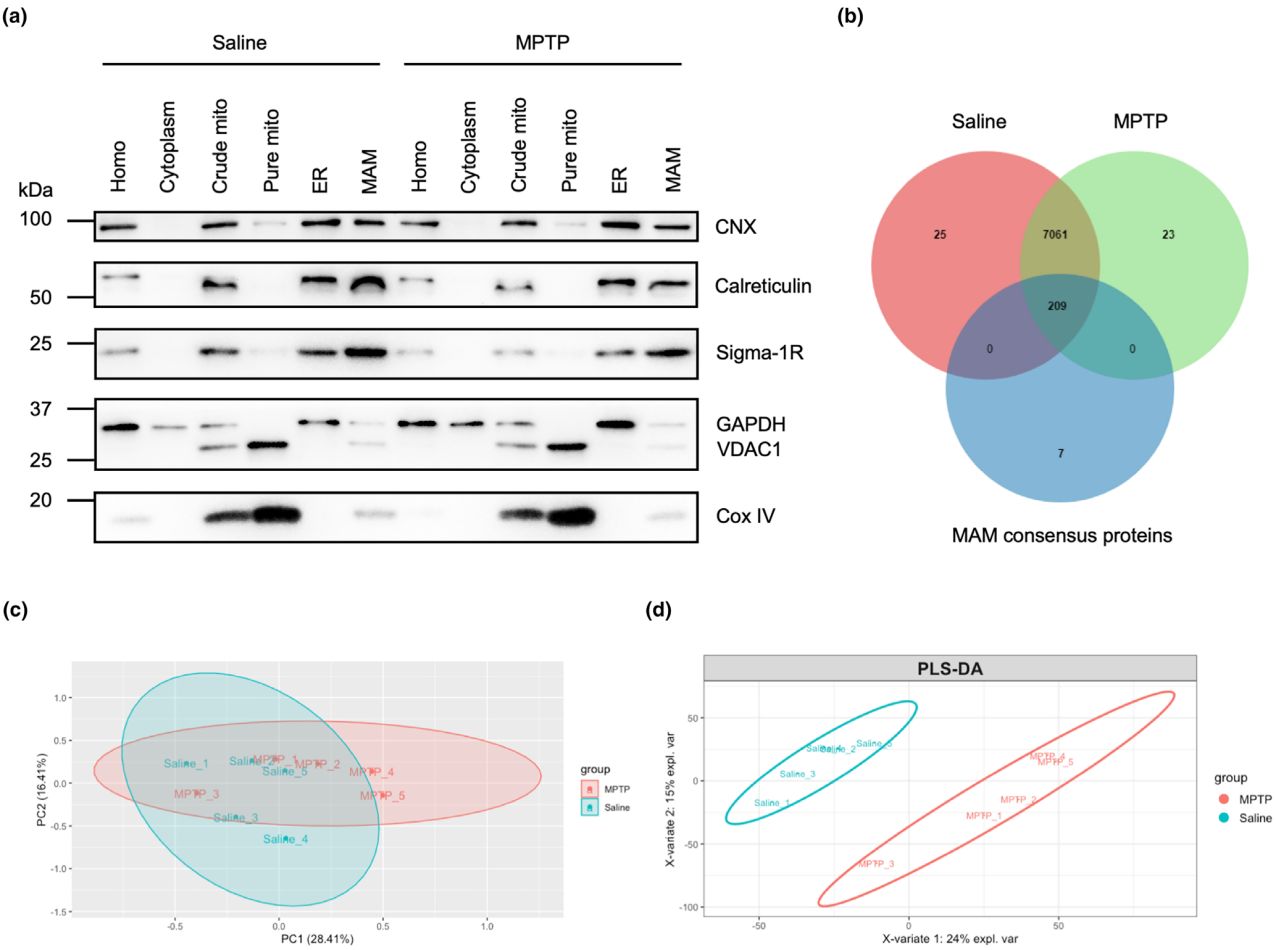
Initial proteomic parsing of midbrain MAM fractions in controls and MPTP‐treated mice. (a) Subcellular fractions extracted from midbrain tissues were validated by specific organelle protein markers. Calnexin, Calreticulin, and Sigma‐1R were considered as consensus proteins for ER and MAM. VDAC1 and Cox IV were applied for mitochondria and MAM markers, and GAPDH was to prove cytosolic fractions. (b) Venn graph of identified proteins in each group (*n* = 5 per group) and consensus MAM proteins. (c, d) Sample distribution plots by PCA (c) and PLS‐DA analysis (d) in MAM proteomics between controls and MPTP‐treated mice.

In our study, a qualified MAM biological sample was extracted from eight independent midbrain tissues within one group via the high‐speed differential ultracentrifugation methods (Wieckowski et al., 2009). All the yield compartments were validated with specific protein markers by western blot approach (Figure 3a). To improve the reliability of our findings, we increased the sample size by performing sample mixing and repeated detections. For each group, there were altogether eight MAM proteomic testing results from three biological samples and five technical replicates in initial nano‐UHPLC–MS/MS analysis. After preliminary screening of these data, a final 5 versus 5 design was selected for further proteomic analysis (Figure S1a–c). Quantitative proteomic analysis detected more than 7000 proteins in midbrain MAM fractions (Figure 3b, and Table S2, S3). For proteins identified based on a single unique peptide, associated protein information was provided (Table S4, S5). Many PD related proteins, such as α‐synuclein, LRRK2 and DJ‐1, were also found in MAM fractions (Table S6). To identify potential MAM protein complexes, we conducted a search in protein complex databases. A complex was considered as a potential MAM protein complex if all its constituent proteins were detected in our MAM proteomics analysis. In total, 180 protein complexes fulfilled this criterion (Table S7). Among these complexes, we found a portion of promising MAM complexes, for example, Amfr‐Vcp‐Ubxn1 complex, Dnm1l‐Mff complex and SNARE complex, which have been indicated in endoplasmic reticulum‐associated degradation (ERAD), cytoskeleton organization, signaling pathway, and so forth. Further research is necessary to explore whether these protein complexes are genuinely assembled and function as effective units within the MAM component. PCA and PLS‐DA methods were then applied to show data distribution. For PCA analysis, we could see that MAM samples for each group were located within the 95% confidence interval, and exhibited a relatively tight clustering (Figure 3c). The first principal component, which captured the largest amount of variance in the data set, accounted for 28.41% of the total variability between saline and MPTP‐treated mice, while the second principal component accounted for 16.41%. Three out of five test samples from the two groups were distinctly distributed across the positive and negative regions of the first principal component. However, there was a large overlap between them. The reasons could be that, first, in our MAM proteomic analysis, saline and MPTP group shared a total of 7270 common proteins with a relatively small number of proteins being uniquely detected in either group. Additionally, among these detected proteins, only 158 proteins were designated as DEPs (Table S8). The above findings might account for the substantial overlap observed in the current PCA analysis results. The plot of PLS‐DA, which was a supervised discriminant analysis, clearly revealed the distinct data distribution between the two groups (Figure 3d).

### 3.4 DEPs analysis in midbrain MAM proteomics

**FIGURE 4:**
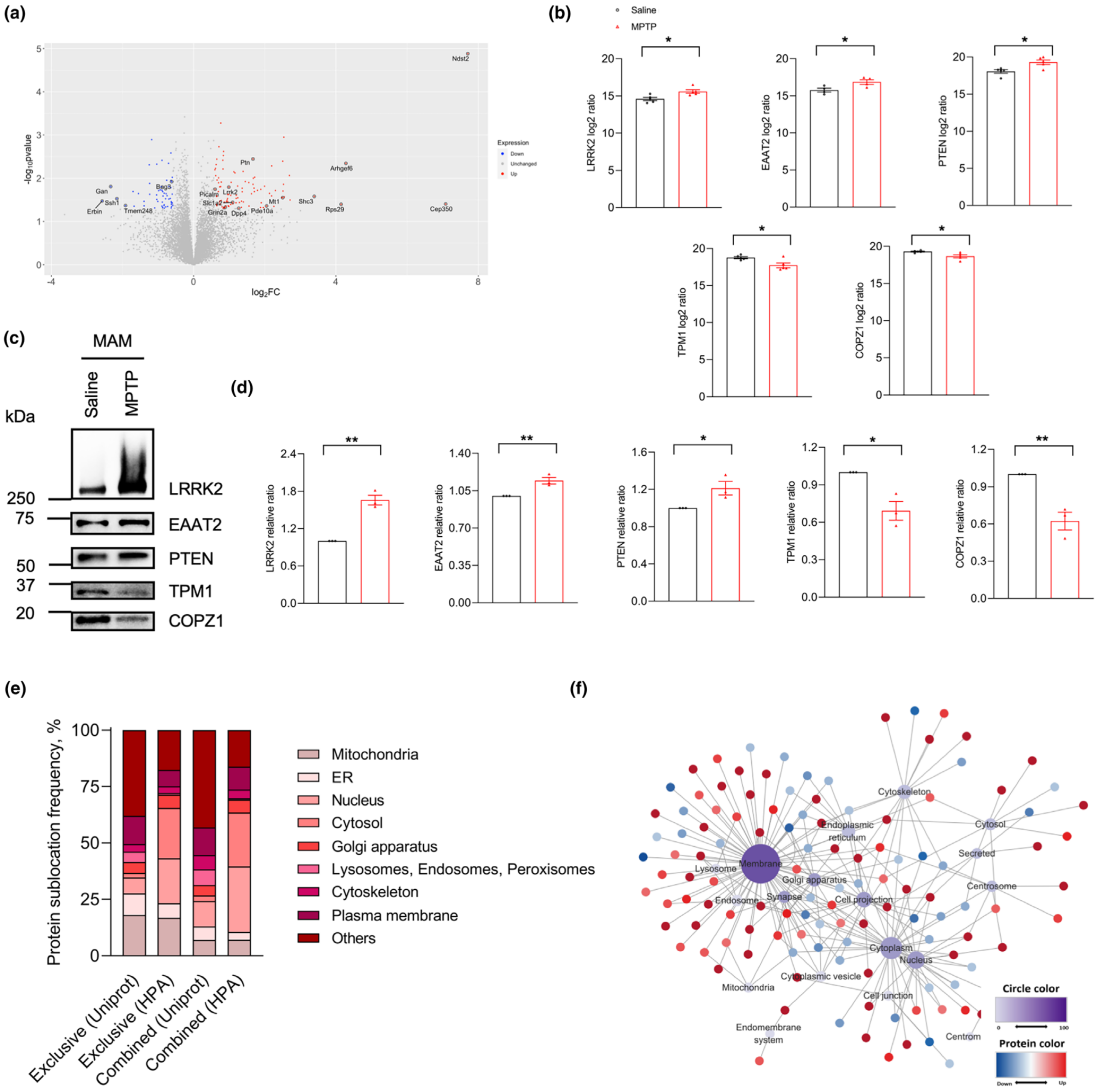
DEPs analysis in midbrain MAM proteomics. (a) Volcano plots in MAM proteomics. The cut‐off definitions of DEPs were FC >1.2 and *p* < 0.05. In volcano plots, red color meant up‐regulation, and blue color represented down‐regulation. Several mostly significant and potential disease related DEPs were labelled. (b) Quantifications analysis of the mass spec values for up‐regulated DEPs LRRK2, EAAT2 and PTEN, and down‐regulated DEPs TPM1 and COPZ1 in MAM proteomics. (c, d) Representative validation results of DEPs by WB approach (c) and grey value analysis (d) (*n* = 3 per group). LRRK2, EAAT2 and PTEN were proved to show up‐regulated trends. TPM1 and COPZ1 presented consistently down‐regulated changes in WB methods. (e) Percentage bar charts of protein localizations retrieved by Uniprot and HPA database. Exclusive results involved data from proteins with one single localization. Combined analysis aimed at involving the total subcellular information in proteomics. (f) Correlation graph of DEPs' cell positions searched by Uniprot database. The color depth of circle representing a certain location was connected with the number of linked proteins. Red or blue small circle identified up‐regulated or down‐regulated DEPs, respectively. The measurement data was presented as the form of means ± SEM, unpaired two‐tailed t test; * < 0.05, **p < 0.01.

Normal distribution of our MAM proteomic data was analyzed by Shapiro–Wilk methods. There were altogether 6722 out of 7295 identified proteins to be normally distributed in saline group, and the percentage rate was 92.15%. For MPTP group, there were altogether 6566 out of 7293 identified proteins to be normally distributed, and the percentage rate was 90.03% (Table S2). Since the majority of proteomic data was normally distributed, *t*‐test rather than non‐parametric Wilcoxon test was adopted in our study. In this research, DEPs were selected for *p* value being less than 0.05, and fold change being more than 1.2 after unpaired two‐tailed t test analysis of converted log_2_ value, similar to other MAM proteomic studies (Dematteis et al., 2020; Lu et al., 2022; Volgyi et al., 2018). In comparison with controls, there was a total of 103 up‐regulated and 55 down‐regulated DEPs in MPTP‐treated mice (Figure 4a, and Table S8). A portion of DEPs were further displayed in the heatmap (Figure S4a). In terms of PD related proteins, LRRK2 was significantly up‐regulated (Figure 4b), while α‐synuclein and DJ‐1 showed no significant differences. Several commonly recognized tethering molecules such as VDAC1, Calreticulin and Calnexin, were also found no differential changes in midbrain MAM proteomics. In order to validate the data accuracy, several DEPs were tested by western blot (Figure 4b–d). Specifically, proteins such as LRRK2, EAAT2, and PTEN were identified as up‐regulated DEPs in MAM proteomic analysis (Figure 4b). Subsequent WB analysis confirmed these trends, showing consistent up‐regulation performances (Figure 4c,d). The down‐regulated DEPs TPM1 and COPZ1 were also decreased in MPTP‐treated mice in the results of western blot (Figure 4b–d). Additionally, other key MAM proteins such as Calnexin, VDAC1 and DJ‐1 were non‐significantly different by both proteomic analysis and WB methods (Figure S4b,c). These findings revealed a degree of consistency between the results from western blot and MAM proteomics.

The subcellular localization of all detected proteins was retrieved by searching Uniprot and HPA databases respectively (Figure 4e, and Table S9). For 158 DEPs, the dominant intracellular localizations were membrane component (30.45%), cytoplasm (13.58%), nucleus (9.05%), cell projection (7.00%), cytoskeleton (5.76%), Golgi apparatus (4.94%), ER (4.94%), and synapse (3.70%) (Figure 4f). 51.33% of up‐regulated DEPs and 55.91% of down‐regulated DEPs were mainly localized in membrane component, cytoplasm and nucleus. Bioinformatic analysis such as GO analysis was also applied to reveal alterations about biological process, cellular component and molecular function for DEPs. In biological process analysis, 158 DEPs were mainly enriched in microtubule organization, synapse and vesicles, and cell responses to stimuli (Figure S5a, and Table S10). The results of cellular component analysis showed that membrane, cytoplasm, and cell projection were three major localizations for DEPs, consistent with previous findings (Dematteis et al., 2020; Lu et al., 2022; Poston et al., 2013). For molecular functions, cytoskeletal protein bindings and metabolite bindings were the major outcomes (Figure S5b, and Table S10).

### 3.5 GSEA analysis in midbrain MAM proteomics

**FIGURE 5:**
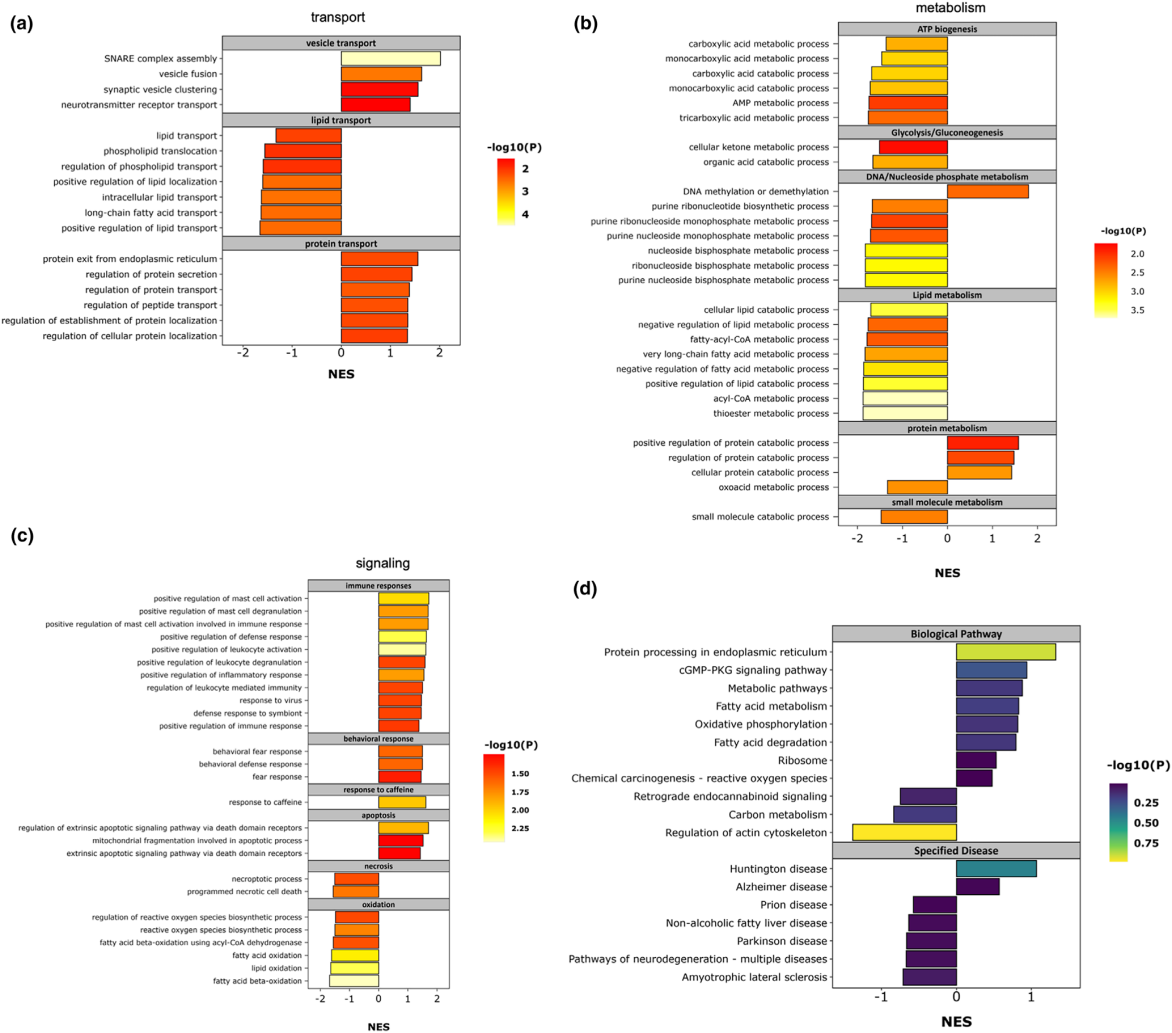
GSEA analysis in midbrain MAM proteomics. (a–c) Bar graphs of NES values from mainly enriched BP terms in transport pathways (a), metabolism pathways (b) and signaling pathways (c) after GSEA analysis. The color of each bar was associated with the *p* value from a certain BP pathway. (d) For consensus MAM proteins, the bar graph of NES values for mainly enriched KEGG pathways after GSEA analysis. The color of each bar was associated with the *p* value from a certain KEGG pathway.

Another bioinformatic analysis, GSEA methods, were then applied (Table S11). One advantage of GSEA analysis is that it incorporates not only the proteins types, but also their fold changes into the bioinformatic analysis. In our study, MPTP‐treated group was set to compare with control group. In the results of biological process analysis, identified MAM proteins were manually summarized to be mainly enriched in aspects of transport (e.g., lipid transport, vesicle transport and protein transport), metabolism, signaling (e.g., immune responses, apoptosis and oxidation) and organelle organization (Table S12). Specifically, in terms of transport pathways, protein associated transport processes tended to be up‐regulated in MPTP‐treated mice, while most lipid associated terms were found down‐regulated (Figure 5a). In metabolism processes, lipid metabolism, nucleic acid metabolism, adenosine triphosphate (ATP) generation, protein metabolism, glycolysis and gluconeogenesis, and so forth, were shown to be significantly down‐regulated in MPTP‐induced PD mouse models (Figure 5b). As for signaling processes, up‐regulated biological pathways were related to immune reaction, behavioral response and apoptosis (Figure 5c). Other signaling pathways such as oxidation reaction and necrosis process were significantly down‐regulated in MPTP‐treated mice.

In addition, Lu's study summarized previously‐published proteomic data of MAM fractions, and identified a total of 216 MAM conserved proteins (Lu et al., 2022). In our study, we identified 209 out of these 216 MAM conserved proteins (Figure 3b, and Table S13). Under different conditions, MAM protein types, fold changes, and related biological pathways could change variably. We were interested in how consensus MAM proteins and related pathways change during PD development. Therefore, we performed GSEA analysis for 209 consensus MAM proteins (Table S14). KEGG analysis featured a certain quantity of neurodegenerative disease (NDs) entries including Alzheimer's disease (AD), PD, Huntington's disease (HD), prion disease and Amyotrophic lateral sclerosis (ALS) (Figure 5d). Even though the *p*‐value did not reach significance and NES values were not remarkably changed, the enriched results indicated the potential link between MAM homeostasis and the pathophysiology of neurodegenerative diseases. By subsequent PPI network analysis, associated KEGG pathways were further clustered into three major groups including metabolism (amino acid metabolism, fatty acid metabolism, etc.), bioenergetic process, and protein synthesis and processing (Figure S6a, and Table S15). Specifically, oxidative phosphorylation (OXPHOS), reactive oxygen species (ROS) generation, calcium ion reabsorption and tricarboxylic acid (TCA) cycle were grouped under bioenergetic process. It was shown that in MPTP‐treated mice, most consensus MAM proteins mediating amino acid metabolism and protein processing tended to be down‐regulated in comparison with control groups. For biological process results, eight categories of functions (cytoskeleton regulation, oxidative phosphorylation, protein folding and processing, clathrin‐mediated endocytosis, tricarboxylic acid cycling, fatty acid oxidation, vesicle‐mediated molecular transport from ER, and protein synthesis) were manually summarized and visually presented by K‐means clustering methods (Figure S6B, and Table S16). These categories indicated the basic molecular pathways for MAM consensus proteins, and provided guidance for the subsequent focus on the function of a certain protein or a certain pathway, especially in the pathogenesis of PD.

## 4 DISCUSSION

Over the recent years, a number of experimental evidences have indicated that the dysregulation of MAM could be a significant hub in the pathogenesis of Parkinson's disease (Markovinovic et al., 2022). In previous literatures, in vitro PD models were mostly applied for studying MAM morphology and biological functions (Basso et al., 2018; Grossmann et al., 2019; Paillusson et al., 2017). To better simulate the disease conditions, in our study, subacute MPTP‐treated PD mouse model was applied. This PD mouse model features parkinsonian motor impairment and the loss of nigrostriatal neurons. The convenient injection methods also facilitate MPTP‐treated mice to be popular in vivo disease models. It is speculated that the abnormal MAM morphology could somehow further affect MAM bioactivities (and vice versa), exacerbating the pathological development of PD. In Basso's study, the movement abilities in disease drosophila were largely improved by over‐expressing MAM linkers (Basso et al., 2018). In this study, we revealed the morphological changes in MPTP‐induced PD mouse models. There was loosened, shortened and reduced MAM tethering in substantia nigral neurons from MPTP‐treated mice. Striatal neurons were also affected in term of number of MAMs per mitochondria and coverage percentage.

Quantitative proteomics and bioinformatic analysis were then applied to reveal the underlying features of midbrain MAM fractions. Up to now, studies on MAM proteomics have been reported in physiological conditions (Poston et al., 2013; Wang et al., 2018), infectious diseases (Horner et al., 2015; Zhang et al., 2011), ageing status (Lu et al., 2022), metabolic disease (mainly diabetes mellitus) (Ma et al., 2017; Wang et al., 2022; Xu et al., 2020), and neurological diseases (mainly AD) (Dematteis et al., 2020; Volgyi et al., 2018). It was commonly considered that MAM fractions were highly conserved across tissues and species (Poston et al., 2013; Wang et al., 2018). However, there were also articles pointing out that cortex tissues from preclinical stages of APP/PS1 mice (Volgyi et al., 2018), and immortalized hippocampal astrocytes from 3xTg mice (Dematteis et al., 2020) shared quite few common proteins in MAM proteomics. These findings indicated that variations of disease model, tissue site and cell type, could partly influence MAM proteomic results. As for PD, related proteomic explorations have not been reported yet. In our study, we separated and validated midbrain MAM fractions following the well‐established isolation protocol (Wieckowski et al., 2009). After UHPLC–MS/MS analysis, there were more than 7000 proteins identified. In comparison with other proteomic data that encompassed whole brain MAM fractions, our study shared 577 out of 1204 total proteins with Poston's study (Poston et al., 2013), 1002 out of 1312 total proteins with Ma's study (Ma et al., 2017), and 980 out of 2478 total proteins with Wang's study (Wang et al., 2018), separately. Völgyi's study (Volgyi et al., 2018) identified 5957 MAM proteins in the cortex of APP/PS1 Alzheimer's disease mouse models. Regrettably, complete proteomic data from this study was not accessible. Due to the diversity of proteomic approaches, brain regions and disease conditions, our study identified a subset of common proteins while also revealing large variations.

In midbrain MAM proteomics, we performed analysis of data normality and correction for multiple comparison. However, no DEPs remained statistically significant after correction analysis. In viewing of the fact that the Benjamini and Hochberg (BH) approach tended to be overly conservative and non‐sensitive to data internal structure, strictly adhering to the thresholds of statistical significance could probably miss potentially important biological information. Therefore, we followed related proteomics articles to define DEPs by the initial *p*‐value after t tests (Dematteis et al., 2020; Lu et al., 2022; Volgyi et al., 2018). Among 158 DEPs, up‐regulated proteins LRRK2, PTEN and EAAT2, and down‐regulated proteins TPM1 and COPZ1 were further validated by WB methods. WB validation not only substantiated our proteomic data, but also underscored the possibility that stringent statistical criteria of BH methods in small sample size might sometimes disregard important biologically relevant information.

The reasons for choosing proteins above were that these proteins were reported to be largely (LRRK2) or potentially (PTEN, EAAT2, TPM1, and COPZ1) associated with Parkinson's disease, and their roles on MAM homeostasis have not been fully understood. Specifically, as a PD associated protein, LRRK2 mediates various biological processes, for example, vesicle and neurotransmitter transport, lysosome function and autophagy, mitochondria homeostasis, cytoskeleton regulation, and neuroinflammation reaction. The pathological mutation form, LRRK2 G2019S could result in reduction of mitophagy and abnormality of mitochondria deoxyribonucleic acid (DNA) clearance, contributing to mitochondria dysfunction. Recent discoveries also stated that both LRRK2 WT and LRRK2 G2019S exacerbated the disruption of MAM ultrastructure (Toyofuku et al., 2020). In our study, WB validation showed increased level of LRRK2 in MAM fractions, aligning with proteomic results. Combined with the related articles previously reported, the consistently increased MAM LRRK2 could probably interfere with MAM formation, and affect MAM mediated biofunctions in the pathogenesis of PD. Meanwhile, acting as a tumor suppressor, PTEN was mostly focused on cell proliferation, DNA repair, and related hormone modulations in neurodegenerative diseases especially Alzheimer's disease. In PD, PTEN was found to be involved in ROS production and neuronal death in in vitro models (Zhu et al., 2007). It was recently reported that the mitochondrial hydrogen peroxide produced by PD toxins, could also activate PTEN, contributing to autophagy inhibition‐dependent neuronal apoptosis (Yu et al., 2023). In our MAM proteomics, PTEN was significantly up‐regulated in both proteomic and WB results. Such finding indicated that in MPTP treatment, PTEN might play a role in mediating MAM dysfunction and promoting neuronal death. However, there have not been direct evidences linking PTEN and MAM homoeostasis. Further investigation is needed for better understanding the molecular mechanisms involved. Other DEPs such as EAAT2 (Alijanpour et al., 2022), TPM1 (Hill‐Burns et al., 2016) and COPZ1 (Santiago et al., 2018), were also previously reported to be potentially associated with PD pathogenesis. It is worthwhile to further validate related MAM proteins and untangle the underlying mechanisms between MAM and PD.

In addition, other PD closely related proteins, such as α‐synuclein and DJ‐1, were also detected in midbrain MAM proteomics. These proteins have been reported to reside on this site, participating in the maintenance of MAM assembly and function. For example, by over‐expression of WT α‐synuclein or mutated α‐synuclein A53T, cells were discovered to exhibit enhanced mitochondrial fission, reduced ATP generation, impaired calcium flow, and reduced formation of ER‐mitochondria tethering (Di Maio et al., 2016; Paillusson et al., 2017). Over‐expression of WT α‐synuclein could contribute to the dislocation of α‐synuclein from MAM to cytoplasm (Cali et al., 2012), or interfere with the formation of VAPB‐PTPIP51 complex, participating in MAM abnormality (Paillusson et al., 2017). As for DJ‐1, the loss of DJ‐1 could be accompanied with abnormal mitochondrial structure, increased ROS level, disruption of mitochondrial complex I and aberrant calcium flow. Meanwhile, DJ‐1 was found to interact with p53 or nrf2, mediating the homeostasis of mitochondria (Holmström et al., 2016; Sarkar & Singh, 2023). In our previous work, DJ‐1 was shown to interact with IP3R3‐Grp75‐VDAC1, regulating the formation of MAM ultrastructure and related biological functions (Liu et al., 2019). However, we did not find differential expression of α‐synuclein or DJ‐1 in MAM proteomic analysis. Considering their essential functions in preserving MAM homeostasis, it is crucial to explore whether these proteins exhibit alterations across various PD models. Additionally, it is important to investigate if protein modifications, interactions, distributions, rather than overall levels, could affect MAM changes and the progression of the disease.

For bioinformatic analysis of 158 DEPs, the results of cellular component analysis showed that membrane, cytoplasm, and cell projection were three major localizations. In Poston's study, the top one subcellular site was also the plasma membrane (24%) (Poston et al., 2013). The explanations could be the consequence of membrane contact sites between plasma membrane and ER, or a larger membrane network of plasma membrane, ER, and mitochondria (Lebiedzinska et al., 2009). For proteins mainly targeting at cytoplasm and nucleus, validation research might be needed to investigate whether these proteins are newly synthesized and awaiting transportation, previously unrecognized MAM proteins, or artifacts due to inherent limitations in the current methodologies (Lu et al., 2022). Additionally, related studies have found that MAM was enriched in the contents of organelle composition entry, cell projection and synapse formation. A large number of MAM interconnections were also reported to exist in synapses, axons and dendrites (Leal et al., 2020). MAM‐mediated biological functions, such as calcium and mitochondrial homeostasis, are known to modulate neuronal excitability and synaptic activity (Mironov & Symonchuk, 2006). Therefore, the potential connections between ER‐mitochondria contacts and synapse during PD development might be one of the future investigation interests.

As for biological process analysis of 158 DEPs, it was noticed that MAM bioactivities seemed to cover the majority of pathways which were impaired during PD pathogenesis. For example, signaling pathways including immune reaction and apoptosis functions were strengthened in MPTP‐treated mice, while metabolism pathways such as ATP biogenesis and lipid metabolism were down‐regulated. It is acknowledged that ATP generation is closely relied on normal calcium flow and mitochondrial function. In our previous work, we proved that PD associated protein DJ‐1 was a MAM protein. DJ‐1 could interact with IP3R3‐Grp75‐VDAC1 complex, regulating the formation of MAM ultrastructure and related functions including calcium flow and ATP biogenesis (Liu et al., 2019). Thus, it is worth exploring that whether protein changes, protein complex formation, protein distribution or active form of specific proteins, could contribute to associated pathway changes, linking the observed MAM alterations and proteomic changes to PD pathogenesis.

In Lu's study, a total of 216 proteins were summarized and recognized as MAM conserved proteins (Lu et al., 2022). In our study, there were altogether 209 out of 216 consensus proteins to be detected, revealing a relatively high similarity of fraction compositions. The subsequent KEGG pathway results revealed that the potential relationship between neurodegenerative diseases and MAM, as previously reported. Among enriched items, several neurodegenerative diseases including Amyotrophic lateral sclerosis, Huntington's disease and prion disease have not been widely studied on MAM alterations. In the findings of biological process analysis, there were a part of newly clustered pathways such as peroxisome function, clathrin‐mediated endocytosis, and molecular transport from ER to Golgi apparatus, which further enriched our understanding of MAM‐mediated functions.

There were some limitations of this study. Firstly, the subacute MPTP mouse model did not show clear α‐synuclein deposition, and other non‐motor symptoms (Jackson‐Lewis & Przedborski, 2007). Another widely used PD mouse model, 6‐hydroxydopamine (6‐OHDA) model, could allow each experimental animal to compare with itself by the methods of unilateral intracerebral stereotactic injection. The degree of neuron loss could also be mediated by 6‐OHDA injection into specific anatomical regions. Such targeted, region‐specific studies of neurodegeneration could probably offer additional insights in exploring the pathological association between MAM and PD. Meanwhile, it would provide more compelling evidences if there were direct samples of human beings or induced pluripotent stem cells (iPSCs) studies from sporadic PD patients. Secondly, to ensure sufficient samples to study MAM proteomic changes, pooled midbrain tissues were applied for qualified MAM extraction. We also increased the sample size by performing sample mixing and repeated detections. Considering that each biological sample contained a mixture of eight independent midbrain tissues, the limitations of such a small sample size was partially mitigated. However, it should be noted that the use of pooled midbrain tissues introduced a degree of heterogeneity in MAM samples, which represented a limitation of this study. Future studies may aim to refine the extraction process to minimize this variability. Thirdly, in our study, data‐independent acquisition method was applied for quantitative proteomics. The advantages of this method were that it did not require stable isotope labeling, and was more complete and less‐random, leading to higher quantitative throughput and improved accuracy. Besides this, tandem mass tag (TMT)‐labelled proteomics is more suitable for complex protein samples and limited sample number, and is a more sensitive technique for detecting changes in protein expression, which could potentially reveal additional proteins affected in MPTP‐treated mice that were not identified in our study. Specially, in viewing that MAM being a specific membrane contact site, our MAM proteomic findings inevitably revealed partial pathways that were involved in the functioning of either ER or mitochondria. Future experiments involving non‐MAM factions as a control, such as pure mitochondria or ER fractions, will be of great benefit. Fourthly, the posttranslational modifications were not considered in MAM proteomics. The mechanisms of protein targeting and activity form also demand detailed evidences. Fifthly, our study was an observational exploration, and further investigations are required to elucidate the specific molecular and pathway mechanisms, as well as the precise role of MAM components.

In conclusion, we demonstrated disrupted MAM ultrastructure in MPTP‐treated mice, and revealed the underlying signatures of MAM by quantitative proteomics and bioinformatic analysis. To our knowledge, this was the first study to illustrate MAM morphology and proteomic profiling in MPTP‐induced PD mouse models. Considering that our study was an observational exploration, further in‐depth investigations focusing on MAM ultrastructure alterations, MAM protein composition and activity form, MAM‐mediated biological pathways and their role in PD progression, are needed and worth looking forward to, which will probably be of great help to better understand the pathogenesis of PD.

## AUTHOR CONTRIBUTIONS

J. L. performed associated experiments. Y. L., C. G., H. P. and P. H. performed proteomic analysis. J. L. and Y. L. drafted the manuscript. S. C. and Y. T. designed and supervised this study, and revised the manuscript. All authors read, and approved the final manuscript.

## CONFLICT OF INTEREST STATEMENT

The authors declared no inflict of interests to report.

## Supporting information


**Figure S1.** Procedure information of initial MAM proteomic analysis. (a) Protein concentrations of independent MAM biological samples from saline and MPTP groups (*n* = 3 per group). (b) Flow diagram of the processing procedure of initial MAM proteomics (*n* = 8 per group). Sample size was increased by performing sample mixing and repeated detections. Selected samples for further analysis were followed by a red tick in the flow diagram. (c) Sample distribution plots by PLS‐DA analysis in initial MAM proteomics. Three sets of data from each group were excluded due to dispersed distribution, as these results might introduce potential data bias. The finally applied samples (5 vs. 5) were circled by a black box in each group.

**Figure S2.** The disrupted mitochondrial ultrastructure from substantia nigral neurons in MPTP‐treated mice. (a–d) Quantitative analysis of average mitochondrial area (a), average mitochondrial length (b), average aspect ratio (c), and average cristae score (d) in substantia nigral neurons between controls (mitochondria *n* = 307) and MPTP‐treated mice (mitochondria *n* = 353) (mouse *n* = 3 per group). The measurement data was presented as the form of means ± SEM, unpaired two‐tailed *t* test; ***p < 0.001, ****p < 0.0001.

**Figure S3.** The disrupted MAM ultrastructure from striatal neurons in MPTP‐treated mice. (a–d) Quantitative analysis of average MAM thickness as the shortest vertical distance between ER and mitochondrial outer membrane (a), average MAM length as ER length which apposed to mitochondrial outer membrane within 30 nm thickness (b), average MAM number existed per mitochondria (c), and average MAM coverage percentage as coverage percentage of mitochondria surface forming close contacts with ER (d) in striatal neurons between controls (mitochondria *n* = 358) and MPTP‐treated mice (mitochondria *n* = 343) (mouse *n* = 3 per group). The measurement data was presented as the form of means ± SEM, unpaired two‐tailed t test; **p < 0.01.

**Figure S4.** Basic analysis in MAM proteomics. (a) Heatmap for DEPs in midbrain MAM proteomics. These DEPs obtained complete data values in all samples. Red color meant up‐regulation, and blue color represented down‐regulation. (b, c) Representative validation results of non‐DEPs by WB approach (b) and grey value analysis (c) (*n* = 3 per group). Calnexin, VDAC1 and DJ‐1 were consistently unchanged in WB methods. The measurement data was presented as the form of means ± SEM, unpaired two‐tailed *t* test.

**Figure S5.** GO analysis of DEPs in MAM proteomics. (a, b) Bubble graph of enriched biological process terms (a) and molecular function terms (b) for DEPs after GO analysis.

**Figure S6.** Bioinformatic analysis of consensus MAM proteins in MAM proteomics. (a) PPI network graph for enriched KEGG pathways of consensus MAM proteins after GSEA analysis. Red color meant up‐regulation, and blue color represented down‐regulation. (b) K‐means functionally cluster map of biological process terms for consensus MAM proteins after GSEA analysis.

**Table S1.** Statistical information of MAM and mitochondria in control and MPTP‐treated mice.

**Table S2.** Total proteomic data in MAM proteomics.

**Table S3.** Information of protein sequence percentage coverage in MAM proteomics.

**Table S4.** List of proteins identified based on a single unique peptide in MAM proteomics.

**Table S5.** List of DEPs and consensus MAM proteins identified based on a single unique peptide in MAM proteomics.

**Table S6.** List of PD related proteins in MAM proteomics.

**Table S7.** Information of potential protein complexes in MAM proteomics.

**Table S8.** List of DEPs in MAM proteomics.

**Table S9.** Information of protein subcellular localizations in MAM proteomics.

**Table S10.** Results of GO and KEGG analysis for DEPs in MAM proteomics.

**Table S11.** GSEA data for all detected proteins in MAM proteomics.

**Table S12.** Summary of enriched BP terms in MAM proteomics.

**Table S13.** List of consensus MAM proteins in MAM proteomics.

**Table S14.** GSEA data for consensus MAM proteins in MAM proteomics.

**Table S15.** PPI network information of KEGG pathways for consensus MAM proteins in MAM proteomics.

**Table S16.** K‐means functionally clustered information of BP terms for consensus MAM proteins in MAM proteomics.

## Data Availability

The mass spectrometry proteomic data have been deposited to the ProteomeXchange Consortium (https://proteomecentral.proteomexchange.org) via the iProX partner repository (Chen et al., 2022; Ma et al., 2019) with the dataset identifier PXD051805.
