# Supplementary figures and images for: The ultrastructural and proteomic analysis of mitochondria‐associated endoplasmic reticulum membrane in the midbrain of a Parkinson's disease mouse model
Source: Aging Cell. 2024 Nov 29;24(4):e14436. doi: 10.1111/acel.14436 (PMC11984660; doi:10.1111/acel.14436)

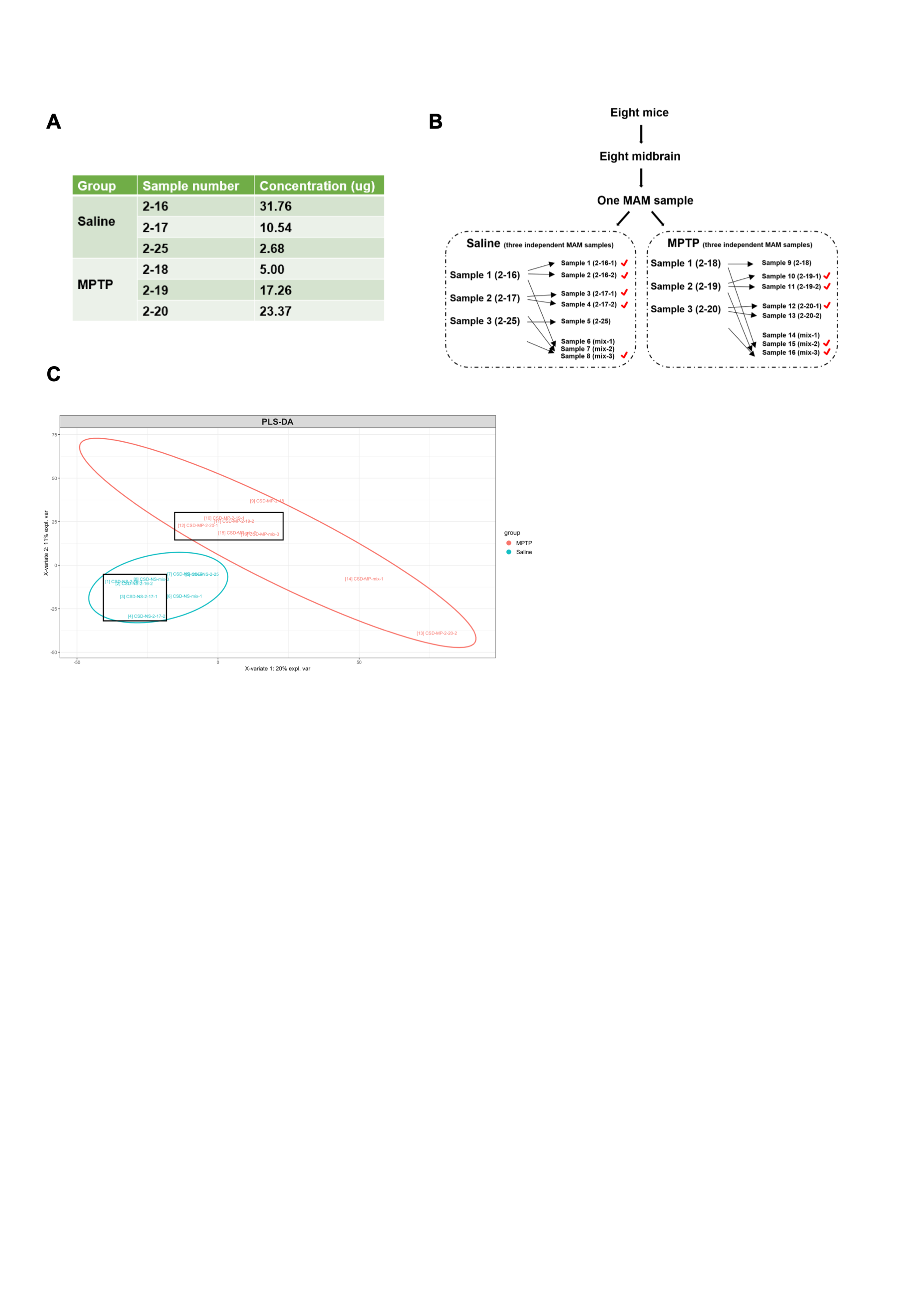

Supplement: Supplementary file 1 — Figure S1. Procedure information of initial MAM proteomic analysis. (a) Protein concentrations of independent MAM biological samples from saline and MPTP groups (n = 3 per group). (b) Flow diagram of the processing procedure of initial MAM proteomics (n = 8 per group). Sample size was increased by performing sample mixing and repeated detections. Selected samples for further analysis were followed by a red tick in the flow diagram. (c) Sample distribution plots by PLS‐DA analysis in initial MAM proteomics. Three sets of data from each group were excluded due to dispersed distribution, as these results might introduce potential data bias. The finally applied samples (5 vs. 5) were circled by a black box in each group. [file ACEL-24-e14436-s005.tiff]

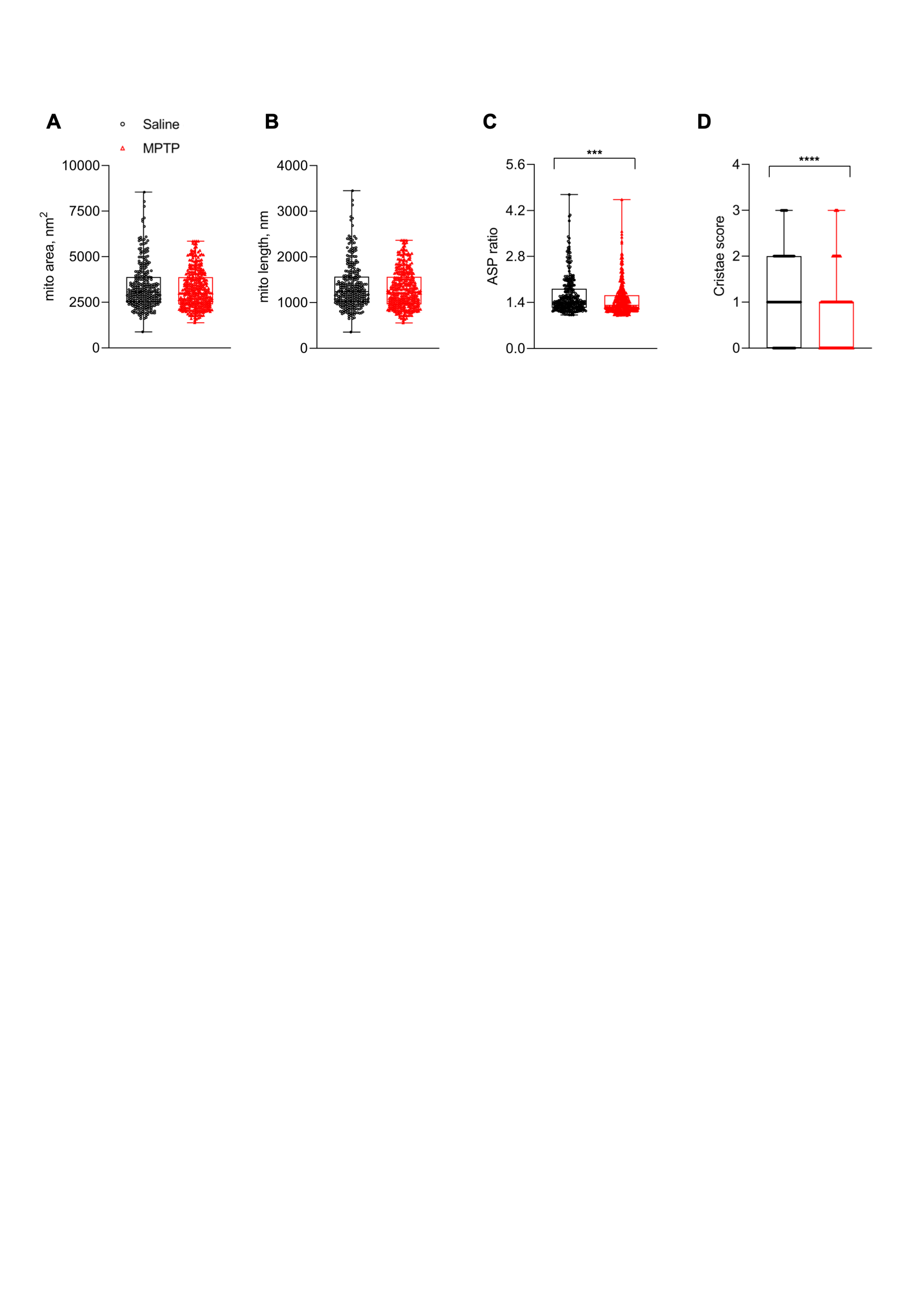

Supplement: Supplementary file 2 — Figure S2. The disrupted mitochondrial ultrastructure from substantia nigral neurons in MPTP‐treated mice. (a–d) Quantitative analysis of average mitochondrial area (a), average mitochondrial length (b), average aspect ratio (c), and average cristae score (d) in substantia nigral neurons between controls (mitochondria n = 307) and MPTP‐treated mice (mitochondria n = 353) (mouse n = 3 per group). The measurement data was presented as the form of means ± SEM, unpaired two‐tailed t test; ***p < 0.001, ****p < 0.0001. [file ACEL-24-e14436-s017.tiff]

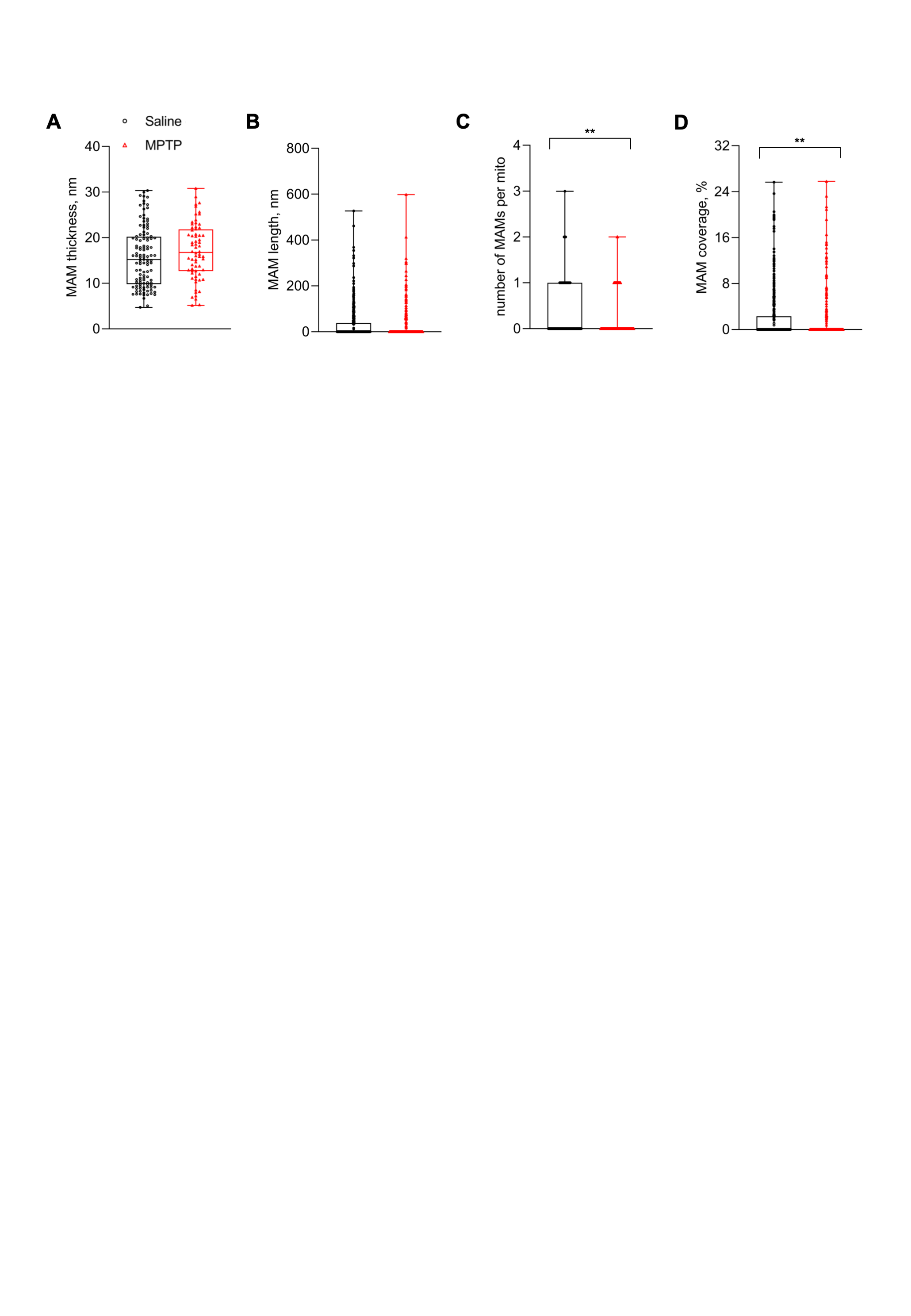

Supplement: Supplementary file 3 — Figure S3. The disrupted MAM ultrastructure from striatal neurons in MPTP‐treated mice. (a–d) Quantitative analysis of average MAM thickness as the shortest vertical distance between ER and mitochondrial outer membrane (a), average MAM length as ER length which apposed to mitochondrial outer membrane within 30 nm thickness (b), average MAM number existed per mitochondria (c), and average MAM coverage percentage as coverage percentage of mitochondria surface forming close contacts with ER (d) in striatal neurons between controls (mitochondria n = 358) and MPTP‐treated mice (mitochondria n = 343) (mouse n = 3 per group). The measurement data was presented as the form of means ± SEM, unpaired two‐tailed t test; **p < 0.01. [file ACEL-24-e14436-s008.tiff]

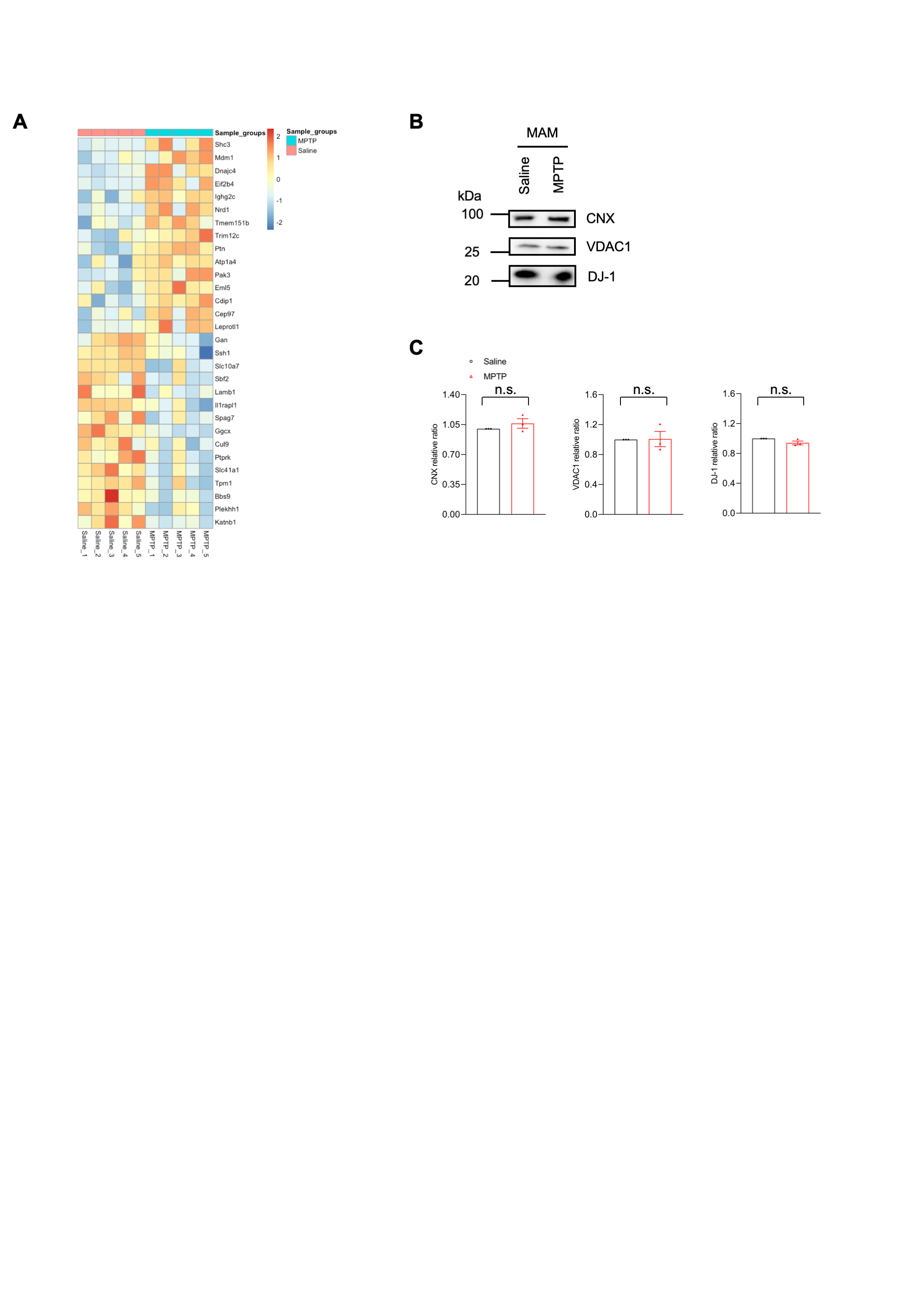

Supplement: Supplementary file 4 — Figure S4. Basic analysis in MAM proteomics. (a) Heatmap for DEPs in midbrain MAM proteomics. These DEPs obtained complete data values in all samples. Red color meant up‐regulation, and blue color represented down‐regulation. (b, c) Representative validation results of non‐DEPs by WB approach (b) and grey value analysis (c) (n = 3 per group). Calnexin, VDAC1 and DJ‐1 were consistently unchanged in WB methods. The measurement data was presented as the form of means ± SEM, unpaired two‐tailed t test. [file ACEL-24-e14436-s022.tiff]

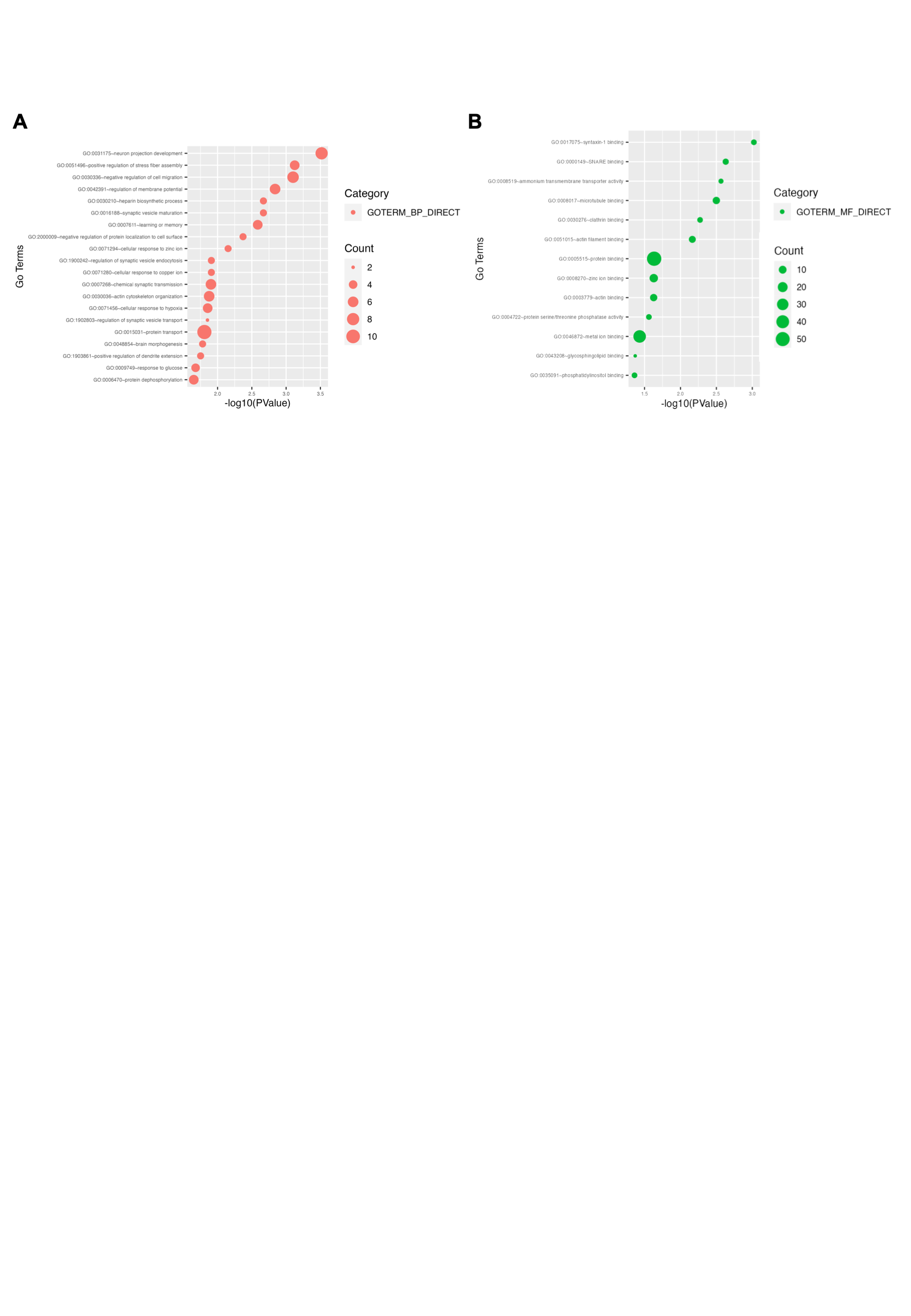

Supplement: Supplementary file 5 — Figure S5. GO analysis of DEPs in MAM proteomics. (a, b) Bubble graph of enriched biological process terms (a) and molecular function terms (b) for DEPs after GO analysis. [file ACEL-24-e14436-s019.tiff]

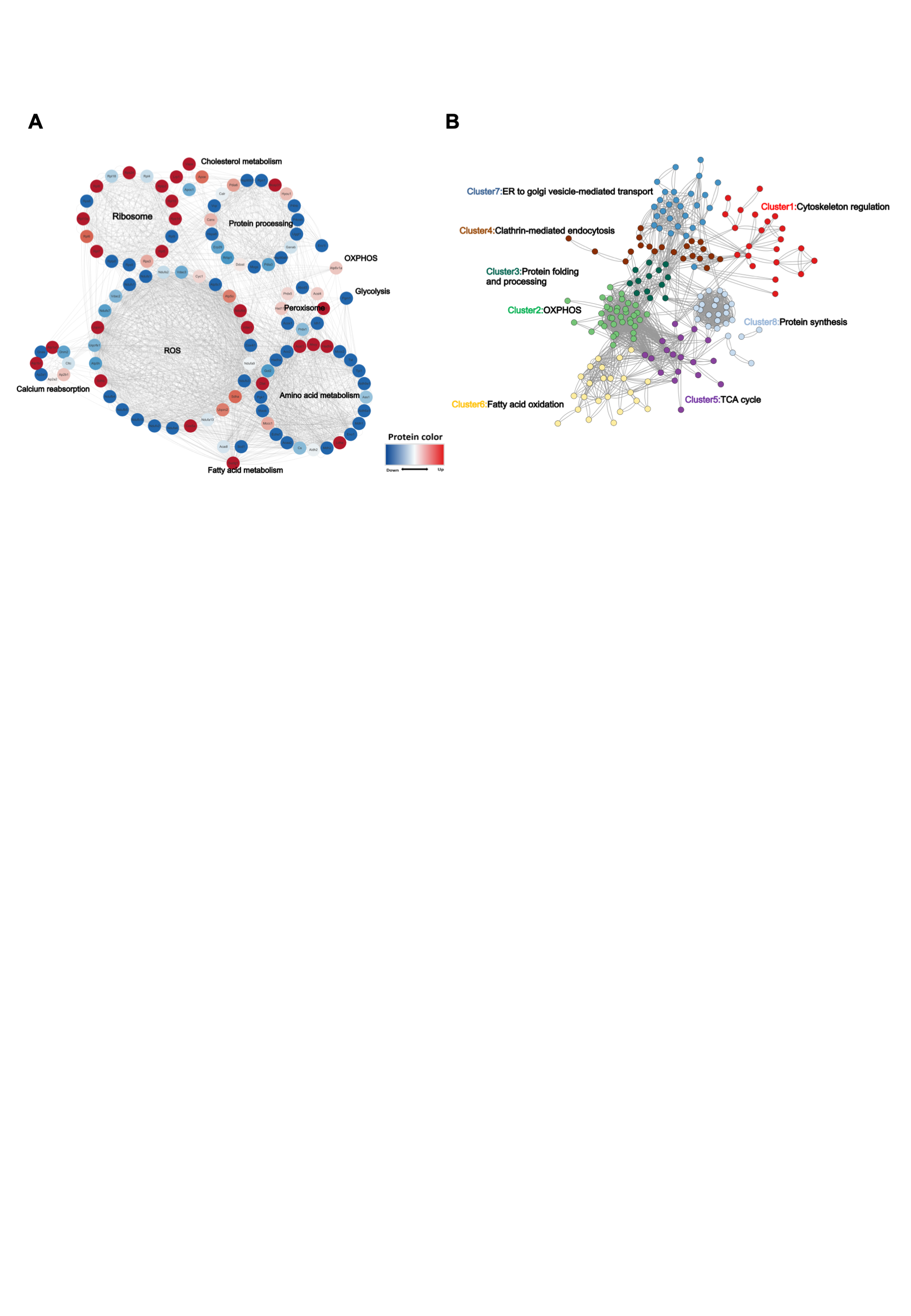

Supplement: Supplementary file 6 — Figure S6. Bioinformatic analysis of consensus MAM proteins in MAM proteomics. (a) PPI network graph for enriched KEGG pathways of consensus MAM proteins after GSEA analysis. Red color meant up‐regulation, and blue color represented down‐regulation. (b) K‐means functionally cluster map of biological process terms for consensus MAM proteins after GSEA analysis. [file ACEL-24-e14436-s013.tiff]
