# Supplementary material for: The ultrastructural and proteomic analysis of mitochondria‐associated endoplasmic reticulum membrane in the midbrain of a Parkinson's disease mouse model
Source: Aging Cell. 2024 Nov 29;24(4):e14436. doi: 10.1111/acel.14436 (PMC11984660; doi:10.1111/acel.14436)
Supplement: Supplementary file 7 — Table S1. Statistical information of MAM and mitochondria in control and MPTP‐treated mice. [file ACEL-24-e14436-s012.docx]

**Supplementary Table 1** **Statistical information of MAM and mitochondria in control and MPTP-treated mice**

| Group  Parameter | SN | | Striatum | |
| --- | --- | --- | --- | --- |
|  | Saline | MPTP | Saline | MPTP |
| Mitochondria number | 307 | 353 | 358 | 343 |
| MAM number | 174 | 139 | 116 | 68 |
| MAM thickness (nm, mean ± SEM) | 12.685 (0.361) | 15.020 (0.438) | 15.733 (0.601) | 17.029 (0.737) |
| Super tight type (n, %) | 90 (51.724) | 46 (33.094) | 43 (37.069) | 15 (22.059) |
| Tight type (n, %) | 79 (45.402) | 83 (59.712) | 58 (50.000) | 45 (66.176) |
| Loose type (n, %) | 5 (2.874) | 10 (7.194) | 15 (12.931) | 8 (11.765) |
| MAM length (nm, mean ± SEM) | 193.551 (9.874) | 184.618 (10.271) | 100.186 (7.201) | 119.437 (11.892) |
| Short type (n, %) | 112 (64.368) | 91 (65.468) | 109 (93.966) | 58 (85.294) |
| Medium type (n, %) | 47 (27.011) | 39 (28.058) | 5 (4.310) | 8 (11.765) |
| Long type (n, %) | 15 (8.621) | 9 (6.475) | 2 (1.724) | 2 (2.941) |
| MAM coverage (%, mean ± SEM) | 8.436 (0.700) | 5.586 (0.530) | 2.144 (0.242) | 1.397 (0.211) |
| Number of MAMs per mitochondria | 0.567 (0.039) | 0.394 (0.031) | 0.324 (0.030) | 0.198 (0.023) |
| Mitochondria area (nm^2^, mean ± SEM) | 3347.615 (68.635) | 3205.818 (54.176) | 3869.243 (77.547) | 4048.848 (71.849) |
| Mitochondria length (nm, mean ± SEM) | 1352.013 (27.730) | 1294.694 (21.887) | 1562.687 (31.333) | 1635.284 (29.025) |
| Mitochondria ASP ratio (mean ± SEM) | 1.625 (0.033) | 1.487 (0.026) | 1.558 (0.038) | 1.589 (0.028) |
| Mitochondria cristae score (mean ± SEM) | 1.127 (0.051) | 0.742 (0.042) | 1.601 (0.047) | 1.420 (0.054) |
| SN, substantia nigra; MPTP, 1-methyl-4-phenyl-1,2,3,6-tetrahydropyridine; MAM, mitochondria-associated ER membrane; SEM, standard error of mean; ASP, aspect. | | | | |
