# Supplementary material for: The ultrastructural and proteomic analysis of mitochondria‐associated endoplasmic reticulum membrane in the midbrain of a Parkinson's disease mouse model
Source: Aging Cell. 2024 Nov 29;24(4):e14436. doi: 10.1111/acel.14436 (PMC11984660; doi:10.1111/acel.14436)
Supplement: Supplementary file 12 — Table S6. List of PD related proteins in MAM proteomics. [file ACEL-24-e14436-s020.docx]

**Supplementary Table 6** **List of PD related proteins in MAM proteomics**

| Detected proteins | Undetected proteins |
| --- | --- |
| Snca | Pink1 |
| Park7 | Itga8 |
| Lrrk2 | Hla |
| Pacrg | Fbx07 |
| Vps35 | Park16 |
| Gba2 | Rab25 |
| Pla2g6 | Parkin |
| Atp13a2 |  |
| Mapt |  |
| Syt11 |  |
| Hip1r |  |
| Dgkq |  |
| Gak |  |
| Mccc1 |  |
| Stk39 |  |
| Tmem163 |  |
| Stx1b |  |
