# Supplementary material for: The ultrastructural and proteomic analysis of mitochondria‐associated endoplasmic reticulum membrane in the midbrain of a Parkinson's disease mouse model
Source: Aging Cell. 2024 Nov 29;24(4):e14436. doi: 10.1111/acel.14436 (PMC11984660; doi:10.1111/acel.14436)
Supplement: Supplementary file 13 — Table S7. Information of potential protein complexes in MAM proteomics. [file ACEL-24-e14436-s007.docx]

**Supplementary Table 7 Information of potential protein complexes in MAM proteomics**

| Complex ID | Complex Name | Subunits (UniProt IDs) | Subunits (Entrez IDs) | GO description |
| --- | --- | --- | --- | --- |
| 141 | Sos1-Abi1-Eps8 complex | Q08509;Q62245;Q8CBW3 | 13860;20662;11308 | small GTPase mediated signal transduction |
| 307 | Dtnb-Sntb2-Utrn complex | E9Q6R7;O70585;Q61235 | 22288;13528;20650 | plasma membrane |
| 675 | Nrsn1-Tmed10 complex | P97799;Q9D1D4 | 22360;68581 | vesicle-mediated transport;endoplasmic reticulum;Golgi apparatus |
| 688 | Cd2ap-Fyn complex | P39688;Q9JLQ0 | 14360;12488 | protein binding |
| 690 | Cd2ap-Synpo complex | Q8CC35;Q9JLQ0 | 104027;12488 | protein binding;cytoskeleton organization |
| 931 | Scrib-Git1-Arhgef7 complex | Q68FF6;Q80U72;Q9ES28 | 216963;105782;54126 | exocytosis;plasma membrane;chemical synaptic transmission |
| 975 | Ocrl-Cdc42 complex | P60766;Q6NVF0 | 12540;320634 | intracellular protein transport;protein targeting;protein transport;Golgi apparatus |
| 990 | Agap1-Ap3b1-Ap3m1-Ap3m2-Ap3s2 complex | Q8BSZ2;Q8BXK8;Q8R2R9;Q9JKC8;Q9Z1T1 | 11778;347722;64933;55946;11774 | vesicle-mediated transport;endosome |
| 993 | Kif5b-Dtnb complex | O70585;Q61768 | 13528;16573 | intracellular protein transport;protein targeting;protein transport;cell motility |
| 1112 | Psd-Actn1 complex | Q5DTT2;Q7TPR4 | 73728;109711 | neuron projection development |
| 1115 | Cdk5-Cdk5r1 complex | P49615;P61809 | 12568;12569 | protein phosphorylation;nervous system development;astrocyte projection |
| 1157 | Cdh1-Cdh2-Ctnnb1-Psen1 complex | P09803;P15116;P49769;Q02248 | 12550;12558;19164;12387 | calcium ion binding;cell-cell junction organization;cell-cell junction assembly;cell-cell junction;cell-cell adhesion |
| 1449 | Dlg3-Lin7a-Dlg1 complex | P70175;Q811D0;Q8JZS0 | 53310;13383;108030 | intracellular protein transport;protein targeting;protein transport;anisotropic cell growth;axis specification |
| 1523 | Stat3-Crebbp complex | P42227;P45481 | 20848;12914 | brain development |
| 1549 | Kpna2-Kpnb1 complex | P52293;P70168 | 16647;16211 | protein transport;transport;nucleus;protein transporter activity |
| 1988 | Bcl2 homodimer complex | P10417 | 12043 | regulation of programmed cell death |
| 1995 | Sgk3 homodimer complex | Q9ERE3 | None | cell morphogenesis;cell growth |
| 2006 | Ptpra homodimer complex | P18052 | 19262 | enzyme inhibitor activity;cytoplasm |
| 2008 | Prnp homodimer complex | P04925 | 19122 | neuron differentiation |
| 2009 | Birc6 homodimer complex | O88738 | 12211 | negative regulation of apoptotic process |
| 2296 | Abi1-Wasl complex | Q8CBW3;Q91YD9 | 11308;73178 | actin cytoskeleton organization |
| 2360 | Itgav-Itgb3-Gsn complex | O54890;P13020;P43406 | 16416;227753;16410 | protein binding |
| 2415 | Itgb1-Rap1a-Prkd1 complex | P09055;P62835;Q62101 | 16412;109905;18760 | regulation of integrin activation |
| 2475 | Cbl-Crkl-Rapgef1 complex | P22682;P47941;Q91ZZ2 | 12402;12929;107746 | integrin-mediated signaling pathway;cell migration |
| 2548 | Egfr-Grb2-Sos1 complex | Q01279;Q60631;Q62245 | 13649;14784;20662 | G protein-coupled receptor signaling pathway;epidermal growth factor receptor signaling pathway |
| 2553 | Grb2-Shc1-Sos1 complex | P98083;Q60631;Q62245 | 20416;14784;20662 | G protein-coupled receptor signaling pathway;transmembrane receptor protein tyrosine kinase signaling pathway |
| 2710 | A2m-Anxa6-Lrp1 complex | P01023;P08133;P14824;Q07954;Q6GQT1;Q91ZX7 | 2;309;11749;4035;232345;16971 | receptor-mediated endocytosis |
| 2712 | Aph1a-Psen1-Ncstn complex | P49769;P57716;Q8BVF7 | 19164;59287;226548 | proteasome-mediated ubiquitin-dependent protein catabolic process;ubiquitin-dependent protein catabolic process;Notch signaling pathway;plasma membrane |
| 2777 | Ecsit complex (Ecsit2, Smad4), Bmp4 treated or untreated | P97471;Q9QZH6 | 17128;26940 | BMP signaling pathway |
| 2778 | Ecsit complex (Ecsit2, Smad1), Bmp4 treated | P70340;Q9QZH6 | 17125;26940 | BMP signaling pathway |
| 2779 | Ecsit complex (Ecsit2, Smad1, Smad4) | P70340;P97471;Q9QZH6 | 17125;17128;26940 | regulation of RNA biosynthetic process;regulation of DNA-templated transcription;DNA binding;nucleus;BMP signaling pathway;SMAD protein signal transduction |
| 2832 | Smad4-Smad2-Smad3 complex | P97471;Q62432;Q8BUN5 | 17128;17126;17127 | regulation of RNA biosynthetic process;regulation of DNA-templated transcription;transforming growth factor beta receptor signaling pathway;nucleus |
| 2999 | Smad2 homotrimer complex | Q62432 | 17126 | positive regulation of DNA-templated transcription;transforming growth factor beta receptor signaling pathway |
| 3000 | Smad2-Smad3 complex | Q62432;Q8BUN5 | 17126;17127 | positive regulation of DNA-templated transcription;transforming growth factor beta receptor signaling pathway |
| 3001 | Smad2-Smad4 complex | P97471;Q62432 | 17128;17126 | positive regulation of DNA-templated transcription;transforming growth factor beta receptor signaling pathway;SMAD protein signal transduction |
| 3002 | Smad3-Smad4 complex | P97471;Q8BUN5 | 17128;17127 | positive regulation of DNA-templated transcription;DNA binding;transforming growth factor beta receptor signaling pathway;nucleus;SMAD protein signal transduction |
| 3003 | Smad3 homotrimer complex | Q8BUN5 | 17127 | positive regulation of DNA-templated transcription;DNA binding;transforming growth factor beta receptor signaling pathway |
| 3130 | Tle3-Aes complex | P63002;Q08122 | 14797;21887 | negative regulation of DNA-templated transcription;protein binding;regulation of binding;regulation of DNA-binding transcription factor activity;nucleus |
| 3143 | Sos1-Abi1-Eps8 complex | Q08509;Q62245;Q8CBW3 | 13860;20662;11308 | small GTPase mediated signal transduction;transmembrane receptor protein tyrosine kinase signaling pathway |
| 3211 | Cask-Dlg1 complex | O70589;Q811D0 | 12361;13383 | protein targeting;protein transport;anisotropic cell growth;axis specification |
| 3741 | Rab27a-Sytl5 complex | Q80T23;Q9ERI2 | 236643;11891 | vesicle-mediated transport |
| 5003 | Vcp homohexamer complex | Q01853 | 269523 | proteasome-mediated ubiquitin-dependent protein catabolic process;ubiquitin-dependent protein catabolic process;retrograde transport, vesicle recycling within Golgi;vesicle fusion;endoplasmic reticulum unfolded protein response |
| 5158 | Smarca2-Smarce1-Mecp2 complex | O54941;Q6DIC0;Q9Z2D6 | 57376;67155;;17257 | chromatin remodeling;negative regulation of DNA-templated transcription;heterochromatin formation |
| 5252 | Dctn1-Hap1-Htt complex | O08788;O35668;P42859 | 13191;15114;15194 | vesicle-mediated transport;cytoskeleton-dependent intracellular transport;microtubule cytoskeleton |
| 5302 | Rab3a-Rims2-Rapgef4 complex | P63011;Q9EQZ6;Q9EQZ7 | 19339;56508;116838 | vesicle fusion;exocytosis |
| 5493 | Tmsb4x-Lims1-Ilk complex | O55222;P20065;Q99JW4 | 16202;19241;110829 | cell migration;response to stimulus |
| 5574 | Vps29-Vps35-Vps26a complex | P40336;Q9EQH3;Q9QZ88 | 30930;65114;56433 | intracellular protein transport;protein targeting;protein transport;retrograde transport, vesicle recycling within Golgi |
| 5578 | Sdc2-Ephb2 complex | P43407;P54763 | 15529;13844 | neuron differentiation |
| 5627 | Cdh23-Myo1c complex | Q99PF4;Q9WTI7 | 22295;17913 | ion channel activity;stereocilia tip link;sensory perception of sound |
| 5722 | Cbl-Cd2ap complex | P22682;Q9JLQ0 | 12402;12488 | protein binding;endocytosis;transport vesicle |
| 5723 | Cbl-Cd2ap-Flt1 complex | P22682;P35969;Q9JLQ0 | 12402;14254;12488 | protein ubiquitination;endocytosis;transmembrane receptor protein tyrosine kinase signaling pathway;angiogenesis;transport vesicle |
| 5886 | Ksr1 complex (Ksr1, Mek, 14-3-3), unstimulated | P31938;P63101;Q61097 | 26395;22631;16706 | protein phosphorylation;MAPK cascade;cytoplasm;regulation of Ras protein signal transduction |
| 5909 | Ksr1 complex (Ksr1, Mek, 14-3-3, Mapk), EGF stimulated | P31938;P63085;P63101;Q61097 | 26395;26413;22631;16706 | protein phosphorylation;MAPK cascade;plasma membrane;regulation of Ras protein signal transduction |
| 6049 | Ncstn-Psen1 complex | P49769;P57716 | None;None | protein processing;peptidase activator activity;signaling;plasma membrane;Golgi apparatus |
| 6222 | Ccm2-Krit1 complex | Q6S5J6;Q8K2Y9 | 79264;216527 | angiogenesis;regulation of cell migration involved in sprouting angiogenesis;regulation of blood vessel endothelial cell proliferation involved in sprouting angiogenesis |
| 6252 | Ap1g1-Ap1s1-Rabep1 complex | O35551;P22892;P61967 | 54189;11765;11769 | recycling endosome membrane |
| 6397 | Dnajc5-Sgta complex | P60904;P63017;Q8BJU0 | 13002;15481;52551 | membrane;trans-Golgi network |
| 6407 | Vangl1-Vangl2 complex | Q80Z96;Q91ZD4 | 229658;93840 | cochlea development |
| 6475 | Dnm1l-Mff complex | Q6PCP5;Q8K1M6 | 75734;74006 | mitochondrial fission |
| 6478 | Dnm1l-Mief1 complex | Q8BGV8;Q8K1M6 | 239555;74006 | mitochondrial fission |
| 6508 | L1cam-Ncam1 complex | P11627;P13595 | None;17967 | cell adhesion;neuron cell-cell adhesion |
| 6511 | L1cam-Itga5 complex | P11627;P11688 | None;16402 | cell-cell adhesion mediator activity;cell aggregation |
| 6539 | Plp1-Zfyve27 complex | P60202;Q3TXX3 | 18823;319740 | endoplasmic reticulum;endoplasmic reticulum tubular network formation;endoplasmic reticulum tubular network maintenance;vesicle-mediated transport |
| 6541 | Rtn1-Zfyve27 complex | Q3TXX3;Q8K0T0 | 319740;104001 | endoplasmic reticulum;endoplasmic reticulum tubular network formation;endoplasmic reticulum tubular network maintenance;vesicle-mediated transport |
| 6542 | Rtn3-Zfyve27 complex | Q3TXX3;Q9ES97 | 319740;20168 | endoplasmic reticulum;endoplasmic reticulum tubular network formation;endoplasmic reticulum tubular network maintenance;vesicle-mediated transport |
| 6543 | Rtn4-Zfyve27 complex | Q3TXX3;Q99P72 | 319740;68585 | endoplasmic reticulum;endoplasmic reticulum tubular network formation;endoplasmic reticulum tubular network maintenance;vesicle-mediated transport |
| 6544 | Kif5a-Zfyve27 complex | P33175;Q3TXX3 | 16572;319740 | endoplasmic reticulum;endoplasmic reticulum tubular network formation;endoplasmic reticulum tubular network maintenance;vesicle-mediated transport |
| 6547 | Kif5b-Zfyve27 complex | Q3TXX3;Q61768 | 319740;16573 | endoplasmic reticulum;endoplasmic reticulum tubular network formation;endoplasmic reticulum tubular network maintenance;vesicle-mediated transport |
| 6548 | Kif5c-Zfyve27 complex | P28738;Q3TXX3 | 16574;319740 | endoplasmic reticulum;endoplasmic reticulum tubular network formation;endoplasmic reticulum tubular network maintenance;vesicle-mediated transport |
| 6633 | Rbx1- Cul1-Skp1-Fbxw8-Cul7-Rbx1 | P62878;Q8BIA4;Q8VE73;Q9WTX5;Q9WTX6 | 56438;231672;66515;21402;26965 | placenta development;protein ubiquitination |
| 6672 | Ank2-Kcnj11-Abcc8 complex | Q61743;Q6PGE2;Q8C8R3 | 16514;20927;109676 | potassium ion transmembrane transport |
| 6687 | Arl6-Sec61b complex | O88848;Q9CQS8 | 56297;66212 | cytosol |
| 6759 | Vangl1-Dvl1 complex | P51141;Q80Z96 | 13542;229658 | plasma membrane |
| 6761 | Vangl1-Dvl3 complex | Q61062;Q80Z96 | 13544;229658 | plasma membrane |
| 6762 | Vangl2-Dvl1 complex | P51141;Q91ZD4 | 13542;93840 | plasma membrane |
| 6764 | Vangl2-Dvl3 complex | Q61062;Q91ZD4 | 13544;93840 | plasma membrane |
| 6774 | Fhl1-Pdlim1-Gsn-Actn1 complex | O70400;P13020;P97447;Q7TPR4 | 54132;227753;14199;109711 | None |
| 6812 | Arrb1-Becn1-Pik3c3 complex, induced by (OGD) oxygen-glucose deprivation | O88597;Q6PF93;Q8BWG8 | 56208;225326;109689 | regulation of autophagy;autophagosome assembly |
| 6867 | Amfr-Vcp-Ngly1 complex | Q01853;Q9JI78;Q9R049 | 269523;59007;23802 | ERAD pathway |
| 6868 | Amfr-Vcp-Ubxn1 complex | Q01853;Q922Y1;Q9R049 | 269523;225896;23802 | ERAD pathway |
| 6869 | Ngly1-Vcp-Ubxn1 complex | Q01853;Q922Y1;Q9JI78 | 269523;225896;59007 | ERAD pathway |
| 6870 | Amfr-Vcp-Ubxn1-Ngly1-Rad23b complex | P54728;Q01853;Q922Y1;Q9JI78;Q9R049 | 19359;269523;225896;59007;23802 | ERAD pathway |
| 6877 | Hsp90b1-Cnpy3 complex | P08113;Q9DAU1 | 22027;72029 | chaperone cofactor-dependent protein refolding;endoplasmic reticulum |
| 6911 | Frs2-Grb2-Sos1 complex | Q60631;Q62245;Q8C180 | 14784;20662;327826 | plasma membrane;neuron projection development;positive regulation of DNA biosynthetic process;positive regulation of MAPK cascade |
| 6912 | Cbl-Frs2-Grb2 complex | P22682;Q60631;Q8C180 | 12402;14784;327826 | ubiquitin-dependent protein catabolic process |
| 7237 | Dync1i1-Snca complex | O55042;O88485 | 20617;None | None |
| 7244 | Apba1-Cask-Nrxn1-Ppfia1 complex | B2RUJ5;B8QI33;O70589;P0DI97 | 319924;None;12361;18189 | regulation of protein serine/threonine kinase activity |
| 7257 | Abl1-Abl2-Crk-Unc119 complex | P00520;Q4JIM5;Q64010;Q9Z2R6 | 11350;None;12928;22248 | negative regulation of protein kinase activity by protein phosphorylation |
| 7280 | Bin1-Dnm1-Ehbp1 complex | O08539;P39053;Q69ZW3 | 30948;13429;216565 | None |
| 7315 | Ap1g1-Ap2a2-Dcx complex | O88809;P17427;P22892 | 13193;11772;11765 | neuron migration |
| 7484 | Abca1-Apoa1-Snta1 complex | P41233;Q00623;Q61234 | 11303;11806;20648 | None |
| 7492 | Bcl2-Becn1 complex | O88597;P10417 | 56208;12043 | None |
| 7660 | Itch-Ndfip1-Ube2l3 complex | P68037;Q8C863;Q8R0W6 | 22195;16396;65113 | inflammatory response |
| 7778 | Akt1-Ppp2ca complex | P31750;P63330 | 11651;19052 | None |
| 7804 | Gnaz-Rgs17 complex | O70443;Q9QZB0 | 14687;56533 | G protein-coupled receptor signaling pathway;plasma membrane |
| 7810 | Micall2-Rab8a complex | P55258;Q3TN34 | 17274;231830 | cell-cell junction;cell-cell junction assembly |
| 7811 | Micall2-Rab13 complex | Q3TN34;Q9DD03 | 231830;68328 | cell-cell junction;cell-cell junction assembly |
| 7824 | Mob4-Ppp2ca-Ppp2r1a-Strn3 complex | P63330;Q6PEB6;Q76MZ3;Q9ERG2 | 19052;19070;51792;94186 | None |
| 7828 | Mpc1-Mpc2 complex | P63030;Q9D023 | 55951;70456 | mitochondrial inner membrane;pyruvate transport |
| 7929 | Nono-Pspc1-Sfpq complex | Q8R326;Q8VIJ6;Q99K48 | 66645;71514;53610 | regulation of DNA-templated transcription;nucleus |
| 7970 | Elavl1-Khsrp complex | P70372;Q3U0V1 | 15568;16549 | skeletal muscle fiber development;mRNA destabilization |
| 8204 | Megf8-Mgrn1 complex | P60882;Q9D074 | 269878;17237 | protein ubiquitination;smoothened signaling pathway;heart development |
| 8304 | Ppp6c-Ppp6r1-Ppp6r3 complex | Q7TSI3;Q922D4;Q9CQR6 | 243819;52036;67857 | regulation of phosphoprotein phosphatase activity |
| 8361 | Pura-Purb complex | O35295;P42669 | 19291;19290 | nucleus;negative regulation of DNA-templated transcription |
| 8362 | Pura-Purb-Ybx1 complex | O35295;P42669;P62960 | 19291;19290;22608 | nucleus |
| 8413 | Cacna1c-Gja1 complex | P23242;Q01815 | 14609;12288 | regulation of calcium ion transmembrane transporter activity |
| 8451 | Maged1-Ror2 complex | Q9QYH6;Q9Z138 | 94275;None | regulation of DNA-templated transcription;membrane |
| 8478 | Drg1-Zc3h15 complex | P32233;Q3TIV5 | 13494;69082 | gene expression |
| 8479 | Drg2-Rwdd1 complex | Q9CQK7;Q9QXB9 | 66521;13495 | None |
| 8482 | Rsph3b-Rsph4a complex | Q8BYM7;Q9DA80 | 212892;100037282 | cilium movement;motile cilium |
| 8485 | Rsph4a-Rsph9 complex | Q8BYM7;Q9D9V4 | 212892;75564 | cilium movement;motile cilium |
| 8496 | Gpr37l1-Ptch1 complex | Q61115;Q99JG2 | 19206;171469 | cerebellum development;smoothened signaling pathway |
| 8531 | Adcy6-Akap5-Pde4c-Pkd2-Prkar2a complex | D3YVF0;O35245;P12367;Q01341;Q3UEI1 | 238276;18764;19087;11512;110385 | cilium;regulation of adenylate cyclase activity;cAMP metabolic process;cAMP-mediated signaling |
| 8534 | Adcy5-Akap5-Pde4c-Pkd2-Prkar2a complex | D3YVF0;O35245;P12367;P84309;Q3UEI1 | 238276;18764;19087;224129;110385 | cilium;regulation of adenylate cyclase activity;cAMP metabolic process;cAMP-mediated signaling |
| 8646 | Ptpn6-Vav1 complex | P27870;P29351 | 22324;15170 | hematopoietic stem cell differentiation;positive regulation of Ras protein signal transduction |
| 8797 | Ache-Prima1 complex | P21836-1;Q810F0 | None;170952; | acetylcholinesterase activity;plasma membrane;protein tetramerization |
| 8798 | Bche-Prima1 complex | Q03311;Q810F0 | 12038;;170952; | cholinesterase activity;plasma membrane;cytokine receptor binding |
| 8803 | Ache homotetramer | P21836-1 | None | acetylcholinesterase activity;protein tetramerization |
| 40 | COP9 signalosome complex | O35864;O88543;O88544;O88545;P61202;Q8VBV7;Q99LD4;Q9CZ04 | 26754;26572;26891;26893;12848;108679;209318;26894 | COP9 signalosome |
| 146 | Src-dynamin-synapsin complex | O88935;P05480;P39053 | 20964;20779;13429 | synaptic vesicle exocytosis |
| 317 | Dystrobrevin-syntrophin complex, brain-derived | O70585;P11531;Q61234;Q9D2N4 | 13528;13405;20648;13527 | chemical synaptic transmission;plasma membrane |
| 326 | Smooth muscle sarcoglycan complex SGC, beta-delta-zeta | P82347;P82349;Q8BX51 | 24052;24051;244431 | smooth muscle contraction;smooth muscle tissue development;plasma membrane |
| 337 | Dystrophin-sarcoglycan-syntrophin complex, skeletal muscle | P11531;P82347;P82348;P82349;P82350;Q61234 | 13405;24052;24053;24051;20391;20648 | striated muscle contraction;skeletal muscle tissue development;striated muscle tissue development;plasma membrane |
| 416 | Nitric oxide synthase-dystrophin complex, skeletal muscle | P11531;Q9Z0J4 | 13405;18125 | nitric oxide mediated signal transduction;muscle contraction;plasma membrane |
| 427 | Tripeptidyl peptidase | Q64514 | 22019 | cytoplasm;protein catabolic process |
| 495 | Cytochrome bc1-complex, mitochondrial | P00158;P99028;Q8R1I1;Q9CPX8;Q9CQ69;Q9CR68;Q9CZ13;Q9D0M3;Q9D855;Q9DB77 | 17711;66576;66152;66594;22272;66694;22273;66445;67530;67003 | aerobic respiration;generation of precursor metabolites and energy;mitochondrial inner membrane;respiratory electron transport chain;tricarboxylic acid cycle;proton transmembrane transport |
| 735 | SNARE complex (Stx6, Stx7, Vti1b) | O70439;O88384;Q9JKK1 | 53331;53612;58244 | membrane fusion;SNARE complex;Golgi membrane;exocytosis;vesicle fusion;synaptic vesicle transport |
| 736 | SNARE complex (Stx7, Stx8, Vamp8, Vti1b) | O70404;O70439;O88384;O88983 | 22320;53331;53612;55943 | vesicle fusion;synaptic vesicle transport;membrane fusion;exocytosis;SNARE complex |
| 795 | SNARE complex (Cplx1, Snap25, Stx1a, Vamp2) | O35526;P60879;P63040;P63044 | 20907;20614;12889;22318 | signaling receptor regulator activity;vesicle fusion;synaptic vesicle transport;synaptic vesicle exocytosis;signaling receptor binding;membrane fusion;SNARE complex |
| 796 | SNARE complex (Stx1a, Snap25, Cplx2, Vamp2) | O35526;P60879;P63044;P84086 | 20907;20614;22318;12890 | signaling receptor regulator activity;vesicle fusion;synaptic vesicle transport;synaptic vesicle exocytosis;signaling receptor binding;SNARE complex;membrane fusion |
| 859 | SNARE complex (Stx6, Stx7, Vamp7, Vti1b) | O70439;O88384;P70280;Q9JKK1 | 53331;53612;20955;58244 | membrane fusion;late endosome;SNARE complex |
| 935 | Scrib-APC-beta-catenin complex | Q02248;Q61315;Q80U72 | 12387;11789;105782 | anisotropic cell growth;axis specification;cell-cell junction assembly;neuron differentiation;epithelial cell differentiation;morphogenesis of an epithelium;synapse assembly |
| 970 | Rab11Fip2-Ap2a complex | P17426;Q3U366 | 11771;74998 | receptor-mediated endocytosis |
| 1135 | Mss4-Itga3 complex | Q62470;Q91X96 | 16400;98710 | extracellular matrix organization;regulation of metallopeptidase activity |
| 1136 | Mss4-Itga7 complex | Q61738;Q91X96 | 16404;98710 | extracellular matrix organization;regulation of metallopeptidase activity |
| 1212 | SNARE complex (Stx4, Vamp8, Snap23) | O09044;O70404;P70452 | 20619;22320;20909 | vesicle fusion;synaptic vesicle transport;synaptic vesicle exocytosis;SNARE complex;membrane fusion |
| 1213 | SNARE complex (Stx4, Vamp2, Snap23) | O09044;P63044;P70452 | 20619;22318;20909 | vesicle fusion;synaptic vesicle transport;synaptic vesicle exocytosis;SNARE complex;membrane fusion |
| 1423 | L-periaxin-Drp2 complex | E9Q6R7;O55103;P11531;Q05AA6;Q62165 | 22288;19153;13405;13497;13138 | plasma membrane |
| 1595 | Cdk5-c-Abl-Cables complex | P00520;P49615;Q9ESJ1 | 11350;12568;63955 | protein phosphorylation;nervous system development;neuron projection development |
| 1778 | TGF-beta-receptor-Strap complex | Q62312;Q64729;Q9Z1Z2 | 21813;21812;20901 | transforming growth factor beta receptor signaling pathway;cytoplasm |
| 1782 | TGF-beta receptor | Q62312;Q64729 | 21813;21812 | protein binding;transforming growth factor beta receptor signaling pathway;plasma membrane |
| 1913 | GluR delta-2 complex, postsynaptic | D3YZU1;P97772;Q61625;Q62108;Q80Z38;Q9Z2Y3 | 243961;14816;14804;13385;210274;26556 | ion channel activity;chemical synaptic transmission;synapse assembly |
| 2835 | Profilin 2 complex | O88935;P28660;P39053;P60710;P63017;P70336;Q64332;Q7TMB8;Q9JJV2 | 20964;50884;13429;11461;15481;19878;20965;20430;18645 | endocytosis;actin cytoskeleton organization;protein-containing complex assembly |
| 2836 | Profilin 1 complex | P62962;P63017;P63260;Q01853;Q68FD5;Q9CWF2 | 18643;15481;11465;269523;67300;73710 | endocytosis;actin cytoskeleton organization;protein-containing complex assembly |
| 3007 | TGF-beta-receptor type I homodimer complex | Q64729 | 21812 | protein binding;transforming growth factor beta receptor signaling pathway;endoplasmic reticulum |
| 3037 | Ubiquitin E3 ligase (Fbxl20, Skp1, Cul1) | Q9CZV8;Q9WTX5;Q9WTX6 | 72194;21402;26965 | protein ubiquitination;plasma membrane;ubiquitin-dependent protein catabolic process;chemical synaptic transmission |
| 3094 | Metaxin complex (Mtx1, Mtx2), mitochondrial | O88441;P47802 | 53375;17827 | protein transport;mitochondrial outer membrane |
| 3192 | G protein complex (Hdac4, Gnb1, Gng2) | P62874;P63213;Q6NZM9 | 14688;14702;208727 | negative regulation of DNA-templated transcription;G protein-coupled receptor signaling pathway |
| 3193 | G protein complex (Gnb1, Gng2, (Hdac5) | P62874;P63213;Q9Z2V6 | 14688;14702;15184 | negative regulation of DNA-templated transcription;G protein-coupled receptor signaling pathway |
| 5211 | RAF1 complex (Pin1, Raf1, Ppp2cb, Ppp2r1a, Ppp2r2a) | P62715;Q6P1F6;Q76MZ3;Q99N57;Q9QUR7 | 19053;71978;51792;110157;23988 | transmembrane receptor protein serine/threonine kinase signaling pathway;protein phosphorylation |
| 5271 | Kif3-cadherin-catenin complex | P15116;P30999;P70188;Q02248;Q61771 | 12558;12388;16579;12387;16569 | brain development |
| 5791 | PlexinA1-PlexinB1 complex | P70206;Q8CJH3 | 18844;235611 | transmembrane receptor protein tyrosine kinase signaling pathway;angiogenesis |
| 5879 | Ksr1-PP2A holoenzyme complex (Ppp2r1a, Ppp2r2b, Ppp2ca), PDGF stimulated | P63330;Q61097;Q6ZWR4;Q76MZ3 | 19052;16706;72930;51792 | protein dephosphorylation;MAPK cascade;plasma membrane;regulation of Ras protein signal transduction |
| 5880 | Ksr1-PP2A core enzyme complex (Ppp2r1a, Ppp2ca), untreated | P63330;Q61097;Q76MZ3 | 19052;16706;51792 | protein dephosphorylation;MAPK cascade |
| 5881 | Ksr1-PP2A core enzyme complex (Ppp2r1a, Ppp2ca), untreated | P63330;Q61097;Q76MZ3 | 19052;16706;51792 | protein dephosphorylation;MAPK cascade;cytoplasm |
| 5882 | Raf1-PP2A holoenzyme complex (Ppp2r1a, Ppp2r2b, Ppp2ca), PDGF stimulated | P63330;Q6ZWR4;Q76MZ3;Q99N57 | 19052;72930;51792;110157 | protein dephosphorylation;MAPK cascade;plasma membrane |
| 5883 | Raf1-PP2A core enzyme complex (Ppp2r1a, Ppp2ca), untreated | P63330;Q76MZ3;Q99N57 | 19052;51792;110157 | protein dephosphorylation;MAPK cascade;cytoplasm |
| 6048 | Dynein-dynactin complex | O08788;O88485;Q99KJ8 | None;None;None | cytoskeleton-dependent intracellular transport;dynactin binding |
| 6105 | CCT-gelsolin complex | P11983;P13020 | 21454;227753 | cell motility |
| 6201 | Dock3-Elmo-RhoG complex | P84096;Q8BPU7;Q8CIQ7 | 56212;140580;56212 | regulation of brain-derived neurotrophic factor receptor signaling pathway;regulation of neurotrophin TRK receptor signaling pathway |
| 6326 | Dystrophin complex (Dmd; Snta1) | P11531;Q61234 | 13405;20648 | None |
| 6327 | Dystrophin complex (Dmd; Stnb1) | P11531;Q99L88 | 13405;20649 | None |
| 6328 | Utrophin complex | E9Q6R7;Q99L88 | 22288;20649 | None |
| 6593 | Kinesin II complex (Kif3a-Kif3b-Kifap3) | P28741;P70188;Q61771 | 16568;16579;16569 | intraciliary anterograde transport;cilium;intraciliary transport;cilium assembly |
| 6809 | Arrb2-Akt-Ppp2ca complex | P31750;P63330;Q91YI4 | 11651;19052;216869 | protein kinase B signaling;G protein-coupled receptor signaling pathway |
| 6810 | Akt-Arrb2-Ppp2ca-Ppp2r2a complex | P31750;P63330;Q6P1F6;Q91YI4 | 11651;19052;71978;216869 | protein kinase B signaling;G protein-coupled receptor signaling pathway |
| 6813 | Arrb2-Akt-Src complex, insulin induced | P05480;P31750;Q91YI4 | 20779;11651;216869 | protein kinase B signaling;response to insulin |
| 6879 | Dystrophin-glycoprotein complex (DGC) | P46938;P82347;Q62165;Q7TSJ6 | 22601;24052;13138;50523 | plasma membrane;regulation of hippo signaling |
| 6882 | Periaxin complex | A2AMT1;E9Q616;O55103;P20152;P26040;Q68FG2;Q6NVD9;Q810U4;Q8C8R3;Q9QXS1 | 12075;66395;19153;22352;22350;20743;107993;319504;109676;18810 | membrane organization;lens fiber cell morphogenesis;cytoskeleton organization;plasma membrane;cytoplasm |
| 6965 | Glutamate receptor complex (GRIN1, GRIN2A, GRIN2B), ionotropic, NMDA-type | P35436;P35438;Q01097 | 14811;14810;14812 | extracellularly glutamate-gated ion channel activity;NMDA glutamate receptor activity;regulation of synaptic plasticity;glutamate-gated calcium ion channel activity |
| 6966 | Glutamate receptor (Grin1, Grin2A) - Grin3B complex, ionotropic, NMDA-type | P35436;P35438;Q91ZU9 | 14811;14810;170483 | cellular calcium ion homeostasis;extracellularly glutamate-gated ion channel activity;NMDA glutamate receptor activity;regulation of synaptic plasticity |
| 7306 | Arhgef9(splice variant CB1)-Gphn-Nlgn2 complex | Q69ZK9;Q811P8-1;Q8BUV3 | 216856;330914;268566 | regulation of dendrite development |
| 7307 | Arhgef9(splice variant CB2)-Gphn-Nlgn2 complex | Q69ZK9;Q811P8-2;Q8BUV3 | 216856;330914;268566 | regulation of dendrite development |
| 8036 | Fhl1-Titin complex | A2ASS6;P97447 | None;14199 | response to stress |
| 8077 | Ahcyl1-Slc9a3-Slc9a3r1 complex | G3X939;P70441;Q80SW1 | 105243;26941;229709 | None |
| 8139 | BACE-APP complex | P12023;P56818 | 11820;23821 | amyloid precursor protein catabolic process |
| 8392 | SNARE complex (Stx6, Stx7, Vamp8, Vti1b) | O70404;O70439;O88384;Q9JKK1 | 22320;53331;53612;58244 | late endosome;membrane fusion;SNARE complex |
| 8511 | Prom1-radixin complex | O54990;P26043 | 19126;19684 | gluconeogenesis;protein kinase A signaling;cilium assembly |
| 8653 | Golga7b-Zdhhc5 complex | Q8VDZ4;Q9D428 | 228136;71146 | cell adhesion;desmosome assembly;palmitoyltransferase activity;protein palmitoylation;plasma membrane |
| 8804 | ACHE homodimer | P21836-1 | None | acetylcholinesterase activity;protein homodimerization activity |
