# Supplementary material for: The ultrastructural and proteomic analysis of mitochondria‐associated endoplasmic reticulum membrane in the midbrain of a Parkinson's disease mouse model
Source: Aging Cell. 2024 Nov 29;24(4):e14436. doi: 10.1111/acel.14436 (PMC11984660; doi:10.1111/acel.14436)
Supplement: Supplementary file 14 — Table S8. List of DEPs in MAM proteomics. [file ACEL-24-e14436-s010.docx]

**Supplementary Table** **8 List of DEPs in MAM proteomics**

| Up-regulated proteins | Down-regulated proteins |
| --- | --- |
| Ndst2 | Ggcx |
| Ehd2 | Tyw5 |
| Efna3 | Nat8f1 |
| Nploc4 | Fbxw11 |
| Gna14 | Slc41a1 |
| Ddr1 | Dph1 |
| Ptn | Mgat1 |
| Fmn2 | Car11 |
| M6pr | Camsap2 |
| Atraid | Bag3 |
| Arhgef6 | Il1rapl1 |
| Reck | Mmp15 |
| Leprot | Gan |
| Myo1f | Ext2 |
| Tnfrsf1b | Nf2 |
| Krt14 | Katnb1 |
| Cdip1 | Ntn1 |
| Lamc1 | Plekhh1 |
| Gpr137 | Slc10a7 |
| Tmc7 | Elovl5 |
| Unc13a | Naglu |
| Tmem151b | Sh3pxd2b |
| Npepps | Snx11 |
| Sugp2 | Afdn |
| Sars2 | Spag7 |
| Eml5 | Ptprk |
| Tcaf1 | Tom1l1 |
| Cldn11 | Palmd |
| Pak3 | Sec61g |
| Fyb1 | Ssh1 |
| Dnajc4 | Nt5m |
| Trim12c | Opcml |
| Pten | Tpm1 |
| Ighg2c | Erbin |
| Antxr1 | Sbf2 |
| Chkb | Bbs9 |
| Lrrk2 | Aasdhppt |
| Cfap77 | Ophn1 |
| Cep97 | Copz1 |
| Picalm | Slc4a4 |
| Map7d1 | Arhgef10l |
| Nrd1 | Prickle2 |
| Eif2b4 | Rasal2 |
| Gsdma2 | Alg14 |
| Ctdsp1 | Tmem248 |
| Ly6e | Lamb1 |
| Cfap45 | Rps6kb1 |
| Atp2b4 | Gpr84 |
| Carmil1 | Puf60 |
| Krt6a | Tsta3 |
| Vti1a | Derl2 |
| Dmd | Lmbr1 |
| Cd300e | Cul9 |
| Tap1 | Anks1a |
| Aqp1 | Ngly1 |
| Shc3 |  |
| Tomm5 |  |
| Rps27a |  |
| Mt3 |  |
| Mt1 |  |
| Rcsd1 |  |
| Tmprss5 |  |
| Aff3 |  |
| Krt5 |  |
| Mdm1 |  |
| 5031439G07Rik |  |
| Slc12a7 |  |
| Mfsd14b |  |
| Gm19935 |  |
| Clasp2 |  |
| Rhag |  |
| Dynlt3 |  |
| Hmbs |  |
| Ccdc51 |  |
| Slc1a2 |  |
| Leprotl1 |  |
| Sft2d2 |  |
| Cnbp |  |
| H2afz |  |
| Cep350 |  |
| Jak1 |  |
| Gpr17 |  |
| Rps29 |  |
| Pcdhgb6 |  |
| Znf706 |  |
| Hyal2 |  |
| Pgm2 |  |
| Tmem173 |  |
| Pde10a |  |
| Atp1a4 |  |
| Rnasek |  |
| Angel2 |  |
| Naa30 |  |
| Map4 |  |
| Gemin5 |  |
| Grin2a |  |
| Gabrg3 |  |
| Exoc1 |  |
| Unc13c |  |
| Syp |  |
| Sclt1 |  |
| Dpp4 |  |
| Nyap2 |  |
