# Supplementary material for: The ultrastructural and proteomic analysis of mitochondria‐associated endoplasmic reticulum membrane in the midbrain of a Parkinson's disease mouse model
Source: Aging Cell. 2024 Nov 29;24(4):e14436. doi: 10.1111/acel.14436 (PMC11984660; doi:10.1111/acel.14436)
Supplement: Supplementary file 15 — Table S9. Information of protein subcellular localizations in MAM proteomics. [file ACEL-24-e14436-s014.docx]

**Supplementary Table 9** **Information of protein subcellular localizations in MAM proteomics**

| Subcellular components | Exclusive (Uniprot) | Exclusive (HPA) | Combined (Uniprot) | Combined (HPA) |
| --- | --- | --- | --- | --- |
| Mitochondria (n, %) | 514 (18.067) | 388 (16.768) | 1020 (6.996) | 694 (7.133) |
| ER (n, %) | 272 (9.561) | 151 (6.525) | 871 (5.974) | 334 (3.433) |
| Nucleus (n, %) | 201 (7.065) | 461 (19.922) | 1653 (11.337) | 2826 (29.044) |
| Cytosol (n, %) | 59 (2.074) | 516 (22.299) | 354 (2.428) | 2322 (23.864) |
| Golgi apparatus (n, %) | 135 (4.745) | 136 (5.877) | 673 (4.616) | 562 (5.776) |
| Lysosomes, Endosomes, Peroxisomes (n, %) | 132 (4.640) | 18 (0.778) | 1020 (6.996) | 54 (0.555) |
| Cytoskeleton (n, %) | 97 (3.409) | 69 (2.982) | 914 (6.269) | 375 (3.854) |
| Plasma membrane (n, %) | 354 (12.443) | 167 (7.217) | 1785 (12.243) | 987 (10.144) |
| Others (n, %) | 1081 (37.996) | 408 (17.632) | 6290 (43.141) | 1576 (16.197) |
| Total number of locations | 2845 | 2314 | 14580 | 9730 |
| Total number of involved proteins | 2845 | 2314 | 6380 | 2866 |
| HPA, human protein atlas; ER, endoplasmic reticulum. | | | | |
