# Supplementary material for: The ultrastructural and proteomic analysis of mitochondria‐associated endoplasmic reticulum membrane in the midbrain of a Parkinson's disease mouse model
Source: Aging Cell. 2024 Nov 29;24(4):e14436. doi: 10.1111/acel.14436 (PMC11984660; doi:10.1111/acel.14436)
Supplement: Supplementary file 16 — Table S10. Results of GO and KEGG analysis for DEPs in MAM proteomics. [file ACEL-24-e14436-s009.docx]

### Supplementary Table 10 Results of GO and KEGG analysis for DEPs in MAM proteomics

| Term | Count | Percentage | Fold Enrichment | p value | Genes |
| --- | --- | --- | --- | --- | --- |
| BP Terms |  | | | | |
| GO:0031175~neuron projection development | 8 | 5.263 | 6.220 | 0.000 | OPHN1, PTEN, PRICKLE2, LAMB1, LAMC1, PTPRK, MAP4, CAMSAP2 |
| GO:0051496~positive regulation of stress fiber assembly | 5 | 3.289 | 12.132 | 0.001 | CARMIL1, TPM1, SH3PXD2B, ARHGEF10L, NF2 |
| GO:0030336~negative regulation of cell migration | 7 | 4.605 | 6.397 | 0.001 | AFDN, TCAF1, TPM1, PTEN, PTPRK, PTN, RECK |
| GO:0042391~regulation of membrane potential | 6 | 3.947 | 7.217 | 0.001 | GRIN2A, LRRK2, ATP1A4, DMD, SLC4A4, GABRG3 |
| GO:0030210~heparin biosynthetic process | 3 | 1.974 | 42.220 | 0.002 | NDST2, EXT2, SLC10A7 |
| GO:0016188~synaptic vesicle maturation | 3 | 1.974 | 42.220 | 0.002 | UNC13C, UNC13A, PICALM |
| GO:0007611~learning or memory | 5 | 3.289 | 8.687 | 0.003 | GRIN2A, SHC3, PTEN, LAMB1, PICALM |
| GO:2000009~negative regulation of protein localization to cell surface | 3 | 1.974 | 30.157 | 0.004 | LEPROT, LEPROTL1, PICALM |
| GO:0071294~cellular response to zinc ion | 3 | 1.974 | 23.455 | 0.007 | GRIN2A, MT1, MT3 |
| GO:1900242~regulation of synaptic vesicle endocytosis | 3 | 1.974 | 17.592 | 0.012 | OPHN1, LRRK2, PICALM |
| GO:0071280~cellular response to copper ion | 3 | 1.974 | 17.592 | 0.012 | MT1, MT3, AQP1 |
| GO:0007268~chemical synaptic transmission | 6 | 3.947 | 4.330 | 0.012 | UNC13C, GRIN2A, UNC13A, SYP, SLC12A7, GABRG3 |
| GO:0030036~actin cytoskeleton organization | 6 | 3.947 | 4.265 | 0.013 | EHD2, OPHN1, ARHGEF10L, DMD, NF2, SSH1 |
| GO:0071456~cellular response to hypoxia | 5 | 3.289 | 5.371 | 0.014 | NPEPPS, PTEN, FMN2, PTN, AQP1 |
| GO:1902803~regulation of synaptic vesicle transport | 2 | 1.316 | 140.732 | 0.014 | LRRK2, PICALM |
| GO:0015031~protein transport | 11 | 7.237 | 2.415 | 0.016 | TOM1L1, SEC61G, VTI1A, BBS9, TAP1, FMN2, SNX11, SFT2D2, TOMM5, COPZ1, EXOC1 |
| GO:0048854~brain morphogenesis | 3 | 1.974 | 15.078 | 0.017 | AFDN, FBXW11, PTEN |
| GO:1903861~positive regulation of dendrite extension | 3 | 1.974 | 14.559 | 0.018 | AFDN, UNC13A, PICALM |
| GO:0009749~response to glucose | 4 | 2.632 | 6.782 | 0.021 | EIF2B4, RPS6KB1, PTEN, RASAL2 |
| GO:0006470~protein dephosphorylation | 5 | 3.289 | 4.660 | 0.022 | FBXW11, CTDSP1, PTEN, PTPRK, SSH1 |
| GO:0060400~negative regulation of growth hormone receptor signaling pathway | 2 | 1.316 | 93.822 | 0.021 | LEPROT, LEPROTL1 |
| GO:0000226~microtubule cytoskeleton organization | 5 | 3.289 | 4.629 | 0.023 | CUL9, MAP7D1, MAP4, CLASP2, CAMSAP2 |
| GO:0007026~negative regulation of microtubule depolymerization | 3 | 1.974 | 11.728 | 0.027 | KATNB1, CLASP2, CAMSAP2 |
| GO:0042493~response to drug | 7 | 4.605 | 3.050 | 0.027 | GRIN2A, RPS6KB1, HMBS, PTEN, SLC1A2, PTN, GABRG3 |
| GO:0006811~ion transport | 10 | 6.579 | 2.334 | 0.027 | GRIN2A, SLC10A7, ATP1A4, ATP2B4, SLC41A1, TMC7, SLC12A7, SLC4A4, GABRG3, CCDC51 |
| GO:1903690~negative regulation of wound healing, spreading of epidermal cells | 2 | 1.316 | 70.366 | 0.028 | PTEN, CLASP2 |
| GO:0061789~dense core granule priming | 2 | 1.316 | 70.366 | 0.028 | UNC13C, UNC13A |
| GO:2000463~positive regulation of excitatory postsynaptic potential | 3 | 1.974 | 11.110 | 0.029 | GRIN2A, PTEN, SSH1 |
| GO:0009888~tissue development | 3 | 1.974 | 10.297 | 0.034 | LAMB1, LAMC1, NTN1 |
| GO:0006875~cellular metal ion homeostasis | 2 | 1.316 | 56.293 | 0.035 | MT1, MT3 |
| GO:0034614~cellular response to reactive oxygen species | 3 | 1.974 | 10.052 | 0.035 | TPM1, PTPRK, MT3 |
| GO:0007528~neuromuscular junction development | 3 | 1.974 | 10.052 | 0.035 | UNC13C, UNC13A, LRRK2 |
| GO:0010975~regulation of neuron projection development | 3 | 1.974 | 9.595 | 0.039 | IL1RAPL1, PTEN, PAK3 |
| GO:0010273~detoxification of copper ion | 2 | 1.316 | 46.911 | 0.042 | MT1, MT3 |
| GO:1902083~negative regulation of peptidyl-cysteine S-nitrosylation | 2 | 1.316 | 46.911 | 0.042 | ATP2B4, DMD |
| GO:1903215~negative regulation of protein targeting to mitochondrion | 2 | 1.316 | 46.911 | 0.042 | BAG3, LRRK2 |
| GO:0008152~metabolic process | 5 | 3.289 | 3.783 | 0.043 | NDST2, PDE10A, NAGLU, HYAL2, PAK3 |
| GO:0035249~synaptic transmission, glutamatergic | 3 | 1.974 | 8.983 | 0.043 | UNC13C, SHC3, UNC13A |
| GO:0032880~regulation of protein localization | 4 | 2.632 | 4.982 | 0.046 | TRIM12C, AFDN, KRT5, PICALM |
| GO:0042177~negative regulation of protein catabolic process | 3 | 1.974 | 8.616 | 0.047 | GRIN2A, FMN2, ATRAID |
| GO:0007568~aging | 5 | 3.289 | 3.646 | 0.048 | RPS6KB1, KRT14, PTEN, DMD, TNFRSF1B |
| GO:0015696~ammonium transport | 2 | 1.316 | 40.209 | 0.048 | RHAG, AQP1 |
| GO:0015014~heparan sulfate proteoglycan biosynthetic process, polysaccharide chain biosynthetic process | 2 | 1.316 | 40.209 | 0.048 | NDST2, EXT2 |
| GO:0071474~cellular hyperosmotic response | 2 | 1.316 | 40.209 | 0.048 | RCSD1, AQP1 |
| MF Terms |  | | | | |
| GO:0017075~syntaxin-1 binding | 4 | 2.632 | 20.347 | 0.001 | UNC13C, UNC13A, LRRK2, SYP |
| GO:0000149~SNARE binding | 5 | 3.289 | 8.918 | 0.002 | UNC13A, LRRK2, VTI1A, SYP, PICALM |
| GO:0008519~ammonium transmembrane transporter activity | 3 | 1.974 | 37.457 | 0.003 | RHAG, SLC12A7, AQP1 |
| GO:0008017~microtubule binding | 8 | 5.263 | 4.146 | 0.003 | EML5, CEP350, KATNB1, FMN2, MAP4, CLASP2, MDM1, CAMSAP2 |
| GO:0030276~clathrin binding | 4 | 2.632 | 11.211 | 0.005 | TOM1L1, LRRK2, SCLT1, PICALM |
| GO:0051015~actin filament binding | 7 | 4.605 | 4.144 | 0.007 | AFDN, TPM1, DMD, RCSD1, ANTXR1, MYO1F, CLASP2 |
| GO:0005515~protein binding | 51 | 33.553 | 1.301 | 0.023 | LRRK2, NGLY1, PTEN, LAMC1, SLC4A4, OPHN1, NRD1, BBS9, SBF2, JAK1, UNC13A, FBXW11, DYNLT3, TPM1, KRT5, SYP, TNFRSF1B, TOM1L1, CLDN11, CTDSP1, IL1RAPL1, ARHGEF6, LEPROT, SHC3, PRICKLE2, DERL2, KATNB1, NYAP2, NTN1, CAMSAP2, GRIN2A, PALMD, DPH1, BAG3, DMD, MAP4, PAK3, CLASP2, GPR17, CEP350, ERBIN, M6PR, ATP2B4, LAMB1, AFDN, EXT2, EHD2, RPS6KB1, KRT14, NF2, RECK |
| GO:0008270~zinc ion binding | 12 | 7.895 | 2.146 | 0.024 | TRIM12C, NPEPPS, GRIN2A, RPS29, MMP15, CNBP, PRICKLE2, CDIP1, CAR11, DMD, MT1, MT3 |
| GO:0003779~actin binding | 8 | 5.263 | 2.810 | 0.024 | OPHN1, LRRK2, TPM1, DMD, NF2, FMN2, MYO1F, SSH1 |
| GO:0004722~protein serine/threonine phosphatase activity | 4 | 2.632 | 6.104 | 0.027 | CTDSP1, LRRK2, PTEN, SSH1 |
| GO:0046872~metal ion binding | 36 | 23.684 | 1.367 | 0.037 | DDR1, CUL9, NGLY1, ATP1A4, SLC1A2, PRICKLE2, ANTXR1, GNA14, NPEPPS, GRIN2A, NRD1, DPH1, PGM2, MGAT1, DMD, NT5M, RPS27A, PAK3, AASDHPPT, JAK1, UNC13C, UNC13A, TYW5, CNBP, ATP2B4, TAP1, MT1, MT3, EXT2, EHD2, NPLOC4, PDE10A, CTDSP1, MMP15, RPS29, CDIP1 |
| GO:0043208~glycosphingolipid binding | 2 | 1.316 | 45.780 | 0.043 | LAMB1, LAMC1 |
| GO:0035091~phosphatidylinositol binding | 4 | 2.632 | 5.087 | 0.044 | TOM1L1, SH3PXD2B, SNX11, SBF2 |
| CC Terms |  | | | | |
| GO:0016020~membrane | 84 | 55.263 | 1.561 | 0.000 | DDR1, PCDHGB6, TMEM151B, LRRK2, FMN2, PTPRK, LAMC1, SNX11, SLC4A4, ANTXR1, ATRAID, AQP1, NDST2, LMBR1, HYAL2, SEC61G, NAT8F1, GPR137, BBS9, SLC12A7, SBF2, JAK1, GSDMA2, CCDC51, UNC13C, UNC13A, ELOVL5, ALG14, TAP1, TMC7, SYP, TNFRSF1B, GABRG3, COPZ1, TOM1L1, CLDN11, MMP15, IL1RAPL1, DNAJC4, CDIP1, CD300E, SFT2D2, LY6E, EXOC1, LEPROT, RNASEK, TMPRSS5, SLC1A2, ATP1A4, PRICKLE2, DERL2, SLC41A1, GPR84, PTN, DPP4, GRIN2A, PALMD, BAG3, TMEM248, LEPROTL1, VTI1A, MGAT1, DMD, CLASP2, GPR17, CARMIL1, TCAF1, SLC10A7, ERBIN, M6PR, ATP2B4, LAMB1, MFSD14B, EXT2, EHD2, EFNA3, PDE10A, RPS6KB1, GGCX, RHAG, NF2, RECK, TOMM5, PICALM |
| GO:0042995~cell projection | 26 | 17.105 | 3.114 | 0.000 | LRRK2, PTEN, ATP1A4, ANTXR1, CAMSAP2, DPP4, GRIN2A, OPHN1, PALMD, NRD1, SH3PXD2B, BBS9, DMD, SBF2, CFAP45, CLASP2, UNC13C, CARMIL1, UNC13A, ELOVL5, CEP350, ATP2B4, SSH1, IL1RAPL1, NF2, ARHGEF6 |
| GO:0030424~axon | 15 | 9.868 | 4.305 | 0.000 | UNC13A, LRRK2, SLC1A2, KATNB1, PTPRK, TNFRSF1B, MT3, AQP1, CLDN11, AFDN, IL1RAPL1, HMBS, DMD, MAP4, SBF2 |
| GO:0005737~cytoplasm | 73 | 48.026 | 1.436 | 0.000 | DDR1, LRRK2, NGLY1, PTEN, ARHGEF10L, FMN2, AQP1, NPEPPS, OPHN1, HYAL2, SH3PXD2B, BBS9, PGM2, CEP97, SBF2, CFAP45, JAK1, GSDMA2, UNC13C, NAA30, UNC13A, FBXW11, DYNLT3, ANGEL2, TPM1, KRT5, COPZ1, TOM1L1, EML5, GAN, MAP7D1, HMBS, IL1RAPL1, GEMIN5, EXOC1, ARHGEF6, CUL9, TRIM12C, PRICKLE2, KATNB1, PTN, NTN1, CAMSAP2, PALMD, DPH1, BAG3, MGAT1, DMD, MAP4, PAK3, RPS27A, AASDHPPT, CLASP2, EIF2B4, CARMIL1, CHKB, CEP350, CNBP, ERBIN, SCLT1, MT1, MT3, SSH1, AFDN, EHD2, NPLOC4, PDE10A, RPS6KB1, RPS29, KRT14, NF2, MYO1F, MDM1 |
| GO:0045202~synapse | 19 | 12.500 | 2.751 | 0.000 | UNC13C, SHC3, UNC13A, LRRK2, PTEN, SLC1A2, SYP, GABRG3, SSH1, GRIN2A, OPHN1, PALMD, RPS6KB1, DMD, NF2, RPS27A, SLC12A7, LY6E, PICALM |
| GO:0005856~cytoskeleton | 23 | 15.132 | 2.378 | 0.000 | CARMIL1, CEP350, DYNLT3, TPM1, SCLT1, KATNB1, FMN2, CAMSAP2, SSH1, EML5, GAN, MAP7D1, KRT14, BBS9, DMD, NF2, MAP4, CEP97, PLEKHH1, CFAP45, JAK1, CLASP2, MDM1 |
| GO:0043195~terminal bouton | 6 | 3.947 | 9.633 | 0.000 | UNC13C, GRIN2A, UNC13A, OPHN1, LRRK2, SYP |
| GO:0031594~neuromuscular junction | 6 | 3.947 | 7.969 | 0.001 | UNC13C, UNC13A, ERBIN, SYP, LAMC1, PTN |
| GO:0005829~cytosol | 45 | 29.605 | 1.550 | 0.002 | CUL9, TRIM12C, LRRK2, NGLY1, PTEN, KATNB1, ARHGEF10L, FMN2, AFF3, NTN1, CAMSAP2, NPEPPS, BAG3, HYAL2, VTI1A, PGM2, MAP4, RPS27A, CEP97, PAK3, SBF2, AASDHPPT, JAK1, CLASP2, GSDMA2, CARMIL1, NAA30, FBXW11, CNBP, TPM1, MT1, MT3, SSH1, TOM1L1, AFDN, EHD2, NPLOC4, RPS6KB1, RPS29, MAP7D1, HMBS, GEMIN5, NF2, MYO1F, MDM1 |
| GO:0098794~postsynapse | 8 | 5.263 | 3.989 | 0.004 | GRIN2A, RPS6KB1, LRRK2, ERBIN, PTN, RPS27A, GABRG3, PICALM |
| GO:0045121~membrane raft | 8 | 5.263 | 3.619 | 0.007 | DPP4, HYAL2, LRRK2, SLC1A2, ATP2B4, DMD, NF2, TNFRSF1B |
| GO:0042734~presynaptic membrane | 6 | 3.947 | 4.816 | 0.008 | UNC13C, GRIN2A, UNC13A, SLC1A2, SYP, PICALM |
| GO:0008021~synaptic vesicle | 6 | 3.947 | 4.764 | 0.009 | GRIN2A, LRRK2, VTI1A, SYP, MT3, PICALM |
| GO:0005768~endosome | 12 | 7.895 | 2.412 | 0.011 | TOM1L1, EHD2, LEPROT, LEPROTL1, LRRK2, M6PR, VTI1A, CDIP1, SNX11, PAK3, SBF2, PICALM |
| GO:0005874~microtubule | 8 | 5.263 | 3.193 | 0.013 | EML5, DYNLT3, KATNB1, MAP4, MT3, CLASP2, MDM1, CAMSAP2 |
| GO:0005886~plasma membrane | 56 | 36.842 | 1.319 | 0.013 | DDR1, PCDHGB6, LRRK2, PTEN, FMN2, PTPRK, SLC4A4, ANTXR1, ATRAID, AQP1, LMBR1, HYAL2, BBS9, SLC12A7, GSDMA2, UNC13C, UNC13A, TMC7, KRT5, GABRG3, CLDN11, MMP15, IL1RAPL1, CD300E, LY6E, EXOC1, SHC3, TMPRSS5, SLC1A2, ATP1A4, KATNB1, SLC41A1, GPR84, PTN, DPP4, GRIN2A, BAG3, DMD, MAP4, CLASP2, GPR17, CARMIL1, TCAF1, SLC10A7, ERBIN, ATP2B4, MT3, SSH1, AFDN, EHD2, EFNA3, RHAG, NF2, RECK, MYO1F, PICALM |
| GO:0043025~neuronal cell body | 11 | 7.237 | 2.457 | 0.014 | PDE10A, TMPRSS5, ELOVL5, FBXW11, LRRK2, VTI1A, KATNB1, DMD, PTPRK, TNFRSF1B, PICALM |
| GO:0043197~dendritic spine | 6 | 3.947 | 4.115 | 0.015 | GRIN2A, PALMD, OPHN1, PTEN, SLC1A2, MT3 |
| GO:0030672~synaptic vesicle membrane | 5 | 3.289 | 5.144 | 0.016 | UNC13C, UNC13A, LRRK2, DMD, SYP |
| GO:0005606~laminin-1 complex | 2 | 1.316 | 97.398 | 0.020 | LAMB1, LAMC1 |
| GO:0043259~laminin-10 complex | 2 | 1.316 | 97.398 | 0.020 | LAMB1, LAMC1 |
| GO:0030054~cell junction | 9 | 5.921 | 2.619 | 0.022 | CLDN11, DPP4, AFDN, PUF60, DPH1, SH3PXD2B, ERBIN, DMD, PTPRK |
| GO:0005902~microvillus | 4 | 2.632 | 6.566 | 0.023 | HYAL2, LRRK2, FMN2, MYO1F |
| GO:0043541~UDP-N-acetylglucosamine transferase complex | 2 | 1.316 | 73.049 | 0.027 | EXT2, ALG14 |
| GO:0000139~Golgi membrane | 9 | 5.921 | 2.458 | 0.030 | NDST2, EXT2, LEPROT, SLC10A7, HYAL2, LRRK2, VTI1A, MGAT1, COPZ1 |
| GO:0098978~glutamatergic synapse | 10 | 6.579 | 2.230 | 0.035 | GRIN2A, UNC13A, RPS6KB1, OPHN1, LRRK2, IL1RAPL1, ERBIN, SLC1A2, ATP2B4, PAK3 |
| GO:0060076~excitatory synapse | 3 | 1.974 | 9.131 | 0.042 | AFDN, UNC13A, SYP |
| GO:0005741~mitochondrial outer membrane | 5 | 3.289 | 3.765 | 0.044 | RPS6KB1, LRRK2, DMD, MT3, TOMM5 |
| GO:0098793~presynapse | 6 | 3.947 | 3.108 | 0.044 | UNC13C, UNC13A, OPHN1, SYP, PTN, RPS27A |
| GO:0014069~postsynaptic density | 7 | 4.605 | 2.684 | 0.046 | GRIN2A, DMD, SYP, MAP4, PAK3, MT3, PICALM |
| GO:0045211~postsynaptic membrane | 6 | 3.947 | 3.054 | 0.047 | GRIN2A, IL1RAPL1, PTEN, DMD, GABRG3, PICALM |
| GO:0005604~basement membrane | 4 | 2.632 | 4.911 | 0.048 | LAMB1, LAMC1, PTN, NTN1 |
| GO:0030027~lamellipodium | 5 | 3.289 | 3.634 | 0.049 | DPP4, CARMIL1, DMD, NF2, ARHGEF6 |
| GO:0005887~integral component of plasma membrane | 15 | 9.868 | 1.735 | 0.049 | DDR1, GPR17, ATP2B4, SLC1A2, TMC7, GPR84, SLC4A4, GABRG3, AQP1, CLDN11, LMBR1, GRIN2A, MMP15, RHAG, SLC12A7 |
| KEGG Terms |  | | | | |
| mmu04964:Proximal tubule bicarbonate reclamation | 3 | 1.974 | 17.808 | 0.012 | ATP1A4, SLC4A4, AQP1 |
| mmu01521:EGFR tyrosine kinase inhibitor resistance | 4 | 2.632 | 6.612 | 0.022 | SHC3, RPS6KB1, PTEN, JAK1 |
| mmu04014:Ras signaling pathway | 6 | 3.947 | 3.334 | 0.032 | AFDN, EFNA3, GRIN2A, SHC3, RASAL2, PAK3 |
| mmu05170:Human immunodeficiency virus 1 infection | 6 | 3.947 | 3.265 | 0.034 | TRIM12C, RPS6KB1, FBXW11, TAP1, PAK3, TNFRSF1B |
