# Supplementary material for: The ultrastructural and proteomic analysis of mitochondria‐associated endoplasmic reticulum membrane in the midbrain of a Parkinson's disease mouse model
Source: Aging Cell. 2024 Nov 29;24(4):e14436. doi: 10.1111/acel.14436 (PMC11984660; doi:10.1111/acel.14436)
Supplement: Supplementary file 17 — Table S11. GSEA data for all detected proteins in MAM proteomics. [file ACEL-24-e14436-s021.docx]

**Supplementary Table 11 GSEA data for all detected proteins in MAM proteomics**

| ID | Description | Set size | Enrichment Score | NES | p value | Core enrichment genes |
| --- | --- | --- | --- | --- | --- | --- |
| BP Terms | | | | | | |
| GO:0035493 | SNARE complex assembly | 17 | 0.806 | 2.018 | 0.000 | Vamp8/Uvrag/Vamp2/Vps11/Vps18/Vamp3/Vamp7/Snap25 |
| GO:0006637 | acyl-CoA metabolic process | 61 | -0.533 | -1.873 | 0.000 | Vamp8/Uvrag/Vamp2/Vps11/Vps18/Vamp3/Vamp7/Snap25 |
| GO:0035383 | thioester metabolic process | 61 | -0.533 | -1.873 | 0.000 | Pdk1/Acaca/Mvd/Acadsb/Acsl3/Far1/Acot1/Acsl6/Acot6/Acot2/Acat1/Acsl1/Acly/Acot8/Acss1/Acsl5/Acot7/Hsd17b4/Mpc1/Acot9/Abcd1 |
| GO:0046718 | viral entry into host cell | 32 | 0.684 | 1.960 | 0.000 | Vamp8/Uvrag/Vps16/Vps18 |
| GO:0006631 | fatty acid metabolic process | 208 | -0.378 | -1.591 | 0.000 | Cyp2u1/Elovl2/Sgpl1/Lpin1/Fads6/Eci2/Gnpat/Echdc1/Cav1/Cygb/Akt1/Agt/Etfa/Lipa/Aldh3a2/Lipe/Pdk1/Acaca/Gpx4/Acad9/Abcd4/Acadm/Acsbg1/Acadsb/Acsl3/Ephx1/Etfb/Aacs/Acadl/Acot1/Cnr1/Dbi/C3/Mif/Acsl6/Acot6/Acot2/Abhd3/Acat1/Acsf3/Cyp2s1/Acsl1/Cpt1a/Acly/Acot8/Abcd3/Apoa4/Cd36/Acaa1a/Abcd2/Pex13/Alox5ap/Acad11/Appl2/Adipor2/Acox1/Acss1/Acsl5/Acot7/Acadvl/Abhd5/Hsd17b4/Abcd1/Wdtc1 |
| GO:0044242 | cellular lipid catabolic process | 137 | -0.420 | -1.703 | 0.000 | Gba2/Sgpl1/Lpin1/Eci2/Echdc1/Hexa/Akt1/Gla/Etfa/Gdpd1/Lipe/Inpp5f/Srd5a3/Asah1/Idh1/Abcd4/Acadm/Apoa2/Etfb/Acadl/Naga/Cnr1/Abhd6/Dbi/Abhd12/Acot2/Abhd3/Abhd16a/Acat1/Bscl2/Cpt1a/Acot8/Abcd3/Apoa4/Acaa1a/Apoh/Abcd2/Pex13/Acad11/Acox1/Acsl5/Acot7/Acadvl/Abhd5/Hsd17b4/Abcd1 |
| GO:0050996 | positive regulation of lipid catabolic process | 16 | -0.735 | -1.869 | 0.000 | Apoa2/Cpt1a/Apoa4/Apoh/Abcd2/Acsl5/Abhd5/Abcd1 |
| GO:0033865 | nucleoside bisphosphate metabolic process | 72 | -0.506 | -1.824 | 0.000 | Mvd/Acadsb/Mccc2/Acsl3/Far1/Coasy/Acot1/Acsl6/Acot6/Acot2/Acat1/Acsl1/Acly/Acot8/Acss1/Acsl5/Acot7/Hsd17b4/Mpc1/Acot9/Abcd1 |
| GO:0033875 | ribonucleoside bisphosphate metabolic process | 72 | -0.506 | -1.824 | 0.000 | Mvd/Acadsb/Mccc2/Acsl3/Far1/Coasy/Acot1/Acsl6/Acot6/Acot2/Acat1/Acsl1/Acly/Acot8/Acss1/Acsl5/Acot7/Hsd17b4/Mpc1/Acot9/Abcd1 |
| GO:0034032 | purine nucleoside bisphosphate metabolic process | 72 | -0.506 | -1.824 | 0.000 | Mvd/Acadsb/Mccc2/Acsl3/Far1/Coasy/Acot1/Acsl6/Acot6/Acot2/Acat1/Acsl1/Acly/Acot8/Acss1/Acsl5/Acot7/Hsd17b4/Mpc1/Acot9/Abcd1 |
| GO:0045922 | negative regulation of fatty acid metabolic process | 10 | -0.823 | -1.862 | 0.000 | Acadl/Cnr1/Dbi/Appl2/Acadvl/Wdtc1 |
| GO:0140029 | exocytic process | 81 | 0.526 | 1.800 | 0.001 | Vamp8/Vamp2/Vps11/Ykt6/Vps33a/Napb/Vps18/Vps33b/Vamp7/Pclo/Snapin/Nlgn1/Nrxn1/Snap25/Ncam1 |
| GO:0007031 | peroxisome organization | 29 | -0.638 | -1.843 | 0.001 | Pex11b/Abcd4/Rab8b/Mavs/Pex12/Acot8/Abcd3/Abcd2/Pex13/Pex10/Acox1/Abcd1 |
| GO:0032787 | monocarboxylic acid metabolic process | 304 | -0.333 | -1.459 | 0.001 | Slc1a3/Cyp2u1/Elovl2/Sgpl1/Lpin1/Fads6/Eci2/Gnpat/Echdc1/Gm3839/Cav1/Cygb/Akt1/Agt/Etfa/Lipa/Aldh3a2/Lipe/Insr/Pdk1/Ldhb/Adpgk/Mtch2/Acaca/Gpx4/Acad9/Idh1/Abcd4/Acadm/Acsbg1/Pnkd/Acadsb/Acsl3/Hk2/Ephx1/Etfb/Aldh1a1/Aacs/Acadl/Gapdh/Acot1/Cnr1/Dbi/Aldoc/C3/Mif/Acsl6/Acot6/Acot2/Abhd3/Acat1/Ugt1a7c/Akr1a1/Acsf3/Cyp2s1/Acsl1/Aldh1a2/Cpt1a/Acly/Acot8/Abcd3/Apoa4/Cd36/Acaa1a/Abcd2/Pex13/Alox5ap/Acad11/Appl2/Adipor2/Acox1/Acss1/Acsl5/Acot7/Acadvl/Abhd5/Hsd17b4/Abat/Mpc1/Abcd1/Wdtc1 |
| GO:0008154 | actin polymerization or depolymerization | 144 | -0.402 | -1.621 | 0.001 | Pycard/Gmfb/Gba2/Snx9/Mical3/Brk1/Pfn2/Rhoa/Mtpn/Arhgap35/Capn1/Ctnna2/Ap1ar/Wdr1/Evl/Coro1b/Myadm/Spta1/Wasf3/Bin1/Bbs4/Coro1a/Cttn/Dstn/Enah/Twf1/Capza1/Myo1c/Arpc1b/Plek/Carmil1/Cyfip1/Arf1/Cfl1/Wasl/Arpc1a/Add2/Washc1/Arfgef1/Bag4/Arpc2/Aif1/Wasf2/Abi1/Twf2/Wasf1/Abi2 |
| GO:0046395 | carboxylic acid catabolic process | 124 | -0.424 | -1.682 | 0.001 | Sardh/Abcd4/Acadm/Ddah1/Mccc2/Etfb/Gcsh/Acadl/Cnr1/Dbi/Bckdhb/Acot2/Abhd3/Acat1/Akr1a1/Acsf3/Cpt1a/Acot8/Abcd3/Acaa1a/Abcd2/Pex13/Acad11/Gcat/Acox1/Acsl5/Acot7/Acadvl/Hsd17b4/Ddo/Abat/Dao/Abcd1 |
| GO:0072329 | monocarboxylic acid catabolic process | 74 | -0.476 | -1.717 | 0.001 | Abcd4/Acadm/Etfb/Acadl/Cnr1/Dbi/Acot2/Abhd3/Acat1/Akr1a1/Cpt1a/Acot8/Abcd3/Acaa1a/Abcd2/Pex13/Acad11/Acox1/Acsl5/Acot7/Acadvl/Hsd17b4/Abcd1 |
| GO:0044409 | entry into host | 35 | 0.641 | 1.872 | 0.001 | Vamp8/Uvrag/Vps16/Vps18 |
| GO:0009150 | purine ribonucleotide metabolic process | 211 | -0.361 | -1.526 | 0.001 | Cacnb4/Insr/Pdk1/Atp5h/Adpgk/Mtch2/Sphk2/Acaca/Ak1/Adcy8/Nudt10/Mvd/Acadsb/Uqcc3/Mccc2/Acsl3/Hk2/Adss/Far1/Coasy/Atp5j2/Adcy5/Pfas/Nudt2/Gapdh/Acot1/Aldoc/Adssl1/Mif/Acsl6/Acot6/Acot2/Acat1/Acsl1/Acly/Acot8/Itpa/Ak2/Ada/Adcy10/Acss1/Acsl5/Acot7/Hsd17b4/Ampd3/Atp2b2/Mpc1/Acot9/Abcd1/Ak4/Adcy6 |
| GO:0048284 | organelle fusion | 112 | 0.486 | 1.739 | 0.001 | Vamp8/Vcpip1/Uvrag/Vamp2/Vps11/Bnip3/Vps16/Vps39/Vps18/Vav3/Vamp3/Vamp7/Chchd3/Snapin/Usp30/Eno3/Snap25/Vti1a/Bnip1 |
| GO:0008064 | regulation of actin polymerization or depolymerization | 131 | -0.410 | -1.646 | 0.001 | Pycard/Gmfb/Gba2/Snx9/Brk1/Pfn2/Rhoa/Mtpn/Arhgap35/Capn1/Ctnna2/Ap1ar/Wdr1/Evl/Coro1b/Myadm/Spta1/Wasf3/Bin1/Bbs4/Coro1a/Cttn/Dstn/Twf1/Capza1/Myo1c/Arpc1b/Plek/Carmil1/Cyfip1/Arf1/Cfl1/Wasl/Arpc1a/Add2/Washc1/Arfgef1/Bag4/Arpc2/Wasf2/Twf2/Wasf1/Abi2 |
| GO:0030832 | regulation of actin filament length | 131 | -0.410 | -1.646 | 0.001 | Pycard/Gmfb/Gba2/Snx9/Brk1/Pfn2/Rhoa/Mtpn/Arhgap35/Capn1/Ctnna2/Ap1ar/Wdr1/Evl/Coro1b/Myadm/Spta1/Wasf3/Bin1/Bbs4/Coro1a/Cttn/Dstn/Twf1/Capza1/Myo1c/Arpc1b/Plek/Carmil1/Cyfip1/Arf1/Cfl1/Wasl/Arpc1a/Add2/Washc1/Arfgef1/Bag4/Arpc2/Wasf2/Twf2/Wasf1/Abi2 |
| GO:0019752 | carboxylic acid metabolic process | 483 | -0.297 | -1.361 | 0.001 | Atcay/Apip/Atic/Apc/Etfa/Ears2/Lipa/Aldh3a2/Npl/Iars/Lipe/Insr/Pdk1/Nars/Ldhb/Adpgk/Mtch2/Bcat2/Acaca/Gpx4/Acad9/Uros/Idh1/Sardh/Abcd4/Acadm/Acsbg1/Pnkd/Acadsb/Ddah1/Mccc2/Mdh2/Acsl3/Comt/Hk2/Ephx1/Adss/Etfb/Gcsh/Aldh1a1/Aacs/Icmt/Pfas/Acadl/Gapdh/Acot1/Yars2/Cnr1/Dbi/Wars2/Aldoc/C3/Adssl1/Mif/Bckdhb/Acsl6/Acot6/Acot2/Abhd3/Acat1/Ugt1a7c/Akr1a1/Acsf3/Cyp2s1/Aldh18a1/Cars2/Wars/Acsl1/Aldh1a2/Aco1/Cpt1a/Acly/Acot8/Abcd3/Apoa4/Idh3b/Cd36/Aasdhppt/Acaa1a/Abcd2/Pex13/Alox5ap/Acad11/Cth/Gcat/Appl2/Adipor2/Cars/Vars2/Acox1/Acss1/Acsl5/Acot7/Acadvl/Aars2/Abhd5/Hsd17b4/Aco2/Ddo/Abat/Aars/Mpc1/Dao/Abcd1/Wdtc1 |
| GO:0030833 | regulation of actin filament polymerization | 118 | -0.415 | -1.637 | 0.001 | Pycard/Gmfb/Gba2/Snx9/Brk1/Pfn2/Rhoa/Mtpn/Capn1/Ctnna2/Ap1ar/Evl/Coro1b/Myadm/Spta1/Wasf3/Bin1/Bbs4/Coro1a/Cttn/Twf1/Capza1/Myo1c/Arpc1b/Carmil1/Cyfip1/Arf1/Cfl1/Wasl/Arpc1a/Add2/Washc1/Arfgef1/Bag4/Arpc2/Wasf2/Twf2/Wasf1/Abi2 |
| GO:0016054 | organic acid catabolic process | 132 | -0.411 | -1.655 | 0.001 | Lpin1/Nagk/Eci2/Echdc1/Akt1/Blmh/Etfa/Npl/Lipe/Bckdha/Bcat2/Sardh/Abcd4/Acadm/Acadsb/Ddah1/Mccc2/Etfb/Gcsh/Acadl/Cnr1/Dbi/Bckdhb/Acot2/Abhd3/Acat1/Akr1a1/Acsf3/Cpt1a/Acot8/Abcd3/Acaa1a/Abcd2/Pex13/Acad11/Gcat/Acox1/Acsl5/Acot7/Acadvl/Hsd17b4/Ddo/Abat/Dao/Abcd1 |
| GO:0032446 | protein modification by small protein conjugation | 273 | 0.388 | 1.544 | 0.001 | Ube2j1/Ubr3/Ube2k/Zswim2/Vcpip1/Ube2j2/Urm1/Vps11/Vps28/Ube2m/Uba7/Ube2i/Cdc23/Vps18/Uba2/Ube2v2/Ube2d1/Ube2l3/Klhl9/Atg3/Pja1/Septin4/Usp4/Uba3/Vcp/Cul5/Fgfr3/Nub1/Rpl23/Ube2a/Ube2n/Isg15/Rnf167/Uba5/Ltn1/Cul9/Sharpin/Ctnnb1/Hace1/Wwp1/Uba1/Bcl2/Vhl/Tollip/Tbc1d7/Prpf19/Wdr48/Anapc13/Ubr2/Smurf2/Hecw1/Traf3/Ufl1/Ddrgk1/Marchf6/Mgrn1/Ube2f/Fbxo7/Ube2g2/Gclc/Fkbp1a/Ubr1/Herc4/Arrdc3/Commd1/Atg7/Aktip/Pten/Nedd4/Os9/Rc3h1/Arih2 |
| GO:0031334 | positive regulation of protein-containing complex assembly | 148 | -0.398 | -1.607 | 0.001 | Pycard/Gmfb/Cck/Snx9/Brk1/Pfn2/Rhoa/Cav1/Ctnna2/Ap1ar/Tfrc/Evl/Bak1/Coro1b/Apc/Wasf3/Bin1/Piezo1/Bid/Atat1/Coro1a/Cttn/Psmc3/Myo1c/Arpc1b/Plek/Caly/Carmil1/Cyfip1/Fes/Psmc2/Drg1/Arf1/Wasl/Arpc1a/Wars/Cd36/Msn/Washc1/Bag4/Arpc2/Abca3/Abca1/Wasf2/Wasf1/Abi2 |
| GO:0009062 | fatty acid catabolic process | 67 | -0.485 | -1.730 | 0.001 | Abcd4/Acadm/Etfb/Acadl/Cnr1/Dbi/Acot2/Abhd3/Acat1/Cpt1a/Acot8/Abcd3/Acaa1a/Abcd2/Pex13/Acad11/Acox1/Acsl5/Acot7/Acadvl/Hsd17b4/Abcd1 |
| GO:0000038 | very long-chain fatty acid metabolic process | 23 | -0.661 | -1.832 | 0.001 | Abcd4/Acsbg1/Acsl6/Acot2/Acsl1/Abcd3/Acaa1a/Abcd2/Acox1/Hsd17b4/Abcd1 |
| GO:0009259 | ribonucleotide metabolic process | 221 | -0.346 | -1.480 | 0.001 | Cacnb4/Insr/Pdk1/Atp5h/Adpgk/Mtch2/Sphk2/Acaca/Ak1/Adcy8/Nudt10/Mvd/Acadsb/Uqcc3/Mccc2/Acsl3/Hk2/Adss/Far1/Coasy/Atp5j2/Entpd4/Adcy5/Pfas/Nudt2/Gapdh/Acot1/Aldoc/Adssl1/Mif/Acsl6/Acot6/Acot2/Acat1/Acsl1/Acly/Acot8/Itpa/Ak2/Ada/Adcy10/Cmpk1/Acss1/Acsl5/Acot7/Hsd17b4/Ampd3/Atp2b2/Mpc1/Acot9/Abcd1/Ak4/Adcy6 |
| GO:0051258 | protein polymerization | 203 | -0.355 | -1.491 | 0.001 | Pycard/Hdgfl3/Gmfb/Gba2/Snx9/Brk1/Pfn2/Dyrk1a/Rhoa/Dnm3/Cav1/Mtpn/Fgb/Capn1/Ctnna2/Ap1ar/Evl/Coro1b/Apc/Myadm/Spta1/Wasf3/Bin1/Gpx4/Bbs4/Coro1a/Chmp3/Cttn/Tubgcp3/Twf1/Capza1/Myo1c/Arpc1b/Carmil1/Cyfip1/Fes/Drg1/Arf1/Cfl1/Wasl/Fgf13/Arpc1a/Add2/Washc1/Arfgef1/Bag4/Arpc2/Chmp2a/Aif1/Wasf2/Vtn/Tubgcp4/Twf2/Wasf1/Abi2/Tubg2/Vdac2/Tubgcp6 |
| GO:0044257 | cellular protein catabolic process | 452 | 0.343 | 1.425 | 0.001 | Uqcc2/Vps37a/Ube2j1/Ubr3/Ube2k/Usp9x/Svip/Wdr91/Aup1/Yme1l1/Ube2j2/Vps11/Vps28/Bnip3/Usp8/Uba7/Ube2i/Cdc23/Psmd1/Ubap1/Ube2v2/Ube2d1/Ube2l3/Brsk2/Usp4/Usp47/Amn1/Vcp/Agbl4/Cul5/Nub1/Usp46/Ubxn6/Uchl1/Rpl23/Tmem67/Usp30/Ube2a/Ube2n/Isg15/Rnf167/Ltn1/Vps35/Ptpn23/Cul9/Zmpste24/Sharpin/Dcaf11/Ctnnb1/Ubac2/Hace1/Ctsf/Mgat3/Eif3h/Ddi2/H13/Wwp1/Ufd1/Uba1/Vps37c/Pdcd6ip/Vhl/Tollip/Atpif1/Ccar2/Prpf19/Clpx/Usp24/Dnajb12/Vps25/Zfand2b/Map1a/Ubr2/Smurf2/Hecw1/Ufl1/Lrp1/Psmd2/Pomt2/Atxn3/Ddrgk1/Marchf6/Usp7/Tpp1/Get4/Fbxo7/Ube2g2/Gclc/Ubr1/Csnk1d/Erlin1/Commd1/Kctd6/Atg7/Pten/Nedd4/Os9/Chmp4b/Rc3h1/Abca2/Arih2/Ezr/Otud7b/Fbxl18 |
| GO:0019693 | ribose phosphate metabolic process | 230 | -0.337 | -1.443 | 0.001 | Cacnb4/Insr/Pdk1/Atp5h/Adpgk/Mtch2/Sphk2/Acaca/Ak1/Adcy8/Nudt10/Mvd/Acadsb/Uqcc3/Mccc2/Acsl3/Hk2/Adss/Far1/Coasy/Atp5j2/Entpd4/Adcy5/Pfas/Nudt2/Gapdh/Acot1/Aldoc/Adssl1/Mif/Acsl6/Acot6/Acot2/Acat1/Acsl1/Acly/Acot8/Itpa/Ak2/Ada/Adcy10/Cmpk1/Acss1/Acsl5/Acot7/Hsd17b4/Ampd3/Atp2b2/Mpc1/Acot9/Abcd1/Ak4/Adcy6 |
| GO:0043436 | oxoacid metabolic process | 491 | -0.292 | -1.332 | 0.001 | Atcay/Apip/Atic/Apc/Etfa/Ears2/Lipa/Aldh3a2/Npl/Iars/Lipe/Insr/Pdk1/Nars/Ldhb/Adpgk/Mtch2/Bcat2/Acaca/Gpx4/Acad9/Uros/Idh1/Sardh/Abcd4/Acadm/Acsbg1/Pnkd/Acadsb/Ddah1/Mccc2/Mdh2/Acsl3/Comt/Hk2/Ephx1/Adss/Etfb/Gcsh/Aldh1a1/Aacs/Icmt/Pfas/Acadl/Gapdh/Acot1/Yars2/Cnr1/Dbi/Wars2/Aldoc/C3/Adssl1/Mif/Bckdhb/Acsl6/Acot6/Acot2/Abhd3/Acat1/Ugt1a7c/Akr1a1/Acsf3/Cyp2s1/Aldh18a1/Cars2/Wars/Acsl1/Aldh1a2/Aco1/Cpt1a/Acly/Acot8/Abcd3/Apoa4/Idh3b/Cd36/Aasdhppt/Acaa1a/Abcd2/Pex13/Alox5ap/Acad11/Cth/Gcat/Appl2/Adipor2/Cars/Vars2/Acox1/Acss1/Acsl5/Acot7/Acadvl/Aars2/Abhd5/Hsd17b4/Aco2/Ddo/Abat/Aars/Mpc1/Dao/Abcd1/Wdtc1 |
| GO:0061025 | membrane fusion | 103 | 0.470 | 1.660 | 0.002 | Vamp8/Vcpip1/Uvrag/Vamp2/Vps11/Vps39/Vps18/Vav3/Vamp3/Vps33b/Vamp7/Snapin/Snap25/Folr1/Vti1a/Hace1/Bnip1 |
| GO:0015919 | peroxisomal membrane transport | 18 | -0.688 | -1.781 | 0.002 | Abcd4/Rab8b/Pex12/Abcd3/Abcd2/Pex13/Pex10/Abcd1 |
| GO:0051055 | negative regulation of lipid biosynthetic process | 18 | -0.688 | -1.782 | 0.002 | Hrh1/Dkk3/Acadl/Atp1a1/Acadvl/Wdtc1 |
| GO:0019216 | regulation of lipid metabolic process | 179 | -0.364 | -1.506 | 0.002 | Asah1/Idh1/Disp3/Bbs4/Apoa2/Phb2/Acsl3/Hrh1/Fmc1/Dkk3/Mtmr2/Apod/Fmo5/Dnajc15/Acadl/Tspo/Arf1/Cnr1/Abhd6/Dbi/C3/H6pd/Bscl2/Cpt1a/Atp1a1/Apoa4/Cd36/Ttc39b/Apoh/Abcd2/Washc1/Abca7/Appl2/Epha8/Acsl5/Acadvl/Abhd5/Abca3/Abcd1/Atg14/Wdtc1 |
| GO:0070647 | protein modification by small protein conjugation or removal | 332 | 0.363 | 1.468 | 0.002 | Ube2j1/Ubr3/Ube2k/Usp9x/Zswim2/Vcpip1/Ube2j2/Urm1/Usp54/Vps11/Vps28/Usp8/Ube2m/Uba7/Ube2i/Cdc23/Vps18/Uba2/Ube2v2/Ube2d1/Ube2l3/Klhl9/Atg3/Pja1/Septin4/Usp4/Usp47/Uba3/Vcp/Cul5/Fgfr3/Nub1/Usp46/Uchl1/Rpl23/Usp30/Ube2a/Ube2n/Isg15/Rnf167/Uba5/Ltn1/Cul9/Sharpin/Senp8/Ctnnb1/Hace1/Wwp1/Uba1/Bcl2/Usp35/Vhl/Tollip/Tbc1d7/Prpf19/Wdr48/Abraxas2/Usp24/Anapc13/Ubr2/Smurf2/Hecw1/Traf3/Ufl1/Atxn3/Ddrgk1/Marchf6/Usp7/Mgrn1/Ube2f/Fbxo7/Ube2g2/Gclc/Fkbp1a/Ubr1/Herc4/Arrdc3/Commd1/Atg7/Aktip/Pten/Nedd4/Os9/Rc3h1/Arih2/Gps1/Otud7b |
| GO:0010591 | regulation of lamellipodium assembly | 27 | -0.618 | -1.760 | 0.002 | Aqp1/Twf1/Cyfip1/Cfl1/Arpc2/Wasf2/Twf2/Abi2 |
| GO:0090174 | organelle membrane fusion | 85 | 0.490 | 1.693 | 0.002 | Vamp8/Vcpip1/Uvrag/Vamp2/Vps11/Vps39/Vps18/Vav3/Vamp3/Vamp7/Snapin/Snap25/Vti1a/Bnip1 |
| GO:0044282 | small molecule catabolic process | 195 | -0.354 | -1.475 | 0.002 | Aig1/Inpp5k/Shmt2/Mgat1/Acat2/Pfkl/Ppat/Slc27a4/Pnp/Qdpr/Cda/Mtmr7/Bad/Synj2/Pipox/Inpp1/Haghl/Acad8/Acat3/Glo1/Aldh1l1/Gmpr2/Lpin1/Nagk/Esd/Eci2/Echdc1/Akt1/Blmh/Etfa/Npl/Lipe/Bckdha/Dera/Inpp4b/Srd5a3/Bcat2/Sardh/Abcd4/Inpp5b/Acadm/Pnkd/Acadsb/Ddah1/Mccc2/Etfb/Gcsh/Mtmr2/Inpp5a/Acadl/Cnr1/Dbi/Bckdhb/Acot2/Abhd3/Acat1/Akr1a1/Acsf3/Cpt1a/Acot8/Abcd3/Acaa1a/Abcd2/Pex13/Ada/Acad11/Gcat/Acox1/Acsl5/Acot7/Acadvl/Hsd17b4/Ddo/Abat/Dao/Abcd1 |
| GO:0007032 | endosome organization | 37 | 0.584 | 1.726 | 0.002 | Tmem9/Vps11/Usp8/Washc4/Vps18/Hook3/Arfgef2/Vps33b/Coro1c/Chmp2b/Rab5c/Tmcc1/Als2 |
| GO:0009152 | purine ribonucleotide biosynthetic process | 92 | -0.442 | -1.669 | 0.002 | Dcakd/Aldoa/Impdh2/Aprt/Adcy1/Atp5j/Dld/Pnp/Acsl4/Adcy9/Gucy1b1/Paics/Flcn/Pdk2/Ampd2/Adsl/Adcy7/Atic/Atp5c1/Pdk1/Atp5h/Sphk2/Ak1/Adcy8/Uqcc3/Adss/Coasy/Atp5j2/Adcy5/Pfas/Nudt2/Adssl1/Acsl6/Acat1/Acsl1/Acly/Ak2/Adcy10/Acss1/Acsl5/Acot7/Ampd3/Mpc1/Ak4/Adcy6 |
| GO:0046890 | regulation of lipid biosynthetic process | 83 | -0.449 | -1.653 | 0.002 | Sphk2/Asah1/Idh1/Acsl3/Hrh1/Dkk3/Acadl/Tspo/Abhd6/Dbi/C3/H6pd/Bscl2/Atp1a1/Apoa4/Abcd2/Acsl5/Acadvl/Abca3/Abcd1/Wdtc1 |
| GO:0097352 | autophagosome maturation | 26 | 0.666 | 1.807 | 0.002 | Vamp8/Uvrag/Vps33a/Vps16/Fyco1/Vcp/Snapin |
| GO:0016042 | lipid catabolic process | 176 | -0.365 | -1.499 | 0.002 | Gba2/Sgpl1/Lpin1/Eci2/Echdc1/Hexa/Alk/Akt1/Gla/Etfa/Lipa/Gdpd1/Lipe/Inpp5f/Srd5a3/Asah1/Idh1/Abcd4/Acadm/Apoa2/Etfb/Fmc1/Plcxd3/Acadl/Naga/Cnr1/Abhd6/Dbi/Abhd12/Acot2/Abhd3/Abhd16a/Acat1/Ugt1a7c/Bscl2/Cpt1a/Acot8/Abcd3/Apoa4/Acaa1a/Apoh/Abcd2/Pex13/Acad11/Abhd4/Acox1/Acsl5/Acot7/Acadvl/Abhd5/Hsd17b4/Abcd1 |
| GO:0030838 | positive regulation of actin filament polymerization | 70 | -0.470 | -1.690 | 0.002 | Pycard/Gmfb/Snx9/Brk1/Pfn2/Rhoa/Ctnna2/Ap1ar/Evl/Coro1b/Wasf3/Bin1/Coro1a/Cttn/Myo1c/Arpc1b/Carmil1/Cyfip1/Arf1/Wasl/Arpc1a/Washc1/Bag4/Arpc2/Wasf2/Wasf1/Abi2 |
| GO:0072350 | tricarboxylic acid metabolic process | 13 | -0.726 | -1.758 | 0.003 | Idh1/Aco1/Acly/Idh3b/Aco2 |
| GO:0044728 | DNA methylation or demethylation | 20 | 0.697 | 1.803 | 0.003 | Usp9x/Tdrkh/Zmpste24/Fkbp6/Usp7 |
| GO:0045833 | negative regulation of lipid metabolic process | 44 | -0.535 | -1.763 | 0.003 | Apoa2/Hrh1/Fmc1/Dkk3/Apod/Acadl/Cnr1/Dbi/Bscl2/Atp1a1/Washc1/Appl2/Acadvl/Wdtc1 |
| GO:0070129 | regulation of mitochondrial translation | 15 | 0.754 | 1.822 | 0.003 | Uqcc2/Mief1/Rpusd3 |
| GO:0035337 | fatty-acyl-CoA metabolic process | 14 | -0.724 | -1.789 | 0.003 | Acsl3/Far1/Acsl6/Acsl1/Acsl5/Acot7/Hsd17b4/Abcd1 |
| GO:0009126 | purine nucleoside monophosphate metabolic process | 28 | -0.596 | -1.710 | 0.003 | Impdh2/Aprt/Ppat/Pnp/Paics/Ampd2/Gmpr2/Adsl/Atic/Nt5c1a/Ak1/Adss/Pfas/Nudt2/Adssl1/Ak2/Ada/Ampd3/Ak4 |
| GO:0003231 | cardiac ventricle development | 39 | 0.576 | 1.720 | 0.003 | Naca/Vangl2/Ryr2/Frs2/Luzp1/Hes1/Zmpste24/Fzd2/Ctnnb1/Ap2b1/Ly6e |
| GO:0006635 | fatty acid beta-oxidation | 51 | -0.499 | -1.681 | 0.003 | Abcd4/Acadm/Etfb/Acadl/Cnr1/Dbi/Acat1/Cpt1a/Abcd3/Acaa1a/Abcd2/Acad11/Acox1/Acsl5/Acadvl/Hsd17b4/Abcd1 |
| GO:0007620 | copulation | 11 | -0.777 | -1.820 | 0.003 | Cnr1/Ada/Vgf/Ddo/Abat |
| GO:0030104 | water homeostasis | 24 | -0.622 | -1.754 | 0.004 | Cdh1/Aqp1/Krt1/Ugcg/Anxa7/Stard7/Wfs1/Adcy6 |
| GO:0043574 | peroxisomal transport | 21 | -0.646 | -1.745 | 0.004 | Abcd4/Rab8b/Pex12/Abcd3/Abcd2/Pex13/Pex10/Abcd1 |
| GO:0006163 | purine nucleotide metabolic process | 224 | -0.334 | -1.428 | 0.004 | Cacnb4/Insr/Pdk1/Atp5h/Adpgk/Mtch2/Sphk2/Acaca/Nt5c1a/Ak1/Adcy8/Nudt10/Mvd/Acadsb/Uqcc3/Mccc2/Acsl3/Hk2/Adss/Far1/Coasy/Atp5j2/Adcy5/Pfas/Nudt2/Gapdh/Acot1/Aldoc/Adssl1/Mif/Acsl6/Acot6/Acot2/Acat1/Acsl1/Acly/Acot8/Itpa/Ak2/Ada/Adcy10/Acss1/Acsl5/Acot7/Hsd17b4/Ampd3/Atp2b2/Mpc1/Acot9/Abcd1/Ak4/Adcy6 |
| GO:0002696 | positive regulation of leukocyte activation | 107 | 0.454 | 1.613 | 0.004 | Vamp8/Adk/Slc39a10/Bloc1s3/Vcam1/Vav3/Hspd1/Cd276/Vamp7/Efnb2/Igkc/Hes1/Fgr/Pcid2/Bcl2/Atp11c/Dpp4/Gas6/Itgam/Mdk/Gpam/Prkcz/Ighm/Thy1/Efnb3/Pla2g4a |
| GO:0030041 | actin filament polymerization | 127 | -0.395 | -1.582 | 0.004 | Pycard/Gmfb/Gba2/Snx9/Brk1/Pfn2/Rhoa/Mtpn/Capn1/Ctnna2/Ap1ar/Evl/Coro1b/Myadm/Spta1/Wasf3/Bin1/Bbs4/Coro1a/Cttn/Twf1/Capza1/Myo1c/Arpc1b/Carmil1/Cyfip1/Arf1/Cfl1/Wasl/Arpc1a/Add2/Washc1/Arfgef1/Bag4/Arpc2/Aif1/Wasf2/Twf2/Wasf1/Abi2 |
| GO:0032543 | mitochondrial translation | 60 | 0.508 | 1.624 | 0.004 | Uqcc2/Mrps18b/Sars2/Mief1/Mrpl57/Mrpl43/Mtrf1l/Rpusd3/Rars2 |
| GO:0032273 | positive regulation of protein polymerization | 94 | -0.417 | -1.578 | 0.004 | Ptk2b/Mapre1/Pde4dip/Tppp3/Arpc3/Arl2/Mapk8/Grb2/Arpc5/Carmil2/Arfip1/Nck2/Ttbk1/Pycard/Gmfb/Snx9/Brk1/Pfn2/Rhoa/Cav1/Ctnna2/Ap1ar/Evl/Coro1b/Apc/Wasf3/Bin1/Coro1a/Cttn/Myo1c/Arpc1b/Carmil1/Cyfip1/Fes/Drg1/Arf1/Wasl/Arpc1a/Washc1/Bag4/Arpc2/Wasf2/Wasf1/Abi2 |
| GO:0042176 | regulation of protein catabolic process | 231 | 0.378 | 1.477 | 0.004 | Uqcc2/Ube2k/Svip/Wdr91/Rhbdd3/Tmem9/Vps11/Vps28/Usp8/Gpc3/Psmd1/Cst3/Ube2v2/Vcp/Agbl4/Nub1/Rpl23/Tmem67/Apc2/Vps35/Hace1/Mgat3/Eif3h/Flna/Wwp1/App/Vhl/Atpif1/Ccar2/Snca/Map1a/Smurf2/Hecw1/Ufl1/Lrp1/Psmd2/Atxn3/Ddrgk1/Usp7/Gclc/Csnk1d/Commd1/Atg7/Gpld1/Pten/Nedd4/Abca2/Arih2/Ezr |
| GO:0070131 | positive regulation of mitochondrial translation | 11 | 0.807 | 1.802 | 0.004 | Uqcc2/Mief1/Rpusd3 |
| GO:1902743 | regulation of lamellipodium organization | 34 | -0.543 | -1.631 | 0.004 | Brk1/Coro1b/Aqp1/Twf1/Carmil1/Cyfip1/Cfl1/Arpc2/Wasf2/Twf2/Abi2 |
| GO:0009167 | purine ribonucleoside monophosphate metabolic process | 27 | -0.592 | -1.686 | 0.004 | Impdh2/Aprt/Ppat/Pnp/Paics/Ampd2/Gmpr2/Adsl/Atic/Ak1/Adss/Pfas/Nudt2/Adssl1/Ak2/Ada/Ampd3/Ak4 |
| GO:0034440 | lipid oxidation | 70 | -0.457 | -1.642 | 0.004 | Abcd4/Acadm/Etfb/Apod/Acadl/Cnr1/Dbi/Acat1/Cpt1a/Abcd3/Cd36/Acaa1a/Abcd2/Pex13/Acad11/Appl2/Adipor2/Acox1/Acsl5/Acadvl/Hsd17b4/Abcd1 |
| GO:0031349 | positive regulation of defense response | 81 | 0.478 | 1.634 | 0.004 | Vamp8/Ube2k/Vav1/Fcgr1/Hspd1/Vamp7 |
| GO:0006906 | vesicle fusion | 80 | 0.479 | 1.634 | 0.005 | Vamp8/Uvrag/Vamp2/Vps11/Vps39/Vps18/Vav3/Vamp3/Vamp7/Snapin/Snap25/Vti1a |
| GO:0046033 | AMP metabolic process | 15 | -0.697 | -1.743 | 0.005 | Ampd2/Adsl/Ak1/Adss/Nudt2/Adssl1/Ak2/Ada/Ampd3/Ak4 |
| GO:0001676 | long-chain fatty acid metabolic process | 47 | -0.508 | -1.696 | 0.005 | Lipe/Gpx4/Acad9/Acsbg1/Acsl3/Ephx1/Acadl/Acot1/Acsl6/Acot2/Cyp2s1/Acsl1/Cpt1a/Cd36/Acsl5/Acot7/Abcd1 |
| GO:0007517 | muscle organ development | 94 | 0.451 | 1.581 | 0.005 | Uqcc2/Naca/Prkaa1/Vangl2/Chat/Ryr2/Myorg/Snw1/Fzd2/Ctnnb1/Ly6e/Bcl2/Erbb3/Rps6kb1/Homer1/Mapk14/Ttn/Crhr2/Fkbp1a/Cntfr/Nf1/Luc7l/Sgcb |
| GO:0033866 | nucleoside bisphosphate biosynthetic process | 31 | -0.554 | -1.622 | 0.005 | Coasy/Acsl6/Acat1/Acsl1/Acly/Acss1/Acsl5/Acot7/Mpc1 |
| GO:0034030 | ribonucleoside bisphosphate biosynthetic process | 31 | -0.554 | -1.622 | 0.005 | Coasy/Acsl6/Acat1/Acsl1/Acly/Acss1/Acsl5/Acot7/Mpc1 |
| GO:0034033 | purine nucleoside bisphosphate biosynthetic process | 31 | -0.554 | -1.622 | 0.005 | Coasy/Acsl6/Acat1/Acsl1/Acly/Acss1/Acsl5/Acot7/Mpc1 |
| GO:0010592 | positive regulation of lamellipodium assembly | 21 | -0.637 | -1.721 | 0.005 | Aqp1/Cyfip1/Cfl1/Arpc2/Wasf2/Twf2/Abi2 |
| GO:0032365 | intracellular lipid transport | 22 | -0.597 | -1.628 | 0.005 | Abcd4/Abcd3/Vps4a/Abcd2/Abca1/Abcd1 |
| GO:0032535 | regulation of cellular component size | 270 | -0.321 | -1.391 | 0.005 | Pycard/Gmfb/Gba2/Snx9/Akt3/Brk1/Pfn2/Rhoa/Mtpn/Arhgap35/Capn1/Ctnna2/Akt1/Ap1ar/Wdr1/Evl/Coro1b/Myadm/Cntn2/Spta1/Pex11b/Ntrk3/Wasf3/Bin1/Npm1/Aqp1/Bbs4/Coro1a/Chmp3/Cttn/Dstn/Twf1/Capza1/Myo1c/Cln8/Arpc1b/Plek/Carmil1/Cyfip1/Akt1s1/Arf1/Cfl1/Wasl/Fgf13/Anxa7/Arpc1a/Add2/Anapc2/Washc1/Arfgef1/Bag4/Arpc2/Xk/Atp2b2/Wasf2/Aatk/Hp1bp3/Ulk1/Twf2/Wasf1/Abi2/Ulk2/Wdtc1 |
| GO:0051125 | regulation of actin nucleation | 22 | -0.594 | -1.619 | 0.005 | Gmfb/Brk1/Ctnna2/Ap1ar/Coro1b/Wasf3/Coro1a/Cyfip1/Arf1/Wasl/Wasf2/Wasf1/Abi2 |
| GO:0016567 | protein ubiquitination | 250 | 0.364 | 1.434 | 0.006 | Ube2j1/Ubr3/Ube2k/Zswim2/Vcpip1/Ube2j2/Vps11/Vps28/Cdc23/Vps18/Ube2v2/Ube2d1/Ube2l3/Klhl9/Atg3/Pja1/Septin4/Usp4/Vcp/Cul5/Fgfr3/Nub1/Rpl23/Ube2a/Ube2n/Isg15/Rnf167/Ltn1/Cul9/Sharpin/Ctnnb1/Hace1/Wwp1/Uba1/Bcl2/Vhl/Tbc1d7/Prpf19/Wdr48/Anapc13/Ubr2/Smurf2/Hecw1/Traf3/Ufl1/Marchf6/Mgrn1/Fbxo7/Ube2g2/Gclc/Fkbp1a/Ubr1/Herc4/Arrdc3/Commd1/Atg7/Aktip/Pten/Nedd4/Os9/Rc3h1/Arih2 |
| GO:0042304 | regulation of fatty acid biosynthetic process | 19 | -0.659 | -1.732 | 0.006 | Acadl/Apoa4/Abcd2/Acadvl/Abcd1/Wdtc1 |
| GO:0060537 | muscle tissue development | 150 | 0.414 | 1.540 | 0.006 | Uqcc2/Naca/Prkaa1/Poglut1/Ryr2/Ndufv2/Cdc42/Frs2/Sgcz/Efnb2/Myorg/Snw1/Zfand5/Cacybp/Zmpste24/Ncam1/Ctnnb1/Ly6e/Bcl2/Itgb1/Erbb3/Rps6kb1/Homer1/Cby1/Mapk14/Ttn/Crhr2/Fkbp1a/G6pdx/Atg7/Csrp2/Pten/Nf1/Prkg1/Luc7l/Sgcb/Csrp1 |
| GO:0015909 | long-chain fatty acid transport | 30 | -0.561 | -1.632 | 0.006 | Abcd4/Acsl3/Mif/Acsl6/Acsl1/Abcd3/Cd36/Abcd2/Acsl5/Abcd1 |
| GO:0050867 | positive regulation of cell activation | 115 | 0.435 | 1.564 | 0.006 | Vamp8/Adk/Slc39a10/Bloc1s3/Vcam1/Vav3/Hspd1/Cd276/Vamp7/Efnb2/Igkc/Hes1/Fgr/Pcid2/Bcl2/Atp11c/Dpp4/Gas6/Itgam/Mdk/Gpam/Dgat1/Prkcz/Ighm/Thy1/Efnb3/Pla2g4a |
| GO:0032271 | regulation of protein polymerization | 162 | -0.358 | -1.457 | 0.006 | Sptbn1/Ptk2b/Mapre1/Pde4dip/Eml2/Clasp2/Tppp3/Arpc3/Capg/Capza2/Arl2/Prkcd/Mapk8/Grb2/Arpc5/Map2/Carmil2/Prune1/Arfip1/Abl1/Nck2/Pik3r2/Fkbp4/Ttbk1/Pycard/Gmfb/Gba2/Snx9/Brk1/Pfn2/Dyrk1a/Rhoa/Cav1/Mtpn/Capn1/Ctnna2/Ap1ar/Evl/Coro1b/Apc/Myadm/Spta1/Wasf3/Bin1/Bbs4/Coro1a/Cttn/Twf1/Capza1/Myo1c/Arpc1b/Carmil1/Cyfip1/Fes/Drg1/Arf1/Cfl1/Wasl/Arpc1a/Add2/Washc1/Arfgef1/Bag4/Arpc2/Wasf2/Twf2/Wasf1/Abi2/Vdac2 |
| GO:0032370 | positive regulation of lipid transport | 57 | -0.477 | -1.657 | 0.006 | Dbi/Mif/Acsl6/Dennd5b/Atp8a2/Acsl1/Washc1/Abca7/Acsl5/Abca3/Abca1/Abat |
| GO:1905954 | positive regulation of lipid localization | 66 | -0.448 | -1.597 | 0.006 | Dbi/C3/Mif/Acsl6/Dennd5b/Atp8a2/Acsl1/Cd36/Washc1/Abca7/Acsl5/Abca3/Abca1/Abat |
| GO:0044089 | positive regulation of cellular component biogenesis | 335 | -0.304 | -1.355 | 0.006 | Pycard/Gmfb/Tgfbr1/Cck/Snx9/Pip4k2b/Brk1/Pfn2/Rhoa/Dnm3/Cav1/Arhgap35/Iqgap1/Ctnna2/Ptprd/Ap1ar/Rock2/Dynll1/Tfrc/Evl/Flot1/Agt/Bak1/Coro1b/Apc/Adgrb3/Flrt3/Map4k4/Ntrk3/Wasf3/Nlgn3/Bin1/Piezo1/Lrrtm3/Arhgef10l/Bid/Amigo2/Adgrb2/Atat1/Aqp1/Bbs4/G3bp2/Coro1a/Cttn/Phldb1/Psmc3/Myo1c/Adgrb1/Arpc1b/Lrtm2/Plek/Cep135/Caly/Carmil1/Vps4b/Cyfip1/Fes/Psmc2/Drg1/Arf1/Cfl1/Slitrk1/Wasl/Chga/Arpc1a/Asic2/Wars/Sdc4/Dag1/Cd36/Msn/Vps4a/Lingo2/Washc1/Bag4/Arpc2/Chmp2a/Adgrl1/Abca3/Abca1/Wasf2/Cbln2/Ulk1/Twf2/Wasf1/Abi2 |
| GO:0019217 | regulation of fatty acid metabolic process | 44 | -0.510 | -1.680 | 0.006 | Acadl/Cnr1/Dbi/Cpt1a/Apoa4/Abcd2/Appl2/Acsl5/Acadvl/Abcd1/Wdtc1 |
| GO:0062125 | regulation of mitochondrial gene expression | 18 | 0.691 | 1.753 | 0.006 | Uqcc2/Prkaa1/Mief1/Rpusd3 |
| GO:0045732 | positive regulation of protein catabolic process | 126 | 0.434 | 1.577 | 0.007 | Uqcc2/Rhbdd3/Vps11/Vps28/Gpc3/Ube2v2/Vcp/Agbl4/Nub1/Tmem67/Apc2/Vps35/Hace1/Wwp1/App/Atpif1/Smurf2/Hecw1/Lrp1/Atxn3/Ddrgk1/Gclc/Csnk1d/Atg7/Gpld1/Pten/Nedd4/Abca2/Arih2/Ezr |
| GO:0051153 | regulation of striated muscle cell differentiation | 28 | 0.593 | 1.639 | 0.007 | Naca/Frs2/Ybx1/Efnb2/Bcl2/Mapk14/G6pdx |
| GO:0072521 | purine-containing compound metabolic process | 237 | -0.317 | -1.362 | 0.007 | Cacnb4/Insr/Pdk1/Atp5h/Adpgk/Mtch2/Sphk2/Acaca/Nt5c1a/Ak1/Adcy8/Nudt10/Mvd/Acadsb/Uqcc3/Mccc2/Acsl3/Macrod2/Hk2/Adss/Far1/Coasy/Atp5j2/Adcy5/Icmt/Pfas/Nudt2/Gapdh/Acot1/Aldoc/Adssl1/Mif/Acsl6/Acot6/Acot2/Acat1/Acsl1/Acly/Acot8/Itpa/Ak2/Ada/Adcy10/Acss1/Acsl5/Acot7/Hsd17b4/Ampd3/Atp2b2/Mpc1/Acot9/Abcd1/Ak4/Adcy6 |
| GO:0019395 | fatty acid oxidation | 68 | -0.452 | -1.616 | 0.007 | Abcd4/Acadm/Etfb/Acadl/Cnr1/Dbi/Acat1/Cpt1a/Abcd3/Cd36/Acaa1a/Abcd2/Pex13/Acad11/Appl2/Adipor2/Acox1/Acsl5/Acadvl/Hsd17b4/Abcd1 |
| GO:0050891 | multicellular organismal water homeostasis | 20 | -0.627 | -1.682 | 0.007 | Cdh1/Aqp1/Krt1/Ugcg/Stard7/Wfs1/Adcy6 |
| GO:0009260 | ribonucleotide biosynthetic process | 99 | -0.398 | -1.528 | 0.007 | Adcy9/Gucy1b1/Paics/Flcn/Pdk2/Ampd2/Adsl/Adcy7/Atic/Atp5c1/Pdk1/Atp5h/Sphk2/Ak1/Adcy8/Uqcc3/Adss/Coasy/Atp5j2/Adcy5/Pfas/Nudt2/Adssl1/Acsl6/Acat1/Acsl1/Acly/Ak2/Adcy10/Cmpk1/Acss1/Acsl5/Acot7/Ampd3/Mpc1/Ak4/Adcy6 |
| GO:0045933 | positive regulation of muscle contraction | 12 | -0.731 | -1.743 | 0.007 | Chrm3/Rhoa/Cttn/Chga/Atp1a1/Adra1a/Ada/Abat |
| GO:0032418 | lysosome localization | 51 | 0.528 | 1.644 | 0.008 | Vamp8/Myh9/Vps33a/Vps33b/Vamp7/Snapin/Fgr/Borcs5/Bloc1s2 |
| GO:0045010 | actin nucleation | 38 | -0.517 | -1.599 | 0.008 | Gmfb/Brk1/Ctnna2/Ap1ar/Evl/Coro1b/Wasf3/Coro1a/Arpc1b/Cyfip1/Arf1/Wasl/Arpc1a/Washc1/Arpc2/Wasf2/Wasf1/Abi2 |
| GO:0090066 | regulation of anatomical structure size | 345 | -0.298 | -1.327 | 0.008 | Dbh/Pycard/Chrm3/Gmfb/Gba2/Snx9/Akt3/Brk1/Pfn2/Rhoa/Cav1/Mtpn/Arhgap35/Fgb/Capn1/Ctnna2/Akt1/Ap1ar/Rock2/Wdr1/Evl/Agt/Coro1b/Myadm/Cntn2/Spta1/Pex11b/Ntrk3/Wasf3/Bin1/Npm1/Aqp1/Bbs4/Coro1a/Chmp3/Atg5/Egfr/Cacna1c/Cttn/Dstn/Hrh1/Twf1/Capza1/Atp1a2/Myo1c/Cln8/Arpc1b/Plek/Carmil1/Cyfip1/Akt1s1/Arf1/Cfl1/Acta2/Wasl/Fgf13/Anxa7/Arpc1a/Asic2/Atp2b1/Add2/Adra1a/Anapc2/Washc1/Arfgef1/Bag4/Arpc2/Xk/Atp2b2/Wasf2/Aatk/Hp1bp3/Cacna1g/Ulk1/Twf2/Wasf1/Abi2/Ulk2/Adcy6/Wdtc1 |
| GO:0033005 | positive regulation of mast cell activation | 12 | 0.753 | 1.717 | 0.008 | Vamp8/Vamp7/Fgr |
| GO:0140115 | export across plasma membrane | 40 | -0.494 | -1.555 | 0.009 | Agrn/Abcc1/Abcc4/Kcnb1/Slc36a2/Slc8a1/Abcg2/Atp1b3/Atp1a3/Atp1a4/Atp1a2/Ywhae/Atp2b1/Atp1a1/Atp2b3/Abcb1a/Abca1/Atp12a |
| GO:1902186 | regulation of viral release from host cell | 18 | -0.627 | -1.625 | 0.009 | Ddb1/Chmp3/Vapb/Vps4b/Vps4a/Chmp2a/Vps37b/Vapa |
| GO:0045844 | positive regulation of striated muscle tissue development | 10 | 0.758 | 1.635 | 0.009 | Naca/Prkaa1/Ctnnb1/Bcl2/Erbb3/Rps6kb1 |
| GO:0048636 | positive regulation of muscle organ development | 10 | 0.758 | 1.635 | 0.009 | Naca/Prkaa1/Ctnnb1/Bcl2/Erbb3/Rps6kb1 |
| GO:1901863 | positive regulation of muscle tissue development | 10 | 0.758 | 1.635 | 0.009 | Naca/Prkaa1/Ctnnb1/Bcl2/Erbb3/Rps6kb1 |
| GO:0042180 | cellular ketone metabolic process | 99 | -0.393 | -1.511 | 0.009 | Stard3/Aldoa/Coq7/Kyat3/Gk/Scap/Acsl4/Eif2ak3/Coq4/Fabp3/Haghl/Glo1/Pdk2/Ncor2/Coq5/Cav1/Ndufa9/Akt1/Agt/Atcay/Apc/Fdxr/Pdk1/Coq8a/Cacna1h/Pnkd/Comt/Dkk3/Acadl/Cnr1/Dbi/H6pd/Akr1a1/Cpt1a/Apoa4/Abcd2/Aifm2/Appl2/Acsl5/Acadvl/Abcd1/Wdtc1 |
| GO:1902745 | positive regulation of lamellipodium organization | 26 | -0.569 | -1.604 | 0.009 | Brk1/Coro1b/Aqp1/Carmil1/Cyfip1/Cfl1/Arpc2/Wasf2/Twf2/Abi2 |
| GO:0051223 | regulation of protein transport | 331 | 0.343 | 1.385 | 0.009 | Vamp8/Uqcc2/Ube2j1/Svip/Ran/Vamp2/Rhbdd3/Prkaa1/Vps28/Ttc21b/Glrx/Hpca/Rptor/Gcc2/Brsk2/Ahi1/Nup62/Cdc42/Vamp7/Cacna1e/Pard6a/Usp46/Efnb2/Pam/Glul/Nrxn1/Vps35/Ptpn23/Ripor1/Tmed10/Ubac2/Pick1/Flna/Nlgn2/Doc2b/App/Hspa1l/Oxct1/Atpif1/Arfip1/Hap1/Camk1/Nadk/Sirt3/Ptpn11/Gas6/Hadh/Itgam/C2cd5/Lrp1/Jagn1/Mapk14/Ptpn1/Arf6/Prkcz/Ttn/Ube2g2/Crhr2/Erp29/Commd1/Ipo5/Atg7/Pkia/Gpld1/Nedd4/Os9/Abcg1/Nf1 |
| GO:0014706 | striated muscle tissue development | 142 | 0.411 | 1.510 | 0.009 | Uqcc2/Naca/Prkaa1/Ryr2/Ndufv2/Cdc42/Frs2/Sgcz/Efnb2/Myorg/Snw1/Cacybp/Zmpste24/Ncam1/Ctnnb1/Ly6e/Bcl2/Itgb1/Erbb3/Rps6kb1/Homer1/Cby1/Mapk14/Ttn/Crhr2/Fkbp1a/G6pdx/Atg7/Pten/Nf1/Prkg1/Luc7l/Sgcb |
| GO:0034314 | Arp2/3 complex-mediated actin nucleation | 31 | -0.532 | -1.559 | 0.010 | Gmfb/Brk1/Ctnna2/Ap1ar/Coro1b/Wasf3/Arpc1b/Cyfip1/Arf1/Wasl/Arpc1a/Washc1/Arpc2/Wasf2/Wasf1/Abi2 |
| GO:0051127 | positive regulation of actin nucleation | 10 | -0.735 | -1.664 | 0.010 | Brk1/Wasf3/Cyfip1/Wasl/Wasf2/Wasf1/Abi2 |
| GO:0009154 | purine ribonucleotide catabolic process | 27 | -0.562 | -1.602 | 0.010 | Acat1/Itpa/Ada/Acot7/Ampd3/Abcd1 |
| GO:0034315 | regulation of Arp2/3 complex-mediated actin nucleation | 18 | -0.622 | -1.610 | 0.010 | Gmfb/Brk1/Ctnna2/Ap1ar/Coro1b/Wasf3/Cyfip1/Arf1/Wasl/Wasf2/Wasf1/Abi2 |
| GO:0035384 | thioester biosynthetic process | 23 | -0.601 | -1.665 | 0.010 | Acsl6/Acat1/Acsl1/Acly/Acss1/Acsl5/Mpc1 |
| GO:0071616 | acyl-CoA biosynthetic process | 23 | -0.601 | -1.665 | 0.010 | Acsl6/Acat1/Acsl1/Acly/Acss1/Acsl5/Mpc1 |
| GO:0031000 | response to caffeine | 10 | 0.750 | 1.617 | 0.010 | Prkaa1/Tmem38b/Ryr2/Gnal |
| GO:1901568 | fatty acid derivative metabolic process | 24 | -0.593 | -1.671 | 0.010 | Acsl3/Far1/Acsl6/Abhd16a/Acat1/Acsl1/Acsl5/Acot7/Hsd17b4/Abcd1 |
| GO:0008333 | endosome to lysosome transport | 37 | 0.531 | 1.567 | 0.011 | Vps11/Vps33a/Vps16/Vps39/Vps18/Hook3/Vamp7/Vcp/Ubxn6/Snapin |
| GO:0090087 | regulation of peptide transport | 346 | 0.333 | 1.354 | 0.011 | Vamp8/Uqcc2/Ube2j1/Svip/Ran/Vamp2/Rhbdd3/Prkaa1/Vps28/Ttc21b/Glrx/Hpca/Rptor/Gcc2/Brsk2/Ahi1/Nup62/Cdc42/Vamp7/Cacna1e/Pard6a/Usp46/Efnb2/Pam/Glul/Nrxn1/Vps35/Ptpn23/Ripor1/Tmed10/Ubac2/Pick1/Flna/Nlgn2/Doc2b/App/Hspa1l/Oxct1/Atpif1/Arfip1/Hap1/Camk1/Nadk/Sirt3/Ptpn11/Gas6/Hadh/Itgam/C2cd5/Lrp1/Jagn1/Mapk14/Ptpn1/Arf6/Prkcz/Ttn/Ube2g2/Crhr2/Erp29/Commd1/Ipo5/Atg7/Pkia/Gpld1/Nedd4/Os9/Abcg1/Nf1/Ezr/Itsn1/Snap91/Tiam1 |
| GO:0030837 | negative regulation of actin filament polymerization | 45 | -0.494 | -1.633 | 0.011 | Pfn2/Mtpn/Capn1/Evl/Myadm/Spta1/Bbs4/Twf1/Capza1/Carmil1/Cfl1/Add2/Arfgef1/Arpc2/Twf2 |
| GO:0046755 | viral budding | 15 | -0.668 | -1.671 | 0.011 | Chmp3/Chmp5/Vps4b/Vps4a/Chmp2a/Chmp6/Vps37b/Vta1 |
| GO:0006883 | cellular sodium ion homeostasis | 16 | -0.623 | -1.583 | 0.011 | Slc8a1/Slc1a3/Atp1b3/Atp1a3/Agt/Atp1a4/Atp1a2/Atp1a1/Atp12a |
| GO:0035272 | exocrine system development | 16 | -0.622 | -1.581 | 0.011 | Cdh1/Insr/Egfr/Plxna1/Dag1/Wls |
| GO:1902041 | regulation of extrinsic apoptotic signaling pathway via death domain receptors | 23 | 0.641 | 1.706 | 0.011 | Zswim2/Faim/Bmpr1b/Faim2/Bcl2l1/Pten/Raf1 |
| GO:0045727 | positive regulation of translation | 72 | 0.457 | 1.519 | 0.011 | Uqcc2/Ythdf1/Slc35a4/Mief1/Eif3e |
| GO:0031929 | TOR signaling | 73 | 0.460 | 1.541 | 0.012 | Wdr59/Eif4ebp2/Prkaa1/Rptor/Alg13/Lars/Zmpste24/Mios/Tiprl/Tbc1d7/Gas6/Rps6kb1/Ubr2/Slc7a3/Ubr1/Dgkq |
| GO:0009127 | purine nucleoside monophosphate biosynthetic process | 16 | -0.620 | -1.576 | 0.012 | Impdh2/Aprt/Ppat/Paics/Ampd2/Adsl/Atic/Adss/Pfas/Nudt2/Adssl1/Ada/Ampd3 |
| GO:0009168 | purine ribonucleoside monophosphate biosynthetic process | 16 | -0.620 | -1.576 | 0.012 | Impdh2/Aprt/Ppat/Paics/Ampd2/Adsl/Atic/Adss/Pfas/Nudt2/Adssl1/Ada/Ampd3 |
| GO:0035914 | skeletal muscle cell differentiation | 15 | 0.711 | 1.720 | 0.012 | Uqcc2/Snw1 |
| GO:0051289 | protein homotetramerization | 28 | -0.547 | -1.569 | 0.012 | Hsd17b10/Crtc1/Shmt2/Trpm2/Osbpl2/B2m/Pkd2/Evl/Apip/Acaca/Aldh9a1/Acot13/Aldh1a2/Cth/Appl2/Cryz |
| GO:0032527 | protein exit from endoplasmic reticulum | 34 | 0.538 | 1.560 | 0.012 | Ube2j1/Svip/Aup1/Gcc2/Vcp/Ubac2/H13/Ufd1 |
| GO:0009117 | nucleotide metabolic process | 267 | -0.305 | -1.322 | 0.012 | Cacnb4/Insr/Pdk1/Atp5h/Adpgk/Mtch2/Dera/Dtymk/Sphk2/Acaca/Nt5c1a/Ak1/Adcy8/Nudt10/Mvd/Enpp5/Acadsb/Uqcc3/Mccc2/Acsl3/Hk2/Flad1/Adss/Far1/Coasy/Atp5j2/Entpd4/Adcy5/Pfas/Nudt2/Gapdh/Acot1/Aldoc/Adssl1/Mif/Acsl6/Acot6/Acot2/Acat1/Acsl1/Acly/Acot8/Itpa/Ak2/Ada/Adcy10/Cmpk1/Acss1/Acsl5/Acot7/Hsd17b4/Ampd3/Atp2b2/Mpc1/Acot9/Abcd1/Ak4/Adcy6 |
| GO:1902001 | fatty acid transmembrane transport | 30 | -0.532 | -1.547 | 0.012 | Septin2/Slc1a3/Arl6ip1/Akt1/Slc25a18/Abcd4/Cln8/Acsl6/Acsl1/Abcd3/Cd36/Abcd2/Acsl5/Abcd1 |
| GO:0042178 | xenobiotic catabolic process | 10 | -0.728 | -1.647 | 0.012 | Acsl1/Acaa1a/Cryz |
| GO:0048670 | regulation of collateral sprouting | 14 | -0.668 | -1.651 | 0.012 | Fgf13/Ulk1/Ulk2 |
| GO:0140053 | mitochondrial gene expression | 79 | 0.460 | 1.569 | 0.013 | Uqcc2/Mrps18b/Sars2/Prkaa1/Mief1/Mterf2/Mrpl57/Mrpl43/Mtrf1l/Tfb1m/Rpusd3/Rars2 |
| GO:0098727 | maintenance of cell number | 36 | 0.532 | 1.558 | 0.013 | Eif4e/Vangl2/Ddx6/Hook3/Fgfr3/Hes1/Ctnnb1/Crebbp |
| GO:0070201 | regulation of establishment of protein localization | 350 | 0.334 | 1.354 | 0.013 | Vamp8/Uqcc2/Ube2j1/Svip/Ran/Vamp2/Rhbdd3/Prkaa1/Vps28/Ttc21b/Glrx/Hpca/Rptor/Gcc2/Brsk2/Ahi1/Nup62/Cdc42/Vamp7/Cacna1e/Pard6a/Usp46/Efnb2/Pam/Glul/Nrxn1/Vps35/Ptpn23/Snap25/Ripor1/Tmed10/Ubac2/Pick1/Flna/Nlgn2/Doc2b/App/Hspa1l/Oxct1/Atpif1/Arfip1/Hap1/Camk1/Nadk/Sirt3/Ptpn11/Gas6/Hadh/Itgam/C2cd5/Lrp1/Jagn1/Mapk14/Ptpn1/Arf6/Prkcz/Ttn/Ube2g2/Crhr2/Cep295/Erp29/Commd1/Ipo5/Atg7/Pkia/Gpld1/Nedd4/Os9/Abcg1/Nf1 |
| GO:0051216 | cartilage development | 54 | 0.503 | 1.586 | 0.013 | Bbs2/Bmpr1b/Cbs/Fgfr3/Bbs1/Csgalnact1/Zmpste24/Ctnnb1/Mboat2/Ptpn11/Mdk/Mapk14/Ddrgk1/Efemp1/Itgb8/Gpld1 |
| GO:0006641 | triglyceride metabolic process | 52 | -0.476 | -1.614 | 0.013 | Gykl1/Apobr/Dbi/C3/Acsl6/Acsl1/Cpt1a/Apoa4/Cd36/Apoh/Acsl5/Abhd5/Atg14 |
| GO:0019827 | stem cell population maintenance | 35 | 0.547 | 1.595 | 0.014 | Eif4e/Vangl2/Ddx6/Hook3/Fgfr3/Hes1/Ctnnb1/Crebbp |
| GO:0046390 | ribose phosphate biosynthetic process | 105 | -0.385 | -1.495 | 0.014 | Adcy9/Gucy1b1/Paics/Flcn/Pdk2/Ampd2/Adsl/Adcy7/Atic/Atp5c1/Pdk1/Atp5h/Sphk2/Ak1/Adcy8/Uqcc3/Adss/Coasy/Atp5j2/Adcy5/Pfas/Nudt2/Adssl1/Acsl6/Acat1/Acsl1/Acly/Ak2/Adcy10/Cmpk1/Acss1/Acsl5/Acot7/Ampd3/Mpc1/Ak4/Adcy6 |
| GO:0006753 | nucleoside phosphate metabolic process | 274 | -0.303 | -1.314 | 0.014 | Cacnb4/Insr/Pdk1/Atp5h/Adpgk/Mtch2/Dera/Dtymk/Sphk2/Acaca/Nt5c1a/Ak1/Adcy8/Nudt10/Mvd/Enpp5/Acadsb/Uqcc3/Mccc2/Acsl3/Hk2/Flad1/Adss/Far1/Coasy/Atp5j2/Entpd4/Adcy5/Pfas/Nudt2/Gapdh/Acot1/Entpd3/Aldoc/Adssl1/Mif/Acsl6/Acot6/Acot2/Acat1/Acsl1/Acly/Acot8/Itpa/Ak2/Ada/Adcy10/Cmpk1/Acss1/Acsl5/Acot7/Hsd17b4/Ampd3/Atp2b2/Mpc1/Acot9/Abcd1/Ak4/Adcy6 |
| GO:0046320 | regulation of fatty acid oxidation | 19 | -0.628 | -1.651 | 0.014 | Acadl/Cnr1/Dbi/Cpt1a/Abcd2/Appl2/Acsl5/Acadvl/Abcd1 |
| GO:0050729 | positive regulation of inflammatory response | 47 | 0.508 | 1.551 | 0.014 | Vamp8/Fcgr1/Hspd1/Vamp7 |
| GO:0033008 | positive regulation of mast cell activation involved in immune response | 11 | 0.761 | 1.697 | 0.014 | Vamp8/Vamp7/Fgr |
| GO:0043306 | positive regulation of mast cell degranulation | 11 | 0.761 | 1.697 | 0.014 | Vamp8/Vamp7/Fgr |
| GO:0110053 | regulation of actin filament organization | 190 | -0.328 | -1.371 | 0.014 | Pycard/Gmfb/Tgfbr1/Gba2/Snx9/Brk1/Pfn2/Rhoa/Mtpn/Arhgap35/Capn1/Ctnna2/Ap1ar/Rock2/Wdr1/Evl/Coro1b/Myadm/Spta1/Wasf3/Bin1/Arhgef10l/Bbs4/Coro1a/Ppfia1/Cttn/Dstn/Twf1/Capza1/Myo1c/Arpc1b/Plek/Carmil1/Cyfip1/Arhgef18/Arf1/Cfl1/Wasl/Arpc1a/Sdc4/Add2/Washc1/Arfgef1/Bag4/Arpc2/Wasf2/Twf2/Wasf1/Abi2 |
| GO:0050708 | regulation of protein secretion | 152 | 0.387 | 1.440 | 0.014 | Vamp8/Uqcc2/Rhbdd3/Glrx/Rptor/Brsk2/Ahi1/Cacna1e/Pard6a/Pam/Glul/Nrxn1/Vps35/Ptpn23/Tmed10/Pick1/Nlgn2/Doc2b/Oxct1/Arfip1/Nadk/Sirt3/Ptpn11/Hadh/Lrp1/Jagn1/Arf6/Ttn/Crhr2/Erp29/Atg7/Gpld1/Abcg1/Ezr/Tiam1 |
| GO:0051603 | proteolysis involved in cellular protein catabolic process | 409 | 0.323 | 1.333 | 0.015 | Vps37a/Ube2j1/Ubr3/Ube2k/Usp9x/Svip/Aup1/Yme1l1/Ube2j2/Vps28/Usp8/Uba7/Ube2i/Cdc23/Psmd1/Ubap1/Ube2v2/Ube2d1/Ube2l3/Brsk2/Usp4/Usp47/Amn1/Vcp/Agbl4/Cul5/Nub1/Usp46/Ubxn6/Uchl1/Rpl23/Tmem67/Usp30/Ube2a/Ube2n/Isg15/Rnf167/Ltn1/Ptpn23/Cul9/Zmpste24/Sharpin/Dcaf11/Ctnnb1/Ubac2/Hace1/Ctsf/Eif3h/Ddi2/H13/Wwp1/Ufd1/Uba1/Vps37c/Pdcd6ip/Vhl/Tollip/Atpif1/Ccar2/Prpf19/Clpx/Usp24/Dnajb12/Vps25/Zfand2b/Map1a/Ubr2/Smurf2/Hecw1/Ufl1/Psmd2/Pomt2/Atxn3/Ddrgk1/Marchf6/Usp7/Get4/Fbxo7/Ube2g2/Gclc/Ubr1/Csnk1d/Erlin1/Commd1/Kctd6/Atg7/Pten/Nedd4/Os9/Chmp4b/Rc3h1/Arih2 |
| GO:0046823 | negative regulation of nucleocytoplasmic transport | 15 | -0.659 | -1.648 | 0.015 | Akap1/Apod/Ei24/Cd36/Ufm1 |
| GO:0007608 | sensory perception of smell | 13 | 0.710 | 1.648 | 0.015 | Ubr3/Gnal/Bbs1/Fzd2/Adcy3/Ttc8/Dmxl2 |
| GO:0006869 | lipid transport | 221 | -0.311 | -1.328 | 0.015 | Osbpl10/Esyt2/Cln8/Apod/Ttpa/Ugcg/Vps4b/Apobr/Tspo/Dbi/Atp9a/Mif/Acsl6/Dennd5b/Abca9/Osbpl1a/Atp8a2/Acsl1/Osbpl9/Abcd3/Apoa4/Cd36/Xkr7/Ttc39b/Vps4a/Apoh/Abcb1a/Abcd2/Washc1/Abca7/Acsl5/Abca3/Abca1/Abat/Abcd1/Ano4 |
| GO:0019076 | viral release from host cell | 19 | -0.626 | -1.646 | 0.015 | Ddb1/Chmp3/Vapb/Vps4b/Vps4a/Chmp2a/Vps37b/Vapa |
| GO:0035890 | exit from host | 19 | -0.626 | -1.646 | 0.015 | Ddb1/Chmp3/Vapb/Vps4b/Vps4a/Chmp2a/Vps37b/Vapa |
| GO:0035891 | exit from host cell | 19 | -0.626 | -1.646 | 0.015 | Ddb1/Chmp3/Vapb/Vps4b/Vps4a/Chmp2a/Vps37b/Vapa |
| GO:0090114 | COPII-coated vesicle budding | 18 | -0.603 | -1.561 | 0.015 | Sec24c/Pef1/Vapb/Pdcd6/Sar1a/Vapa |
| GO:0006164 | purine nucleotide biosynthetic process | 103 | -0.384 | -1.482 | 0.016 | Oas1a/Dcakd/Aldoa/Impdh2/Aprt/Adcy1/Atp5j/Ppat/Dld/Pnp/Acsl4/Adcy9/Gucy1b1/Paics/Flcn/Pdk2/Ampd2/Adsl/Adcy7/Atic/Atp5c1/Pdk1/Atp5h/Sphk2/Ak1/Adcy8/Uqcc3/Adss/Coasy/Atp5j2/Adcy5/Pfas/Nudt2/Adssl1/Acsl6/Acat1/Acsl1/Acly/Ak2/Ada/Adcy10/Acss1/Acsl5/Acot7/Ampd3/Mpc1/Ak4/Adcy6 |
| GO:0030856 | regulation of epithelial cell differentiation | 34 | 0.530 | 1.535 | 0.016 | Vcl/Ahi1/Prom1/Hes1/Ctnnb1/Spred2/Vhl/F11r |
| GO:0006677 | glycosylceramide metabolic process | 14 | -0.654 | -1.616 | 0.016 | B4galt3/Prkcd/B3galt2/B3galt1/Gba2/Gla/Ugcg/Naga/Gal3st1 |
| GO:0022412 | cellular process involved in reproduction in multicellular organism | 101 | 0.414 | 1.464 | 0.016 | Bbs2/Ube2j1/Ythdc2/Pacrg/Tdrkh/Bmpr1b/Tdrd7/Septin4/Prdx4/Ctnnb1/Folr1/Pafah1b1/Bcl2/Bcl2l1/Itgb1/Fkbp6/Rps6kb1/Ubr2/Mdk |
| GO:0062014 | negative regulation of small molecule metabolic process | 35 | -0.504 | -1.525 | 0.016 | Flcn/Fam3c/Fam3a/Akt1/Atcay/Mtch2/Dkk3/Plek/Acadl/Cnr1/Dbi/Appl2/Acadvl/Wdtc1 |
| GO:0090207 | regulation of triglyceride metabolic process | 24 | -0.565 | -1.594 | 0.016 | C3/Apoa4/Cd36/Apoh/Acsl5/Abhd5/Atg14 |
| GO:1905515 | non-motile cilium assembly | 35 | 0.537 | 1.566 | 0.016 | Bbs2/Vangl2/Ttbk2/Ahi1/Bbs1/Tmem67/Htt/Hap1/Ttc8/Rpgrip1l/Cc2d2a/Csnk1d |
| GO:1902905 | positive regulation of supramolecular fiber organization | 142 | -0.354 | -1.422 | 0.016 | Tac1/Arl2/Mapk8/Grb2/Arpc5/Psen1/Carmil2/Arfip1/Abl1/Nck2/Colgalt1/Htr1a/Arhgef10/Pycard/Gmfb/Tgfbr1/Snx9/Brk1/Pfn2/Rhoa/Cav1/Ctnna2/Ap1ar/Rock2/Wdr1/Evl/Coro1b/Apc/Wasf3/Bin1/Arhgef10l/Katnb1/Coro1a/Cttn/Dstn/Myo1c/Arpc1b/Plek/Carmil1/Cyfip1/Fes/Drg1/Arf1/Cfl1/Wasl/Arpc1a/Sdc4/Washc1/Bag4/Arpc2/Wasf2/Wasf1/Abi2 |
| GO:1903827 | regulation of cellular protein localization | 369 | 0.330 | 1.347 | 0.016 | Vamp8/Ube2j1/Rhog/Vcpip1/Svip/Dbn1/Ran/Vamp2/Prkaa1/Vps28/Gpc3/Ttc21b/Hpca/Ttbk2/Gcc2/Nup62/Cdc42/Zdhhc5/Vamp7/Vcp/Cltc/Pard6a/Usp46/Efnb2/Grip2/Glul/Nrxn1/Dpp6/Vps35/Ripor1/Ctnnb1/Ubac2/Pick1/Ankrd13a/Gpc5/Flna/Htt/Nlgn2/Limk2/App/Hspa1l/Vhl/Atpif1/Itga3/Hap1/Rhoq/Camk1/Bcl2l1/Itgb1/Rer1/Myo5a/Cnpy4/Ptpn11/Gas6/Map1a/Itgam/C2cd5/Synj2bp/Lrp1/Mapk14/Ptpn1/Arf6/Prkcz/Gpd1l/Ddrgk1/Epb41l2 |
| GO:0008217 | regulation of blood pressure | 84 | -0.391 | -1.445 | 0.016 | Bbs4/Cacna1c/Ddah1/Atp1a2/Cnr1/Acta2/Mif/Asic2/Atp2b1/Atp1a1/Cd36/Adra1a/Wnk1/Col1a2/Npy/Trhde/Abat |
| GO:0072522 | purine-containing compound biosynthetic process | 106 | -0.368 | -1.431 | 0.017 | Oas1a/Dcakd/Aldoa/Impdh2/Shmt2/Aprt/Adcy1/Atp5j/Ppat/Dld/Pnp/Acsl4/Adcy9/Gucy1b1/Paics/Flcn/Pdk2/Ampd2/Adsl/Adcy7/Atic/Atp5c1/Pdk1/Atp5h/Sphk2/Ak1/Adcy8/Uqcc3/Adss/Coasy/Atp5j2/Adcy5/Pfas/Nudt2/Adssl1/Acsl6/Acat1/Acsl1/Acly/Ak2/Ada/Adcy10/Acss1/Acsl5/Acot7/Ampd3/Mpc1/Ak4/Adcy6 |
| GO:0006195 | purine nucleotide catabolic process | 29 | -0.531 | -1.534 | 0.017 | Nt5c1a/Nudt10/Acat1/Itpa/Ada/Acot7/Ampd3/Abcd1 |
| GO:0007519 | skeletal muscle tissue development | 55 | 0.487 | 1.540 | 0.018 | Uqcc2/Naca/Prkaa1/Myorg/Snw1/Ctnnb1/Bcl2/Rps6kb1/Homer1/Mapk14/Crhr2 |
| GO:1903409 | reactive oxygen species biosynthetic process | 66 | -0.421 | -1.499 | 0.018 | Gbf1/Pkd2/Rhoa/Cav1/Akt1/Rock2/Dynll1/Agt/Gla/Sirpa/Insr/Sphk2/Ddah1/Hrh1/Adgrb1/Tspo/Cd36/Abcd2/Aif1/Acox1/Abcd1 |
| GO:0043632 | modification-dependent macromolecule catabolic process | 349 | 0.322 | 1.307 | 0.018 | Vps37a/Ube2j1/Ubr3/Ube2k/Usp9x/Svip/Aup1/Ube2j2/Vps28/Usp8/Uba7/Ube2i/Cdc23/Psmd1/Ubap1/Ube2v2/Ube2d1/Ube2l3/Usp4/Usp47/Amn1/Vcp/Agbl4/Cul5/Nub1/Usp46/Uchl1/Rpl23/Tmem67/Usp30/Ube2a/Ube2n/Isg15/Rnf167/Ltn1/Ptpn23/Cul9/Zmpste24/Sharpin/Dcaf11/Ctnnb1/Hace1/Eif3h/Wwp1/Ufd1/Uba1/Vps37c/Tollip/Ccar2/Usp24/Dnajb12/Vps25/Zfand2b/Map1a/Ubr2/Smurf2/Hecw1/Ufl1/Psmd2/Atxn3/Ddrgk1/Marchf6/Usp7/Fbxo7/Ube2g2/Gclc/Ubr1/Csnk1d/Erlin1/Commd1/Kctd6/Atg7/Pten/Nedd4/Os9/Rc3h1/Arih2/Otud7b/Fbxl18 |
| GO:0072673 | lamellipodium morphogenesis | 13 | -0.654 | -1.585 | 0.018 | Snx1/Cd44/Myo9b/Coro1b/Wasf2/Abi1/Wasf1 |
| GO:0010565 | regulation of cellular ketone metabolic process | 64 | -0.434 | -1.544 | 0.018 | Cav1/Akt1/Agt/Atcay/Apc/Pdk1/Comt/Dkk3/Acadl/Cnr1/Dbi/H6pd/Cpt1a/Apoa4/Abcd2/Appl2/Acsl5/Acadvl/Abcd1/Wdtc1 |
| GO:0043001 | Golgi to plasma membrane protein transport | 32 | 0.548 | 1.569 | 0.018 | Bbs2/Vamp2/Vamp3/Golga4/Vamp7/Bbs1 |
| GO:0010876 | lipid localization | 248 | -0.301 | -1.293 | 0.019 | Osbpl10/Esyt2/Cln8/Apod/Ttpa/Ugcg/Vps4b/Apobr/Tspo/Dbi/C3/Atp9a/Mif/Acsl6/Dennd5b/Abca9/Osbpl1a/Zfyve1/Atp8a2/Acsl1/Bscl2/Osbpl9/Abcd3/Apoa4/Cd36/Xkr7/Ttc39b/Vps4a/Apoh/Abcb1a/Abcd2/Washc1/Abca7/Acsl5/Abhd5/Abca3/Abca1/Abat/Abcd1/Ano4 |
| GO:0017156 | calcium-ion regulated exocytosis | 56 | 0.483 | 1.536 | 0.019 | Vamp8/Vamp2/Vamp3/Vamp7/Snapin/Snap25/Unc13a/Arhgap17/Doc2b |
| GO:0006900 | vesicle budding from membrane | 47 | -0.464 | -1.550 | 0.019 | Arfgap3/Arfgap2/Gbf1/Dnm3/Sec24c/Pef1/Chmp5/Vapb/Pdcd6/Sar1a/Arf1/Chmp7/Wasl/Vps4a/Chmp6/Vapa |
| GO:0032272 | negative regulation of protein polymerization | 58 | -0.442 | -1.544 | 0.019 | Pfn2/Dyrk1a/Mtpn/Capn1/Evl/Myadm/Spta1/Bbs4/Twf1/Capza1/Carmil1/Cfl1/Add2/Arfgef1/Arpc2/Twf2/Vdac2 |
| GO:0055092 | sterol homeostasis | 34 | -0.496 | -1.488 | 0.019 | Apoa4/Ttc39b/Ccdc22/Acox1/Abca1/Abcd1 |
| GO:0198738 | cell-cell signaling by wnt | 164 | 0.373 | 1.403 | 0.020 | Prkaa1/Tmem9/Wdr61/Usp8/Vangl2/Gpc3/Ttc21b/Calcoco1/Fzd3/Usp47/Vcp/Fgfr3/Apc2/Vps35/Ptpn23/Gskip/Fzd2/Ctnnb1/Ubac2/Folr1/Strn/Gpc5/Mgat3/Csnk1g1/App/Ccar2/Itga3/Daam2/Csnk2b/Mdk/Lrp1/Cby1/Lgr4/Mapk14/Ppm1b/Gpc4/Dact3/Csnk1d/Ctdnep1 |
| GO:0097300 | programmed necrotic cell death | 24 | -0.554 | -1.562 | 0.020 | Ripk1/Mapk8/Rbck1/Pgam5/Cav1/Map3k7/Ppif/Itpk1/Cyld/Asah1/Casp8/Ybx3/Bok |
| GO:0007286 | spermatid development | 41 | 0.505 | 1.512 | 0.020 | Bbs2/Ube2j1/Ythdc2/Pacrg/Septin4 |
| GO:1905952 | regulation of lipid localization | 105 | -0.375 | -1.457 | 0.020 | Dbi/C3/Mif/Acsl6/Dennd5b/Atp8a2/Acsl1/Apoa4/Cd36/Ttc39b/Washc1/Abca7/Acsl5/Abhd5/Abca3/Abca1/Abat |
| GO:0010557 | positive regulation of macromolecule biosynthetic process | 442 | 0.315 | 1.307 | 0.020 | Uqcc2/Ythdf1/Rhog/Naca/Ttc5/Prkaa1/Bag1/Glmp/Calcoco1/Cst3/Slc35a4/Bmpr1b/Rptor/Mief1/Ahi1/Nup62/Cdc42/Mbtps2/Eif3e/Cys1/Soat1/Ybx1/Ubtf/Kpna6/Bcar3/Snw1/Ablim1/Igbp1/Cacybp/Hes1/Rpusd3/Map2k3/Fzd2/Ctnnb1/Pcid2/Arhgef2/App/Snx5/Vhl/Crebbp/Bloc1s2/Rhoq/Camk1/Nek7/Sirt3/Rps6kb1/Smarca2/Eif4g1/Mdk/Slc30a9/Grin1/Lgr4/Ubp1/Mapk14/Pomt2/Ddrgk1/Myo6/Yes1/Crhr2/Tsc22d1/Ankrd49/Hmga1/Supt20/Ppp2r5d/Triap1/Wbp2/Hnrnpk/Actr2/Trmt10c/Ago1/Abca2/Dcn/Ilf2/Ppp2r5b/Elavl1/Ppm1a/Eif4g3/Raf1/Eef2/Vim/Khdrbs1 |
| GO:0006633 | fatty acid biosynthetic process | 77 | -0.408 | -1.477 | 0.020 | Acsbg1/Acsl3/Acadl/Mif/Abhd3/Acsf3/Acly/Abcd3/Apoa4/Abcd2/Acss1/Acot7/Acadvl/Abcd1/Wdtc1 |
| GO:0022406 | membrane docking | 69 | 0.461 | 1.516 | 0.021 | Vamp8/Vamp2/Vps11/Ykt6/Vps33a/Vps18/Esyt1/Vps33b/Nrxn1/Ncam1/Unc13a/Ahcyl1 |
| GO:0003205 | cardiac chamber development | 54 | 0.491 | 1.547 | 0.021 | Naca/Vangl2/Ryr2/Frs2/Luzp1/Hes1/Zmpste24/Fzd2/Ctnnb1/Ap2b1/Ly6e |
| GO:2001138 | regulation of phospholipid transport | 10 | -0.701 | -1.588 | 0.021 | Dbi/Atp8a2/Abca7/Abca3 |
| GO:0045332 | phospholipid translocation | 24 | -0.552 | -1.557 | 0.021 | Atp9a/Atp8a2/Xkr7/Abcb1a/Abca7/Abca1/Ano4 |
| GO:0044003 | modulation by symbiont of host process | 14 | -0.641 | -1.584 | 0.022 | Insr/Atg5/Phb2/Vapb/Dag1/Ulk1/Ulk2 |
| GO:0016050 | vesicle organization | 194 | 0.356 | 1.361 | 0.022 | Vamp8/Uvrag/Vamp2/Tmem9/Vps11/Usp8/Washc4/Vps39/Bloc1s3/Vps18/Hook3/Vav3/Vamp3/Arfgef2/Vps33b/Vamp7/Coro1c/Snapin/Snap25/Tmed10/Pafah1b1/Vti1a/Chmp2b/Unc13a/Doc2b/Bcl2/Pdcd6ip/Rab5c/Snx11/S100a10/Snca/Ap1s2/C2cd5/Tmcc1/Als2 |
| GO:0140056 | organelle localization by membrane tethering | 65 | 0.466 | 1.512 | 0.022 | Vamp8/Vamp2/Vps11/Ykt6/Vps33a/Vps18/Esyt1/Vps33b/Nrxn1/Ncam1/Unc13a/Ahcyl1 |
| GO:0009261 | ribonucleotide catabolic process | 31 | -0.491 | -1.437 | 0.023 | Nudt10/Entpd4/Acat1/Itpa/Ada/Acot7/Ampd3/Abcd1 |
| GO:0001662 | behavioral fear response | 41 | 0.501 | 1.501 | 0.023 | Mapk8ip2/Vdac3/Eif4e/Cacna1e/Usp46/Brinp1/Vdac1/Bcl2/Dpp4/Rps6kb1/Eif4g1/Mdk/Als2 |
| GO:0002209 | behavioral defense response | 41 | 0.501 | 1.501 | 0.023 | Mapk8ip2/Vdac3/Eif4e/Cacna1e/Usp46/Brinp1/Vdac1/Bcl2/Dpp4/Rps6kb1/Eif4g1/Mdk/Als2 |
| GO:1901983 | regulation of protein acetylation | 32 | 0.536 | 1.535 | 0.023 | Prkaa1/Ctbp1/Zmpste24/App/Camk1/Sirt3/Snca/Cep295/Wbp2/Atg7 |
| GO:0072523 | purine-containing compound catabolic process | 31 | -0.487 | -1.428 | 0.023 | Nt5c1a/Nudt10/Acat1/Itpa/Ada/Acot7/Ampd3/Abcd1 |
| GO:0051608 | histamine transport | 11 | 0.739 | 1.648 | 0.023 | Vamp8/Vamp7 |
| GO:0060271 | cilium assembly | 124 | 0.388 | 1.409 | 0.023 | Bbs2/Vdac3/Vangl2/Ttbk2/Ahi1/Atg3/Atxn10/Wdr11/Bbs1/Tmem67/Ablim1/Ptpn23/Wdr35/Flna/Htt/Limk2/Dync2li1/Rab3ip/Tbc1d7/Rilpl1/Hap1/Cby1/Ttc8/Rpgrip1l/Ehd1/Cc2d2a/Tctn3/Csnk1d/Cdkl5/Ocrl/Actr2/Odf2l/Bbs9/Ift140/B9d2/Lrrc49/Cfap20/Actr3 |
| GO:0072657 | protein localization to membrane | 441 | 0.308 | 1.277 | 0.024 | Vamp8/Srp19/Vps37a/Bbs2/Rhog/Naca/Dbn1/Ttc7b/Vamp2/Ttc7/Gpc3/Emc8/Hpca/Gorasp2/Vamp3/Timm10/Golga4/Zdhhc5/Timm22/Vamp7/Cltc/Arl6ip5/Cacng4/Bbs1/Grip2/Nlgn1/Samm50/Nrxn1/Dpp6/Vps35/Micall1/Snap25/Lin7c/Folr1/Pick1/Lrrtm4/Gpc5/Fam126b/Flna/Nlgn2/Emc9/Nfasc/Rab3ip/Efr3b/Vps37c/Kcnip3/Clstn1/Itga3/Rilpl1/Rhoq/F11r/Bcl2l1/S100a10/Itgb1/Arl5a/Rer1/Tub/Myo5a/Cnpy4/Lin7a/Gas6/Zfand2b/Itgam/C2cd5/Zfyve27/Synj2bp/Lrp1/Grin1/Ttc8/Arf6/Prkcz/Cyp46a1/Ehd1/Thy1/Get4 |
| GO:1903902 | positive regulation of viral life cycle | 19 | -0.604 | -1.588 | 0.024 | Ddb1/Bsg/Chmp3/Vapb/Vps4b/Vps4a/Chmp2a/Vps37b/Tyro3/Vapa |
| GO:0031503 | protein-containing complex localization | 161 | 0.373 | 1.403 | 0.024 | Dbn1/Ran/Vamp2/Ttc21b/Hpca/Stau2/Usp46/Efnb2/Snapin/Cacng4/Bbs1/Grip2/Nlgn1/Exoc3l4/Nrxn1/Xpot/Vps35/Snap25/Ddx19a/Pick1/Wdr35/Pcid2/Ap2b1/Dync2li1/Mios/Kif5a/Clstn1/Hap1/Tub/Exoc3/Synj2bp/Prkcz/Myo6/Gpc4/Stau1/Nedd4/Ezr/Ift140/Grip1/Xpo1/Ndc1/Arhgap44/Nme7/Slc1a1/Syne1/Ttc30a2/Washc5/Rab8a |
| GO:0034204 | lipid translocation | 26 | -0.532 | -1.500 | 0.024 | Atp9a/Atp8a2/Xkr7/Abcb1a/Abca7/Abca1/Ano4 |
| GO:0050994 | regulation of lipid catabolic process | 35 | -0.476 | -1.441 | 0.025 | Idh1/Apoa2/Fmc1/Cnr1/Dbi/Bscl2/Cpt1a/Apoa4/Apoh/Abcd2/Acsl5/Abhd5/Abcd1 |
| GO:0060538 | skeletal muscle organ development | 59 | 0.464 | 1.484 | 0.025 | Uqcc2/Naca/Prkaa1/Myorg/Snw1/Ctnnb1/Bcl2/Rps6kb1/Homer1/Mapk14/Crhr2/Cntfr |
| GO:0033006 | regulation of mast cell activation involved in immune response | 20 | 0.607 | 1.571 | 0.025 | Vamp8/Vamp7/Fgr |
| GO:0043304 | regulation of mast cell degranulation | 20 | 0.607 | 1.571 | 0.025 | Vamp8/Vamp7/Fgr |
| GO:0006511 | ubiquitin-dependent protein catabolic process | 339 | 0.316 | 1.279 | 0.026 | Vps37a/Ube2j1/Ubr3/Ube2k/Usp9x/Svip/Aup1/Ube2j2/Vps28/Usp8/Ube2i/Cdc23/Psmd1/Ubap1/Ube2v2/Ube2d1/Ube2l3/Usp4/Usp47/Amn1/Vcp/Agbl4/Cul5/Nub1/Usp46/Uchl1/Rpl23/Tmem67/Usp30/Ube2a/Ube2n/Rnf167/Ltn1/Ptpn23/Cul9/Sharpin/Dcaf11/Ctnnb1/Hace1/Eif3h/Wwp1/Ufd1/Uba1/Vps37c/Tollip/Ccar2/Usp24/Dnajb12/Vps25/Zfand2b/Map1a/Ubr2/Smurf2/Hecw1/Ufl1/Psmd2/Atxn3/Ddrgk1/Marchf6/Usp7/Fbxo7/Ube2g2/Gclc/Ubr1/Csnk1d/Erlin1/Commd1/Kctd6/Atg7/Pten/Nedd4/Os9/Rc3h1/Arih2/Otud7b/Fbxl18 |
| GO:0007281 | germ cell development | 76 | 0.425 | 1.430 | 0.026 | Bbs2/Ube2j1/Ythdc2/Pacrg/Bmpr1b/Tdrd7/Septin4/Ctnnb1/Pafah1b1/Bcl2/Bcl2l1/Rps6kb1/Mdk |
| GO:1905114 | cell surface receptor signaling pathway involved in cell-cell signaling | 230 | 0.345 | 1.350 | 0.026 | Dbn1/Prkaa1/Mapk8ip2/Tmem9/Wdr61/Usp8/Vangl2/Gpc3/Ttc21b/Calcoco1/Fzd3/Glra1/Usp47/Vcp/Fgfr3/Grip2/Nlgn1/Apc2/Nrxn1/Vps35/Gskip/Fzd2/Ctnnb1/Ubac2/Folr1/Strn/Gpc5/Mgat3/Nlgn2/Csnk1g1/App/Ccar2/Itga3/Daam2/Snca/Begain/Csnk2b/Itgam/Mdk/Lrp1/Grin1/Cby1/Lgr4/Mapk14/Prkcz/Ppm1b/Gpc4/Dact3/Csnk1d/Ctdnep1 |
| GO:0030308 | negative regulation of cell growth | 92 | -0.378 | -1.428 | 0.026 | Ahsg/Cryab/Ei24/Ndufa13/Cfl1/Fgf13/Acvr1b/Apbb1/Cth/Adipor2/Aatk/Ulk1/Ulk2 |
| GO:0006354 | DNA-templated transcription, elongation | 11 | 0.731 | 1.632 | 0.026 | Wdr61/Ubtf/Ctnnb1/Pcid2/Ccar2 |
| GO:0061061 | muscle structure development | 221 | 0.346 | 1.347 | 0.026 | Uqcc2/Naca/Prkaa1/Cmtm5/Myh9/Vangl2/Chat/Ryr2/Frs2/Ybx1/Efnb2/Uchl1/Utrn/Myorg/Snw1/Cacybp/Hes1/Zmpste24/Fzd2/Ctnnb1/Ly6e/Tmod3/Bcl2/Camk1/Itgb1/Erbb3/Rps6kb1/Smarca2/Homer1/Cby1/Mapk14/Ttn/Ehd1/Crhr2/Fkbp1a/Cntfr/G6pdx/Actg1/Atg7/Csrp2/Nf1/Prkg1/Luc7l/Sgcb |
| GO:0016055 | Wnt signaling pathway | 162 | 0.370 | 1.392 | 0.026 | Prkaa1/Tmem9/Wdr61/Usp8/Vangl2/Gpc3/Ttc21b/Calcoco1/Fzd3/Usp47/Vcp/Fgfr3/Apc2/Vps35/Gskip/Fzd2/Ctnnb1/Ubac2/Folr1/Strn/Gpc5/Mgat3/Csnk1g1/App/Ccar2/Itga3/Daam2/Csnk2b/Mdk/Lrp1/Cby1/Lgr4/Mapk14/Ppm1b/Gpc4/Dact3/Csnk1d/Ctdnep1/Pten |
| GO:1901673 | regulation of mitotic spindle assembly | 13 | -0.634 | -1.535 | 0.027 | Eml3/Chmp1a/Ccsap/Chmp5/Vps4b/Cep97/Drg1/Chmp2a |
| GO:0019941 | modification-dependent protein catabolic process | 347 | 0.323 | 1.313 | 0.027 | Vps37a/Ube2j1/Ubr3/Ube2k/Usp9x/Svip/Aup1/Ube2j2/Vps28/Usp8/Uba7/Ube2i/Cdc23/Psmd1/Ubap1/Ube2v2/Ube2d1/Ube2l3/Usp4/Usp47/Amn1/Vcp/Agbl4/Cul5/Nub1/Usp46/Uchl1/Rpl23/Tmem67/Usp30/Ube2a/Ube2n/Isg15/Rnf167/Ltn1/Ptpn23/Cul9/Zmpste24/Sharpin/Dcaf11/Ctnnb1/Hace1/Eif3h/Wwp1/Ufd1/Uba1/Vps37c/Tollip/Ccar2/Usp24/Dnajb12/Vps25/Zfand2b/Map1a/Ubr2/Smurf2/Hecw1/Ufl1/Psmd2/Atxn3/Ddrgk1/Marchf6/Usp7/Fbxo7/Ube2g2/Gclc/Ubr1/Csnk1d/Erlin1/Commd1/Kctd6/Atg7/Pten/Nedd4/Os9/Rc3h1/Arih2/Otud7b/Fbxl18 |
| GO:0033539 | fatty acid beta-oxidation using acyl-CoA dehydrogenase | 10 | -0.686 | -1.554 | 0.027 | Etfa/Acadm/Etfb/Acadl/Acad11/Acadvl |
| GO:0070266 | necroptotic process | 18 | -0.581 | -1.506 | 0.028 | Rnf31/Ripk1/Rbck1/Pgam5/Cav1/Map3k7/Ppif/Itpk1/Cyld/Casp8/Ybx3/Bok |
| GO:0043254 | regulation of protein-containing complex assembly | 274 | -0.293 | -1.269 | 0.028 | Pycard/Gmfb/Gba2/Cck/Snx9/Brk1/Pfn2/Dyrk1a/Rhoa/Cav1/Mtpn/Capn1/Map3k7/Ctnna2/Ap1ar/Tfrc/Evl/Stx1a/Bak1/Coro1b/Apc/Myadm/Ctnnbip1/Spta1/Wasf3/Bin1/Piezo1/Bid/Atat1/Bbs4/Coro1a/Jam3/Cttn/Psmc3/Twf1/Capza1/Myo1c/Arpc1b/Cryab/Plek/Dnajc15/Caly/Carmil1/Cyfip1/Sar1a/Fes/Psmc2/Drg1/Arf1/Cfl1/Wasl/Arpc1a/Wars/Add2/Cd36/Msn/Washc1/Arfgef1/Bag4/Arpc2/Aida/Abca3/Abca1/Wasf2/Ica1/Ulk1/Twf2/Wasf1/Abi2/Vdac2 |
| GO:0031100 | animal organ regeneration | 12 | -0.665 | -1.587 | 0.028 | Apoa2/Egfr/Apoh/Vtn |
| GO:0045185 | maintenance of protein location | 55 | -0.428 | -1.470 | 0.029 | Bbs4/Hk2/Twf1/Tspo/Chchd10/Vps13d/Ccdc22/Ywhab/Twf2/Vps13a/Adcy6 |
| GO:1903426 | regulation of reactive oxygen species biosynthetic process | 56 | -0.429 | -1.479 | 0.029 | Pkd2/Rhoa/Cav1/Akt1/Rock2/Dynll1/Agt/Gla/Sirpa/Insr/Sphk2/Ddah1/Hrh1/Adgrb1/Tspo/Cd36/Abcd2/Aif1/Abcd1 |
| GO:0002791 | regulation of peptide secretion | 166 | 0.370 | 1.394 | 0.029 | Vamp8/Uqcc2/Rhbdd3/Glrx/Rptor/Brsk2/Ahi1/Cacna1e/Pard6a/Pam/Glul/Nrxn1/Vps35/Ptpn23/Tmed10/Pick1/Nlgn2/Doc2b/Oxct1/Arfip1/Nadk/Sirt3/Ptpn11/Hadh/Lrp1/Jagn1/Arf6/Ttn/Crhr2/Erp29/Atg7/Gpld1/Abcg1/Ezr/Itsn1/Tiam1 |
| GO:1903362 | regulation of cellular protein catabolic process | 147 | 0.370 | 1.370 | 0.029 | Uqcc2/Ube2k/Svip/Wdr91/Vps11/Ube2v2/Vcp/Agbl4/Nub1/Rpl23/Tmem67/Vps35/Mgat3/Eif3h/Atpif1/Ccar2/Map1a/Ufl1/Lrp1/Atxn3/Ddrgk1/Usp7/Gclc/Csnk1d/Commd1/Atg7/Pten/Abca2/Arih2/Ezr |
| GO:0009896 | positive regulation of catabolic process | 241 | 0.335 | 1.318 | 0.029 | Uqcc2/Ythdf1/Svip/Rhbdd3/Prkaa1/Vps11/Vps28/Bnip3/Gpc3/Grsf1/Ube2v2/Fyco1/Vcp/Agbl4/Rida/Nub1/Tmem67/Apc2/Vps35/Hace1/Htt/Wwp1/Vdac1/App/Atpif1/Daglb/Snca/Smurf2/Hecw1/Ufl1/Lrp1/Ptpn1/Atxn3/Ddrgk1/Fbxo7/Pip4k2c/Gclc/Csnk1d/Atg7/Gpld1/Pten/Nedd4/Rc3h1/Abca2/Dcn/Arih2/Ezr |
| GO:0033108 | mitochondrial respiratory chain complex assembly | 66 | 0.452 | 1.471 | 0.029 | Uqcc2/Ndufs7/Ndufb5/Ndufa1/Ndufb11/Ndufb9/Samm50/Cox20/Ndufb7/Ndufa11/Ndufb1/Ndufb8/Ndufa2/Ndufaf3 |
| GO:0044782 | cilium organization | 137 | 0.383 | 1.400 | 0.030 | Bbs2/Vdac3/Vangl2/Ttc21b/Ttbk2/Ahi1/Atg3/Atxn10/Wdr11/Bbs1/Tmem67/Ablim1/Ptpn23/Wdr35/Flna/Htt/Limk2/Dync2li1/Rab3ip/Tbc1d7/Rilpl1/Hap1/Tub/Cby1/Ttc8/Rpgrip1l/Ehd1/Cc2d2a/Tctn3/Csnk1d/Cdkl5/Ttc17/Ocrl/Actr2/Odf2l/Bbs9/Ift140/B9d2/Lrrc49/Cfap20/Actr3/Nme7/Syne1/Mark4/Ttc30a2 |
| GO:0072330 | monocarboxylic acid biosynthetic process | 94 | -0.364 | -1.378 | 0.030 | Acsbg1/Acsl3/Aldh1a1/Acadl/Mif/Abhd3/Acsf3/Aldh1a2/Acly/Abcd3/Apoa4/Abcd2/Acss1/Acot7/Acadvl/Abcd1/Wdtc1 |
| GO:0010573 | vascular endothelial growth factor production | 10 | -0.680 | -1.540 | 0.030 | Eif2ak3/Ndrg2/Bsg/Adgrg1/C5ar1/C3 |
| GO:1902188 | positive regulation of viral release from host cell | 12 | -0.660 | -1.576 | 0.030 | Ddb1/Chmp3/Vapb/Vps4b/Vps4a/Chmp2a/Vps37b/Vapa |
| GO:0002703 | regulation of leukocyte mediated immunity | 70 | 0.455 | 1.503 | 0.030 | Vamp8/Vav1/Fcgr1/Hspd1/Vamp7/H2-K1/Fgr/Dpp4/Susd4/Itgam/Tap2/Prkcz/Cd84 |
| GO:0009615 | response to virus | 84 | 0.425 | 1.463 | 0.031 | Vamp8/Aup1/Tspan6/Bnip3/Unc93b1/Agbl4/Isg15/Zmpste24/Ufd1/Bcl2/Crebbp/Bcl2l1/Lsm14a/Traf3/Gpam/Mapk14/Ppm1b/Itgb8/Atg7 |
| GO:0043302 | positive regulation of leukocyte degranulation | 15 | 0.658 | 1.590 | 0.031 | Vamp8/Vamp7/Fgr |
| GO:0030834 | regulation of actin filament depolymerization | 39 | -0.462 | -1.441 | 0.031 | Mtpn/Wdr1/Evl/Spta1/Dstn/Twf1/Capza1/Plek/Carmil1/Cfl1/Add2/Arpc2/Twf2 |
| GO:0051607 | defense response to virus | 65 | 0.449 | 1.459 | 0.031 | Vamp8/Tspan6/Bnip3/Unc93b1/Agbl4/Isg15/Zmpste24/Ufd1/Bcl2/Bcl2l1/Lsm14a/Traf3/Gpam |
| GO:0140546 | defense response to symbiont | 65 | 0.449 | 1.459 | 0.031 | Vamp8/Tspan6/Bnip3/Unc93b1/Agbl4/Isg15/Zmpste24/Ufd1/Bcl2/Bcl2l1/Lsm14a/Traf3/Gpam |
| GO:0006639 | acylglycerol metabolic process | 72 | -0.389 | -1.402 | 0.031 | Lmf1/Lpin1/Cav1/Plpp1/Lipe/Dgkb/Apoa2/Gykl1/Apobr/Abhd6/Dbi/Abhd12/C3/Acsl6/Abhd16a/Acsl1/Cpt1a/Apoa4/Cd36/Apoh/Acsl5/Abhd5/Atg14 |
| GO:0090208 | positive regulation of triglyceride metabolic process | 14 | -0.620 | -1.534 | 0.031 | Apoa4/Cd36/Apoh/Acsl5/Abhd5 |
| GO:1903364 | positive regulation of cellular protein catabolic process | 82 | 0.428 | 1.469 | 0.031 | Uqcc2/Vps11/Ube2v2/Vcp/Agbl4/Nub1/Tmem67/Vps35/Atpif1/Lrp1/Atxn3/Ddrgk1/Gclc/Csnk1d/Atg7/Pten/Abca2/Arih2/Ezr |
| GO:0010800 | positive regulation of peptidyl-threonine phosphorylation | 11 | 0.723 | 1.613 | 0.031 | Ube2k/Rptor/App/Cab39/Tnks1bp1 |
| GO:0072659 | protein localization to plasma membrane | 221 | 0.339 | 1.319 | 0.032 | Vamp8/Bbs2/Rhog/Ttc7b/Vamp2/Ttc7/Gorasp2/Vamp3/Golga4/Zdhhc5/Vamp7/Cltc/Arl6ip5/Bbs1/Grip2/Nrxn1/Dpp6/Vps35/Fam126b/Flna/Nfasc/Efr3b/Kcnip3/Itga3/Rilpl1/Rhoq/F11r/Bcl2l1/S100a10/Itgb1/Rer1/Myo5a/Cnpy4/Gas6/C2cd5/Zfyve27/Lrp1/Ttc8/Arf6/Prkcz |
| GO:0071346 | cellular response to interferon-gamma | 51 | 0.474 | 1.477 | 0.033 | Vamp8/Vps26b/Vamp3/Cdc42 |
| GO:0032368 | regulation of lipid transport | 93 | -0.366 | -1.385 | 0.033 | Dbi/Mif/Acsl6/Dennd5b/Atp8a2/Acsl1/Apoa4/Ttc39b/Washc1/Abca7/Acsl5/Abca3/Abca1/Abat |
| GO:0055078 | sodium ion homeostasis | 27 | -0.520 | -1.483 | 0.033 | Ext1/Ext2/Tesc/Slc12a2/Atp1b1/Adora1/Ace/Tac1/Slc8a1/Slc1a3/Atp1b3/Atp1a3/Agt/Comt/Atp1a4/Atp1a2/Atp1a1/Atp12a |
| GO:0050778 | positive regulation of immune response | 166 | 0.366 | 1.379 | 0.034 | Vamp8/Ube2k/Usp9x/Vav1/Fcgr1/Slc39a10/Vav3/Hspd1/Cd276/Dgkz/Vamp7/Usp46/Ube2n/Igkc/H2-K1/Fgr/Bcl2/Susd4/Lsm14a/Eif2b3/Itgam/Tap2/Bcar1/Prkcz/Ighm/Thy1/Pla2g4a/Gpld1/Plcg1/Rc3h1/Ezr/Usp12/Khdrbs1/Nckap1l/Lpxn/Ptpn6/Hspa8/Fcgr2b/Gab2/C4b/Serping1/Pde4b |
| GO:0000278 | mitotic cell cycle | 310 | 0.325 | 1.309 | 0.034 | Tubb2b/Vcpip1/Tuba8/Ythdc2/Ran/Tubb2a/Eif4e/Tubgcp5/Usp8/Cdc23/Tubb6/Anln/Rptor/Brsk2/Reep3/Tuba3a/Unc119/Dgkz/Nup62/Cdc42/Ctdsp1/Fzd3/Tubb1/Usp47/Uba3/Vcp/Cltc/Fgfr3/Tuba4a/Hes1/Cul9/Zmpste24/Tubb4a/Ctnnb1/Brinp3/Pafah1b1/Tubg1/Pcid2/Brinp1/Tubb5/Flna/Ttll12/Rpl17/Htt/Chmp2b/Tmod3/Arhgef2/Kif3b/Tubb4b/Bcl2/App/Pdcd6ip/Crebbp/Bcl2l1/Itgb1/Abraxas2/Cradd/Dync1h1/Ptpn11/Lcmt1/Rps6kb1/Lsm14a |
| GO:0034250 | positive regulation of cellular amide metabolic process | 92 | 0.407 | 1.418 | 0.034 | Uqcc2/Ythdf1/Slc35a4/Mief1/Eif3e/Rpusd3/Sirt3/Rps6kb1/Eif4g1/Slc2a13/Abcg1/Trmt10c/Abca2/Elavl1/Eif4g3/Eef2/Vim/Khdrbs1/Eif4g2/Dnajc3/Dhx36/Chrna7 |
| GO:0033604 | negative regulation of catecholamine secretion | 12 | -0.656 | -1.566 | 0.034 | Cnr1/Chga/Abat |
| GO:0002444 | myeloid leukocyte mediated immunity | 49 | 0.469 | 1.454 | 0.034 | Vamp8/Vamp2/Fcgr1/Vamp7/Tusc2 |
| GO:0010863 | positive regulation of phospholipase C activity | 15 | -0.610 | -1.525 | 0.034 | Phb/Pdgfrb/Flt1/Gnaq/Abl2/Agt/Phb2/Adcyap1r1/Adra1a |
| GO:0050999 | regulation of nitric-oxide synthase activity | 14 | -0.616 | -1.522 | 0.035 | Atp2b4/Ptk2b/Acvr2a/Cav1/Akt1/Gla/Egfr/Cnr1 |
| GO:0051693 | actin filament capping | 28 | -0.500 | -1.435 | 0.035 | Mtpn/Evl/Spta1/Twf1/Capza1/Carmil1/Cfl1/Add2/Arpc2/Twf2 |
| GO:0090263 | positive regulation of canonical Wnt signaling pathway | 49 | 0.467 | 1.447 | 0.035 | Tmem9/Usp8/Gpc3/Ttc21b/Usp47/Vcp/Fgfr3/Vps35/Gskip/Gpc5/Csnk1g1/Ccar2/Daam2/Lgr4/Ppm1b/Csnk1d/Ctdnep1 |
| GO:0048483 | autonomic nervous system development | 13 | 0.670 | 1.553 | 0.036 | Vcam1/Fzd3/Hes1/Ctnnb1/Plxna4 |
| GO:0031328 | positive regulation of cellular biosynthetic process | 494 | 0.300 | 1.254 | 0.037 | Uqcc2/Ythdf1/Rhog/Naca/Svip/Ttc5/Prkaa1/Bag1/Glmp/Calcoco1/Cst3/Slc35a4/Bmpr1b/Rptor/Mief1/Ahi1/Nup62/Cdc42/Mbtps2/Eif3e/Cys1/Soat1/Ybx1/Vcp/Ubtf/Kpna6/Bcar3/Snw1/Ablim1/Igbp1/Vps35/Cacybp/Hes1/Rpusd3/Map2k3/Fzd2/Pcx/Ctnnb1/Pcid2/Elovl5/Arhgef2/Mid1ip1/App/Snx5/Vhl/Crebbp/Bloc1s2/Rhoq/Camk1/Bcl2l1/Nek7/Sirt3/Rps6kb1/Snca/Smarca2/Eif4g1/Mdk/Slc30a9/Grin1/Gpam/Lgr4/Ddah2/Ubp1/Mapk14/Dgat1/Pomt2/Ddrgk1/Myo6/Yes1/Crhr2/Tsc22d1/Ankrd49/Hmga1/Supt20/Pla2g4a/Ppp2r5d/Triap1/Ctdnep1/Wbp2/Hnrnpk/Gpld1/Actr2/Trmt10c/Ago1/Abca2/Dcn/Ilf2/Ppp2r5b/Elavl1/Ppm1a/Eif4g3/Raf1/Eef2/Vim/Khdrbs1 |
| GO:0045923 | positive regulation of fatty acid metabolic process | 17 | -0.580 | -1.494 | 0.038 | Cpt1a/Apoa4/Abcd2/Acsl5/Abcd1 |
| GO:0006304 | DNA modification | 24 | 0.581 | 1.548 | 0.038 | Usp9x/Tdrkh/Zmpste24/Fkbp6/Usp7 |
| GO:0006417 | regulation of translation | 169 | 0.351 | 1.330 | 0.038 | Uqcc2/Ythdf1/Eif4ebp2/Eif4e/Cnot11/Ddx6/Cnot9/Slc35a4/Mief1/Eif3e/Rida/Ltn1/Rpusd3 |
| GO:0016236 | macroautophagy | 146 | 0.375 | 1.385 | 0.039 | Vamp8/Vmp1/Uvrag/Aup1/Bnip3/Vps33a/Wdr45/Atg12/Vps16/Fyco1/Atg3/Gabarapl1/Vcp/Ubxn6/Snapin/Usp30/Uba5 |
| GO:0031060 | regulation of histone methylation | 12 | 0.680 | 1.551 | 0.041 | Wdr61/Snw1/Ctnnb1 |
| GO:0018205 | peptidyl-lysine modification | 88 | 0.400 | 1.390 | 0.042 | Prkaa1/Vipas39/Wdr61/Ube2i/Uba2/Vps33b/Ctbp1/Snw1/Zmpste24/Senp8/Ctnnb1/App/Dpy30/Tollip/Crebbp/Sirt3/Snca |
| GO:0008589 | regulation of smoothened signaling pathway | 39 | 0.483 | 1.444 | 0.042 | Gpc3/Ttc21b/Ttbk2/Wdr11/Vcp/Fgfr3/Pdcl/Rpgrip1l/Mgrn1/Enpp1/Herc4/Kctd6/Prkacb/Ift140 |
| GO:0042308 | negative regulation of protein import into nucleus | 11 | -0.663 | -1.552 | 0.042 | Akap1/Apod/Ei24/Cd36/Ufm1 |
| GO:1904590 | negative regulation of protein import | 11 | -0.663 | -1.552 | 0.042 | Akap1/Apod/Ei24/Cd36/Ufm1 |
| GO:0006638 | neutral lipid metabolic process | 73 | -0.381 | -1.373 | 0.042 | Apoa2/Gykl1/Apobr/Abhd6/Dbi/Abhd12/C3/Acsl6/Abhd16a/Acsl1/Cpt1a/Apoa4/Cd36/Apoh/Acsl5/Abhd5/Atg14 |
| GO:0002694 | regulation of leukocyte activation | 172 | 0.342 | 1.297 | 0.043 | Vamp8/Rhbdd3/Adk/Slc39a10/Bloc1s3/Vcam1/Vav3/Hspd1/Cd276/Vamp7/Efnb2/Igkc/Hes1/Ctnnb1/Fgr/Pcid2/Bcl2/Atp11c/Dpp4/Gas6/Snca/Itgam/Mdk/Gpam/Prkcz/Cd84/Ighm/Thy1/Fbxo7/Efnb3/Pla2g4a |
| GO:1905475 | regulation of protein localization to membrane | 145 | 0.360 | 1.331 | 0.043 | Vamp8/Rhog/Dbn1/Gpc3/Hpca/Zdhhc5/Vamp7/Cltc/Grip2/Nrxn1/Dpp6/Gpc5/Nlgn2/Itga3/Rhoq/Bcl2l1/Itgb1/Rer1/Myo5a/Cnpy4/Itgam/C2cd5/Lrp1/Arf6/Prkcz |
| GO:0042596 | fear response | 44 | 0.484 | 1.453 | 0.043 | Mapk8ip2/Vdac3/Eif4e/Cacna1e/Usp46/Brinp1/Vdac1/Bcl2/Dpp4/Rps6kb1/Eif4g1/Mdk/Als2 |
| GO:0015914 | phospholipid transport | 46 | -0.428 | -1.424 | 0.044 | Dbi/Atp9a/Atp8a2/Apoa4/Xkr7/Abcb1a/Abca7/Abca3/Abca1/Ano4 |
| GO:0034341 | response to interferon-gamma | 54 | 0.449 | 1.413 | 0.044 | Vamp8/Vps26b/Vamp3/Cdc42 |
| GO:0000209 | protein polyubiquitination | 101 | 0.385 | 1.363 | 0.044 | Ube2j1/Ube2k/Zswim2/Ube2j2/Cdc23/Ube2v2/Ube2d1/Ube2l3/Ube2a/Ube2n/Rnf167/Sharpin/Ctnnb1/Hace1/Wwp1/Bcl2/Prpf19/Anapc13/Smurf2/Hecw1/Traf3/Marchf6/Mgrn1/Ube2g2/Aktip/Nedd4/Rc3h1/Arih2 |
| GO:0006891 | intra-Golgi vesicle-mediated transport | 23 | -0.537 | -1.486 | 0.046 | Rab6b/Golga5/Copb2/Cope/Cog3/Cog1/Cux1/Copz1/Vti1b/Cog5 |
| GO:0009914 | hormone transport | 174 | 0.341 | 1.296 | 0.046 | Vamp8/Uqcc2/Vamp2/Glrx/Slco1c1/Rptor/Brsk2/Vamp7/Cacna1e/Pclo/Glul/Nrxn1/Snap25/Pick1/Htt/Nlgn2/Doc2b/Oxct1/Nadk/Sirt3/Myo5a/Ptpn11/Hadh/Lrp1/Jagn1/Dgat1 |
| GO:0032008 | positive regulation of TOR signaling | 31 | 0.522 | 1.484 | 0.046 | Wdr59/Rptor/Lars/Mios/Gas6 |
| GO:0000226 | microtubule cytoskeleton organization | 277 | 0.318 | 1.270 | 0.046 | Bbs2/Tubb2b/Tuba8/Uvrag/Prkaa1/Myh9/Tubb2a/Tubgcp5/Nefl/Tubb6/Ttbk2/Hook3/Gcc2/Brsk2/Tuba3a/Nup62/Tubb1/Vcp/Cltc/Pard6a/Bbs1/Tuba4a/Tmem67/Apc2/Slc39a12/Cul9/Tubb4a/Ctnnb1/Pafah1b1/Tubg1/Tubb5/Flna/Htt/Chmp2b/Limk2/Arhgef2/Kif3b/Mid1ip1/Tubb4b/Pdcd6ip/Gas2l1/Cnp/Itgb1/Abraxas2/Dync1h1/Snca/Lsm14a/Stmn2/Map1a/Nav3/Mdm1/Prkcz/Map7d1/Atxn3 |
| GO:1990778 | protein localization to cell periphery | 276 | 0.318 | 1.266 | 0.046 | Vamp8/Bbs2/Rhog/Ttc7b/Vamp2/Ttc7/Gorasp2/Vamp3/Golga4/Zdhhc5/Vamp7/Cltc/Arl6ip5/Cacng4/Bbs1/Grip2/Nrxn1/Dpp6/Vps35/Snap25/Lin7c/Fam126b/Flna/Nfasc/Efr3b/Kcnip3/Clstn1/Itga3/Rilpl1/Rhoq/F11r/Bcl2l1/S100a10/Itgb1/Rer1/Tub/Myo5a/Cnpy4/Lin7a/Gas6/C2cd5/Zfyve27/Lrp1/Grin1/Ttc8/Arf6/Prkcz/Epb41l2 |
| GO:0051016 | barbed-end actin filament capping | 19 | -0.580 | -1.524 | 0.047 | Mtpn/Evl/Twf1/Capza1/Carmil1/Cfl1/Add2/Arpc2/Twf2 |
| GO:0046883 | regulation of hormone secretion | 136 | 0.374 | 1.364 | 0.047 | Vamp8/Uqcc2/Glrx/Rptor/Brsk2/Vamp7/Cacna1e/Glul/Nrxn1/Snap25/Pick1/Nlgn2/Doc2b/Oxct1/Nadk/Sirt3/Ptpn11/Hadh/Lrp1/Jagn1 |
| GO:0007224 | smoothened signaling pathway | 60 | 0.437 | 1.398 | 0.047 | Gpc3/Ttc21b/Ttbk2/Wdr11/Vcp/Fgfr3/Evc2/Hes1/Pdcl/Rpgrip1l/Mgrn1/Enpp1/Herc4/Cc2d2a/Tctn3/Kctd6/Prkacb/Ift140 |
| GO:0002886 | regulation of myeloid leukocyte mediated immunity | 33 | 0.512 | 1.482 | 0.047 | Vamp8/Fcgr1/Vamp7/Fgr |
| GO:0002275 | myeloid cell activation involved in immune response | 42 | 0.479 | 1.433 | 0.048 | Vamp8/Vamp2/Vamp7 |
| GO:0047496 | vesicle transport along microtubule | 40 | 0.476 | 1.430 | 0.048 | Bloc1s3/Fyco1/Vamp7/Snapin/Pafah1b1/Htt/Kif5a/Borcs5/Bloc1s2/Hap1/Dync1h1/Prkcz |
| GO:0097091 | synaptic vesicle clustering | 18 | 0.615 | 1.560 | 0.048 | Pclo/Nlgn1/Nrxn1/Ctnnb1/Nlgn2/Bcl2l1/Pten/Snap91/Syn2/Syn3 |
| GO:0060070 | canonical Wnt signaling pathway | 113 | 0.381 | 1.366 | 0.049 | Tmem9/Usp8/Gpc3/Ttc21b/Fzd3/Usp47/Vcp/Fgfr3/Apc2/Vps35/Gskip/Fzd2/Ctnnb1/Ubac2/Folr1/Gpc5/Mgat3/Csnk1g1/App/Ccar2/Daam2/Mdk/Cby1/Lgr4/Mapk14/Ppm1b/Dact3/Csnk1d/Ctdnep1/Pten |
| GO:1901293 | nucleoside phosphate biosynthetic process | 127 | -0.334 | -1.337 | 0.049 | Oas1a/Dcakd/Aldoa/Impdh2/Aprt/Naprt/Adcy1/Atp5j/Ppat/Dld/Pnp/Acsl4/Adcy9/Gucy1b1/Paics/Flcn/Pdk2/Ampd2/Ak5/Adsl/Adcy7/Cmpk2/Atic/Atp5c1/Pdk1/Atp5h/Dtymk/Sphk2/Ak1/Adcy8/Uqcc3/Flad1/Adss/Coasy/Atp5j2/Adcy5/Pfas/Nudt2/Adssl1/Acsl6/Acat1/Acsl1/Acly/Ak2/Ada/Adcy10/Cmpk1/Acss1/Acsl5/Acot7/Ampd3/Mpc1/Ak4/Adcy6 |
| GO:0032006 | regulation of TOR signaling | 62 | 0.442 | 1.420 | 0.049 | Wdr59/Prkaa1/Rptor/Alg13/Lars/Zmpste24/Mios/Tbc1d7/Gas6/Ubr2/Slc7a3/Ubr1/Dgkq |
| GO:0045601 | regulation of endothelial cell differentiation | 14 | 0.650 | 1.537 | 0.049 | Vcl/Ctnnb1/Vhl/F11r |
| GO:0051645 | Golgi localization | 14 | 0.647 | 1.528 | 0.049 | Uvrag/Hook3/Cdc42/Arcn1/Ripor1 |
| GO:0033233 | regulation of protein sumoylation | 10 | 0.710 | 1.531 | 0.049 | Ube2i/Uba2/Ctnnb1/Tollip |
| GO:0051495 | positive regulation of cytoskeleton organization | 151 | -0.322 | -1.304 | 0.050 | Pycard/Gmfb/Tgfbr1/Snx9/Brk1/Pfn2/Rhoa/Cav1/Ctnna2/Ap1ar/Rock2/Wdr1/Evl/Coro1b/Apc/Ntrk3/Wasf3/Bin1/Arhgef10l/Katnb1/Coro1a/Cttn/Dstn/Myo1c/Arpc1b/Plek/Carmil1/Vps4b/Cyfip1/Fes/Drg1/Arf1/Cfl1/Wasl/Arpc1a/Sdc4/Washc1/Bag4/Arpc2/Wasf2/Wasf1/Abi2 |
| GO:0010608 | posttranscriptional regulation of gene expression | 211 | 0.328 | 1.275 | 0.050 | Uqcc2/Ythdf1/Eif4ebp2/Bag1/Eif4e/Cnot11/Ddx6/Cnot9/Slc35a4/Mief1/Tdrd7/Eif3e/Ybx1/Rida/Ltn1/Rpusd3/Zmpste24 |
| MF Terms | | | | | | |
| GO:0005484 | SNAP receptor activity | 30 | 0.704 | 1.966 | 0.000 | Vamp8/Use1/Vamp2/Ykt6/Vamp3/Vamp7/Snap25/Vti1a/Pick1/Bnip1 |
| GO:0016289 | CoA hydrolase activity | 14 | -0.786 | -1.912 | 0.000 | Acot1/Acot6/Acot2/Acot13/Acot8/Acot7/Acot9 |
| GO:0030674 | protein-macromolecule adaptor activity | 153 | 0.444 | 1.640 | 0.001 | Vamp8/Wwc1/Use1/Vamp2/Mapk8ip2/Myh9/Vps11/Ykt6/Nefl/Napb/Vps18/Rptor/Vamp3/Nup62/Frs2/Vamp7/Snap25/Vti1a/Pick1/Bnip1/Hip1/Vhl/Gas2l1/Cradd/Ptpn11/Gas6/Map1a |
| GO:0047617 | acyl-CoA hydrolase activity | 12 | -0.801 | -1.891 | 0.001 | Acot1/Acot6/Acot2/Acot13/Acot8/Acot7/Acot9 |
| GO:0048038 | quinone binding | 10 | 0.871 | 1.897 | 0.001 | Vkorc1/Vkorc1l1/Ndufs7/Dhodh/Sqor |
| GO:0015662 | ion transmembrane transporter activity, phosphorylative mechanism | 17 | -0.724 | -1.868 | 0.002 | Atp6v0c/Atp13a4/Atp1a4/Atp1a2/Anxa5/Atp2b1/Atp1a1/Atp2b3/Atp2b2/Atp12a |
| GO:0016878 | acid-thiol ligase activity | 19 | -0.686 | -1.839 | 0.002 | Acsbg1/Acsl3/Aacs/Acsl6/Acsf3/Acsl1/Acss1/Acsl5 |
| GO:0016290 | palmitoyl-CoA hydrolase activity | 11 | -0.788 | -1.816 | 0.003 | Acot1/Acot2/Acot13/Acot8/Acot7/Acot9 |
| GO:0070491 | repressing transcription factor binding | 22 | 0.687 | 1.779 | 0.004 | Bbs2/Eif4e/Ctbp1/Bbs1/Ctnnb1/Bcl2/Ttc8/Ddrgk1 |
| GO:0102991 | myristoyl-CoA hydrolase activity | 10 | -0.795 | -1.787 | 0.004 | Acot1/Acot2/Acot13/Acot8/Acot7/Acot9 |
| GO:0016405 | CoA-ligase activity | 16 | -0.712 | -1.805 | 0.005 | Acsbg1/Acsl3/Aacs/Acsl6/Acsf3/Acsl1/Acss1/Acsl5 |
| GO:0140303 | intramembrane lipid transporter activity | 19 | -0.666 | -1.784 | 0.005 | Atp9a/Atp8a2/Abcb1a/Abca7/Abca3/Abca1/Ano4 |
| GO:0044389 | ubiquitin-like protein ligase binding | 181 | 0.390 | 1.467 | 0.006 | Vcl/Ube2j1/Ube2k/Usp9x/Aup1/Ube2j2/Bag1/Psmd1/Pacrg/Ube2d1/Ube2l3/Hspd1/Cbs/Gabarapl1/Vcp/Cul5/Uchl1/Rpl23/Ubash3b/Ube2a/Ube2n/Isg15/Cacybp/Cul9/Tubb5/H13/Bcl2/Hspa1l/Dbt/Tollip/Erbb3/Ubxn7/Traf3/Atxn3/Ddrgk1/Usp7/Jak1/Fbxo7 |
| GO:0050660 | flavin adenine dinucleotide binding | 51 | -0.485 | -1.624 | 0.006 | Kdm1a/Foxred2/Dld/Cyb5r3/Cyb5r1/Acad8/Maob/Mical3/Mto1/Etfa/Dhcr24/Acad9/Sardh/Acadm/Acadsb/Aifm1/Fmo5/Acadl/Txnrd3/Maoa/Acad11/Aifm2/Acox1/Acadvl/Ddo/Dao |
| GO:0015399 | primary active transmembrane transporter activity | 49 | -0.501 | -1.669 | 0.008 | Atp1b3/Atp1a3/Abcb7/Cpox/Atp7b/Abcd4/Abcb8/Atp1a4/Atp1a2/Abcb9/Abca9/Atp1a1/Abcd3/Abcb1a/Abcd2/Abca7/Abcb10/Abca3/Abca1/Abcb6/Atp12a/Abcd1 |
| GO:0031625 | ubiquitin protein ligase binding | 173 | 0.397 | 1.482 | 0.009 | Vcl/Ube2j1/Ube2k/Usp9x/Aup1/Ube2j2/Bag1/Psmd1/Pacrg/Ube2d1/Ube2l3/Hspd1/Cbs/Gabarapl1/Vcp/Cul5/Uchl1/Rpl23/Ubash3b/Ube2a/Ube2n/Isg15/Cacybp/Cul9/Tubb5/H13/Bcl2/Hspa1l/Dbt/Tollip/Erbb3/Ubxn7/Traf3/Atxn3/Usp7/Jak1/Fbxo7 |
| GO:0019905 | syntaxin binding | 68 | 0.479 | 1.575 | 0.011 | Vamp8/Vamp2/Vps11/Napb/Vps18/Vamp3/Vamp7/Snap25/Tmed10/Unc13a/Doc2b |
| GO:0060090 | molecular adaptor activity | 204 | 0.377 | 1.433 | 0.011 | Vamp8/Wwc1/Use1/Vamp2/Mapk8ip2/Myh9/Vps11/Ykt6/Nefl/Napb/Vps18/Rptor/Vamp3/Magi3/Septin4/Nup62/Frs2/Vamp7/Chchd3/Snap25/Vti1a/Pick1/Bnip1/Hip1/Vhl/Gas2l1/Cradd/Ptpn11/Gas6/Map1a |
| GO:0003924 | GTPase activity | 194 | 0.376 | 1.422 | 0.012 | Tubb2b/Rhog/Tuba8/Ran/Gnb4/Tubb2a/Eef1a2/Tubb6/Gnal/Septin4/Cdc42/Tubb1/Eftud2/Tuba4a/Tubb4a/Tbcc/Rap2b/Atl2/Tubg1/Tubb5/Arhgdib/Tubb4b/Rab5c/Rhoq/Entpd7/Atl1/Gnb2/Gpn1/Rras/Arf6/Mfn1/Rab37/Rab9b/Rhoc/Mras/Rab10/Gfm1/Gbp9/Gna11/Gng13/Arhgap5/Septin5/Eef2/Gspt2/Gng12 |
| GO:0050145 | nucleoside monophosphate kinase activity | 11 | -0.735 | -1.695 | 0.012 | Ak5/Cmpk2/Dtymk/Ak1/Mpp6/Ak2/Cmpk1/Ak4 |
| GO:0042626 | ATPase-coupled transmembrane transporter activity | 44 | -0.510 | -1.663 | 0.012 | Atp1b3/Atp1a3/Abcb7/Cpox/Atp7b/Abcd4/Abcb8/Atp1a4/Atp1a2/Abcb9/Abca9/Atp1a1/Abcd3/Abcb1a/Abcd2/Abca7/Abcb10/Abca3/Abca1/Abcb6/Atp12a/Abcd1 |
| GO:0042826 | histone deacetylase binding | 31 | -0.554 | -1.670 | 0.012 | Ywhae/Ywhab/Nudt21/Wdtc1 |
| GO:0015645 | fatty acid ligase activity | 12 | -0.719 | -1.698 | 0.014 | Slc27a4/Acsl4/Acsbg1/Acsl3/Acsl6/Acsf3/Acsl1/Acsl5 |
| GO:0043274 | phospholipase binding | 14 | 0.705 | 1.658 | 0.014 | Nefl/Ptpn11/Snca/Prkcz/Dgkq/Snap91/Arhgap6/Lmnb1 |
| GO:0052689 | carboxylic ester hydrolase activity | 72 | -0.438 | -1.572 | 0.016 | Acot1/Abhd6/Abhd12/H6pd/Acot6/Acot2/Abhd3/Abhd16a/Acot8/Abhd4/Vars2/Acot7/Aars2/Abhd5/Aars/Acot9 |
| GO:0019787 | ubiquitin-like protein transferase activity | 159 | 0.388 | 1.442 | 0.016 | Ube2j1/Ubr3/Ube2k/Zswim2/Ube2j2/Vps11/Ube2m/Uba7/Ube2i/Vps18/Ube2d1/Ube2l3/Klhl9/Atg3/Pja1/Cul5/Ube2a/Ube2n/Rnf167/Ltn1/Sharpin/Hace1/Wwp1/Prpf19/Ubr2/Smurf2/Hecw1/Traf3/Ufl1/Marchf6/Mgrn1/Ube2f/Ube2g2/Ubr1/Herc4/Aktip/Nedd4/Rc3h1/Arih2 |
| GO:0016790 | thiolester hydrolase activity | 34 | -0.519 | -1.600 | 0.017 | Acot1/Abhd12/Acot6/Acot2/Acot13/Abhd16a/Ufsp2/Acot8/Abhd17b/Acot7/Acot9 |
| GO:0016887 | ATPase activity | 191 | -0.325 | -1.341 | 0.018 | Pfn2/Kif3c/Atp1b3/Atp1a3/Dhx15/Ddx17/Helb/Abcb7/Atp5c1/Ide/Cpox/Atp7b/Eif4a1/Spata5/Ddx42/Abcf1/Kif1b/Dync2h1/Atp8b2/Atp13a4/Abcd4/Acsbg1/Acsl3/Abcb8/Atp1a4/Atp1a2/Myo1c/Lonp1/Vps4b/Abce1/Atp13a1/Upf1/Abcf3/Psmc2/Myo1e/Abcb9/Chtf8/Atp9a/Hspa14/Acsl6/Abca9/Afg1l/Acsf3/Atp8a2/Atp2b1/Acsl1/Atp1a1/Abcd3/Vwa8/Atad1/Vps4a/Abcb1a/Abcd2/Abca7/Acsl5/Abcb10/Abca3/Atp2b2/Abca1/Abcb6/Atp12a/Abcd1 |
| GO:0140358 | P-type transmembrane transporter activity | 12 | -0.705 | -1.664 | 0.020 | Atp1b3/Atp1a3/Cpox/Atp7b/Atp1a4/Atp1a2/Atp1a1/Atp12a |
| GO:0004842 | ubiquitin-protein transferase activity | 152 | 0.379 | 1.397 | 0.021 | Ube2j1/Ubr3/Ube2k/Zswim2/Ube2j2/Vps11/Ube2m/Uba7/Ube2i/Vps18/Ube2d1/Ube2l3/Klhl9/Pja1/Cul5/Ube2a/Ube2n/Rnf167/Ltn1/Sharpin/Hace1/Wwp1/Prpf19/Ubr2/Smurf2/Hecw1/Traf3/Marchf6/Mgrn1/Ube2g2/Ubr1/Herc4/Aktip/Nedd4/Rc3h1/Arih2 |
| GO:0005391 | P-type sodium:potassium-exchanging ATPase activity | 10 | -0.729 | -1.638 | 0.021 | Atp1b3/Atp1a3/Cpox/Atp1a4/Atp1a2/Atp1a1/Atp12a |
| GO:0140326 | ATPase-coupled intramembrane lipid transporter activity | 15 | -0.646 | -1.598 | 0.023 | Atp8b2/Atp9a/Atp8a2/Abcb1a/Abca7/Abca3/Abca1 |
| GO:0008289 | lipid binding | 431 | -0.273 | -1.248 | 0.023 | Cd1d1/Snx27/Dgkb/Bin1/Inpp4b/Fnbp1l/Npm1/Gpaa1/Ttpal/Cln6/Chmp3/Snx25/Anxa3/Apoa2/Phb2/Uqcc3/Twf1/Osbpl10/Vps36/Atp1a2/Esyt2/Myo1c/Cln8/Adgrb1/Anxa5/Apod/Ttpa/Pgrmc1/Adap2/Exoc8/Fes/Tspo/Cdk5r2/Myo1e/Dbi/Pitpnm3/Psmb4/Cfl1/Anxa6/Doc2a/C3/Cpne1/Anxa7/Unc13c/Washc2/Osbpl1a/Wipi1/Unc119b/Zfyve1/Stard7/Acbd6/F3/Bscl2/Aldh1a2/Atp1a1/Osbpl9/Apoa4/Cd36/Tulp3/Bdh1/Apoh/Alox5ap/Chmp2a/Appl2/Cyth3/Acox1/Zfyve16/Cpne7/Aida/Acap2/Abca1/Ica1/Akr1b10/Atg2a/Twf2/Vdac2/Acbd5 |
| GO:0000149 | SNARE binding | 98 | 0.418 | 1.449 | 0.024 | Vamp8/Uvrag/Vamp2/Vps11/Napb/Vps18/Vamp3/Vamp7/Vps50/Snapin/Exoc3l4/Snap25/Tmed10/Vti1a/Unc13a/Doc2b/Myo5a/Snca/Exoc3 |
| GO:0042625 | ATPase-coupled ion transmembrane transporter activity | 33 | -0.491 | -1.504 | 0.024 | Atp7b/Atp6v0c/Atp13a4/Atp6ap1/Atp6v1f/Atp1a4/Atp1a2/Anxa5/Atp6v1c1/Atp2b1/Atp1a1/Atp6v0a2/Atp2b3/Atp2b2/Atp12a |
| GO:0004467 | long-chain fatty acid-CoA ligase activity | 10 | -0.721 | -1.619 | 0.025 | Slc27a4/Acsl4/Acsbg1/Acsl3/Acsl6/Acsl1/Acsl5 |
| GO:0005319 | lipid transporter activity | 90 | -0.379 | -1.417 | 0.026 | Slc25a18/Atp8b2/Abcd4/Apoa2/Osbpl10/Ttpa/Atp9a/Abca9/Osbpl1a/Atp8a2/Osbpl9/Abcd3/Apoa4/Cd36/Abcb1a/Abcd2/Abca7/Abca3/Abca1/Abcd1/Ano4 |
| GO:0005201 | extracellular matrix structural constituent | 32 | -0.512 | -1.558 | 0.027 | Col1a2/Col12a1/Vtn/Vwa1 |
| GO:0043548 | phosphatidylinositol 3-kinase binding | 24 | -0.537 | -1.543 | 0.034 | Dab2ip/Ptpn13/Irs2/Dab1/Pik3r2/Pdgfrb/Flt3/Fbxl2/Calm1/Insr/Coro1a/Cbl/Atp1a1/Tyro3 |
| GO:0019829 | ATPase-coupled cation transmembrane transporter activity | 32 | -0.494 | -1.502 | 0.041 | Atp7b/Atp6v0c/Atp13a4/Atp6ap1/Atp6v1f/Atp1a4/Atp1a2/Anxa5/Atp6v1c1/Atp2b1/Atp1a1/Atp6v0a2/Atp2b3/Atp2b2/Atp12a |
| GO:0032182 | ubiquitin-like protein binding | 56 | 0.453 | 1.442 | 0.044 | Aup1/Vps28/Ubap1/Uba2/Nup62/Uchl1/Ube2n/Sharpin/Ddi2/Tollip/Wdr48/Zfand2b/Ubxn7/Fbxo7/Nbr1 |
| GO:0071933 | Arp2/3 complex binding | 11 | -0.677 | -1.561 | 0.047 | Gmfb/Snx9/Ap1ar/Coro1b/Wasf3/Cttn/Wasf2/Wasf1 |
| GO:0005324 | long-chain fatty acid transporter activity | 13 | -0.655 | -1.569 | 0.048 | Abcc1/Slc27a4/Fabp3/Abcd4/Abcd3/Cd36/Abcd2/Abcd1 |
| CC Terms | | | | | | |
| GO:0005777 | peroxisome | 110 | -0.454 | -1.743 | 0.000 | Idh1/Abcd4/Mvd/Prdx5/Acsl3/Far1/Rab8b/Mavs/Dhrs7b/Arf1/Pex12/Acsl6/Acot6/Acsl1/Acot8/Abcd3/Vwa8/Atad1/Acaa1a/Abcd2/Pex13/Acad11/Pex10/Acox1/Hsd17b4/Ddo/Dao/Abcd1/Acbd5 |
| GO:0042579 | microbody | 110 | -0.454 | -1.743 | 0.000 | Idh1/Abcd4/Mvd/Prdx5/Acsl3/Far1/Rab8b/Mavs/Dhrs7b/Arf1/Pex12/Acsl6/Acot6/Acsl1/Acot8/Abcd3/Vwa8/Atad1/Acaa1a/Abcd2/Pex13/Acad11/Pex10/Acox1/Hsd17b4/Ddo/Dao/Abcd1/Acbd5 |
| GO:0005778 | peroxisomal membrane | 40 | -0.606 | -1.938 | 0.000 | Abcd4/Far1/Rab8b/Mavs/Arf1/Pex12/Acsl1/Abcd3/Abcd2/Pex13/Pex10/Acox1/Dao/Abcd1 |
| GO:0031903 | microbody membrane | 40 | -0.606 | -1.938 | 0.000 | Abcd4/Far1/Rab8b/Mavs/Arf1/Pex12/Acsl1/Abcd3/Abcd2/Pex13/Pex10/Acox1/Dao/Abcd1 |
| GO:0031201 | SNARE complex | 41 | 0.626 | 1.872 | 0.001 | Vamp8/Use1/Vamp2/Ykt6/Napb/Vamp3/Vamp7/Snap25/Vti1a/Bnip1/Doc2b |
| GO:0005581 | collagen trimer | 12 | -0.754 | -1.793 | 0.002 | Colec12/C1ql2/C1ql4/Col1a2/Col12a1/C1qa |
| GO:0042588 | zymogen granule | 13 | 0.782 | 1.834 | 0.002 | Vamp8/Vamp2/Hspd1/Tmed10/Scamp1 |
| GO:0031209 | SCAR complex | 10 | -0.765 | -1.706 | 0.004 | Brk1/Wasf3/Cyfip1/Wasf2/Abi1/Wasf1/Abi2 |
| GO:0005766 | primary lysosome | 10 | 0.791 | 1.751 | 0.004 | Vamp8/Vamp7 |
| GO:0042582 | azurophil granule | 10 | 0.791 | 1.751 | 0.004 | Vamp8/Vamp7 |
| GO:0031902 | late endosome membrane | 48 | 0.535 | 1.662 | 0.005 | Vamp8/Wdr91/Tmem9/Vps39/Vamp7/Ubxn6/Vti1a |
| GO:0005773 | vacuole | 359 | 0.337 | 1.375 | 0.006 | Vamp8/Wdr59/Atp6v1g1/Vmp1/Uvrag/Aup1/Vamp2/Thbd/Tmem9/Vps11/Glmp/Vps33a/Gpc3/Hck/Napb/Atg12/Vps16/Vps39/Cst3/Vps18/Rptor/Fyco1/Lmbrd1/Cdc42/Vps33b/Unc93b1/Vamp7/Gabarapl1/Ctbs/Cltc/Fgfr3/Ubxn6/Snapin/Lars/Vps35/Sppl2b/Vti1a/Ctsf/Htt/Slc26a11/Chmp2b/Vma21/Mios/Uba1/App/Borcs5/Bloc1s2/Atp6v0d1/Arsg/Hap1/Wdr48 |
| GO:0000323 | lytic vacuole | 300 | 0.340 | 1.361 | 0.007 | Vamp8/Wdr59/Atp6v1g1/Uvrag/Tmem9/Vps11/Glmp/Vps33a/Gpc3/Hck/Vps16/Vps39/Cst3/Vps18/Rptor/Fyco1/Lmbrd1/Vps33b/Unc93b1/Vamp7/Ctbs/Cltc/Fgfr3/Ubxn6/Snapin/Lars/Vps35/Sppl2b/Ctsf/Slc26a11/Chmp2b/Mios/Uba1/App/Borcs5/Bloc1s2/Atp6v0d1/Arsg/Hap1/Wdr48 |
| GO:0005764 | lysosome | 300 | 0.340 | 1.361 | 0.007 | Vamp8/Wdr59/Atp6v1g1/Uvrag/Tmem9/Vps11/Glmp/Vps33a/Gpc3/Hck/Vps16/Vps39/Cst3/Vps18/Rptor/Fyco1/Lmbrd1/Vps33b/Unc93b1/Vamp7/Ctbs/Cltc/Fgfr3/Ubxn6/Snapin/Lars/Vps35/Sppl2b/Ctsf/Slc26a11/Chmp2b/Mios/Uba1/App/Borcs5/Bloc1s2/Atp6v0d1/Arsg/Hap1/Wdr48 |
| GO:0080008 | Cul4-RING E3 ubiquitin ligase complex | 10 | -0.730 | -1.630 | 0.011 | Ddb1/Wdtc1 |
| GO:0005770 | late endosome | 163 | 0.387 | 1.462 | 0.012 | Vamp8/Uvrag/Wdr91/Vipas39/Tmem9/Vps11/Vps33a/Vps16/Vps39/Vps26b/Cst3/Vps18/Fyco1/Vps33b/Vamp7/Ubxn6/Vps35/Micall1/Vti1a/Ankrd13a/Htt/Chmp2b |
| GO:0030123 | AP-3 adaptor complex | 11 | 0.737 | 1.686 | 0.013 | Vps11/Vps33a/Vps16/Vps39/Vps18 |
| GO:0005765 | lysosomal membrane | 96 | 0.431 | 1.508 | 0.015 | Vamp8/Wdr59/Atp6v1g1/Tmem9/Vps39/Lmbrd1/Vamp7/Ubxn6/Sppl2b/Slc26a11/Mios/Uba1/Borcs5/Atp6v0d1 |
| GO:0098852 | lytic vacuole membrane | 96 | 0.431 | 1.508 | 0.015 | Vamp8/Wdr59/Atp6v1g1/Tmem9/Vps39/Lmbrd1/Vamp7/Ubxn6/Sppl2b/Slc26a11/Mios/Uba1/Borcs5/Atp6v0d1 |
| GO:0010494 | cytoplasmic stress granule | 40 | 0.508 | 1.519 | 0.017 | Eif4e/Ddx6/Rptor/Ybx1/Vcp/Stau2/Ddx19a/Lsm14a/Stau1/Hnrnpk/Rc3h1/Elavl1 |
| GO:0009295 | nucleoid | 33 | 0.534 | 1.534 | 0.022 | Uqcc2/Grsf1/Mterf2/Tfb1m/Hadha/Vdac1/Dbt/Clpx/Elac2 |
| GO:0042645 | mitochondrial nucleoid | 33 | 0.534 | 1.534 | 0.022 | Uqcc2/Grsf1/Mterf2/Tfb1m/Hadha/Vdac1/Dbt/Clpx/Elac2 |
| GO:0045335 | phagocytic vesicle | 76 | 0.446 | 1.491 | 0.023 | Vamp8/Uvrag/Vps26b/Vamp3/Cdc42/Zdhhc5/Unc93b1/Vamp7/Tapbp/H2-K1/Kif16b |
| GO:0005769 | early endosome | 215 | 0.346 | 1.340 | 0.024 | Vamp8/Uvrag/Wdr91/Vipas39/Vps11/Vps28/Usp8/Vps33a/Vps16/Vps26b/Vps18/Vcam1/Hspd1/Vamp3/Zfyve28/Vps33b/Ubxn6/Vps35/Ptpn23/Kif16b/Pick1/Htt/App/Snx5/Atp6v0d1/Rab5c/Myo5a/Bace1/Lrp1/Als2/Ptpn1/Arf6/Slc17a6/Mgrn1/Ehd1/Lamp5/Rap1gap/Commd1/Ocrl |
| GO:0036064 | ciliary basal body | 75 | 0.446 | 1.488 | 0.026 | Bbs2/Ttbk2/Ahi1/Tuba3a/Wdr11/Cys1/Agbl4/Bbs1/Ptpn23/Wdr35/Tubg1/Dync2li1/Tbc1d7/Rilpl1/Cby1/Ttc8/Rpgrip1l/Csnk1d/Cdkl5/Odf2l/Ezr/Bbs9/Ift140/B9d2/Cfap20/Nme7/Mark4/Ttc30a2/Psen2/Rab8a |
| GO:0005774 | vacuolar membrane | 137 | 0.392 | 1.445 | 0.029 | Vamp8/Wdr59/Atp6v1g1/Vmp1/Uvrag/Thbd/Tmem9/Napb/Vps39/Lmbrd1/Vamp7/Gabarapl1/Ubxn6/Sppl2b/Slc26a11/Mios/Uba1/Borcs5/Atp6v0d1 |
| GO:0030136 | clathrin-coated vesicle | 84 | 0.427 | 1.467 | 0.030 | Vamp2/Ap1s1/Vps16/Vamp3/Lmbrd1/Vps33b/Necap1/Cltc/Vti1a/Ap2b1/Htt/Hip1/Scamp1/Lrp1/Myo6/Necap2/Slc18a3/Nrgn/Gad1/Ocrl/Plcg1/Clta/Snap91 |
| GO:0005779 | integral component of peroxisomal membrane | 14 | -0.633 | -1.578 | 0.037 | Pex11b/Far1/Pex12/Pex13/Pex10/Abcd1 |
| GO:0031231 | intrinsic component of peroxisomal membrane | 14 | -0.633 | -1.578 | 0.037 | Pex11b/Far1/Pex12/Pex13/Pex10/Abcd1 |
| GO:0030496 | midbody | 87 | 0.412 | 1.423 | 0.040 | Vamp8/Uvrag/Ran/Usp8/Anln/Klhl9/Nup62/Mical1/Apc2 |
| GO:0005747 | mitochondrial respiratory chain complex I | 39 | 0.499 | 1.488 | 0.044 | Ndufs7/Ndufb5/Ndufv2/Ndufa1/Ndufb11/Ndufb9/Ndufb7/Ndufa11/Ndufb1/Ndufb8/Ndufa2/Ndufb4/Ndufb3 |
| GO:0030964 | NADH dehydrogenase complex | 39 | 0.499 | 1.488 | 0.044 | Ndufs7/Ndufb5/Ndufv2/Ndufa1/Ndufb11/Ndufb9/Ndufb7/Ndufa11/Ndufb1/Ndufb8/Ndufa2/Ndufb4/Ndufb3 |
| GO:0045271 | respiratory chain complex I | 39 | 0.499 | 1.488 | 0.044 | Ndufs7/Ndufb5/Ndufv2/Ndufa1/Ndufb11/Ndufb9/Ndufb7/Ndufa11/Ndufb1/Ndufb8/Ndufa2/Ndufb4/Ndufb3 |
| GO:0010008 | endosome membrane | 180 | 0.340 | 1.301 | 0.045 | Vamp8/Vps37a/Wdr91/Tmem9/Vps28/Usp8/Vps16/Vps39/Ubap1/Fig4/Zfyve28/Vps33b/Arhgap1/Vamp7/Ubxn6/Coro1c/Vps35/Micall1/Sppl2b/Vti1a/Rap2b/Chmp2b/Uba1/Vps37c/Snx5 |
| GO:0035770 | ribonucleoprotein granule | 111 | 0.378 | 1.355 | 0.046 | Ythdc2/Eif4e/Grsf1/Ddx6/Cnot9/Tdrkh/Rptor/Tdrd7/Ybx1/Vcp/Stau2/Lsm4/Ddx19a/Tubb5/Htt/Kif5a/Ddx3y/Edc4/Lsm14a |
| KEGG Terms | | | | | | |
| mmu04146 | Peroxisome | 69 | -0.557 | -1.978 | 0.000 | Idh1/Abcd4/Prdx5/Acsl3/Far1/Pex12/Acsl6/Acsl1/Acot8/Abcd3/Acaa1a/Abcd2/Pex13/Pex10/Acox1/Acsl5/Hsd17b4/Ddo/Dao/Abcd1 |
| mmu02010 | ABC transporters | 28 | -0.619 | -1.780 | 0.001 | Abcd4/Abcb8/Abcb9/Abca9/Abcd3/Abcb1a/Abcd2/Abca7/Abcb10/Abca3/Abca1/Abcb6/Abcd1 |
| mmu03320 | PPAR signaling pathway | 41 | -0.566 | -1.786 | 0.002 | Acadm/Apoa2/Acsbg1/Acsl3/Gykl1/Acadl/Dbi/Acsl6/Acsl1/Cpt1a/Cd36/Acaa1a/Acox1/Acsl5 |
| mmu04925 | Aldosterone synthesis and secretion | 57 | -0.496 | -1.691 | 0.002 | Atp1b3/Atp1a3/Adcy7/Agt/Calm1/Lipe/Cacna1h/Adcy8/Camk4/Cacna1c/Atp1a4/Atp1a2/Adcy5/Camk2a/Atp2b1/Atp1a1/Camk2d/Camk2g/Atp2b3/Atp2b2/Cacna1g/Adcy6 |
| mmu05012 | Parkinson disease | 185 | 0.406 | 1.539 | 0.003 | Ube2j1/Tubb2b/Tuba8/Ube2j2/Vdac3/Tubb2a/Cox6b1/Uqcrh/Uba7/Slc39a10/Psmd1/Tubb6/Ndufs7/Ube2l3/Ndufb5/Tuba3a/Ndufv2/Ndufa1/Gnal/Ndufb11/Tubb1/Slc39a7/Uchl1/Tuba4a/Ndufb9/Slc39a12/Tubb4a/Tubb5/Vdac1/Ndufb7/Tubb4b/Uba1/Kif5a/Ndufa11/Bcl2l1/Uqcrc2/Snca/Ndufb8/Cox7a2/Psmd2/Ndufa2/Mfn1/Ube2g2/Cox5a/Ndufb4/Ndufb3/Slc39a6/Plcg1/Prkacb/Septin5 |
| mmu04130 | SNARE interactions in vesicular transport | 27 | 0.666 | 1.800 | 0.003 | Vamp8/Use1/Vamp2/Ykt6/Vamp3/Vamp7/Vti1a/Bnip1 |
| mmu00071 | Fatty acid degradation | 36 | -0.572 | -1.752 | 0.003 | Acadm/Acsbg1/Acadsb/Acsl3/Aldh9a1/Acadl/Acsl6/Acat1/Acsl1/Cpt1a/Acaa1a/Acox1/Acsl5/Acadvl/Aldh1b1 |
| mmu04145 | Phagosome | 87 | 0.453 | 1.552 | 0.012 | Atp6v1g1/Tubb2b/Tuba8/Tubb2a/Fcgr1/Calr4/Tubb6/Tuba3a/Vamp3/Itgb5/Tubb1/Canx/Tuba4a/Tubb4a/H2-K1/Itga5/Tubb5/Tubb4b/Atp6v0d1/Rab5c/Itgb1/Dync1h1/Itgam/Tap2 |
| mmu04972 | Pancreatic secretion | 50 | -0.477 | -1.579 | 0.012 | Adcy9/Gnaq/Chrm3/Cck/Rhoa/Atp1b3/Atp1a3/Adcy7/Adcy8/Prss1/Atp1a4/Atp1a2/Adcy5/Atp2b1/Atp1a1/Atp2b3/Atp2b2/2210010C04Rik/Adcy6 |
| mmu04976 | Bile secretion | 37 | -0.497 | -1.533 | 0.016 | Hmgcr/Prkaca/Abcc4/Adcy1/Kcnn2/Adcy9/Abcg2/Nceh1/Atp1b3/Atp1a3/Adcy7/Aqp1/Adcy8/Ephx1/Atp1a4/Atp1a2/Adcy5/Ugt1a7c/Atp1a1/Abcb1a/Adcy6 |
| mmu01212 | Fatty acid metabolism | 50 | -0.455 | -1.508 | 0.019 | Acaca/Acadm/Acsbg1/Acadsb/Acsl3/Acadl/Acsl6/Acat1/Acsf3/Acsl1/Cpt1a/Acaa1a/Acox1/Acsl5/Acadvl/Hsd17b4 |
| mmu04971 | Gastric acid secretion | 52 | -0.451 | -1.506 | 0.021 | Adcy9/Sst/Gnaq/Chrm3/Atp1b3/Atp1a3/Adcy7/Calm1/Adcy8/Atp1a4/Atp1a2/Adcy5/Camk2a/Gnai1/Atp1a1/Actb/Camk2d/Camk2g/Adcy6 |
| mmu05022 | Pathways of neurodegeneration - multiple diseases | 309 | 0.330 | 1.316 | 0.026 | Ube2j1/Tubb2b/Tuba8/Ube2j2/Vdac3/Tubb2a/Cox6b1/Uqcrh/Nefl/Uba7/Wdr41/Psmd1/Tubb6/Ryr2/Ndufs7/Fig4/Ube2l3/Ndufb5/Tuba3a/Ndufv2/Ndufa1/Gria3/Ndufb11/Fzd3/Tubb1/Vcp/Uchl1/Actr1b/Tuba4a/Ndufb9/Apc2/Map2k3/Fzd2/Tubb4a/Ctnnb1/Tubb5/Htt/Chmp2b/Hip1/Vdac1/Ndufb7/Tubb4b/Uba1/Kif5a/Bcl2/Ndufa11/App/Hap1/Bcl2l1/Uqcrc2/Snca/Csnk2b/Ndufb8/Cox7a2/Grin1/Psmd2/Als2/Mapk14/Ndufa2/Atxn3/Mfn1 |
| mmu00040 | Pentose and glucuronate interconversions | 11 | -0.694 | -1.581 | 0.027 | Ugt1a7c/Akr1a1/Akr1b10 |
| mmu01040 | Biosynthesis of unsaturated fatty acids | 21 | -0.556 | -1.507 | 0.027 | Acot1/Acot2/Acaa1a/Acox1/Acot7/Hsd17b4 |
| mmu05020 | Prion disease | 180 | 0.362 | 1.373 | 0.028 | Tubb2b/Tuba8/Vdac3/Tubb2a/Cox6b1/Uqcrh/Psmd1/Tubb6/Ryr2/Ndufs7/Ndufb5/Tuba3a/Ndufv2/Ndufa1/Ndufb11/Tubb1/Tuba4a/Ndufb9/Tubb4a/Ncam1/Tubb5/Vdac1/Ndufb7/Tubb4b/Kif5a/Ndufa11/Hspa1l/Uqcrc2/Csnk2b/Ndufb8/Cox7a2/Grin1/Psmd2/Mapk14/Ndufa2 |
| mmu04120 | Ubiquitin mediated proteolysis | 78 | 0.439 | 1.481 | 0.030 | Ube2j1/Ube2k/Ube2j2/Ube2m/Uba7/Ube2i/Cdc23/Uba2/Ube2d1/Ube2l3/Klhl9/Uba3/Cul5/Ube2a/Ube2n/Wwp1/Uba1/Vhl/Prpf19/Anapc13/Smurf2/Mgrn1/Ube2f/Ube2g2/Herc4 |
| mmu04261 | Adrenergic signaling in cardiomyocytes | 92 | -0.363 | -1.353 | 0.031 | Cacng3/Akt3/Atp1b3/Atp1a3/Adcy7/Ppp1cb/Akt1/Agt/Calm1/Cacnb4/Adcy8/Cacna1c/Ppp2r2b/Atp1a4/Ppp2r2c/Atp1a2/Adcy5/Camk2a/Gnai1/Atp2b1/Atp1a1/Camk2d/Camk2g/Atp2b3/Adra1a/Atp2b2/Adcy6 |
| mmu04114 | Oocyte meiosis | 67 | -0.406 | -1.432 | 0.032 | Adcy7/Ppp1cb/Calm1/Anapc4/Btrc/Adcy8/Ywhaz/Adcy5/Ywhae/Camk2a/Cpeb3/Camk2d/Camk2g/Anapc2/Ywhaq/Anapc1/Ywhab/Adcy6 |
| mmu04974 | Protein digestion and absorption | 30 | -0.528 | -1.553 | 0.034 | Slc7a8/Atp1b3/Atp1a3/Slc1a5/Prss1/Atp1a4/Atp1a2/Atp1a1/Col1a2/Col12a1/2210010C04Rik |
| mmu01232 | Nucleotide metabolism | 53 | -0.436 | -1.463 | 0.034 | Impdh2/Aprt/Tk2/Pnp/Cda/Hddc2/Nt5c3b/Ampd2/Gmpr2/Cant1/Ak5/Adsl/Cmpk2/Dtymk/Nt5c1a/Ak1/Adss/Entpd4/Entpd3/Adssl1/Itpa/Ak2/Ada/Cmpk1/Ampd3/Ak4 |
| mmu05132 | Salmonella infection | 169 | 0.356 | 1.337 | 0.038 | Tubb2b/Rhog/Tuba8/Tubb2a/Vps11/Vps33a/Vps16/Vps39/Tubb6/Vps18/Actr3b/Gcc2/Fyco1/Tuba3a/Cdc42/Tubb1/Actr1b/Tuba4a/Map2k3/Tubb4a/Ctnnb1/Tubb5/Flna/Dync2li1/Tubb4b/Kif5a/Bcl2/Rab5c/S100a10/Dync1h1/Rras/Mapk14/Arf6 |
| mmu04022 | cGMP-PKG signaling pathway | 84 | -0.369 | -1.361 | 0.040 | Slc8a1/Adcy9/Gucy1b1/Gnaq/Akt3/Rhoa/Atp1b3/Atp1a3/Adcy7/Ppp1cb/Akt1/Rock2/Ppif/Calm1/Insr/Adcy8/Cacna1c/Atp1a4/Atp1a2/Adcy5/Gnai1/Atp2b1/Atp1a1/Atp2b3/Adra1a/Atp2b2/Vdac2/Adcy6 |
| mmu04024 | cAMP signaling pathway | 122 | -0.338 | -1.317 | 0.042 | Akt3/Grin3a/Rhoa/Atp1b3/Atp1a3/Adcy7/Ppp1cb/Akt1/Rock2/Calm1/Lipe/Hcn4/Adcy8/Camk4/Cacna1c/Gria1/Chrm2/Atp1a4/Atp1a2/Adcy5/Afdn/Camk2a/Gnai1/Atp2b1/Atp1a1/Adcyap1r1/Camk2d/Camk2g/Atp2b3/Adcy10/Acox1/Npy/Atp2b2/Adcy6 |
| mmu00230 | Purine metabolism | 86 | -0.365 | -1.354 | 0.046 | Impdh2/Aprt/Adcy1/Prune1/Ppat/Pnp/Adcy9/Gucy1b1/Hddc2/Paics/Ampd2/Gmpr2/Cant1/Ak5/Adsl/Adcy7/Atic/Nt5c1a/Ak1/Adcy8/Adss/Entpd4/Adcy5/Pfas/Nudt2/Entpd3/Adssl1/Itpa/Ak2/Ada/Adcy10/Ampd3/Ak4/Adcy6 |
