# Supplementary material for: The ultrastructural and proteomic analysis of mitochondria‐associated endoplasmic reticulum membrane in the midbrain of a Parkinson's disease mouse model
Source: Aging Cell. 2024 Nov 29;24(4):e14436. doi: 10.1111/acel.14436 (PMC11984660; doi:10.1111/acel.14436)
Supplement: Supplementary file 18 — Table S12. Summary of enriched BP terms in MAM proteomics. [file ACEL-24-e14436-s011.docx]

### Supplementary Table 12 Summary of enriched BP terms in MAM proteomics

| Primary category | Secondary category | Tertiary category ID | Tertiary category | Set size | Enrichment score | NES | p value | Core enrichment genes |
| --- | --- | --- | --- | --- | --- | --- | --- | --- |
| organelle organization | actin filament induced organelles movement | GO:0008154 | actin polymerization or depolymerization | 144 | -0.402 | -1.621 | 0.001 | Pycard/Gmfb/Gba2/Snx9/Mical3/Brk1/Pfn2/Rhoa/Mtpn/Arhgap35/Capn1/Ctnna2/Ap1ar/Wdr1/Evl/Coro1b/Myadm/Spta1/Wasf3/Bin1/Bbs4/Coro1a/Cttn/Dstn/Enah/Twf1/Capza1/Myo1c/Arpc1b/Plek/Carmil1/Cyfip1/Arf1/Cfl1/Wasl/Arpc1a/Add2/Washc1/Arfgef1/Bag4/Arpc2/Aif1/Wasf2/Abi1/Twf2/Wasf1/Abi2 |
| organelle organization | actin filament induced organelles movement | GO:0008064 | regulation of actin polymerization or depolymerization | 131 | -0.410 | -1.646 | 0.001 | Pycard/Gmfb/Gba2/Snx9/Brk1/Pfn2/Rhoa/Mtpn/Arhgap35/Capn1/Ctnna2/Ap1ar/Wdr1/Evl/Coro1b/Myadm/Spta1/Wasf3/Bin1/Bbs4/Coro1a/Cttn/Dstn/Twf1/Capza1/Myo1c/Arpc1b/Plek/Carmil1/Cyfip1/Arf1/Cfl1/Wasl/Arpc1a/Add2/Washc1/Arfgef1/Bag4/Arpc2/Wasf2/Twf2/Wasf1/Abi2 |
| organelle organization | actin filament induced organelles movement | GO:0030832 | regulation of actin filament length | 131 | -0.410 | -1.646 | 0.001 | Pycard/Gmfb/Gba2/Snx9/Brk1/Pfn2/Rhoa/Mtpn/Arhgap35/Capn1/Ctnna2/Ap1ar/Wdr1/Evl/Coro1b/Myadm/Spta1/Wasf3/Bin1/Bbs4/Coro1a/Cttn/Dstn/Twf1/Capza1/Myo1c/Arpc1b/Plek/Carmil1/Cyfip1/Arf1/Cfl1/Wasl/Arpc1a/Add2/Washc1/Arfgef1/Bag4/Arpc2/Wasf2/Twf2/Wasf1/Abi2 |
| organelle organization | actin filament induced organelles movement | GO:0030833 | regulation of actin filament polymerization | 118 | -0.415 | -1.637 | 0.001 | Pycard/Gmfb/Gba2/Snx9/Brk1/Pfn2/Rhoa/Mtpn/Capn1/Ctnna2/Ap1ar/Evl/Coro1b/Myadm/Spta1/Wasf3/Bin1/Bbs4/Coro1a/Cttn/Twf1/Capza1/Myo1c/Arpc1b/Carmil1/Cyfip1/Arf1/Cfl1/Wasl/Arpc1a/Add2/Washc1/Arfgef1/Bag4/Arpc2/Wasf2/Twf2/Wasf1/Abi2 |
| organelle organization | actin filament induced organelles movement | GO:0030838 | positive regulation of actin filament polymerization | 70 | -0.470 | -1.690 | 0.002 | Pycard/Gmfb/Snx9/Brk1/Pfn2/Rhoa/Ctnna2/Ap1ar/Evl/Coro1b/Wasf3/Bin1/Coro1a/Cttn/Myo1c/Arpc1b/Carmil1/Cyfip1/Arf1/Wasl/Arpc1a/Washc1/Bag4/Arpc2/Wasf2/Wasf1/Abi2 |
| organelle organization | actin filament induced organelles movement | GO:0030041 | actin filament polymerization | 127 | -0.395 | -1.582 | 0.004 | Pycard/Gmfb/Gba2/Snx9/Brk1/Pfn2/Rhoa/Mtpn/Capn1/Ctnna2/Ap1ar/Evl/Coro1b/Myadm/Spta1/Wasf3/Bin1/Bbs4/Coro1a/Cttn/Twf1/Capza1/Myo1c/Arpc1b/Carmil1/Cyfip1/Arf1/Cfl1/Wasl/Arpc1a/Add2/Washc1/Arfgef1/Bag4/Arpc2/Aif1/Wasf2/Twf2/Wasf1/Abi2 |
| organelle organization | actin filament induced organelles movement | GO:0051125 | regulation of actin nucleation | 22 | -0.594 | -1.619 | 0.005 | Gmfb/Brk1/Ctnna2/Ap1ar/Coro1b/Wasf3/Coro1a/Cyfip1/Arf1/Wasl/Wasf2/Wasf1/Abi2 |
| organelle organization | actin filament induced organelles movement | GO:0045010 | actin nucleation | 38 | -0.517 | -1.599 | 0.008 | Gmfb/Brk1/Ctnna2/Ap1ar/Evl/Coro1b/Wasf3/Coro1a/Arpc1b/Cyfip1/Arf1/Wasl/Arpc1a/Washc1/Arpc2/Wasf2/Wasf1/Abi2 |
| organelle organization | actin filament induced organelles movement | GO:0034314 | Arp2/3 complex-mediated actin nucleation | 31 | -0.532 | -1.559 | 0.010 | Gmfb/Brk1/Ctnna2/Ap1ar/Coro1b/Wasf3/Arpc1b/Cyfip1/Arf1/Wasl/Arpc1a/Washc1/Arpc2/Wasf2/Wasf1/Abi2 |
| organelle organization | actin filament induced organelles movement | GO:0051127 | positive regulation of actin nucleation | 10 | -0.735 | -1.664 | 0.010 | Brk1/Wasf3/Cyfip1/Wasl/Wasf2/Wasf1/Abi2 |
| organelle organization | actin filament induced organelles movement | GO:0034315 | regulation of Arp2/3 complex-mediated actin nucleation | 18 | -0.622 | -1.610 | 0.010 | Gmfb/Brk1/Ctnna2/Ap1ar/Coro1b/Wasf3/Cyfip1/Arf1/Wasl/Wasf2/Wasf1/Abi2 |
| organelle organization | mitochondrial morphology | GO:0070131 | positive regulation of mitochondrial translation | 11 | 0.807 | 1.802 | 0.004 | Uqcc2/Mief1/Rpusd3 |
| organelle organization | mitochondrial morphology | GO:0032543 | mitochondrial translation | 60 | 0.508 | 1.624 | 0.004 | Uqcc2/Mrps18b/Sars2/Mief1/Mrpl57/Mrpl43/Mtrf1l/Rpusd3/Rars2 |
| organelle organization | mitochondrial morphology | GO:0070129 | regulation of mitochondrial translation | 15 | 0.754 | 1.822 | 0.003 | Uqcc2/Mief1/Rpusd3 |
| organelle organization | mitochondrial morphology | GO:0140053 | mitochondrial gene expression | 79 | 0.460 | 1.569 | 0.013 | Uqcc2/Mrps18b/Sars2/Prkaa1/Mief1/Mterf2/Mrpl57/Mrpl43/Mtrf1l/Tfb1m/Rpusd3/Rars2 |
| organelle organization | mitochondrial morphology | GO:0033108 | mitochondrial respiratory chain complex assembly | 66 | 0.452 | 1.471 | 0.029 | Uqcc2/Ndufs7/Ndufb5/Ndufa1/Ndufb11/Ndufb9/Samm50/Cox20/Ndufb7/Ndufa11/Ndufb1/Ndufb8/Ndufa2/Ndufaf3 |
| organelle organization | mitochondrial morphology | GO:0062125 | regulation of mitochondrial gene expression | 18 | 0.691 | 1.753 | 0.006 | Uqcc2/Prkaa1/Mief1/Rpusd3 |
| organelle organization | peroxisome | GO:0007031 | peroxisome organization | 29 | -0.638 | -1.843 | 0.001 | Pex11b/Abcd4/Rab8b/Mavs/Pex12/Acot8/Abcd3/Abcd2/Pex13/Pex10/Acox1/Abcd1 |
| organelle organization | peroxisome | GO:0015919 | peroxisomal membrane transport | 18 | -0.688 | -1.781 | 0.002 | Abcd4/Rab8b/Pex12/Abcd3/Abcd2/Pex13/Pex10/Abcd1 |
| organelle organization | peroxisome | GO:0043574 | peroxisomal transport | 21 | -0.646 | -1.745 | 0.004 | Abcd4/Rab8b/Pex12/Abcd3/Abcd2/Pex13/Pex10/Abcd1 |
|  |  |  |  |  |  |  |  |  |
| organelle organization | autophagosome | GO:0097352 | autophagosome maturation | 26 | 0.666 | 1.807 | 0.002 | Vamp8/Uvrag/Vps33a/Vps16/Fyco1/Vcp/Snapin |
| organelle organization | autophagosome | GO:0016236 | macroautophagy | 146 | 0.375 | 1.385 | 0.039 | Vamp8/Vmp1/Uvrag/Aup1/Bnip3/Vps33a/Wdr45/Atg12/Vps16/Fyco1/Atg3/Gabarapl1/Vcp/Ubxn6/Snapin/Usp30/Uba5 |
| organelle organization | endosome | GO:0007032 | endosome organization | 37 | 0.584 | 1.726 | 0.002 | Tmem9/Vps11/Usp8/Washc4/Vps18/Hook3/Arfgef2/Vps33b/Coro1c/Chmp2b/Rab5c/Tmcc1/Als2 |
| organelle organization | endosome | GO:0008333 | endosome to lysosome transport | 37 | 0.531 | 1.567 | 0.011 | Vps11/Vps33a/Vps16/Vps39/Vps18/Hook3/Vamp7/Vcp/Ubxn6/Snapin |
| organelle organization | lysosome | GO:0032418 | lysosome localization | 51 | 0.528 | 1.644 | 0.008 | Vamp8/Myh9/Vps33a/Vps33b/Vamp7/Snapin/Fgr/Borcs5/Bloc1s2 |
| organelle organization | fusion | GO:0048284 | organelle fusion | 112 | 0.486 | 1.739 | 0.001 | Vamp8/Vcpip1/Uvrag/Vamp2/Vps11/Bnip3/Vps16/Vps39/Vps18/Vav3/Vamp3/Vamp7/Chchd3/Snapin/Usp30/Eno3/Snap25/Vti1a/Bnip1 |
| organelle organization | fusion | GO:0061025 | membrane fusion | 103 | 0.470 | 1.660 | 0.002 | Vamp8/Vcpip1/Uvrag/Vamp2/Vps11/Vps39/Vps18/Vav3/Vamp3/Vps33b/Vamp7/Snapin/Snap25/Folr1/Vti1a/Hace1/Bnip1 |
| organelle organization | fusion | GO:0090174 | organelle membrane fusion | 85 | 0.490 | 1.693 | 0.002 | Vamp8/Vcpip1/Uvrag/Vamp2/Vps11/Vps39/Vps18/Vav3/Vamp3/Vamp7/Snapin/Snap25/Vti1a/Bnip1 |
| transport | vesicle transport | GO:0035493 | SNARE complex assembly | 17 | 0.806 | 2.018 | 0.000 | Vamp8/Uvrag/Vamp2/Vps11/Vps18/Vamp3/Vamp7/Snap25 |
| transport | vesicle transport | GO:0006906 | vesicle fusion | 80 | 0.479 | 1.634 | 0.005 | Vamp8/Uvrag/Vamp2/Vps11/Vps39/Vps18/Vav3/Vamp3/Vamp7/Snapin/Snap25/Vti1a |
| transport | vesicle transport | GO:0097091 | synaptic vesicle clustering | 18 | 0.615 | 1.560 | 0.048 | Pclo/Nlgn1/Nrxn1/Ctnnb1/Nlgn2/Bcl2l1/Pten/Snap91/Syn2/Syn3 |
| transport | vesicle transport | GO:0099637 | neurotransmitter receptor transport | 57 | 0.440 | 1.400 | 0.054 | Vamp2/Hpca/Usp46/Efnb2/Cacng4/Grip2/Vps35/Snap25/Pick1/Ap2b1/Kif5a/Clstn1/Hap1 |
| transport | lipid transport | GO:0032370 | positive regulation of lipid transport | 57 | -0.477 | -1.657 | 0.006 | Dbi/Mif/Acsl6/Dennd5b/Atp8a2/Acsl1/Washc1/Abca7/Acsl5/Abca3/Abca1/Abat |
| transport | lipid transport | GO:1905954 | positive regulation of lipid localization | 66 | -0.448 | -1.597 | 0.006 | Dbi/C3/Mif/Acsl6/Dennd5b/Atp8a2/Acsl1/Cd36/Washc1/Abca7/Acsl5/Abca3/Abca1/Abat |
| transport | lipid transport | GO:0015909 | long-chain fatty acid transport | 30 | -0.561 | -1.632 | 0.006 | Abcd4/Acsl3/Mif/Acsl6/Acsl1/Abcd3/Cd36/Abcd2/Acsl5/Abcd1 |
| transport | lipid transport | GO:0032365 | intracellular lipid transport | 22 | -0.597 | -1.628 | 0.005 | Abcd4/Abcd3/Vps4a/Abcd2/Abca1/Abcd1 |
| transport | lipid transport | GO:0006869 | lipid transport | 221 | -0.311 | -1.328 | 0.015 | Osbpl10/Esyt2/Cln8/Apod/Ttpa/Ugcg/Vps4b/Apobr/Tspo/Dbi/Atp9a/Mif/Acsl6/Dennd5b/Abca9/Osbpl1a/Atp8a2/Acsl1/Osbpl9/Abcd3/Apoa4/Cd36/Xkr7/Ttc39b/Vps4a/Apoh/Abcb1a/Abcd2/Washc1/Abca7/Acsl5/Abca3/Abca1/Abat/Abcd1/Ano4 |
| transport | lipid transport | GO:2001138 | regulation of phospholipid transport | 10 | -0.701 | -1.588 | 0.021 | Dbi/Atp8a2/Abca7/Abca3 |
| transport | lipid transport | GO:0045332 | phospholipid translocation | 24 | -0.552 | -1.557 | 0.021 | Atp9a/Atp8a2/Xkr7/Abcb1a/Abca7/Abca1/Ano4 |
| transport | protein transport | GO:0051223 | regulation of protein transport | 331 | 0.343 | 1.385 | 0.009 | Vamp8/Uqcc2/Ube2j1/Svip/Ran/Vamp2/Rhbdd3/Prkaa1/Vps28/Ttc21b/Glrx/Hpca/Rptor/Gcc2/Brsk2/Ahi1/Nup62/Cdc42/Vamp7/Cacna1e/Pard6a/Usp46/Efnb2/Pam/Glul/Nrxn1/Vps35/Ptpn23/Ripor1/Tmed10/Ubac2/Pick1/Flna/Nlgn2/Doc2b/App/Hspa1l/Oxct1/Atpif1/Arfip1/Hap1/Camk1/Nadk/Sirt3/Ptpn11/Gas6/Hadh/Itgam/C2cd5/Lrp1/Jagn1/Mapk14/Ptpn1/Arf6/Prkcz/Ttn/Ube2g2/Crhr2/Erp29/Commd1/Ipo5/Atg7/Pkia/Gpld1/Nedd4/Os9/Abcg1/Nf1 |
| transport | protein transport | GO:0090087 | regulation of peptide transport | 346 | 0.333 | 1.354 | 0.011 | Vamp8/Uqcc2/Ube2j1/Svip/Ran/Vamp2/Rhbdd3/Prkaa1/Vps28/Ttc21b/Glrx/Hpca/Rptor/Gcc2/Brsk2/Ahi1/Nup62/Cdc42/Vamp7/Cacna1e/Pard6a/Usp46/Efnb2/Pam/Glul/Nrxn1/Vps35/Ptpn23/Ripor1/Tmed10/Ubac2/Pick1/Flna/Nlgn2/Doc2b/App/Hspa1l/Oxct1/Atpif1/Arfip1/Hap1/Camk1/Nadk/Sirt3/Ptpn11/Gas6/Hadh/Itgam/C2cd5/Lrp1/Jagn1/Mapk14/Ptpn1/Arf6/Prkcz/Ttn/Ube2g2/Crhr2/Erp29/Commd1/Ipo5/Atg7/Pkia/Gpld1/Nedd4/Os9/Abcg1/Nf1/Ezr/Itsn1/Snap91/Tiam1 |
| transport | protein transport | GO:0070201 | regulation of establishment of protein localization | 350 | 0.334 | 1.354 | 0.013 | Vamp8/Uqcc2/Ube2j1/Svip/Ran/Vamp2/Rhbdd3/Prkaa1/Vps28/Ttc21b/Glrx/Hpca/Rptor/Gcc2/Brsk2/Ahi1/Nup62/Cdc42/Vamp7/Cacna1e/Pard6a/Usp46/Efnb2/Pam/Glul/Nrxn1/Vps35/Ptpn23/Snap25/Ripor1/Tmed10/Ubac2/Pick1/Flna/Nlgn2/Doc2b/App/Hspa1l/Oxct1/Atpif1/Arfip1/Hap1/Camk1/Nadk/Sirt3/Ptpn11/Gas6/Hadh/Itgam/C2cd5/Lrp1/Jagn1/Mapk14/Ptpn1/Arf6/Prkcz/Ttn/Ube2g2/Crhr2/Cep295/Erp29/Commd1/Ipo5/Atg7/Pkia/Gpld1/Nedd4/Os9/Abcg1/Nf1 |
| transport | protein transport | GO:1903827 | regulation of cellular protein localization | 369 | 0.330 | 1.347 | 0.016 | Vamp8/Ube2j1/Rhog/Vcpip1/Svip/Dbn1/Ran/Vamp2/Prkaa1/Vps28/Gpc3/Ttc21b/Hpca/Ttbk2/Gcc2/Nup62/Cdc42/Zdhhc5/Vamp7/Vcp/Cltc/Pard6a/Usp46/Efnb2/Grip2/Glul/Nrxn1/Dpp6/Vps35/Ripor1/Ctnnb1/Ubac2/Pick1/Ankrd13a/Gpc5/Flna/Htt/Nlgn2/Limk2/App/Hspa1l/Vhl/Atpif1/Itga3/Hap1/Rhoq/Camk1/Bcl2l1/Itgb1/Rer1/Myo5a/Cnpy4/Ptpn11/Gas6/Map1a/Itgam/C2cd5/Synj2bp/Lrp1/Mapk14/Ptpn1/Arf6/Prkcz/Gpd1l/Ddrgk1/Epb41l2 |
| transport | protein transport | GO:0050708 | regulation of protein secretion | 152 | 0.387 | 1.440 | 0.014 | Vamp8/Uqcc2/Rhbdd3/Glrx/Rptor/Brsk2/Ahi1/Cacna1e/Pard6a/Pam/Glul/Nrxn1/Vps35/Ptpn23/Tmed10/Pick1/Nlgn2/Doc2b/Oxct1/Arfip1/Nadk/Sirt3/Ptpn11/Hadh/Lrp1/Jagn1/Arf6/Ttn/Crhr2/Erp29/Atg7/Gpld1/Abcg1/Ezr/Tiam1 |
| transport | protein transport | GO:0032527 | protein exit from endoplasmic reticulum | 34 | 0.538 | 1.560 | 0.012 | Ube2j1/Svip/Aup1/Gcc2/Vcp/Ubac2/H13/Ufd1 |
| signaling | immune responses | GO:0002696 | positive regulation of leukocyte activation | 107 | 0.454 | 1.613 | 0.004 | Vamp8/Adk/Slc39a10/Bloc1s3/Vcam1/Vav3/Hspd1/Cd276/Vamp7/Efnb2/Igkc/Hes1/Fgr/Pcid2/Bcl2/Atp11c/Dpp4/Gas6/Itgam/Mdk/Gpam/Prkcz/Ighm/Thy1/Efnb3/Pla2g4a |
| signaling | immune responses | GO:0031349 | positive regulation of defense response | 81 | 0.478 | 1.634 | 0.004 | Vamp8/Ube2k/Vav1/Fcgr1/Hspd1/Vamp7 |
| signaling | immune responses | GO:0033005 | positive regulation of mast cell activation | 12 | 0.753 | 1.717 | 0.008 | Vamp8/Vamp7/Fgr |
| signaling | immune responses | GO:0050729 | positive regulation of inflammatory response | 47 | 0.508 | 1.551 | 0.014 | Vamp8/Fcgr1/Hspd1/Vamp7 |
| signaling | immune responses | GO:0033008 | positive regulation of mast cell activation involved in immune response | 11 | 0.761 | 1.697 | 0.014 | Vamp8/Vamp7/Fgr |
| signaling | immune responses | GO:0043306 | positive regulation of mast cell degranulation | 11 | 0.761 | 1.697 | 0.014 | Vamp8/Vamp7/Fgr |
| signaling | immune responses | GO:0002703 | regulation of leukocyte mediated immunity | 70 | 0.455 | 1.503 | 0.030 | Vamp8/Vav1/Fcgr1/Hspd1/Vamp7/H2-K1/Fgr/Dpp4/Susd4/Itgam/Tap2/Prkcz/Cd84 |
| signaling | immune responses | GO:0009615 | response to virus | 84 | 0.425 | 1.463 | 0.031 | Vamp8/Aup1/Tspan6/Bnip3/Unc93b1/Agbl4/Isg15/Zmpste24/Ufd1/Bcl2/Crebbp/Bcl2l1/Lsm14a/Traf3/Gpam/Mapk14/Ppm1b/Itgb8/Atg7 |
| signaling | immune responses | GO:0043302 | positive regulation of leukocyte degranulation | 15 | 0.658 | 1.590 | 0.031 | Vamp8/Vamp7/Fgr |
| signaling | immune responses | GO:0140546 | defense response to symbiont | 65 | 0.449 | 1.459 | 0.031 | Vamp8/Tspan6/Bnip3/Unc93b1/Agbl4/Isg15/Zmpste24/Ufd1/Bcl2/Bcl2l1/Lsm14a/Traf3/Gpam |
| signaling | immune responses | GO:0050778 | positive regulation of immune response | 166 | 0.366 | 1.379 | 0.034 | Vamp8/Ube2k/Usp9x/Vav1/Fcgr1/Slc39a10/Vav3/Hspd1/Cd276/Dgkz/Vamp7/Usp46/Ube2n/Igkc/H2-K1/Fgr/Bcl2/Susd4/Lsm14a/Eif2b3/Itgam/Tap2/Bcar1/Prkcz/Ighm/Thy1/Pla2g4a/Gpld1/Plcg1/Rc3h1/Ezr/Usp12/Khdrbs1/Nckap1l/Lpxn/Ptpn6/Hspa8/Fcgr2b/Gab2/C4b/Serping1/Pde4b |
| signaling | behavioral response | GO:0001662 | behavioral fear response | 41 | 0.501 | 1.501 | 0.023 | Mapk8ip2/Vdac3/Eif4e/Cacna1e/Usp46/Brinp1/Vdac1/Bcl2/Dpp4/Rps6kb1/Eif4g1/Mdk/Als2 |
| signaling | behavioral response | GO:0002209 | behavioral defense response | 41 | 0.501 | 1.501 | 0.023 | Mapk8ip2/Vdac3/Eif4e/Cacna1e/Usp46/Brinp1/Vdac1/Bcl2/Dpp4/Rps6kb1/Eif4g1/Mdk/Als2 |
| signaling | behavioral response | GO:0042596 | fear response | 44 | 0.484 | 1.453 | 0.043 | Mapk8ip2/Vdac3/Eif4e/Cacna1e/Usp46/Brinp1/Vdac1/Bcl2/Dpp4/Rps6kb1/Eif4g1/Mdk/Als2 |
| signaling | response to caffeine | GO:0031000 | response to caffeine | 10 | 0.750 | 1.617 | 0.010 | Prkaa1/Tmem38b/Ryr2/Gnal |
| signaling | apoptosis | GO:0043653 | mitochondrial fragmentation involved in apoptotic process | 10 | 0.705 | 1.521 | 0.053 | Bnip3/Atg3/Vps35/Ccar2 |
| signaling | apoptosis | GO:1902041 | regulation of extrinsic apoptotic signaling pathway via death domain receptors | 23 | 0.641 | 1.706 | 0.011 | Zswim2/Faim/Bmpr1b/Faim2/Bcl2l1/Pten/Raf1 |
| signaling | apoptosis | GO:0008625 | extrinsic apoptotic signaling pathway via death domain receptors | 38 | 0.479 | 1.427 | 0.053 | Zswim2/Faim/Bmpr1b/Faim2/Bcl2/Bloc1s2/Bcl2l1/Pten/Nf1/Raf1 |
| signaling | necrosis | GO:0097300 | programmed necrotic cell death | 24 | -0.554 | -1.562 | 0.020 | Ripk1/Mapk8/Rbck1/Pgam5/Cav1/Map3k7/Ppif/Itpk1/Cyld/Asah1/Casp8/Ybx3/Bok |
| signaling | necrosis | GO:0070266 | necroptotic process | 18 | -0.581 | -1.506 | 0.028 | Rnf31/Ripk1/Rbck1/Pgam5/Cav1/Map3k7/Ppif/Itpk1/Cyld/Casp8/Ybx3/Bok |
| signaling | oxidation | GO:1903426 | regulation of reactive oxygen species biosynthetic process | 56 | -0.429 | -1.479 | 0.029 | Pkd2/Rhoa/Cav1/Akt1/Rock2/Dynll1/Agt/Gla/Sirpa/Insr/Sphk2/Ddah1/Hrh1/Adgrb1/Tspo/Cd36/Abcd2/Aif1/Abcd1 |
| signaling | oxidation | GO:1903409 | reactive oxygen species biosynthetic process | 66 | -0.421 | -1.499 | 0.018 | Gbf1/Pkd2/Rhoa/Cav1/Akt1/Rock2/Dynll1/Agt/Gla/Sirpa/Insr/Sphk2/Ddah1/Hrh1/Adgrb1/Tspo/Cd36/Abcd2/Aif1/Acox1/Abcd1 |
| signaling | oxidation | GO:0033539 | fatty acid beta-oxidation using acyl-CoA dehydrogenase | 10 | -0.686 | -1.554 | 0.027 | Etfa/Acadm/Etfb/Acadl/Acad11/Acadvl |
| signaling | oxidation | GO:0034440 | lipid oxidation | 70 | -0.457 | -1.642 | 0.004 | Abcd4/Acadm/Etfb/Apod/Acadl/Cnr1/Dbi/Acat1/Cpt1a/Abcd3/Cd36/Acaa1a/Abcd2/Pex13/Acad11/Appl2/Adipor2/Acox1/Acsl5/Acadvl/Hsd17b4/Abcd1 |
| signaling | oxidation | GO:0006635 | fatty acid beta-oxidation | 51 | -0.499 | -1.681 | 0.003 | Abcd4/Acadm/Etfb/Acadl/Cnr1/Dbi/Acat1/Cpt1a/Abcd3/Acaa1a/Abcd2/Acad11/Acox1/Acsl5/Acadvl/Hsd17b4/Abcd1 |
| signaling | oxidation | GO:0019395 | fatty acid oxidation | 68 | -0.452 | -1.616 | 0.007 | Abcd4/Acadm/Etfb/Acadl/Cnr1/Dbi/Acat1/Cpt1a/Abcd3/Cd36/Acaa1a/Abcd2/Pex13/Acad11/Appl2/Adipor2/Acox1/Acsl5/Acadvl/Hsd17b4/Abcd1 |
| metabolism | ATP biogenesis | GO:0032787 | monocarboxylic acid metabolic process | 304 | -0.333 | -1.459 | 0.001 | Slc1a3/Cyp2u1/Elovl2/Sgpl1/Lpin1/Fads6/Eci2/Gnpat/Echdc1/Gm3839/Cav1/Cygb/Akt1/Agt/Etfa/Lipa/Aldh3a2/Lipe/Insr/Pdk1/Ldhb/Adpgk/Mtch2/Acaca/Gpx4/Acad9/Idh1/Abcd4/Acadm/Acsbg1/Pnkd/Acadsb/Acsl3/Hk2/Ephx1/Etfb/Aldh1a1/Aacs/Acadl/Gapdh/Acot1/Cnr1/Dbi/Aldoc/C3/Mif/Acsl6/Acot6/Acot2/Abhd3/Acat1/Ugt1a7c/Akr1a1/Acsf3/Cyp2s1/Acsl1/Aldh1a2/Cpt1a/Acly/Acot8/Abcd3/Apoa4/Cd36/Acaa1a/Abcd2/Pex13/Alox5ap/Acad11/Appl2/Adipor2/Acox1/Acss1/Acsl5/Acot7/Acadvl/Abhd5/Hsd17b4/Abat/Mpc1/Abcd1/Wdtc1 |
| metabolism | ATP biogenesis | GO:0046395 | carboxylic acid catabolic process | 124 | -0.424 | -1.682 | 0.001 | Sardh/Abcd4/Acadm/Ddah1/Mccc2/Etfb/Gcsh/Acadl/Cnr1/Dbi/Bckdhb/Acot2/Abhd3/Acat1/Akr1a1/Acsf3/Cpt1a/Acot8/Abcd3/Acaa1a/Abcd2/Pex13/Acad11/Gcat/Acox1/Acsl5/Acot7/Acadvl/Hsd17b4/Ddo/Abat/Dao/Abcd1 |
| metabolism | ATP biogenesis | GO:0072329 | monocarboxylic acid catabolic process | 74 | -0.476 | -1.717 | 0.001 | Abcd4/Acadm/Etfb/Acadl/Cnr1/Dbi/Acot2/Abhd3/Acat1/Akr1a1/Cpt1a/Acot8/Abcd3/Acaa1a/Abcd2/Pex13/Acad11/Acox1/Acsl5/Acot7/Acadvl/Hsd17b4/Abcd1 |
| metabolism | ATP biogenesis | GO:0019752 | carboxylic acid metabolic process | 483 | -0.297 | -1.361 | 0.001 | Atcay/Apip/Atic/Apc/Etfa/Ears2/Lipa/Aldh3a2/Npl/Iars/Lipe/Insr/Pdk1/Nars/Ldhb/Adpgk/Mtch2/Bcat2/Acaca/Gpx4/Acad9/Uros/Idh1/Sardh/Abcd4/Acadm/Acsbg1/Pnkd/Acadsb/Ddah1/Mccc2/Mdh2/Acsl3/Comt/Hk2/Ephx1/Adss/Etfb/Gcsh/Aldh1a1/Aacs/Icmt/Pfas/Acadl/Gapdh/Acot1/Yars2/Cnr1/Dbi/Wars2/Aldoc/C3/Adssl1/Mif/Bckdhb/Acsl6/Acot6/Acot2/Abhd3/Acat1/Ugt1a7c/Akr1a1/Acsf3/Cyp2s1/Aldh18a1/Cars2/Wars/Acsl1/Aldh1a2/Aco1/Cpt1a/Acly/Acot8/Abcd3/Apoa4/Idh3b/Cd36/Aasdhppt/Acaa1a/Abcd2/Pex13/Alox5ap/Acad11/Cth/Gcat/Appl2/Adipor2/Cars/Vars2/Acox1/Acss1/Acsl5/Acot7/Acadvl/Aars2/Abhd5/Hsd17b4/Aco2/Ddo/Abat/Aars/Mpc1/Dao/Abcd1/Wdtc1 |
| metabolism | ATP biogenesis | GO:0072350 | tricarboxylic acid metabolic process | 13 | -0.726 | -1.758 | 0.003 | Idh1/Aco1/Acly/Idh3b/Aco2 |
| metabolism | ATP biogenesis | GO:0046033 | AMP metabolic process | 15 | -0.697 | -1.743 | 0.005 | Ampd2/Adsl/Ak1/Adss/Nudt2/Adssl1/Ak2/Ada/Ampd3/Ak4 |
| metabolism | glycolysis/gluconeogenesis | GO:0042180 | cellular ketone metabolic process | 99 | -0.393 | -1.511 | 0.009 | Stard3/Aldoa/Coq7/Kyat3/Gk/Scap/Acsl4/Eif2ak3/Coq4/Fabp3/Haghl/Glo1/Pdk2/Ncor2/Coq5/Cav1/Ndufa9/Akt1/Agt/Atcay/Apc/Fdxr/Pdk1/Coq8a/Cacna1h/Pnkd/Comt/Dkk3/Acadl/Cnr1/Dbi/H6pd/Akr1a1/Cpt1a/Apoa4/Abcd2/Aifm2/Appl2/Acsl5/Acadvl/Abcd1/Wdtc1 |
| metabolism | glycolysis/gluconeogenesis | GO:0016054 | organic acid catabolic process | 132 | -0.411 | -1.655 | 0.001 | Lpin1/Nagk/Eci2/Echdc1/Akt1/Blmh/Etfa/Npl/Lipe/Bckdha/Bcat2/Sardh/Abcd4/Acadm/Acadsb/Ddah1/Mccc2/Etfb/Gcsh/Acadl/Cnr1/Dbi/Bckdhb/Acot2/Abhd3/Acat1/Akr1a1/Acsf3/Cpt1a/Acot8/Abcd3/Acaa1a/Abcd2/Pex13/Acad11/Gcat/Acox1/Acsl5/Acot7/Acadvl/Hsd17b4/Ddo/Abat/Dao/Abcd1 |
| metabolism | DNA/nucleoside phosphate metabolism | GO:0006163 | purine nucleotide metabolic process | 224 | -0.334 | -1.428 | 0.004 | Cacnb4/Insr/Pdk1/Atp5h/Adpgk/Mtch2/Sphk2/Acaca/Nt5c1a/Ak1/Adcy8/Nudt10/Mvd/Acadsb/Uqcc3/Mccc2/Acsl3/Hk2/Adss/Far1/Coasy/Atp5j2/Adcy5/Pfas/Nudt2/Gapdh/Acot1/Aldoc/Adssl1/Mif/Acsl6/Acot6/Acot2/Acat1/Acsl1/Acly/Acot8/Itpa/Ak2/Ada/Adcy10/Acss1/Acsl5/Acot7/Hsd17b4/Ampd3/Atp2b2/Mpc1/Acot9/Abcd1/Ak4/Adcy6 |
| metabolism | DNA/nucleoside phosphate metabolism | GO:0009126 | purine nucleoside monophosphate metabolic process | 28 | -0.596 | -1.710 | 0.003 | Impdh2/Aprt/Ppat/Pnp/Paics/Ampd2/Gmpr2/Adsl/Atic/Nt5c1a/Ak1/Adss/Pfas/Nudt2/Adssl1/Ak2/Ada/Ampd3/Ak4 |
| metabolism | DNA/nucleoside phosphate metabolism | GO:0044728 | DNA methylation or demethylation | 20 | 0.697 | 1.803 | 0.003 | Usp9x/Tdrkh/Zmpste24/Fkbp6/Usp7 |
| metabolism | DNA/nucleoside phosphate metabolism | GO:0009259 | ribonucleotide metabolic process | 221 | -0.346 | -1.480 | 0.001 | Cacnb4/Insr/Pdk1/Atp5h/Adpgk/Mtch2/Sphk2/Acaca/Ak1/Adcy8/Nudt10/Mvd/Acadsb/Uqcc3/Mccc2/Acsl3/Hk2/Adss/Far1/Coasy/Atp5j2/Entpd4/Adcy5/Pfas/Nudt2/Gapdh/Acot1/Aldoc/Adssl1/Mif/Acsl6/Acot6/Acot2/Acat1/Acsl1/Acly/Acot8/Itpa/Ak2/Ada/Adcy10/Cmpk1/Acss1/Acsl5/Acot7/Hsd17b4/Ampd3/Atp2b2/Mpc1/Acot9/Abcd1/Ak4/Adcy6 |
| metabolism | DNA/nucleoside phosphate metabolism | GO:0019693 | ribose phosphate metabolic process | 230 | -0.337 | -1.443 | 0.001 | Cacnb4/Insr/Pdk1/Atp5h/Adpgk/Mtch2/Sphk2/Acaca/Ak1/Adcy8/Nudt10/Mvd/Acadsb/Uqcc3/Mccc2/Acsl3/Hk2/Adss/Far1/Coasy/Atp5j2/Entpd4/Adcy5/Pfas/Nudt2/Gapdh/Acot1/Aldoc/Adssl1/Mif/Acsl6/Acot6/Acot2/Acat1/Acsl1/Acly/Acot8/Itpa/Ak2/Ada/Adcy10/Cmpk1/Acss1/Acsl5/Acot7/Hsd17b4/Ampd3/Atp2b2/Mpc1/Acot9/Abcd1/Ak4/Adcy6 |
| metabolism | DNA/nucleoside phosphate metabolism | GO:0009152 | purine ribonucleotide biosynthetic process | 92 | -0.442 | -1.669 | 0.002 | Dcakd/Aldoa/Impdh2/Aprt/Adcy1/Atp5j/Dld/Pnp/Acsl4/Adcy9/Gucy1b1/Paics/Flcn/Pdk2/Ampd2/Adsl/Adcy7/Atic/Atp5c1/Pdk1/Atp5h/Sphk2/Ak1/Adcy8/Uqcc3/Adss/Coasy/Atp5j2/Adcy5/Pfas/Nudt2/Adssl1/Acsl6/Acat1/Acsl1/Acly/Ak2/Adcy10/Acss1/Acsl5/Acot7/Ampd3/Mpc1/Ak4/Adcy6 |
| metabolism | DNA/nucleoside phosphate metabolism | GO:0033865 | nucleoside bisphosphate metabolic process | 72 | -0.506 | -1.824 | 0.000 | Mvd/Acadsb/Mccc2/Acsl3/Far1/Coasy/Acot1/Acsl6/Acot6/Acot2/Acat1/Acsl1/Acly/Acot8/Acss1/Acsl5/Acot7/Hsd17b4/Mpc1/Acot9/Abcd1 |
| metabolism | DNA/nucleoside phosphate metabolism | GO:0033875 | ribonucleoside bisphosphate metabolic process | 72 | -0.506 | -1.824 | 0.000 | Mvd/Acadsb/Mccc2/Acsl3/Far1/Coasy/Acot1/Acsl6/Acot6/Acot2/Acat1/Acsl1/Acly/Acot8/Acss1/Acsl5/Acot7/Hsd17b4/Mpc1/Acot9/Abcd1 |
| metabolism | DNA/nucleoside phosphate metabolism | GO:0034032 | purine nucleoside bisphosphate metabolic process | 72 | -0.506 | -1.824 | 0.000 | Mvd/Acadsb/Mccc2/Acsl3/Far1/Coasy/Acot1/Acsl6/Acot6/Acot2/Acat1/Acsl1/Acly/Acot8/Acss1/Acsl5/Acot7/Hsd17b4/Mpc1/Acot9/Abcd1 |
| metabolism | DNA/nucleoside phosphate metabolism | GO:0009150 | purine ribonucleotide metabolic process | 211 | -0.361 | -1.526 | 0.001 | Cacnb4/Insr/Pdk1/Atp5h/Adpgk/Mtch2/Sphk2/Acaca/Ak1/Adcy8/Nudt10/Mvd/Acadsb/Uqcc3/Mccc2/Acsl3/Hk2/Adss/Far1/Coasy/Atp5j2/Adcy5/Pfas/Nudt2/Gapdh/Acot1/Aldoc/Adssl1/Mif/Acsl6/Acot6/Acot2/Acat1/Acsl1/Acly/Acot8/Itpa/Ak2/Ada/Adcy10/Acss1/Acsl5/Acot7/Hsd17b4/Ampd3/Atp2b2/Mpc1/Acot9/Abcd1/Ak4/Adcy6 |
| metabolism | DNA/nucleoside phosphate metabolism | GO:0009167 | purine ribonucleoside monophosphate metabolic process | 27 | -0.592 | -1.686 | 0.004 | Impdh2/Aprt/Ppat/Pnp/Paics/Ampd2/Gmpr2/Adsl/Atic/Ak1/Adss/Pfas/Nudt2/Adssl1/Ak2/Ada/Ampd3/Ak4 |
| metabolism | DNA/nucleoside phosphate metabolism | GO:0033866 | nucleoside bisphosphate biosynthetic process | 31 | -0.554 | -1.622 | 0.005 | Coasy/Acsl6/Acat1/Acsl1/Acly/Acss1/Acsl5/Acot7/Mpc1 |
| metabolism | DNA/nucleoside phosphate metabolism | GO:0034030 | ribonucleoside bisphosphate biosynthetic process | 31 | -0.554 | -1.622 | 0.005 | Coasy/Acsl6/Acat1/Acsl1/Acly/Acss1/Acsl5/Acot7/Mpc1 |
| metabolism | DNA/nucleoside phosphate metabolism | GO:0034033 | purine nucleoside bisphosphate biosynthetic process | 31 | -0.554 | -1.622 | 0.005 | Coasy/Acsl6/Acat1/Acsl1/Acly/Acss1/Acsl5/Acot7/Mpc1 |
| metabolism | DNA/nucleoside phosphate metabolism | GO:0072521 | purine-containing compound metabolic process | 237 | -0.317 | -1.362 | 0.007 | Cacnb4/Insr/Pdk1/Atp5h/Adpgk/Mtch2/Sphk2/Acaca/Nt5c1a/Ak1/Adcy8/Nudt10/Mvd/Acadsb/Uqcc3/Mccc2/Acsl3/Macrod2/Hk2/Adss/Far1/Coasy/Atp5j2/Adcy5/Icmt/Pfas/Nudt2/Gapdh/Acot1/Aldoc/Adssl1/Mif/Acsl6/Acot6/Acot2/Acat1/Acsl1/Acly/Acot8/Itpa/Ak2/Ada/Adcy10/Acss1/Acsl5/Acot7/Hsd17b4/Ampd3/Atp2b2/Mpc1/Acot9/Abcd1/Ak4/Adcy6 |
| metabolism | DNA/nucleoside phosphate metabolism | GO:0009260 | ribonucleotide biosynthetic process | 99 | -0.398 | -1.528 | 0.007 | Adcy9/Gucy1b1/Paics/Flcn/Pdk2/Ampd2/Adsl/Adcy7/Atic/Atp5c1/Pdk1/Atp5h/Sphk2/Ak1/Adcy8/Uqcc3/Adss/Coasy/Atp5j2/Adcy5/Pfas/Nudt2/Adssl1/Acsl6/Acat1/Acsl1/Acly/Ak2/Adcy10/Cmpk1/Acss1/Acsl5/Acot7/Ampd3/Mpc1/Ak4/Adcy6 |
| metabolism | DNA/nucleoside phosphate metabolism | GO:0009154 | purine ribonucleotide catabolic process | 27 | -0.562 | -1.602 | 0.010 | Acat1/Itpa/Ada/Acot7/Ampd3/Abcd1 |
| metabolism | lipid metabolism | GO:1901568 | fatty acid derivative metabolic process | 24 | -0.593 | -1.671 | 0.010 | Acsl3/Far1/Acsl6/Abhd16a/Acat1/Acsl1/Acsl5/Acot7/Hsd17b4/Abcd1 |
| metabolism | lipid metabolism | GO:0035384 | thioester biosynthetic process | 23 | -0.601 | -1.665 | 0.010 | Acsl6/Acat1/Acsl1/Acly/Acss1/Acsl5/Mpc1 |
| metabolism | lipid metabolism | GO:0071616 | acyl-CoA biosynthetic process | 23 | -0.601 | -1.665 | 0.010 | Acsl6/Acat1/Acsl1/Acly/Acss1/Acsl5/Mpc1 |
| metabolism | lipid metabolism | GO:0019217 | regulation of fatty acid metabolic process | 44 | -0.510 | -1.680 | 0.006 | Acadl/Cnr1/Dbi/Cpt1a/Apoa4/Abcd2/Appl2/Acsl5/Acadvl/Abcd1/Wdtc1 |
| metabolism | lipid metabolism | GO:0042304 | regulation of fatty acid biosynthetic process | 19 | -0.659 | -1.732 | 0.006 | Acadl/Apoa4/Abcd2/Acadvl/Abcd1/Wdtc1 |
| metabolism | lipid metabolism | GO:0001676 | long-chain fatty acid metabolic process | 47 | -0.508 | -1.696 | 0.005 | Lipe/Gpx4/Acad9/Acsbg1/Acsl3/Ephx1/Acadl/Acot1/Acsl6/Acot2/Cyp2s1/Acsl1/Cpt1a/Cd36/Acsl5/Acot7/Abcd1 |
| metabolism | lipid metabolism | GO:0035337 | fatty-acyl-CoA metabolic process | 14 | -0.724 | -1.789 | 0.003 | Acsl3/Far1/Acsl6/Acsl1/Acsl5/Acot7/Hsd17b4/Abcd1 |
| metabolism | lipid metabolism | GO:0045833 | negative regulation of lipid metabolic process | 44 | -0.535 | -1.763 | 0.003 | Apoa2/Hrh1/Fmc1/Dkk3/Apod/Acadl/Cnr1/Dbi/Bscl2/Atp1a1/Washc1/Appl2/Acadvl/Wdtc1 |
| metabolism | lipid metabolism | GO:0046890 | regulation of lipid biosynthetic process | 83 | -0.449 | -1.653 | 0.002 | Sphk2/Asah1/Idh1/Acsl3/Hrh1/Dkk3/Acadl/Tspo/Abhd6/Dbi/C3/H6pd/Bscl2/Atp1a1/Apoa4/Abcd2/Acsl5/Acadvl/Abca3/Abcd1/Wdtc1 |
| metabolism | lipid metabolism | GO:0016042 | lipid catabolic process | 176 | -0.365 | -1.499 | 0.002 | Gba2/Sgpl1/Lpin1/Eci2/Echdc1/Hexa/Alk/Akt1/Gla/Etfa/Lipa/Gdpd1/Lipe/Inpp5f/Srd5a3/Asah1/Idh1/Abcd4/Acadm/Apoa2/Etfb/Fmc1/Plcxd3/Acadl/Naga/Cnr1/Abhd6/Dbi/Abhd12/Acot2/Abhd3/Abhd16a/Acat1/Ugt1a7c/Bscl2/Cpt1a/Acot8/Abcd3/Apoa4/Acaa1a/Apoh/Abcd2/Pex13/Acad11/Abhd4/Acox1/Acsl5/Acot7/Acadvl/Abhd5/Hsd17b4/Abcd1 |
| metabolism | lipid metabolism | GO:0006637 | acyl-CoA metabolic process | 61 | -0.533 | -1.873 | 0.000 | Pdk1/Acaca/Mvd/Acadsb/Acsl3/Far1/Acot1/Acsl6/Acot6/Acot2/Acat1/Acsl1/Acly/Acot8/Acss1/Acsl5/Acot7/Hsd17b4/Mpc1/Acot9/Abcd1 |
| metabolism | lipid metabolism | GO:0035383 | thioester metabolic process | 61 | -0.533 | -1.873 | 0.000 | Pdk1/Acaca/Mvd/Acadsb/Acsl3/Far1/Acot1/Acsl6/Acot6/Acot2/Acat1/Acsl1/Acly/Acot8/Acss1/Acsl5/Acot7/Hsd17b4/Mpc1/Acot9/Abcd1 |
| metabolism | lipid metabolism | GO:0006631 | fatty acid metabolic process | 208 | -0.378 | -1.591 | 0.000 | Cyp2u1/Elovl2/Sgpl1/Lpin1/Fads6/Eci2/Gnpat/Echdc1/Cav1/Cygb/Akt1/Agt/Etfa/Lipa/Aldh3a2/Lipe/Pdk1/Acaca/Gpx4/Acad9/Abcd4/Acadm/Acsbg1/Acadsb/Acsl3/Ephx1/Etfb/Aacs/Acadl/Acot1/Cnr1/Dbi/C3/Mif/Acsl6/Acot6/Acot2/Abhd3/Acat1/Acsf3/Cyp2s1/Acsl1/Cpt1a/Acly/Acot8/Abcd3/Apoa4/Cd36/Acaa1a/Abcd2/Pex13/Alox5ap/Acad11/Appl2/Adipor2/Acox1/Acss1/Acsl5/Acot7/Acadvl/Abhd5/Hsd17b4/Abcd1/Wdtc1 |
| metabolism | lipid metabolism | GO:0044242 | cellular lipid catabolic process | 137 | -0.420 | -1.703 | 0.000 | Gba2/Sgpl1/Lpin1/Eci2/Echdc1/Hexa/Akt1/Gla/Etfa/Gdpd1/Lipe/Inpp5f/Srd5a3/Asah1/Idh1/Abcd4/Acadm/Apoa2/Etfb/Acadl/Naga/Cnr1/Abhd6/Dbi/Abhd12/Acot2/Abhd3/Abhd16a/Acat1/Bscl2/Cpt1a/Acot8/Abcd3/Apoa4/Acaa1a/Apoh/Abcd2/Pex13/Acad11/Acox1/Acsl5/Acot7/Acadvl/Abhd5/Hsd17b4/Abcd1 |
| metabolism | lipid metabolism | GO:0050996 | positive regulation of lipid catabolic process | 16 | -0.735 | -1.869 | 0.000 | Apoa2/Cpt1a/Apoa4/Apoh/Abcd2/Acsl5/Abhd5/Abcd1 |
| metabolism | lipid metabolism | GO:0045922 | negative regulation of fatty acid metabolic process | 10 | -0.823 | -1.862 | 0.000 | Acadl/Cnr1/Dbi/Appl2/Acadvl/Wdtc1 |
| metabolism | lipid metabolism | GO:0009062 | fatty acid catabolic process | 67 | -0.485 | -1.730 | 0.001 | Abcd4/Acadm/Etfb/Acadl/Cnr1/Dbi/Acot2/Abhd3/Acat1/Cpt1a/Acot8/Abcd3/Acaa1a/Abcd2/Pex13/Acad11/Acox1/Acsl5/Acot7/Acadvl/Hsd17b4/Abcd1 |
| metabolism | lipid metabolism | GO:0000038 | very long-chain fatty acid metabolic process | 23 | -0.661 | -1.832 | 0.001 | Abcd4/Acsbg1/Acsl6/Acot2/Acsl1/Abcd3/Acaa1a/Abcd2/Acox1/Hsd17b4/Abcd1 |
| metabolism | lipid metabolism | GO:0051055 | negative regulation of lipid biosynthetic process | 18 | -0.688 | -1.782 | 0.002 | Hrh1/Dkk3/Acadl/Atp1a1/Acadvl/Wdtc1 |
| metabolism | lipid metabolism | GO:0019216 | regulation of lipid metabolic process | 179 | -0.364 | -1.506 | 0.002 | Asah1/Idh1/Disp3/Bbs4/Apoa2/Phb2/Acsl3/Hrh1/Fmc1/Dkk3/Mtmr2/Apod/Fmo5/Dnajc15/Acadl/Tspo/Arf1/Cnr1/Abhd6/Dbi/C3/H6pd/Bscl2/Cpt1a/Atp1a1/Apoa4/Cd36/Ttc39b/Apoh/Abcd2/Washc1/Abca7/Appl2/Epha8/Acsl5/Acadvl/Abhd5/Abca3/Abcd1/Atg14/Wdtc1 |
| metabolism | small molecule metabolism | GO:0044282 | small molecule catabolic process | 195 | -0.354 | -1.475 | 0.002 | Aig1/Inpp5k/Shmt2/Mgat1/Acat2/Pfkl/Ppat/Slc27a4/Pnp/Qdpr/Cda/Mtmr7/Bad/Synj2/Pipox/Inpp1/Haghl/Acad8/Acat3/Glo1/Aldh1l1/Gmpr2/Lpin1/Nagk/Esd/Eci2/Echdc1/Akt1/Blmh/Etfa/Npl/Lipe/Bckdha/Dera/Inpp4b/Srd5a3/Bcat2/Sardh/Abcd4/Inpp5b/Acadm/Pnkd/Acadsb/Ddah1/Mccc2/Etfb/Gcsh/Mtmr2/Inpp5a/Acadl/Cnr1/Dbi/Bckdhb/Acot2/Abhd3/Acat1/Akr1a1/Acsf3/Cpt1a/Acot8/Abcd3/Acaa1a/Abcd2/Pex13/Ada/Acad11/Gcat/Acox1/Acsl5/Acot7/Acadvl/Hsd17b4/Ddo/Abat/Dao/Abcd1 |
| metabolism | protein metabolism | GO:0044257 | cellular protein catabolic process | 452 | 0.343 | 1.425 | 0.001 | Uqcc2/Vps37a/Ube2j1/Ubr3/Ube2k/Usp9x/Svip/Wdr91/Aup1/Yme1l1/Ube2j2/Vps11/Vps28/Bnip3/Usp8/Uba7/Ube2i/Cdc23/Psmd1/Ubap1/Ube2v2/Ube2d1/Ube2l3/Brsk2/Usp4/Usp47/Amn1/Vcp/Agbl4/Cul5/Nub1/Usp46/Ubxn6/Uchl1/Rpl23/Tmem67/Usp30/Ube2a/Ube2n/Isg15/Rnf167/Ltn1/Vps35/Ptpn23/Cul9/Zmpste24/Sharpin/Dcaf11/Ctnnb1/Ubac2/Hace1/Ctsf/Mgat3/Eif3h/Ddi2/H13/Wwp1/Ufd1/Uba1/Vps37c/Pdcd6ip/Vhl/Tollip/Atpif1/Ccar2/Prpf19/Clpx/Usp24/Dnajb12/Vps25/Zfand2b/Map1a/Ubr2/Smurf2/Hecw1/Ufl1/Lrp1/Psmd2/Pomt2/Atxn3/Ddrgk1/Marchf6/Usp7/Tpp1/Get4/Fbxo7/Ube2g2/Gclc/Ubr1/Csnk1d/Erlin1/Commd1/Kctd6/Atg7/Pten/Nedd4/Os9/Chmp4b/Rc3h1/Abca2/Arih2/Ezr/Otud7b/Fbxl18 |
| metabolism | protein metabolism | GO:0043436 | oxoacid metabolic process | 491 | -0.292 | -1.332 | 0.001 | Atcay/Apip/Atic/Apc/Etfa/Ears2/Lipa/Aldh3a2/Npl/Iars/Lipe/Insr/Pdk1/Nars/Ldhb/Adpgk/Mtch2/Bcat2/Acaca/Gpx4/Acad9/Uros/Idh1/Sardh/Abcd4/Acadm/Acsbg1/Pnkd/Acadsb/Ddah1/Mccc2/Mdh2/Acsl3/Comt/Hk2/Ephx1/Adss/Etfb/Gcsh/Aldh1a1/Aacs/Icmt/Pfas/Acadl/Gapdh/Acot1/Yars2/Cnr1/Dbi/Wars2/Aldoc/C3/Adssl1/Mif/Bckdhb/Acsl6/Acot6/Acot2/Abhd3/Acat1/Ugt1a7c/Akr1a1/Acsf3/Cyp2s1/Aldh18a1/Cars2/Wars/Acsl1/Aldh1a2/Aco1/Cpt1a/Acly/Acot8/Abcd3/Apoa4/Idh3b/Cd36/Aasdhppt/Acaa1a/Abcd2/Pex13/Alox5ap/Acad11/Cth/Gcat/Appl2/Adipor2/Cars/Vars2/Acox1/Acss1/Acsl5/Acot7/Acadvl/Aars2/Abhd5/Hsd17b4/Aco2/Ddo/Abat/Aars/Mpc1/Dao/Abcd1/Wdtc1 |
| metabolism | protein metabolism | GO:0042176 | regulation of protein catabolic process | 231 | 0.378 | 1.477 | 0.004 | Uqcc2/Ube2k/Svip/Wdr91/Rhbdd3/Tmem9/Vps11/Vps28/Usp8/Gpc3/Psmd1/Cst3/Ube2v2/Vcp/Agbl4/Nub1/Rpl23/Tmem67/Apc2/Vps35/Hace1/Mgat3/Eif3h/Flna/Wwp1/App/Vhl/Atpif1/Ccar2/Snca/Map1a/Smurf2/Hecw1/Ufl1/Lrp1/Psmd2/Atxn3/Ddrgk1/Usp7/Gclc/Csnk1d/Commd1/Atg7/Gpld1/Pten/Nedd4/Abca2/Arih2/Ezr |
| metabolism | protein metabolism | GO:0045732 | positive regulation of protein catabolic process | 126 | 0.434 | 1.577 | 0.007 | Uqcc2/Rhbdd3/Vps11/Vps28/Gpc3/Ube2v2/Vcp/Agbl4/Nub1/Tmem67/Apc2/Vps35/Hace1/Wwp1/App/Atpif1/Smurf2/Hecw1/Lrp1/Atxn3/Ddrgk1/Gclc/Csnk1d/Atg7/Gpld1/Pten/Nedd4/Abca2/Arih2/Ezr |
| others | neurogenesis | GO:0048483 | autonomic nervous system development | 13 | 0.670 | 1.553 | 0.036 | Vcam1/Fzd3/Hes1/Ctnnb1/Plxna4 |
| others | muscle | GO:0051153 | regulation of striated muscle cell differentiation | 28 | 0.593 | 1.639 | 0.007 | Naca/Frs2/Ybx1/Efnb2/Bcl2/Mapk14/G6pdx |
| others | muscle | GO:0060537 | muscle tissue development | 150 | 0.414 | 1.540 | 0.006 | Uqcc2/Naca/Prkaa1/Poglut1/Ryr2/Ndufv2/Cdc42/Frs2/Sgcz/Efnb2/Myorg/Snw1/Zfand5/Cacybp/Zmpste24/Ncam1/Ctnnb1/Ly6e/Bcl2/Itgb1/Erbb3/Rps6kb1/Homer1/Cby1/Mapk14/Ttn/Crhr2/Fkbp1a/G6pdx/Atg7/Csrp2/Pten/Nf1/Prkg1/Luc7l/Sgcb/Csrp1 |
| others | muscle | GO:0045933 | positive regulation of muscle contraction | 12 | -0.731 | -1.743 | 0.007 | Chrm3/Rhoa/Cttn/Chga/Atp1a1/Adra1a/Ada/Abat |
| others | muscle | GO:0048636 | positive regulation of muscle organ development | 10 | 0.758 | 1.635 | 0.009 | Naca/Prkaa1/Ctnnb1/Bcl2/Erbb3/Rps6kb1 |
| others | muscle | GO:1901863 | positive regulation of muscle tissue development | 10 | 0.758 | 1.635 | 0.009 | Naca/Prkaa1/Ctnnb1/Bcl2/Erbb3/Rps6kb1 |
| others | muscle | GO:0014706 | striated muscle tissue development | 142 | 0.411 | 1.510 | 0.009 | Uqcc2/Naca/Prkaa1/Ryr2/Ndufv2/Cdc42/Frs2/Sgcz/Efnb2/Myorg/Snw1/Cacybp/Zmpste24/Ncam1/Ctnnb1/Ly6e/Bcl2/Itgb1/Erbb3/Rps6kb1/Homer1/Cby1/Mapk14/Ttn/Crhr2/Fkbp1a/G6pdx/Atg7/Pten/Nf1/Prkg1/Luc7l/Sgcb |
| others | muscle | GO:0035914 | skeletal muscle cell differentiation | 15 | 0.711 | 1.720 | 0.012 | Uqcc2/Snw1 |
| others | muscle | GO:0007519 | skeletal muscle tissue development | 55 | 0.487 | 1.540 | 0.018 | Uqcc2/Naca/Prkaa1/Myorg/Snw1/Ctnnb1/Bcl2/Rps6kb1/Homer1/Mapk14/Crhr2 |
| others | muscle | GO:0060538 | skeletal muscle organ development | 59 | 0.464 | 1.484 | 0.025 | Uqcc2/Naca/Prkaa1/Myorg/Snw1/Ctnnb1/Bcl2/Rps6kb1/Homer1/Mapk14/Crhr2/Cntfr |
| others | muscle | GO:0061061 | muscle structure development | 221 | 0.346 | 1.347 | 0.026 | Uqcc2/Naca/Prkaa1/Cmtm5/Myh9/Vangl2/Chat/Ryr2/Frs2/Ybx1/Efnb2/Uchl1/Utrn/Myorg/Snw1/Cacybp/Hes1/Zmpste24/Fzd2/Ctnnb1/Ly6e/Tmod3/Bcl2/Camk1/Itgb1/Erbb3/Rps6kb1/Smarca2/Homer1/Cby1/Mapk14/Ttn/Ehd1/Crhr2/Fkbp1a/Cntfr/G6pdx/Actg1/Atg7/Csrp2/Nf1/Prkg1/Luc7l/Sgcb |
| others | ubiquitination | GO:0000209 | protein polyubiquitination | 101 | 0.385 | 1.363 | 0.044 | Ube2j1/Ube2k/Zswim2/Ube2j2/Cdc23/Ube2v2/Ube2d1/Ube2l3/Ube2a/Ube2n/Rnf167/Sharpin/Ctnnb1/Hace1/Wwp1/Bcl2/Prpf19/Anapc13/Smurf2/Hecw1/Traf3/Marchf6/Mgrn1/Ube2g2/Aktip/Nedd4/Rc3h1/Arih2 |
| others | ubiquitination | GO:0016567 | protein ubiquitination | 250 | 0.364 | 1.434 | 0.006 | Ube2j1/Ubr3/Ube2k/Zswim2/Vcpip1/Ube2j2/Vps11/Vps28/Cdc23/Vps18/Ube2v2/Ube2d1/Ube2l3/Klhl9/Atg3/Pja1/Septin4/Usp4/Vcp/Cul5/Fgfr3/Nub1/Rpl23/Ube2a/Ube2n/Isg15/Rnf167/Ltn1/Cul9/Sharpin/Ctnnb1/Hace1/Wwp1/Uba1/Bcl2/Vhl/Tbc1d7/Prpf19/Wdr48/Anapc13/Ubr2/Smurf2/Hecw1/Traf3/Ufl1/Marchf6/Mgrn1/Fbxo7/Ube2g2/Gclc/Fkbp1a/Ubr1/Herc4/Arrdc3/Commd1/Atg7/Aktip/Pten/Nedd4/Os9/Rc3h1/Arih2 |
| others | ubiquitination | GO:0006511 | ubiquitin-dependent protein catabolic process | 339 | 0.316 | 1.279 | 0.026 | Vps37a/Ube2j1/Ubr3/Ube2k/Usp9x/Svip/Aup1/Ube2j2/Vps28/Usp8/Ube2i/Cdc23/Psmd1/Ubap1/Ube2v2/Ube2d1/Ube2l3/Usp4/Usp47/Amn1/Vcp/Agbl4/Cul5/Nub1/Usp46/Uchl1/Rpl23/Tmem67/Usp30/Ube2a/Ube2n/Rnf167/Ltn1/Ptpn23/Cul9/Sharpin/Dcaf11/Ctnnb1/Hace1/Eif3h/Wwp1/Ufd1/Uba1/Vps37c/Tollip/Ccar2/Usp24/Dnajb12/Vps25/Zfand2b/Map1a/Ubr2/Smurf2/Hecw1/Ufl1/Psmd2/Atxn3/Ddrgk1/Marchf6/Usp7/Fbxo7/Ube2g2/Gclc/Ubr1/Csnk1d/Erlin1/Commd1/Kctd6/Atg7/Pten/Nedd4/Os9/Rc3h1/Arih2/Otud7b/Fbxl18 |
| others | protein modification & regulation | GO:0032446 | protein modification by small protein conjugation | 273 | 0.388 | 1.544 | 0.001 | Ube2j1/Ubr3/Ube2k/Zswim2/Vcpip1/Ube2j2/Urm1/Vps11/Vps28/Ube2m/Uba7/Ube2i/Cdc23/Vps18/Uba2/Ube2v2/Ube2d1/Ube2l3/Klhl9/Atg3/Pja1/Septin4/Usp4/Uba3/Vcp/Cul5/Fgfr3/Nub1/Rpl23/Ube2a/Ube2n/Isg15/Rnf167/Uba5/Ltn1/Cul9/Sharpin/Ctnnb1/Hace1/Wwp1/Uba1/Bcl2/Vhl/Tollip/Tbc1d7/Prpf19/Wdr48/Anapc13/Ubr2/Smurf2/Hecw1/Traf3/Ufl1/Ddrgk1/Marchf6/Mgrn1/Ube2f/Fbxo7/Ube2g2/Gclc/Fkbp1a/Ubr1/Herc4/Arrdc3/Commd1/Atg7/Aktip/Pten/Nedd4/Os9/Rc3h1/Arih2 |
| others | protein modification & regulation | GO:0031334 | positive regulation of protein-containing complex assembly | 148 | -0.398 | -1.607 | 0.001 | Pycard/Gmfb/Cck/Snx9/Brk1/Pfn2/Rhoa/Cav1/Ctnna2/Ap1ar/Tfrc/Evl/Bak1/Coro1b/Apc/Wasf3/Bin1/Piezo1/Bid/Atat1/Coro1a/Cttn/Psmc3/Myo1c/Arpc1b/Plek/Caly/Carmil1/Cyfip1/Fes/Psmc2/Drg1/Arf1/Wasl/Arpc1a/Wars/Cd36/Msn/Washc1/Bag4/Arpc2/Abca3/Abca1/Wasf2/Wasf1/Abi2 |
| others | protein modification & regulation | GO:0051258 | protein polymerization | 203 | -0.355 | -1.491 | 0.001 | Pycard/Hdgfl3/Gmfb/Gba2/Snx9/Brk1/Pfn2/Dyrk1a/Rhoa/Dnm3/Cav1/Mtpn/Fgb/Capn1/Ctnna2/Ap1ar/Evl/Coro1b/Apc/Myadm/Spta1/Wasf3/Bin1/Gpx4/Bbs4/Coro1a/Chmp3/Cttn/Tubgcp3/Twf1/Capza1/Myo1c/Arpc1b/Carmil1/Cyfip1/Fes/Drg1/Arf1/Cfl1/Wasl/Fgf13/Arpc1a/Add2/Washc1/Arfgef1/Bag4/Arpc2/Chmp2a/Aif1/Wasf2/Vtn/Tubgcp4/Twf2/Wasf1/Abi2/Tubg2/Vdac2/Tubgcp6 |
| others | protein modification & regulation | GO:0070647 | protein modification by small protein conjugation or removal | 332 | 0.363 | 1.468 | 0.002 | Ube2j1/Ubr3/Ube2k/Usp9x/Zswim2/Vcpip1/Ube2j2/Urm1/Usp54/Vps11/Vps28/Usp8/Ube2m/Uba7/Ube2i/Cdc23/Vps18/Uba2/Ube2v2/Ube2d1/Ube2l3/Klhl9/Atg3/Pja1/Septin4/Usp4/Usp47/Uba3/Vcp/Cul5/Fgfr3/Nub1/Usp46/Uchl1/Rpl23/Usp30/Ube2a/Ube2n/Isg15/Rnf167/Uba5/Ltn1/Cul9/Sharpin/Senp8/Ctnnb1/Hace1/Wwp1/Uba1/Bcl2/Usp35/Vhl/Tollip/Tbc1d7/Prpf19/Wdr48/Abraxas2/Usp24/Anapc13/Ubr2/Smurf2/Hecw1/Traf3/Ufl1/Atxn3/Ddrgk1/Marchf6/Usp7/Mgrn1/Ube2f/Fbxo7/Ube2g2/Gclc/Fkbp1a/Ubr1/Herc4/Arrdc3/Commd1/Atg7/Aktip/Pten/Nedd4/Os9/Rc3h1/Arih2/Gps1/Otud7b |
| others | protein modification & regulation | GO:0032273 | positive regulation of protein polymerization | 94 | -0.417 | -1.578 | 0.004 | Ptk2b/Mapre1/Pde4dip/Tppp3/Arpc3/Arl2/Mapk8/Grb2/Arpc5/Carmil2/Arfip1/Nck2/Ttbk1/Pycard/Gmfb/Snx9/Brk1/Pfn2/Rhoa/Cav1/Ctnna2/Ap1ar/Evl/Coro1b/Apc/Wasf3/Bin1/Coro1a/Cttn/Myo1c/Arpc1b/Carmil1/Cyfip1/Fes/Drg1/Arf1/Wasl/Arpc1a/Washc1/Bag4/Arpc2/Wasf2/Wasf1/Abi2 |
| others | protein modification & regulation | GO:0032271 | regulation of protein polymerization | 162 | -0.358 | -1.457 | 0.006 | Sptbn1/Ptk2b/Mapre1/Pde4dip/Eml2/Clasp2/Tppp3/Arpc3/Capg/Capza2/Arl2/Prkcd/Mapk8/Grb2/Arpc5/Map2/Carmil2/Prune1/Arfip1/Abl1/Nck2/Pik3r2/Fkbp4/Ttbk1/Pycard/Gmfb/Gba2/Snx9/Brk1/Pfn2/Dyrk1a/Rhoa/Cav1/Mtpn/Capn1/Ctnna2/Ap1ar/Evl/Coro1b/Apc/Myadm/Spta1/Wasf3/Bin1/Bbs4/Coro1a/Cttn/Twf1/Capza1/Myo1c/Arpc1b/Carmil1/Cyfip1/Fes/Drg1/Arf1/Cfl1/Wasl/Arpc1a/Add2/Washc1/Arfgef1/Bag4/Arpc2/Wasf2/Twf2/Wasf1/Abi2/Vdac2 |
| others | protein modification & regulation | GO:0051289 | protein homotetramerization | 28 | -0.547 | -1.569 | 0.012 | Hsd17b10/Crtc1/Shmt2/Trpm2/Osbpl2/B2m/Pkd2/Evl/Apip/Acaca/Aldh9a1/Acot13/Aldh1a2/Cth/Appl2/Cryz |
