# Supplementary material for: The ultrastructural and proteomic analysis of mitochondria‐associated endoplasmic reticulum membrane in the midbrain of a Parkinson's disease mouse model
Source: Aging Cell. 2024 Nov 29;24(4):e14436. doi: 10.1111/acel.14436 (PMC11984660; doi:10.1111/acel.14436)
Supplement: Supplementary file 19 — Table S13. List of consensus MAM proteins in MAM proteomics. [file ACEL-24-e14436-s018.docx]

**Supplementary Table 13** **List of consensus MAM proteins in MAM proteomics**

| Detected proteins | Undetected proteins |
| --- | --- |
| Tmed10 | Dgat2 |
| Mogs | Atp5a1 |
| Aldh1l1 | Srprb |
| Itpr1 | Grp78 |
| Apoc1 | Pc |
| Cdc42bpb | Atp5b |
| Rplp0 | Hrsp12 |
| Anxa6 |  |
| Ndrg2 |  |
| Rpl6 |  |
| Clic4 |  |
| Rab5b |  |
| Calr |  |
| Rab1A |  |
| Park7 |  |
| Ncstn |  |
| Mdh1 |  |
| Pdia3 |  |
| Ndufb6 |  |
| Alcam |  |
| Apobr |  |
| Rpl10a |  |
| Rpl18 |  |
| Itgb1 |  |
| Hspa8 |  |
| Cox5a |  |
| Erlin2 |  |
| Cycs |  |
| Gstp1 |  |
| Mttp |  |
| Arpc4 |  |
| Mccc1 |  |
| Pacsin3 |  |
| Maob |  |
| Pdia6 |  |
| Ppib |  |
| Ptprf |  |
| Bdh1 |  |
| Tmem30a |  |
| Ndufa9 |  |
| Rpl9 |  |
| Uqcrc2 |  |
| Prkcsh |  |
| Tmbim6 |  |
| Rab18 |  |
| Lap3 |  |
| Acox1 |  |
| Ero1b |  |
| Uqcrfs1 |  |
| Aldh9a1 |  |
| Aldh2 |  |
| Sptbn1 |  |
| Ndufb4 |  |
| Atp5h |  |
| Slc25a1 |  |
| Jup |  |
| Dnm2 |  |
| Actn1 |  |
| Lrp1 |  |
| Capza2 |  |
| Hsp90b1 |  |
| Itpr2 |  |
| Uqcrc1 |  |
| Cyfip1 |  |
| Rab1b |  |
| Acsl4 |  |
| Cox4i1 |  |
| Scd1 |  |
| Cltc |  |
| Vdac3 |  |
| P4hb |  |
| Atp2a2 |  |
| Ndufb10 |  |
| Prdx5 |  |
| Pgk1 |  |
| Glud1 |  |
| Rps3a |  |
| Rps14 |  |
| Apoe |  |
| Rpl8 |  |
| Decr1 |  |
| Ass1 |  |
| Tst |  |
| Rab32 |  |
| Uggt1 |  |
| Abcb7 |  |
| Ero1a |  |
| Letm1 |  |
| Ndufv2 |  |
| Gnas |  |
| Tpi1 |  |
| Etfa |  |
| Ap2b1 |  |
| Ldha |  |
| Hadhb |  |
| Picalm |  |
| Mdh2 |  |
| Prdx1 |  |
| Canx |  |
| Atp6v1a |  |
| Etfdh |  |
| Ganab |  |
| Serpina3k |  |
| Qdpr |  |
| Pgrmc1 |  |
| Eef1a1 |  |
| Rps3 |  |
| Gnb1 |  |
| Vdac2 |  |
| Dlat |  |
| Cfl1 |  |
| Sfxn1 |  |
| Hyou1 |  |
| Acsl1 |  |
| Hspa9 |  |
| Hsp90ab1 |  |
| Slc25a3 |  |
| Myh9 |  |
| Sigmar1 |  |
| Hspd1 |  |
| Alb |  |
| Rps8 |  |
| Sdhb |  |
| Afg3l2 |  |
| Cyc1 |  |
| Gna13 |  |
| Mtdh |  |
| Erp44 |  |
| Samm50 |  |
| Acaa2 |  |
| Gpd2 |  |
| Cisd1 |  |
| Rpl7 |  |
| Prdx6 |  |
| Slc25a12 |  |
| Vapa |  |
| Pgm1 |  |
| Phb2 |  |
| Ap2a2 |  |
| Stoml2 |  |
| Ptdss1 |  |
| Ndufs3 |  |
| Ndufa4 |  |
| Acat1 |  |
| Atp5o |  |
| Ahcy |  |
| Rpl4 |  |
| Immt |  |
| Rpl10 |  |
| Atp5c1 |  |
| Rab2a |  |
| Rpl5 |  |
| Pa2g4 |  |
| Myo1b |  |
| Ap2a1 |  |
| Eef2 |  |
| Rplp2 |  |
| Hadha |  |
| Ndufb5 |  |
| Idh1 |  |
| Rps9 |  |
| Fh |  |
| Hsd17b4 |  |
| Aifm1 |  |
| Msn |  |
| Mfn1 |  |
| Rps24 |  |
| Rrbp1 |  |
| Vdac1 |  |
| Mthfd1 |  |
| Snd1 |  |
| Phb |  |
| Eno1 |  |
| Ndufv1 |  |
| Cd81 |  |
| Rdx |  |
| Actn4 |  |
| Ndufs1 |  |
| Pcyox1 |  |
| Cct8 |  |
| Mlec |  |
| Rpn1 |  |
| Cs |  |
| Suclg1 |  |
| Nomo1 |  |
| Got2 |  |
| Rala |  |
| Aldh2 |  |
| Erp29 |  |
| Rab7a |  |
| Ndufs7 |  |
| Slc16a1 |  |
| Rab14 |  |
| Rab21 |  |
| Ndufa13 |  |
| Abcd3 |  |
| Echs1 |  |
| Atp1a1 |  |
| Sdha |  |
| Gnai2 |  |
| Aco2 |  |
| Acadl |  |
| Bcap31 |  |
| Ndufs2 |  |
| Aldh6a1 |  |
| Vcp |  |
| Atp1b1 |  |
| Ddost |  |
| Mccc2 |  |
