# Supplementary material for: The ultrastructural and proteomic analysis of mitochondria‐associated endoplasmic reticulum membrane in the midbrain of a Parkinson's disease mouse model
Source: Aging Cell. 2024 Nov 29;24(4):e14436. doi: 10.1111/acel.14436 (PMC11984660; doi:10.1111/acel.14436)
Supplement: Supplementary file 20 — Table S14. GSEA data for consensus MAM proteins in MAM proteomics. [file ACEL-24-e14436-s015.docx]

**Supplementary Table 14** **GSEA data for consensus MAM proteins in MAM proteomics**

| ID | Description | Set size | Enrichment Score | NES | p value | Core enrichment genes |
| --- | --- | --- | --- | --- | --- | --- |
| BP Terms | | | | | | |
| GO:0006898 | receptor-mediated endocytosis | 12 | 0.625 | 1.555 | 0.054 | Ap2b1/Cltc/Apoe/Rab21/Canx |
| GO:0034641 | cellular nitrogen compound metabolic process | 74 | -0.321 | -1.262 | 0.093 | Pa2g4/Stoml2/Rplp2/Ass1/Aldh9a1/Prdx6/Cct8/Maob/Eef1a1/Cyfip1/Tmbim6/Cs/Acaa2/Aldh1l1/Aldh6a1/Actn4 |
| GO:0030036 | actin cytoskeleton organization | 16 | -0.499 | -1.411 | 0.093 | Cfl1/Cdc42bpb/Lrp1/Arpc4/Myh9/Actn1/Cyfip1/Actn4 |
| GO:0006816 | calcium ion transport | 13 | -0.519 | -1.368 | 0.099 | Stoml2/Tmbim6/Atp1b1 |
| GO:0010035 | response to inorganic substance | 11 | -0.571 | -1.431 | 0.100 | Park7/Cfl1/Slc25a12/Sigmar1/Vcp/Ass1/Clic4 |
| GO:0019216 | regulation of lipid metabolic process | 11 | 0.579 | 1.413 | 0.111 | Atp1a1/Idh1/Apoe |
| GO:0034976 | response to endoplasmic reticulum stress | 18 | 0.498 | 1.353 | 0.113 | P4hb/Erp29/Erp44/Hyou1/Hsp90b1/Canx/Uggt1/Pdia6/Calr |
| GO:0019725 | cellular homeostasis | 27 | 0.443 | 1.329 | 0.118 | Atp1a1/P4hb/Atp6v1a/Abcb7/Apoe/Erp44 |
| GO:0008610 | lipid biosynthetic process | 10 | 0.588 | 1.403 | 0.118 | Atp1a1/Idh1/Apoe |
| GO:0071396 | cellular response to lipid | 10 | 0.585 | 1.395 | 0.118 | Atp1a1/Mtdh/Ndufa13/Msn/Calr |
| GO:0007015 | actin filament organization | 10 | -0.553 | -1.342 | 0.121 | Cfl1/Arpc4/Actn1/Cyfip1/Actn4 |
| GO:0006725 | cellular aromatic compound metabolic process | 55 | -0.337 | -1.239 | 0.124 | Prdx6/Cct8/Maob/Tmbim6/Cs/Acaa2/Aldh1l1/Aldh6a1/Actn4 |
| GO:0051640 | organelle localization | 10 | -0.542 | -1.315 | 0.133 | Picalm/Itgb1/Tmed10/Cfl1/Myh9/Dnm2/Alb/Actn4 |
| GO:0009725 | response to hormone | 18 | 0.492 | 1.337 | 0.135 | Hadha/Atp1a1/Idh1 |
| GO:0006396 | RNA processing | 10 | -0.538 | -1.307 | 0.136 | Pa2g4/Prdx6/Tmbim6 |
| GO:0033993 | response to lipid | 13 | 0.537 | 1.373 | 0.137 | Atp1a1/Mtdh/Idh1 |
| GO:0072359 | circulatory system development | 13 | 0.538 | 1.376 | 0.137 | Ap2b1/Mtdh/Apoe/Mthfd1/Jup |
| GO:0009892 | negative regulation of metabolic process | 33 | 0.410 | 1.289 | 0.147 | Cltc/Atp1a1/Mtdh/Erp29/Apoe |
| GO:0002520 | immune system development | 11 | -0.514 | -1.287 | 0.155 | Gnas/Picalm/Rps14/Eef2/Ap2a2/Hspa9/Myh9/Actn1 |
| GO:0031329 | regulation of cellular catabolic process | 16 | 0.491 | 1.312 | 0.169 | Mtdh/Idh1/Apoe |
| GO:0072593 | reactive oxygen species metabolic process | 17 | -0.444 | -1.271 | 0.169 | Park7/Prdx5/Gstp1/Dnm2/Cycs/Ass1/Prdx6/Eef1a1 |
| GO:0030097 | hemopoiesis | 10 | -0.520 | -1.263 | 0.170 | Gnas/Picalm/Rps14/Eef2/Ap2a2/Hspa9/Myh9/Actn1 |
| GO:0048534 | hematopoietic or lymphoid organ development | 10 | -0.520 | -1.263 | 0.170 | Gnas/Picalm/Rps14/Eef2/Ap2a2/Hspa9/Myh9/Actn1 |
| GO:0055085 | transmembrane transport | 37 | 0.386 | 1.227 | 0.172 | Atp1a1/Tst/Atp6v1a/Abcb7/Ndufs7/Atp5h |
| GO:0009890 | negative regulation of biosynthetic process | 22 | 0.446 | 1.267 | 0.183 | Cltc/Atp1a1/Mtdh/Apoe |
| GO:0014070 | response to organic cyclic compound | 18 | 0.469 | 1.275 | 0.185 | Atp1a1/Idh1/Jup/Hsp90b1/Msn/Calr |
| GO:0080134 | regulation of response to stress | 18 | 0.468 | 1.272 | 0.185 | P4hb/Erp29/Apoe/Hyou1 |
| GO:0098660 | inorganic ion transmembrane transport | 20 | 0.445 | 1.244 | 0.185 | Atp1a1/Atp6v1a/Ndufs7/Atp5h |
| GO:0006979 | response to oxidative stress | 17 | 0.468 | 1.260 | 0.192 | P4hb/Idh1/Apoe |
| GO:0098662 | inorganic cation transmembrane transport | 19 | 0.451 | 1.243 | 0.195 | Atp1a1/Atp6v1a/Ndufs7/Atp5h |
| GO:0044085 | cellular component biogenesis | 58 | -0.309 | -1.154 | 0.198 | Cfl1/Hspa9/Lrp1/Arpc4/Sigmar1/Rab1b/Vcp/Dnm2/Vdac3/Rab14/Pa2g4/Stoml2/Ndufa9/Actn1/Samm50/Aldh9a1/Prdx6/Rab32/Cct8/Cyfip1/Actn4 |
| GO:0005975 | carbohydrate metabolic process | 16 | 0.473 | 1.263 | 0.206 | Cltc/Idh1/Dlat/Pgm1 |
| GO:2001242 | regulation of intrinsic apoptotic signaling pathway | 10 | 0.522 | 1.244 | 0.206 | P4hb/Erp29/Hyou1 |
| GO:0051336 | regulation of hydrolase activity | 27 | -0.369 | -1.189 | 0.207 | Picalm/Serpina3k/Gna13/Itgb1/Tmed10/Hsp90ab1/Park7/Bcap31/Lrp1/Apoc1/Vcp/Dnm2/Cycs/Tmbim6/Atp1b1 |
| GO:0048518 | positive regulation of biological process | 71 | -0.296 | -1.152 | 0.207 | Pdia3/Bcap31/Lrp1/Arpc4/Gstp1/Myh9/Rab1b/Vcp/Vapa/Dnm2/Pa2g4/Cycs/Stoml2/Ass1/Ppib/Prdx6/Decr1/Cct8/Maob/Eef1a1/Cyfip1/Alb/Atp1b1/Actn4 |
| GO:0006875 | cellular metal ion homeostasis | 19 | 0.444 | 1.225 | 0.208 | Atp1a1/Atp6v1a/Abcb7/Apoe |
| GO:0001568 | blood vessel development | 11 | 0.507 | 1.238 | 0.216 | Ap2b1/Mtdh/Apoe/Jup |
| GO:0001944 | vasculature development | 11 | 0.507 | 1.238 | 0.216 | Ap2b1/Mtdh/Apoe/Jup |
| GO:0090407 | organophosphate biosynthetic process | 13 | 0.481 | 1.229 | 0.219 | Idh1/Dlat/Mthfd1/Atp5h |
| GO:0006812 | cation transport | 29 | 0.390 | 1.189 | 0.222 | Cltc/Atp1a1/Atp6v1a/Ndufs7/Atp5h |
| GO:0010605 | negative regulation of macromolecule metabolic process | 26 | 0.400 | 1.183 | 0.226 | Cltc/Mtdh/Erp29/Apoe |
| GO:0022607 | cellular component assembly | 53 | -0.312 | -1.150 | 0.228 | Cfl1/Hspa9/Lrp1/Arpc4/Sigmar1/Rab1b/Vcp/Dnm2/Vdac3/Rab14/Stoml2/Ndufa9/Actn1/Samm50/Aldh9a1/Prdx6/Rab32/Cct8/Cyfip1/Actn4 |
| GO:0097193 | intrinsic apoptotic signaling pathway | 14 | 0.474 | 1.226 | 0.233 | P4hb/Erp29/Hyou1 |
| GO:0046483 | heterocycle metabolic process | 54 | -0.310 | -1.137 | 0.237 | Prdx6/Cct8/Tmbim6/Cs/Acaa2/Aldh1l1/Aldh6a1/Actn4 |
| GO:0055065 | metal ion homeostasis | 22 | 0.423 | 1.201 | 0.238 | Atp1a1/Atp6v1a/Abcb7/Apoe |
| GO:0050789 | regulation of biological process | 115 | -0.268 | -1.108 | 0.240 | Ap2a2/Cfl1/Cdc42bpb/Hspa9/Pdia3/Bcap31/Lrp1/Arpc4/Gstp1/Myh9/Sigmar1/Pgk1/Rab1b/Apoc1/Vcp/Vapa/Dnm2/Vdac3/Rab14/Pa2g4/Cycs/Stoml2/Actn1/Gnb1/Ass1/Ppib/Clic4/Prdx6/Decr1/Cct8/Maob/Eef1a1/Cyfip1/Tmbim6/Alb/Atp1b1/Acaa2/Actn4 |
| GO:0008202 | steroid metabolic process | 10 | 0.494 | 1.178 | 0.247 | Atp1a1/Apoe/Apobr |
| GO:0000165 | MAPK cascade | 11 | 0.491 | 1.200 | 0.248 | Erp29/Apoe |
| GO:0043408 | regulation of MAPK cascade | 11 | 0.491 | 1.200 | 0.248 | Erp29/Apoe |
| GO:0098655 | cation transmembrane transport | 21 | 0.422 | 1.195 | 0.248 | Atp1a1/Atp6v1a/Ndufs7/Atp5h |
| GO:0040011 | locomotion | 21 | -0.395 | -1.178 | 0.256 | Cfl1/Cdc42bpb/Lrp1/Myh9/Ppib/Clic4/Cyfip1/Actn4 |
| GO:0071407 | cellular response to organic cyclic compound | 15 | 0.453 | 1.192 | 0.261 | Atp1a1/Jup/Hsp90b1/Msn/Calr |
| GO:0006996 | organelle organization | 68 | -0.288 | -1.109 | 0.264 | Hsp90ab1/Rab18/Park7/Immt/Ndufb6/Abcd3/Cfl1/Cdc42bpb/Bcap31/Lrp1/Arpc4/Myh9/Rab1b/Vcp/Vapa/Dnm2/Vdac3/Rab14/Stoml2/Ndufa9/Actn1/Samm50/Rab32/Cct8/Cyfip1/Acaa2/Actn4 |
| GO:0050821 | protein stabilization | 11 | -0.466 | -1.165 | 0.266 | Hsp90ab1/Park7/Ppib/Cct8/Atp1b1 |
| GO:0048878 | chemical homeostasis | 28 | 0.375 | 1.139 | 0.267 | Atp1a1/Atp6v1a/Abcb7/Apoe/Acox1 |
| GO:0006873 | cellular ion homeostasis | 20 | 0.417 | 1.166 | 0.272 | Atp1a1/Atp6v1a/Abcb7/Apoe |
| GO:0030003 | cellular cation homeostasis | 20 | 0.417 | 1.166 | 0.272 | Atp1a1/Atp6v1a/Abcb7/Apoe |
| GO:0055082 | cellular chemical homeostasis | 20 | 0.417 | 1.166 | 0.272 | Atp1a1/Atp6v1a/Abcb7/Apoe |
| GO:0044419 | biological process involved in interspecies interaction between organisms | 16 | 0.438 | 1.169 | 0.281 | P4hb/Mtdh/Apoe |
| GO:0007155 | cell adhesion | 21 | -0.383 | -1.143 | 0.289 | Cfl1/Lrp1/Gstp1/Myh9/Dnm2/Actn1/Ass1/Atp1b1/Actn4 |
| GO:0050801 | ion homeostasis | 23 | 0.397 | 1.144 | 0.289 | Atp1a1/Atp6v1a/Abcb7/Apoe |
| GO:0055080 | cation homeostasis | 23 | 0.397 | 1.144 | 0.289 | Atp1a1/Atp6v1a/Abcb7/Apoe |
| GO:0098771 | inorganic ion homeostasis | 23 | 0.397 | 1.144 | 0.289 | Atp1a1/Atp6v1a/Abcb7/Apoe |
| GO:0051130 | positive regulation of cellular component organization | 24 | -0.367 | -1.136 | 0.300 | Cfl1/Lrp1/Arpc4/Vcp/Dnm2/Cct8/Eef1a1/Cyfip1/Actn4 |
| GO:0042592 | homeostatic process | 47 | 0.338 | 1.117 | 0.302 | Atp1a1/P4hb/Atp6v1a/Abcb7/Apoe/Erp44/Acox1/Mthfd1 |
| GO:0001816 | cytokine production | 12 | -0.444 | -1.143 | 0.303 | Tmed10/Hsp90ab1/Park7/Lrp1/Gstp1/Stoml2 |
| GO:0009894 | regulation of catabolic process | 20 | 0.408 | 1.141 | 0.309 | Mtdh/Idh1/Apoe |
| GO:0034220 | ion transmembrane transport | 34 | 0.355 | 1.118 | 0.312 | Atp1a1/Tst/Atp6v1a/Ndufs7/Atp5h |
| GO:0031331 | positive regulation of cellular catabolic process | 11 | 0.460 | 1.123 | 0.316 | Mtdh/Apoe |
| GO:0051049 | regulation of transport | 42 | -0.310 | -1.104 | 0.317 | Clic4/Maob/Cyfip1/Tmbim6/Atp1b1/Acaa2/Actn4 |
| GO:0006629 | lipid metabolic process | 29 | 0.361 | 1.100 | 0.318 | Hadha/Atp1a1/Idh1/Apoe/Acox1 |
| GO:0009896 | positive regulation of catabolic process | 12 | 0.451 | 1.123 | 0.330 | Mtdh/Apoe/Ndufa13/Msn |
| GO:0060429 | epithelium development | 11 | 0.451 | 1.102 | 0.333 | Tst/Mthfd1/Msn/Rala |
| GO:0010558 | negative regulation of macromolecule biosynthetic process | 15 | 0.421 | 1.107 | 0.342 | Cltc/Mtdh/Rpl10/Ndufa13/Calr |
| GO:0097435 | supramolecular fiber organization | 15 | -0.392 | -1.092 | 0.343 | Cfl1/Arpc4/Actn1/Cyfip1/Actn4 |
| GO:0022610 | biological adhesion | 22 | -0.362 | -1.094 | 0.345 | Cfl1/Lrp1/Gstp1/Myh9/Dnm2/Actn1/Ass1/Atp1b1/Actn4 |
| GO:0007005 | mitochondrion organization | 25 | -0.351 | -1.095 | 0.345 | Park7/Immt/Ndufb6/Bcap31/Dnm2/Stoml2/Ndufa9/Samm50/Rab32/Acaa2 |
| GO:0009719 | response to endogenous stimulus | 32 | 0.347 | 1.086 | 0.346 | Hadha/Atp1a1/Idh1/Apoe |
| GO:0080135 | regulation of cellular response to stress | 13 | 0.431 | 1.103 | 0.353 | P4hb/Erp29/Hyou1 |
| GO:0031327 | negative regulation of cellular biosynthetic process | 15 | 0.416 | 1.094 | 0.353 | Atp1a1/Mtdh/Rpl10/Ndufa13/Calr |
| GO:0045859 | regulation of protein kinase activity | 10 | 0.448 | 1.068 | 0.355 | Erp29/Apoe |
| GO:0010033 | response to organic substance | 58 | 0.312 | 1.078 | 0.360 | Hadha/Atp1a1/P4hb/Mtdh/Idh1/Apoe/Erp44/Jup/Hsp90b1/Canx/Uggt1/Pdia6/Ndufa13/Msn/Calr |
| GO:0006874 | cellular calcium ion homeostasis | 16 | -0.379 | -1.074 | 0.368 | Immt/Bcap31/Lrp1/Stoml2/Gnb1/Tmbim6/Atp1b1 |
| GO:0072503 | cellular divalent inorganic cation homeostasis | 16 | -0.379 | -1.074 | 0.368 | Immt/Bcap31/Lrp1/Stoml2/Gnb1/Tmbim6/Atp1b1 |
| GO:0031647 | regulation of protein stability | 14 | -0.399 | -1.077 | 0.376 | Hsp90ab1/Park7/Ddost/Ppib/Cct8/Atp1b1 |
| GO:1902531 | regulation of intracellular signal transduction | 28 | 0.352 | 1.069 | 0.377 | P4hb/Mtdh/Erp29/Apoe/Hyou1 |
| GO:0044403 | biological process involved in symbiotic interaction | 11 | 0.431 | 1.052 | 0.382 | P4hb/Apoe |
| GO:0035239 | tube morphogenesis | 13 | 0.418 | 1.068 | 0.386 | Mtdh/Apoe/Mthfd1/Jup |
| GO:0035295 | tube development | 13 | 0.418 | 1.068 | 0.386 | Mtdh/Apoe/Mthfd1/Jup |
| GO:0048522 | positive regulation of cellular process | 64 | -0.271 | -1.032 | 0.389 | Pdia3/Bcap31/Lrp1/Arpc4/Gstp1/Myh9/Rab1b/Vcp/Dnm2/Pa2g4/Cycs/Stoml2/Ass1/Prdx6/Cct8/Maob/Eef1a1/Cyfip1/Atp1b1/Actn4 |
| GO:0016477 | cell migration | 19 | -0.360 | -1.056 | 0.394 | Cfl1/Cdc42bpb/Lrp1/Myh9/Ppib/Clic4/Actn4 |
| GO:0048870 | cell motility | 19 | -0.360 | -1.056 | 0.394 | Cfl1/Cdc42bpb/Lrp1/Myh9/Ppib/Clic4/Actn4 |
| GO:0051674 | localization of cell | 19 | -0.360 | -1.056 | 0.394 | Cfl1/Cdc42bpb/Lrp1/Myh9/Ppib/Clic4/Actn4 |
| GO:0007010 | cytoskeleton organization | 21 | -0.352 | -1.050 | 0.396 | Cfl1/Cdc42bpb/Lrp1/Arpc4/Myh9/Vcp/Actn1/Cyfip1/Actn4 |
| GO:0006897 | endocytosis | 16 | 0.396 | 1.059 | 0.396 | Ap2b1/Cltc/Apoe/Rab21/Canx |
| GO:0044087 | regulation of cellular component biogenesis | 17 | -0.370 | -1.059 | 0.399 | Park7/Cfl1/Lrp1/Arpc4/Rab1b/Vcp/Dnm2/Vdac3/Cyfip1 |
| GO:0006811 | ion transport | 80 | 0.282 | 1.026 | 0.400 | Ap2b1/Cltc/Atp1a1/Tst/Atp6v1a/Erp29/Apoe/Ndufs7/Atp5h/Rab21/Jup/Apobr/Rrbp1/Hsp90b1 |
| GO:0006790 | sulfur compound metabolic process | 15 | 0.398 | 1.048 | 0.406 | Tst/Idh1/Dlat/Mthfd1 |
| GO:1901360 | organic cyclic compound metabolic process | 63 | -0.273 | -1.034 | 0.407 | Prdx6/Cct8/Maob/Tmbim6/Cs/Acaa2/Aldh1l1/Aldh6a1/Actn4 |
| GO:0051172 | negative regulation of nitrogen compound metabolic process | 23 | 0.362 | 1.042 | 0.407 | Cltc/Mtdh/Apoe/Rpl10/Ndufa13/Calr |
| GO:0016032 | viral process | 10 | 0.432 | 1.031 | 0.410 | P4hb/Apoe |
| GO:0009628 | response to abiotic stimulus | 19 | 0.385 | 1.060 | 0.412 | Atp1a1/P4hb/Atp6v1a/Hyou1/Jup/Hsp90b1 |
| GO:0006457 | protein folding | 13 | 0.408 | 1.044 | 0.412 | P4hb/Erp44/Hsp90b1/Canx/Calr |
| GO:0006810 | transport | 91 | 0.281 | 1.033 | 0.412 | Ap2b1/Cltc/Atp1a1/Tst/Atp6v1a/Abcb7/Erp29/Apoe/Ndufs7/Hyou1/Atp5h/Rab21/Jup/Apobr/Rrbp1/Hsp90b1/Canx |
| GO:0051234 | establishment of localization | 91 | 0.281 | 1.033 | 0.412 | Ap2b1/Cltc/Atp1a1/Tst/Atp6v1a/Abcb7/Erp29/Apoe/Ndufs7/Hyou1/Atp5h/Rab21/Jup/Apobr/Rrbp1/Hsp90b1/Canx |
| GO:0051094 | positive regulation of developmental process | 19 | 0.383 | 1.055 | 0.415 | P4hb/Mtdh/Apoe/Rab21/Jup |
| GO:1901137 | carbohydrate derivative biosynthetic process | 17 | 0.390 | 1.051 | 0.421 | Cltc/Dlat/Atp5h/Uggt1/Acat1 |
| GO:0006139 | nucleobase-containing compound metabolic process | 51 | -0.278 | -1.016 | 0.423 | Prdx6/Cct8/Tmbim6/Cs/Acaa2/Aldh6a1/Actn4 |
| GO:0008219 | cell death | 45 | -0.279 | -1.011 | 0.423 | Hspa9/Pdia3/Bcap31/Lrp1/Gstp1/Sigmar1/Vapa/Pa2g4/Cycs/Gnb1/Eef1a1/Tmbim6/Alb/Acaa2/Actn4 |
| GO:0016042 | lipid catabolic process | 17 | 0.388 | 1.045 | 0.427 | Hadha/Idh1/Apoe/Acox1 |
| GO:0051241 | negative regulation of multicellular organismal process | 14 | 0.394 | 1.020 | 0.429 | Atp1a1/Apoe/Jup |
| GO:0006508 | proteolysis | 28 | -0.310 | -1.016 | 0.430 | Pacsin3/Picalm/Afg3l2/Pcyox1/Serpina3k/Uqcrc2/Tmed10/Erlin2/Hsp90ab1/Park7/Cfl1/Bcap31/Myh9/Pgk1/Vcp/Cycs/Stoml2/Lap3 |
| GO:0055074 | calcium ion homeostasis | 17 | -0.358 | -1.025 | 0.432 | Gnas/Atp2a2/Afg3l2/Gna13/Immt/Bcap31/Lrp1/Stoml2/Gnb1/Tmbim6/Atp1b1 |
| GO:0072507 | divalent inorganic cation homeostasis | 17 | -0.358 | -1.025 | 0.432 | Gnas/Atp2a2/Afg3l2/Gna13/Immt/Bcap31/Lrp1/Stoml2/Gnb1/Tmbim6/Atp1b1 |
| GO:0009888 | tissue development | 15 | 0.385 | 1.013 | 0.432 | Tst/Mthfd1/Msn/Rala |
| GO:0051345 | positive regulation of hydrolase activity | 18 | -0.356 | -1.034 | 0.433 | Gnas/Picalm/Gna13/Itgb1/Hsp90ab1/Bcap31/Vcp/Dnm2/Cycs/Atp1b1 |
| GO:2001233 | regulation of apoptotic signaling pathway | 13 | 0.393 | 1.006 | 0.438 | P4hb/Erp29/Hyou1 |
| GO:0033043 | regulation of organelle organization | 19 | -0.351 | -1.029 | 0.441 | Cfl1/Lrp1/Arpc4/Rab1b/Dnm2/Vdac3/Cct8/Cyfip1/Acaa2 |
| GO:0016043 | cellular component organization | 91 | -0.250 | -1.010 | 0.441 | Hsp90ab1/Rab18/Prkcsh/Park7/Immt/Ndufb6/Abcd3/Cfl1/Cdc42bpb/Hspa9/Bcap31/Lrp1/Arpc4/Myh9/Sigmar1/Rab1b/Vcp/Vapa/Dnm2/Vdac3/Rab14/Stoml2/Ndufa9/Actn1/Samm50/Aldh9a1/Prdx6/Rab32/Cct8/Eef1a1/Cyfip1/Acaa2/Actn4 |
| GO:0010720 | positive regulation of cell development | 11 | 0.408 | 0.997 | 0.444 | P4hb/Apoe/Rab21 |
| GO:0006357 | regulation of transcription by RNA polymerase II | 10 | 0.424 | 1.012 | 0.445 | Mtdh/Jup/Rpl10/Calr |
| GO:0006366 | transcription by RNA polymerase II | 10 | 0.424 | 1.012 | 0.445 | Mtdh/Jup/Rpl10/Calr |
| GO:0006351 | transcription, DNA-templated | 18 | 0.378 | 1.028 | 0.448 | Mtdh/Apoe/Jup/Rpl10/Ndufa13/Calr |
| GO:0006355 | regulation of transcription, DNA-templated | 18 | 0.378 | 1.028 | 0.448 | Mtdh/Apoe/Jup/Rpl10/Ndufa13/Calr |
| GO:1901362 | organic cyclic compound biosynthetic process | 35 | 0.321 | 1.016 | 0.455 | Atp1a1/Mtdh/Apoe/Dlat/Mthfd1/Atp5h/Jup/Rpl10 |
| GO:0034330 | cell junction organization | 17 | -0.350 | -1.003 | 0.458 | Cfl1/Lrp1/Vcp/Actn1/Cyfip1/Actn4 |
| GO:0007166 | cell surface receptor signaling pathway | 24 | -0.321 | -0.992 | 0.464 | Acsl1/Hsp90ab1/Park7/Pdia3/Lrp1/Vcp/Dnm2/Rab14/Stoml2/Cyfip1/Actn4 |
| GO:0062197 | cellular response to chemical stress | 10 | 0.415 | 0.990 | 0.464 | P4hb |
| GO:0030334 | regulation of cell migration | 12 | -0.382 | -0.982 | 0.472 | Clic4/Actn4 |
| GO:0016054 | organic acid catabolic process | 19 | -0.338 | -0.990 | 0.474 | Decr1/Acaa2/Aldh1l1/Aldh6a1 |
| GO:0046395 | carboxylic acid catabolic process | 19 | -0.338 | -0.990 | 0.474 | Decr1/Acaa2/Aldh1l1/Aldh6a1 |
| GO:0071840 | cellular component organization or biogenesis | 96 | -0.243 | -0.988 | 0.476 | Hsp90ab1/Rab18/Prkcsh/Park7/Immt/Ndufb6/Abcd3/Cfl1/Cdc42bpb/Hspa9/Bcap31/Lrp1/Arpc4/Myh9/Sigmar1/Rab1b/Vcp/Vapa/Dnm2/Vdac3/Rab14/Pa2g4/Stoml2/Ndufa9/Actn1/Samm50/Aldh9a1/Prdx6/Rab32/Cct8/Eef1a1/Cyfip1/Acaa2/Actn4 |
| GO:0006164 | purine nucleotide biosynthetic process | 10 | 0.409 | 0.976 | 0.478 | Dlat/Mthfd1/Atp5h |
| GO:0009165 | nucleotide biosynthetic process | 10 | 0.409 | 0.976 | 0.478 | Dlat/Mthfd1/Atp5h |
| GO:0072522 | purine-containing compound biosynthetic process | 10 | 0.409 | 0.976 | 0.478 | Dlat/Mthfd1/Atp5h |
| GO:1901293 | nucleoside phosphate biosynthetic process | 10 | 0.409 | 0.976 | 0.478 | Dlat/Mthfd1/Atp5h |
| GO:0009060 | aerobic respiration | 14 | 0.376 | 0.974 | 0.481 | Idh1/Dlat/Ndufs7/Cox4i1 |
| GO:0032787 | monocarboxylic acid metabolic process | 23 | 0.342 | 0.985 | 0.481 | Hadha/Idh1/Dlat/Acox1 |
| GO:1990542 | mitochondrial transmembrane transport | 13 | 0.376 | 0.961 | 0.490 | Tst/Atp5h |
| GO:0030030 | cell projection organization | 26 | -0.304 | -0.975 | 0.492 | Rpl4/Picalm/Afg3l2/Itgb1/Acsl4/Hsp90ab1/Prkcsh/Cfl1/Lrp1/Myh9/Vapa/Dnm2/Vdac3/Prdx6/Eef1a1/Cyfip1 |
| GO:0120036 | plasma membrane bounded cell projection organization | 26 | -0.304 | -0.975 | 0.492 | Rpl4/Picalm/Afg3l2/Itgb1/Acsl4/Hsp90ab1/Prkcsh/Cfl1/Lrp1/Myh9/Vapa/Dnm2/Vdac3/Prdx6/Eef1a1/Cyfip1 |
| GO:0010256 | endomembrane system organization | 11 | 0.394 | 0.962 | 0.494 | Cltc |
| GO:0001932 | regulation of protein phosphorylation | 12 | 0.387 | 0.963 | 0.494 | Erp29/Apoe |
| GO:0050790 | regulation of catalytic activity | 35 | -0.285 | -0.975 | 0.499 | Picalm/Serpina3k/Gna13/Itgb1/Tmed10/Acsl1/Hsp90ab1/Park7/Prdx5/Bcap31/Lrp1/Gstp1/Apoc1/Vcp/Dnm2/Cycs/Eef1a1/Tmbim6/Atp1b1 |
| GO:0140352 | export from cell | 18 | 0.361 | 0.981 | 0.505 | Atp1a1/Erp29/Apoe/Rab21 |
| GO:0030100 | regulation of endocytosis | 10 | -0.405 | -0.983 | 0.507 | Lrp1/Dnm2/Actn4 |
| GO:0035556 | intracellular signal transduction | 37 | -0.275 | -0.949 | 0.519 | Cyfip1/Tmbim6/Atp1b1/Actn4 |
| GO:0019220 | regulation of phosphate metabolic process | 19 | 0.348 | 0.959 | 0.522 | Idh1/Erp29/Apoe |
| GO:0051174 | regulation of phosphorus metabolic process | 19 | 0.348 | 0.959 | 0.522 | Idh1/Erp29/Apoe |
| GO:0030258 | lipid modification | 14 | 0.361 | 0.934 | 0.522 | Hadha/Apoe/Acox1 |
| GO:0097190 | apoptotic signaling pathway | 18 | 0.354 | 0.961 | 0.527 | P4hb/Erp29/Hyou1 |
| GO:0046486 | glycerolipid metabolic process | 12 | 0.377 | 0.939 | 0.528 | Hadha/Apoe/Apobr |
| GO:0070482 | response to oxygen levels | 11 | 0.381 | 0.929 | 0.528 | P4hb/Atp6v1a/Hyou1/Hsp90b1 |
| GO:0022402 | cell cycle process | 13 | 0.364 | 0.930 | 0.529 | Cltc |
| GO:0044242 | cellular lipid catabolic process | 15 | 0.355 | 0.934 | 0.530 | Hadha/Idh1/Acox1 |
| GO:0001666 | response to hypoxia | 10 | -0.396 | -0.961 | 0.532 | Gnb1/Tmbim6/Atp1b1/Acaa2 |
| GO:0036293 | response to decreased oxygen levels | 10 | -0.396 | -0.961 | 0.532 | Gnb1/Tmbim6/Atp1b1/Acaa2 |
| GO:0051051 | negative regulation of transport | 13 | -0.361 | -0.951 | 0.533 | Maob/Cyfip1/Tmbim6 |
| GO:0042981 | regulation of apoptotic process | 35 | -0.276 | -0.946 | 0.534 | Tmbim6/Alb/Acaa2/Actn4 |
| GO:0043067 | regulation of programmed cell death | 35 | -0.276 | -0.946 | 0.534 | Tmbim6/Alb/Acaa2/Actn4 |
| GO:0032879 | regulation of localization | 54 | -0.266 | -0.974 | 0.537 | Apoc1/Vcp/Dnm2/Rab14/Actn1/Clic4/Cct8/Maob/Cyfip1/Tmbim6/Atp1b1/Acaa2/Actn4 |
| GO:0010941 | regulation of cell death | 38 | -0.272 | -0.942 | 0.541 | Eef1a1/Tmbim6/Alb/Acaa2/Actn4 |
| GO:0019219 | regulation of nucleobase-containing compound metabolic process | 24 | -0.301 | -0.931 | 0.546 | Vcp/Pa2g4/Prdx6/Cct8/Tmbim6/Actn4 |
| GO:0051240 | positive regulation of multicellular organismal process | 26 | 0.317 | 0.938 | 0.548 | Atp1a1/Mtdh/Apoe/Rab21/Jup |
| GO:0000003 | reproduction | 13 | -0.355 | -0.936 | 0.549 | Itgb1/Acsl4/Hsp90ab1/Park7/Bcap31/Myh9/Clic4/Cct8 |
| GO:0022414 | reproductive process | 13 | -0.355 | -0.936 | 0.549 | Itgb1/Acsl4/Hsp90ab1/Park7/Bcap31/Myh9/Clic4/Cct8 |
| GO:1904951 | positive regulation of establishment of protein localization | 10 | -0.390 | -0.947 | 0.550 | Tmed10/Acsl4/Hsp90ab1/Bcap31/Lrp1/Cct8 |
| GO:0042221 | response to chemical | 75 | 0.266 | 0.963 | 0.553 | Hadha/Atp1a1/P4hb/Atp6v1a/Mtdh/Idh1/Apoe/Erp44/Hyou1 |
| GO:0055086 | nucleobase-containing small molecule metabolic process | 22 | -0.309 | -0.932 | 0.554 | Pgk1/Vcp/Stoml2/Cs/Acaa2/Aldh6a1 |
| GO:0040012 | regulation of locomotion | 13 | -0.352 | -0.929 | 0.557 | Clic4/Actn4 |
| GO:2000145 | regulation of cell motility | 13 | -0.352 | -0.929 | 0.557 | Clic4/Actn4 |
| GO:0051050 | positive regulation of transport | 27 | -0.289 | -0.931 | 0.563 | Itgb1/Tmed10/Acsl4/Acsl1/Hsp90ab1/Park7/Cfl1/Bcap31/Lrp1/Dnm2/Atp1b1/Actn4 |
| GO:0051716 | cellular response to stimulus | 80 | -0.238 | -0.945 | 0.565 | Cfl1/Cdc42bpb/Hspa9/Pdia3/Bcap31/Lrp1/Gstp1/Sigmar1/Pgk1/Vcp/Dnm2/Rab14/Stoml2/Gnb1/Ass1/Ppib/Clic4/Prdx6/Rab32/Eef1a1/Cyfip1/Tmbim6/Alb/Atp1b1/Acaa2/Actn4 |
| GO:0071345 | cellular response to cytokine stimulus | 13 | -0.350 | -0.924 | 0.566 | Acsl1/Hsp90ab1/Cfl1/Pdia3/Actn4 |
| GO:0030155 | regulation of cell adhesion | 14 | -0.340 | -0.919 | 0.567 | Cfl1/Lrp1/Gstp1/Dnm2/Ass1/Actn4 |
| GO:0072659 | protein localization to plasma membrane | 10 | 0.378 | 0.901 | 0.567 | Cltc/Jup |
| GO:1990778 | protein localization to cell periphery | 10 | 0.378 | 0.901 | 0.567 | Cltc/Jup |
| GO:0009966 | regulation of signal transduction | 36 | -0.269 | -0.931 | 0.568 | Cyfip1/Tmbim6/Acaa2/Actn4 |
| GO:0010876 | lipid localization | 14 | -0.340 | -0.918 | 0.569 | Got2/Itgb1/Acsl4/Acsl1/Abcd3/Lrp1/Slc25a12/Sigmar1/Apoc1/Stoml2 |
| GO:0022604 | regulation of cell morphogenesis | 14 | -0.340 | -0.918 | 0.569 | Cyfip1/Actn4 |
| GO:0071705 | nitrogen compound transport | 48 | 0.283 | 0.938 | 0.569 | Ap2b1/Cltc/Tst/Erp29/Apoe/Rab21/Jup/Rrbp1/Hsp90b1 |
| GO:0051046 | regulation of secretion | 13 | 0.351 | 0.898 | 0.573 | Erp29/Apoe/Rab21 |
| GO:1903530 | regulation of secretion by cell | 13 | 0.351 | 0.898 | 0.573 | Erp29/Apoe/Rab21 |
| GO:0019637 | organophosphate metabolic process | 26 | 0.312 | 0.924 | 0.574 | Hadha/Idh1/Dlat/Mthfd1/Atp5h |
| GO:0051223 | regulation of protein transport | 14 | 0.343 | 0.889 | 0.575 | Erp29/Apoe/Jup |
| GO:0090087 | regulation of peptide transport | 14 | 0.343 | 0.889 | 0.575 | Erp29/Apoe/Jup |
| GO:0031324 | negative regulation of cellular metabolic process | 27 | 0.305 | 0.915 | 0.575 | Atp1a1/Mtdh/Apoe |
| GO:0009889 | regulation of biosynthetic process | 39 | 0.290 | 0.930 | 0.576 | Cltc/Atp1a1/Mtdh/Idh1/Apoe |
| GO:0051402 | neuron apoptotic process | 10 | 0.375 | 0.895 | 0.577 | Apoe/Hyou1 |
| GO:0048589 | developmental growth | 17 | -0.324 | -0.926 | 0.577 | Gnas/Rpl4/Picalm/Afg3l2/Itgb1/Hsp90ab1/Gpd2/Cfl1/Lrp1/Dnm2/Ppib/Clic4/Cyfip1 |
| GO:1902533 | positive regulation of intracellular signal transduction | 16 | 0.337 | 0.900 | 0.578 | Mtdh/Erp29/Apoe |
| GO:0035966 | response to topologically incorrect protein | 11 | 0.363 | 0.887 | 0.583 | Erp44/Canx/Uggt1/Pdia6/Calr |
| GO:0033554 | cellular response to stress | 39 | -0.262 | -0.915 | 0.585 | Erlin2/Hsp90ab1/Park7/Prdx5/Cfl1/Hspa9/Pdia3/Bcap31/Lrp1/Gstp1/Sigmar1/Pgk1/Vcp/Stoml2/Gnb1/Ass1/Tmbim6/Alb/Acaa2 |
| GO:0060627 | regulation of vesicle-mediated transport | 17 | -0.321 | -0.918 | 0.586 | Lrp1/Dnm2/Actn4 |
| GO:0051179 | localization | 98 | 0.254 | 0.947 | 0.586 | Ap2b1/Cltc/Atp1a1/Tst/Atp6v1a/Abcb7/Erp29/Apoe/Ndufs7/Hyou1/Atp5h/Rab21/Jup/Apobr/Rrbp1/Hsp90b1/Canx |
| GO:0007267 | cell-cell signaling | 18 | -0.315 | -0.915 | 0.587 | Itgb1/Acsl4/Park7/Cfl1/Lrp1/Vcp/Vdac3/Maob/Cyfip1 |
| GO:0052547 | regulation of peptidase activity | 12 | -0.345 | -0.887 | 0.588 | Picalm/Serpina3k/Tmed10/Park7/Bcap31/Vcp/Cycs |
| GO:0052548 | regulation of endopeptidase activity | 12 | -0.345 | -0.887 | 0.588 | Picalm/Serpina3k/Tmed10/Park7/Bcap31/Vcp/Cycs |
| GO:2000026 | regulation of multicellular organismal development | 16 | 0.330 | 0.881 | 0.589 | Mtdh/Apoe/Rab21/Jup |
| GO:0060284 | regulation of cell development | 12 | -0.344 | -0.884 | 0.592 | Cyfip1/Actn4 |
| GO:0030029 | actin filament-based process | 19 | -0.314 | -0.920 | 0.592 | Cfl1/Cdc42bpb/Lrp1/Arpc4/Myh9/Actn1/Cyfip1/Actn4 |
| GO:0090304 | nucleic acid metabolic process | 31 | -0.276 | -0.924 | 0.595 | Prdx6/Cct8/Tmbim6/Actn4 |
| GO:0098656 | anion transmembrane transport | 13 | 0.345 | 0.881 | 0.596 | Tst |
| GO:0007049 | cell cycle | 16 | 0.327 | 0.875 | 0.600 | Cltc |
| GO:0034097 | response to cytokine | 15 | -0.321 | -0.893 | 0.600 | Serpina3k/Rplp0/Acsl1/Hsp90ab1/Ddost/Cfl1/Pdia3/Actn4 |
| GO:0043648 | dicarboxylic acid metabolic process | 12 | -0.342 | -0.880 | 0.600 | Mdh1/Ass1/Cs/Aldh1l1 |
| GO:0051338 | regulation of transferase activity | 12 | 0.348 | 0.866 | 0.607 | Erp29/Apoe |
| GO:0044282 | small molecule catabolic process | 23 | -0.294 | -0.897 | 0.609 | Decr1/Acaa2/Aldh1l1/Aldh6a1 |
| GO:0007154 | cell communication | 63 | -0.244 | -0.923 | 0.612 | Cfl1/Cdc42bpb/Pdia3/Bcap31/Lrp1/Gstp1/Vcp/Dnm2/Vdac3/Rab14/Stoml2/Gnb1/Maob/Cyfip1/Tmbim6/Alb/Atp1b1/Acaa2/Actn4 |
| GO:0043549 | regulation of kinase activity | 11 | 0.351 | 0.857 | 0.615 | Erp29/Apoe |
| GO:0051173 | positive regulation of nitrogen compound metabolic process | 36 | -0.262 | -0.905 | 0.615 | Itgb1/Eef2/Acsl1/Hsp90ab1/Park7/Cfl1/Bcap31/Lrp1/Myh9/Rab1b/Vcp/Dnm2/Cycs/Ass1/Prdx6/Cct8/Maob |
| GO:0072657 | protein localization to membrane | 14 | 0.330 | 0.856 | 0.616 | Cltc/Apoe/Jup |
| GO:0006412 | translation | 21 | -0.295 | -0.882 | 0.618 | Pa2g4/Rplp2/Eef1a1/Cyfip1 |
| GO:0043043 | peptide biosynthetic process | 21 | -0.295 | -0.882 | 0.618 | Pa2g4/Rplp2/Eef1a1/Cyfip1 |
| GO:0051128 | regulation of cellular component organization | 45 | -0.254 | -0.922 | 0.620 | Lrp1/Arpc4/Myh9/Rab1b/Vcp/Dnm2/Vdac3/Cct8/Eef1a1/Cyfip1/Acaa2/Actn4 |
| GO:0006066 | alcohol metabolic process | 12 | 0.345 | 0.858 | 0.624 | Idh1/Apoe/Apobr |
| GO:0009893 | positive regulation of metabolic process | 47 | -0.250 | -0.913 | 0.628 | Acsl1/Hsp90ab1/Park7/Prdx5/Scd1/Cfl1/Bcap31/Lrp1/Gstp1/Myh9/Rab1b/Vcp/Dnm2/Cycs/Stoml2/Ass1/Prdx6/Decr1/Cct8/Maob |
| GO:1901214 | regulation of neuron death | 11 | 0.348 | 0.849 | 0.628 | Apoe/Hyou1 |
| GO:0010646 | regulation of cell communication | 41 | -0.253 | -0.892 | 0.628 | Cyfip1/Tmbim6/Acaa2/Actn4 |
| GO:0023051 | regulation of signaling | 41 | -0.253 | -0.892 | 0.628 | Cyfip1/Tmbim6/Acaa2/Actn4 |
| GO:0032870 | cellular response to hormone stimulus | 12 | 0.342 | 0.851 | 0.629 | Atp1a1 |
| GO:0006886 | intracellular protein transport | 30 | 0.292 | 0.898 | 0.632 | Ap2b1/Cltc/Rab21/Jup/Hsp90b1/Ndufa13/Calr |
| GO:0048583 | regulation of response to stimulus | 42 | -0.251 | -0.894 | 0.632 | Cyfip1/Tmbim6/Acaa2/Actn4 |
| GO:0032774 | RNA biosynthetic process | 19 | -0.303 | -0.889 | 0.633 | Tmbim6/Actn4 |
| GO:0097659 | nucleic acid-templated transcription | 19 | -0.303 | -0.889 | 0.633 | Tmbim6/Actn4 |
| GO:1903506 | regulation of nucleic acid-templated transcription | 19 | -0.303 | -0.889 | 0.633 | Tmbim6/Actn4 |
| GO:2001141 | regulation of RNA biosynthetic process | 19 | -0.303 | -0.889 | 0.633 | Tmbim6/Actn4 |
| GO:0031399 | regulation of protein modification process | 16 | 0.316 | 0.844 | 0.635 | Erp29/Apoe/Hsp90b1 |
| GO:0006928 | movement of cell or subcellular component | 27 | -0.273 | -0.880 | 0.636 | Cfl1/Cdc42bpb/Lrp1/Myh9/Actn1/Ppib/Clic4/Cyfip1/Actn4 |
| GO:0006520 | cellular amino acid metabolic process | 11 | 0.346 | 0.844 | 0.637 | Tst/Mthfd1/Acat1/Ahcy/Mccc2 |
| GO:0045892 | negative regulation of transcription, DNA-templated | 11 | 0.342 | 0.836 | 0.639 | Mtdh/Rpl10/Ndufa13/Calr |
| GO:0051253 | negative regulation of RNA metabolic process | 11 | 0.342 | 0.836 | 0.639 | Mtdh/Rpl10/Ndufa13/Calr |
| GO:1902679 | negative regulation of RNA biosynthetic process | 11 | 0.342 | 0.836 | 0.639 | Mtdh/Rpl10/Ndufa13/Calr |
| GO:1903507 | negative regulation of nucleic acid-templated transcription | 11 | 0.342 | 0.836 | 0.639 | Mtdh/Rpl10/Ndufa13/Calr |
| GO:0032880 | regulation of protein localization | 24 | 0.299 | 0.872 | 0.642 | Cltc/Erp29/Apoe |
| GO:0009891 | positive regulation of biosynthetic process | 17 | -0.307 | -0.878 | 0.645 | Eef2/Hsp90ab1/Park7/Prdx5/Vcp/Dnm2/Stoml2/Ass1/Cct8 |
| GO:0043270 | positive regulation of ion transport | 17 | -0.304 | -0.869 | 0.651 | Itgb1/Tmed10/Acsl4/Acsl1/Hsp90ab1/Cfl1/Bcap31/Lrp1/Dnm2/Atp1b1 |
| GO:0006915 | apoptotic process | 41 | -0.250 | -0.882 | 0.655 | Pdia3/Bcap31/Lrp1/Gstp1/Sigmar1/Pa2g4/Cycs/Gnb1/Tmbim6/Alb/Acaa2/Actn4 |
| GO:0012501 | programmed cell death | 41 | -0.250 | -0.882 | 0.655 | Pdia3/Bcap31/Lrp1/Gstp1/Sigmar1/Pa2g4/Cycs/Gnb1/Tmbim6/Alb/Acaa2/Actn4 |
| GO:0030001 | metal ion transport | 14 | -0.319 | -0.861 | 0.661 | Stoml2/Tmbim6/Atp1b1 |
| GO:0050877 | nervous system process | 10 | -0.354 | -0.859 | 0.663 | Vdac3/Gnb1/Cyfip1 |
| GO:0051252 | regulation of RNA metabolic process | 20 | -0.293 | -0.864 | 0.664 | Pa2g4/Prdx6/Tmbim6/Actn4 |
| GO:0043086 | negative regulation of catalytic activity | 12 | -0.327 | -0.842 | 0.665 | Picalm/Serpina3k/Tmed10/Park7/Prdx5/Gstp1/Apoc1/Tmbim6 |
| GO:0044089 | positive regulation of cellular component biogenesis | 10 | -0.353 | -0.858 | 0.665 | Park7/Cfl1/Arpc4/Vcp/Dnm2/Cyfip1 |
| GO:0031323 | regulation of cellular metabolic process | 61 | -0.239 | -0.897 | 0.666 | Acadl/Pacsin3/Picalm/Cisd1/Serpina3k/Rps14/Itgb1/Eef2/Tmed10/Acsl4/Erlin2/Acsl1/Hsp90ab1/Park7/Prdx5/Scd1/Cfl1/Bcap31/Lrp1/Gstp1/Myh9/Rab1b/Vcp/Dnm2/Pa2g4/Cycs/Stoml2/Ass1/Prdx6/Cct8/Maob/Eef1a1/Cyfip1/Tmbim6/Actn4 |
| GO:0045597 | positive regulation of cell differentiation | 15 | 0.314 | 0.826 | 0.669 | P4hb/Apoe/Rab21 |
| GO:0045934 | negative regulation of nucleobase-containing compound metabolic process | 12 | 0.331 | 0.825 | 0.670 | Mtdh/Rpl10/Ndufa13/Calr |
| GO:0070997 | neuron death | 12 | 0.332 | 0.826 | 0.670 | Apoe/Hyou1 |
| GO:2000113 | negative regulation of cellular macromolecule biosynthetic process | 12 | 0.331 | 0.825 | 0.670 | Mtdh/Rpl10/Ndufa13/Calr |
| GO:0019222 | regulation of metabolic process | 69 | -0.233 | -0.900 | 0.670 | Bcap31/Lrp1/Gstp1/Myh9/Rab1b/Apoc1/Vcp/Dnm2/Pa2g4/Cycs/Stoml2/Ass1/Prdx6/Decr1/Cct8/Maob/Eef1a1/Cyfip1/Tmbim6/Atp1b1/Actn4 |
| GO:0044281 | small molecule metabolic process | 55 | 0.258 | 0.889 | 0.673 | Hadha/Tst/Idh1/Apoe/Dlat/Acox1/Pgm1/Mthfd1/Atp5h |
| GO:0051171 | regulation of nitrogen compound metabolic process | 52 | -0.243 | -0.895 | 0.676 | Bcap31/Lrp1/Gstp1/Myh9/Rab1b/Vcp/Dnm2/Pa2g4/Cycs/Ass1/Prdx6/Cct8/Maob/Cyfip1/Tmbim6/Actn4 |
| GO:0016070 | RNA metabolic process | 26 | -0.266 | -0.851 | 0.677 | Prdx6/Tmbim6/Actn4 |
| GO:0031325 | positive regulation of cellular metabolic process | 42 | -0.244 | -0.870 | 0.680 | Acsl1/Hsp90ab1/Park7/Scd1/Cfl1/Bcap31/Lrp1/Gstp1/Myh9/Rab1b/Vcp/Dnm2/Cycs/Stoml2/Ass1/Prdx6/Cct8/Maob |
| GO:0022603 | regulation of anatomical structure morphogenesis | 22 | 0.294 | 0.837 | 0.681 | P4hb/Mtdh/Apoe/Rab21/Jup |
| GO:0016192 | vesicle-mediated transport | 36 | 0.273 | 0.870 | 0.683 | Ap2b1/Cltc/Erp29/Apoe/Hyou1/Rab21/Canx/Msn/Calr/Rala |
| GO:0044255 | cellular lipid metabolic process | 26 | 0.285 | 0.845 | 0.687 | Hadha/Idh1/Apoe/Acox1 |
| GO:0031328 | positive regulation of cellular biosynthetic process | 16 | -0.296 | -0.837 | 0.688 | Eef2/Hsp90ab1/Park7/Vcp/Dnm2/Stoml2/Ass1/Cct8 |
| GO:0031175 | neuron projection development | 21 | -0.283 | -0.845 | 0.689 | Rpl4/Picalm/Afg3l2/Itgb1/Acsl4/Hsp90ab1/Prkcsh/Cfl1/Lrp1/Vapa/Dnm2/Eef1a1/Cyfip1 |
| GO:0040007 | growth | 21 | -0.283 | -0.845 | 0.689 | Gnas/Rpl4/Picalm/Afg3l2/Itgb1/Acsl4/Hsp90ab1/Gpd2/Cfl1/Lrp1/Dnm2/Ppib/Clic4/Cyfip1 |
| GO:0048666 | neuron development | 21 | -0.283 | -0.845 | 0.689 | Rpl4/Picalm/Afg3l2/Itgb1/Acsl4/Hsp90ab1/Prkcsh/Cfl1/Lrp1/Vapa/Dnm2/Eef1a1/Cyfip1 |
| GO:0071702 | organic substance transport | 55 | 0.253 | 0.872 | 0.690 | Ap2b1/Cltc/Tst/Erp29/Apoe/Rab21/Jup/Apobr/Rrbp1/Hsp90b1 |
| GO:0009967 | positive regulation of signal transduction | 19 | -0.286 | -0.838 | 0.693 | Pdia3/Bcap31/Lrp1/Vcp/Cyfip1/Actn4 |
| GO:0010556 | regulation of macromolecule biosynthetic process | 30 | -0.253 | -0.848 | 0.694 | Pa2g4/Stoml2/Cct8/Cyfip1/Tmbim6/Actn4 |
| GO:0048646 | anatomical structure formation involved in morphogenesis | 13 | 0.314 | 0.803 | 0.694 | Mtdh/Mthfd1/Jup |
| GO:0008152 | metabolic process | 160 | 0.246 | 0.918 | 0.695 | Ap2b1/Cltc/Hadha/Atp1a1/Tst/P4hb/Atp6v1a/Mtdh/Idh1/Erp29/Apoe/Dlat/Erp44/Ndufs7/Acox1/Cox4i1/Pgm1/Mthfd1/Atp5h |
| GO:0044271 | cellular nitrogen compound biosynthetic process | 51 | -0.241 | -0.880 | 0.698 | Vcp/Dnm2/Pa2g4/Stoml2/Rplp2/Ass1/Cct8/Eef1a1/Cyfip1/Tmbim6/Actn4 |
| GO:0002376 | immune system process | 34 | -0.250 | -0.845 | 0.699 | Rps14/Itgb1/Eef2/Park7/Ddost/Ap2a2/Hspa9/Bcap31/Lrp1/Myh9/Stoml2/Actn1/Ppib/Rab32/Tmbim6 |
| GO:0046907 | intracellular transport | 44 | 0.265 | 0.863 | 0.701 | Ap2b1/Cltc/Erp29/Hyou1/Atp5h/Rab21/Jup/Hsp90b1/Ndufa13/Msn/Calr |
| GO:0006464 | cellular protein modification process | 30 | 0.272 | 0.837 | 0.704 | P4hb/Erp29/Apoe |
| GO:0036211 | protein modification process | 30 | 0.272 | 0.837 | 0.704 | P4hb/Erp29/Apoe |
| GO:0043412 | macromolecule modification | 30 | 0.272 | 0.837 | 0.704 | P4hb/Erp29/Apoe |
| GO:0000904 | cell morphogenesis involved in differentiation | 20 | -0.284 | -0.837 | 0.706 | Cfl1/Lrp1/Myh9/Dnm2/Actn1/Clic4/Cyfip1/Actn4 |
| GO:0044248 | cellular catabolic process | 50 | 0.254 | 0.857 | 0.709 | Cltc/Hadha/Mtdh/Idh1/Apoe/Acox1 |
| GO:0001934 | positive regulation of protein phosphorylation | 10 | 0.335 | 0.799 | 0.718 | Erp29/Phb/Phb2/Cd81/Rps3/Gnas/Itgb1/Acsl1/Hsp90ab1/Park7 |
| GO:0042127 | regulation of cell population proliferation | 16 | 0.294 | 0.785 | 0.719 | Apoe/Jup/Calr/Ndrg2/Phb/Phb2/Cd81/Gnai2/Rps3/Ptprf/Itpr1/Rps9/Gnas/Gna13/Itgb1 |
| GO:0034654 | nucleobase-containing compound biosynthetic process | 31 | 0.272 | 0.837 | 0.723 | Mtdh/Apoe/Dlat/Mthfd1/Atp5h/Jup/Rpl10/Ndufa13/Calr/Acat1 |
| GO:0018130 | heterocycle biosynthetic process | 33 | 0.263 | 0.828 | 0.732 | Mtdh/Apoe/Dlat/Mthfd1/Atp5h/Jup/Rpl10/Ndufa13/Calr/Acat1 |
| GO:0019438 | aromatic compound biosynthetic process | 33 | 0.263 | 0.828 | 0.732 | Mtdh/Apoe/Dlat/Mthfd1/Atp5h/Jup/Rpl10/Ndufa13/Calr/Acat1 |
| GO:0006793 | phosphorus metabolic process | 57 | 0.244 | 0.847 | 0.738 | Hadha/Idh1/Erp29/Apoe/Dlat/Cox4i1/Mthfd1/Atp5h |
| GO:0006796 | phosphate-containing compound metabolic process | 57 | 0.244 | 0.847 | 0.738 | Hadha/Idh1/Erp29/Apoe/Dlat/Cox4i1/Mthfd1/Atp5h |
| GO:0006869 | lipid transport | 13 | -0.304 | -0.802 | 0.739 | Got2/Itgb1/Acsl4/Acsl1/Abcd3/Lrp1/Slc25a12/Sigmar1/Apoc1 |
| GO:0051641 | cellular localization | 63 | -0.226 | -0.857 | 0.740 | Abcd3/Ap2a2/Cfl1/Hspa9/Bcap31/Lrp1/Myh9/Rab1b/Vcp/Vapa/Dnm2/Rab14/Stoml2/Samm50/Rab32/Cct8/Cyfip1/Tmbim6/Alb/Atp1b1/Actn4 |
| GO:0009605 | response to external stimulus | 28 | 0.266 | 0.808 | 0.745 | Atp1a1/Mtdh/Apoe |
| GO:0048468 | cell development | 33 | -0.247 | -0.826 | 0.745 | Vapa/Dnm2/Actn1/Clic4/Eef1a1/Cyfip1/Actn4 |
| GO:0048584 | positive regulation of response to stimulus | 22 | -0.270 | -0.814 | 0.745 | Pdia3/Bcap31/Lrp1/Vcp/Stoml2/Cyfip1/Actn4 |
| GO:0065008 | regulation of biological quality | 85 | 0.230 | 0.847 | 0.748 | Ap2b1/Atp1a1/P4hb/Atp6v1a/Abcb7/Apoe/Erp44/Acox1/Mthfd1/Rab21/Jup |
| GO:0007165 | signal transduction | 54 | -0.235 | -0.861 | 0.749 | Cfl1/Cdc42bpb/Pdia3/Bcap31/Lrp1/Gstp1/Vcp/Dnm2/Rab14/Stoml2/Gnb1/Cyfip1/Tmbim6/Atp1b1/Acaa2/Actn4 |
| GO:0044092 | negative regulation of molecular function | 15 | -0.288 | -0.802 | 0.749 | Atp2a2/Picalm/Serpina3k/Tmed10/Park7/Prdx5/Gstp1/Apoc1/Tmbim6 |
| GO:0048193 | Golgi vesicle transport | 11 | -0.320 | -0.801 | 0.749 | Bcap31/Rab1b/Vcp/Vapa/Dnm2/Rab14 |
| GO:0072594 | establishment of protein localization to organelle | 13 | 0.299 | 0.765 | 0.750 | Ap2b1 |
| GO:0006839 | mitochondrial transport | 18 | -0.270 | -0.785 | 0.752 | Bcap31/Slc25a12/Stoml2/Samm50/Slc25a1/Acaa2 |
| GO:0070887 | cellular response to chemical stimulus | 56 | -0.233 | -0.861 | 0.753 | Cfl1/Hspa9/Pdia3/Lrp1/Gstp1/Sigmar1/Pgk1/Vcp/Dnm2/Rab14/Gnb1/Ppib/Clic4/Prdx6/Eef1a1/Cyfip1/Tmbim6/Acaa2/Actn4 |
| GO:0018193 | peptidyl-amino acid modification | 11 | 0.314 | 0.766 | 0.756 | P4hb |
| GO:0006950 | response to stress | 60 | -0.225 | -0.844 | 0.758 | Erlin2/Hsp90ab1/Park7/Prdx5/Scd1/Cfl1/Hspa9/Pdia3/Bcap31/Lrp1/Gstp1/Myh9/Sigmar1/Pgk1/Vcp/Vdac3/Rab14/Stoml2/Gnb1/Ass1/Prdx6/Tmbim6/Alb/Atp1b1/Acaa2 |
| GO:0003008 | system process | 17 | 0.279 | 0.752 | 0.760 | Atp1a1/Apoe/Jup |
| GO:0010647 | positive regulation of cell communication | 22 | -0.266 | -0.803 | 0.761 | Itgb1/Acsl4/Hsp90ab1/Park7/Pdia3/Bcap31/Lrp1/Vcp/Cyfip1/Actn4 |
| GO:0023056 | positive regulation of signaling | 22 | -0.266 | -0.803 | 0.761 | Itgb1/Acsl4/Hsp90ab1/Park7/Pdia3/Bcap31/Lrp1/Vcp/Cyfip1/Actn4 |
| GO:0006635 | fatty acid beta-oxidation | 12 | 0.306 | 0.763 | 0.762 | Hadha/Acox1 |
| GO:0009062 | fatty acid catabolic process | 12 | 0.306 | 0.763 | 0.762 | Hadha/Acox1 |
| GO:0019395 | fatty acid oxidation | 12 | 0.306 | 0.763 | 0.762 | Hadha/Acox1 |
| GO:0034440 | lipid oxidation | 12 | 0.306 | 0.763 | 0.762 | Hadha/Acox1 |
| GO:0072329 | monocarboxylic acid catabolic process | 12 | 0.306 | 0.763 | 0.762 | Hadha/Acox1 |
| GO:0070201 | regulation of establishment of protein localization | 15 | 0.288 | 0.757 | 0.763 | Erp29/Apoe/Jup |
| GO:0065007 | biological regulation | 127 | 0.230 | 0.870 | 0.764 | Ap2b1/Cltc/Atp1a1/P4hb/Atp6v1a/Mtdh/Idh1/Abcb7/Erp29/Apoe/Erp44/Hyou1/Acox1/Mthfd1/Rab21/Jup/Rpl10 |
| GO:0015031 | protein transport | 44 | 0.254 | 0.827 | 0.765 | Ap2b1/Cltc/Erp29/Apoe/Rab21/Jup/Rrbp1/Hsp90b1 |
| GO:0015833 | peptide transport | 44 | 0.254 | 0.827 | 0.765 | Ap2b1/Cltc/Erp29/Apoe/Rab21/Jup/Rrbp1/Hsp90b1 |
| GO:0042886 | amide transport | 44 | 0.254 | 0.827 | 0.765 | Ap2b1/Cltc/Erp29/Apoe/Rab21/Jup/Rrbp1/Hsp90b1 |
| GO:0032940 | secretion by cell | 16 | 0.282 | 0.754 | 0.765 | Erp29/Apoe/Rab21 |
| GO:0080090 | regulation of primary metabolic process | 58 | 0.239 | 0.829 | 0.766 | Cltc/Atp1a1/Mtdh/Idh1/Erp29/Apoe |
| GO:0010629 | negative regulation of gene expression | 11 | 0.311 | 0.760 | 0.769 | Erp29/Apoe |
| GO:0050793 | regulation of developmental process | 30 | -0.237 | -0.796 | 0.770 | Ap2a2/Cfl1/Hspa9/Lrp1/Myh9/Pgk1/Dnm2/Rab14/Pa2g4/Ppib/Cyfip1/Actn4 |
| GO:0061024 | membrane organization | 21 | -0.262 | -0.782 | 0.773 | Hspa8/Tmem30a/Sptbn1/Atp2a2/Pacsin3/Picalm/Afg3l2/Itgb1/Tmed10/Immt/Myh9/Vapa/Dnm2/Samm50/Acaa2 |
| GO:0019538 | protein metabolic process | 75 | -0.220 | -0.863 | 0.774 | Pacsin3/Rpl4/Picalm/Afg3l2/Pcyox1/Serpina3k/Rps14/Rplp0/Uqcrc2/Itgb1/Eef2/Tmed10/Rpl18/Erlin2/Acsl1/Hsp90ab1/Prkcsh/Park7/Ddost/Cfl1/Cdc42bpb/Hspa9/Bcap31/Lrp1/Gstp1/Myh9/Pgk1/Rab1b/Apoc1/Vcp/Ganab/Pa2g4/Cycs/Stoml2/Rplp2/Ppib/Eef1a1/Cyfip1/Lap3 |
| GO:0006082 | organic acid metabolic process | 40 | 0.248 | 0.800 | 0.774 | Hadha/Tst/Idh1/Dlat/Acox1/Mthfd1 |
| GO:0019752 | carboxylic acid metabolic process | 40 | 0.248 | 0.800 | 0.774 | Hadha/Tst/Idh1/Dlat/Acox1/Mthfd1 |
| GO:0043436 | oxoacid metabolic process | 40 | 0.248 | 0.800 | 0.774 | Hadha/Tst/Idh1/Dlat/Acox1/Mthfd1 |
| GO:0045321 | leukocyte activation | 11 | -0.310 | -0.777 | 0.774 | Prdx1/Rps3/Ncstn/Ddost/Myh9/Stoml2 |
| GO:0046649 | lymphocyte activation | 11 | -0.310 | -0.777 | 0.774 | Prdx1/Rps3/Ncstn/Ddost/Myh9/Stoml2 |
| GO:0030154 | cell differentiation | 49 | -0.230 | -0.839 | 0.775 | Rpl4/Picalm/Afg3l2/Gna13/Rps14/Itgb1/Eef2/Acsl4/Hsp90ab1/Prkcsh/Scd1/Ap2a2/Cfl1/Hspa9/Lrp1/Myh9/Vapa/Dnm2/Pa2g4/Actn1/Clic4/Rab32/Eef1a1/Cyfip1/Aldh6a1/Actn4 |
| GO:0048869 | cellular developmental process | 49 | -0.230 | -0.839 | 0.775 | Rpl4/Picalm/Afg3l2/Gna13/Rps14/Itgb1/Eef2/Acsl4/Hsp90ab1/Prkcsh/Scd1/Ap2a2/Cfl1/Hspa9/Lrp1/Myh9/Vapa/Dnm2/Pa2g4/Actn1/Clic4/Rab32/Eef1a1/Cyfip1/Aldh6a1/Actn4 |
| GO:0023052 | signaling | 61 | -0.222 | -0.834 | 0.775 | Cfl1/Cdc42bpb/Pdia3/Bcap31/Lrp1/Gstp1/Vcp/Dnm2/Vdac3/Rab14/Stoml2/Gnb1/Maob/Cyfip1/Tmbim6/Atp1b1/Acaa2/Actn4 |
| GO:0009653 | anatomical structure morphogenesis | 33 | 0.253 | 0.796 | 0.776 | P4hb/Mtdh/Apoe/Mthfd1/Rab21/Jup |
| GO:0045595 | regulation of cell differentiation | 18 | -0.264 | -0.768 | 0.778 | Ap2a2/Hspa9/Lrp1/Dnm2/Pa2g4/Cyfip1/Actn4 |
| GO:0043604 | amide biosynthetic process | 26 | -0.244 | -0.782 | 0.780 | Pa2g4/Rplp2/Ass1/Eef1a1/Cyfip1 |
| GO:0016053 | organic acid biosynthetic process | 10 | -0.309 | -0.751 | 0.789 | Acadl/Got2/Erlin2/Park7/Scd1/Abcd3/Ass1 |
| GO:0046394 | carboxylic acid biosynthetic process | 10 | -0.309 | -0.751 | 0.789 | Acadl/Got2/Erlin2/Park7/Scd1/Abcd3/Ass1 |
| GO:0006091 | generation of precursor metabolites and energy | 38 | 0.248 | 0.796 | 0.792 | Idh1/Dlat/Ndufs7/Acox1/Cox4i1/Atp5h |
| GO:0006820 | anion transport | 60 | 0.234 | 0.818 | 0.792 | Ap2b1/Cltc/Tst/Erp29/Apoe/Rab21/Jup/Apobr/Rrbp1/Hsp90b1 |
| GO:0010243 | response to organonitrogen compound | 24 | 0.258 | 0.751 | 0.793 | Hadha/Apoe/Jup/Hsp90b1/Uggt1 |
| GO:1901698 | response to nitrogen compound | 24 | 0.258 | 0.751 | 0.793 | Hadha/Apoe/Jup/Hsp90b1/Uggt1 |
| GO:1901565 | organonitrogen compound catabolic process | 27 | -0.240 | -0.774 | 0.793 | Aldh1l1/Aldh6a1 |
| GO:0050794 | regulation of cellular process | 108 | -0.206 | -0.844 | 0.796 | Ap2a2/Cfl1/Cdc42bpb/Hspa9/Pdia3/Bcap31/Lrp1/Arpc4/Gstp1/Myh9/Sigmar1/Rab1b/Vcp/Dnm2/Vdac3/Rab14/Pa2g4/Cycs/Stoml2/Actn1/Gnb1/Ass1/Clic4/Prdx6/Cct8/Maob/Eef1a1/Cyfip1/Tmbim6/Alb/Atp1b1/Acaa2/Actn4 |
| GO:1901615 | organic hydroxy compound metabolic process | 14 | 0.285 | 0.739 | 0.797 | Idh1/Apoe/Apobr |
| GO:0071310 | cellular response to organic substance | 42 | 0.248 | 0.801 | 0.797 | Atp1a1/P4hb/Mtdh/Jup/Hsp90b1/Canx/Uggt1/Pdia6/Ndufa13/Msn/Calr |
| GO:0071417 | cellular response to organonitrogen compound | 12 | -0.290 | -0.746 | 0.799 | Cfl1/Lrp1/Dnm2/Cyfip1 |
| GO:1901699 | cellular response to nitrogen compound | 12 | -0.290 | -0.746 | 0.799 | Cfl1/Lrp1/Dnm2/Cyfip1 |
| GO:0009150 | purine ribonucleotide metabolic process | 18 | -0.260 | -0.755 | 0.800 | Pgk1/Vcp/Stoml2/Cs/Acaa2 |
| GO:0009259 | ribonucleotide metabolic process | 18 | -0.260 | -0.755 | 0.800 | Pgk1/Vcp/Stoml2/Cs/Acaa2 |
| GO:0019693 | ribose phosphate metabolic process | 18 | -0.260 | -0.755 | 0.800 | Pgk1/Vcp/Stoml2/Cs/Acaa2 |
| GO:0050803 | regulation of synapse structure or activity | 10 | -0.299 | -0.725 | 0.813 | Cfl1/Vcp/Dnm2/Cyfip1 |
| GO:0033036 | macromolecule localization | 59 | 0.227 | 0.792 | 0.817 | Ap2b1/Cltc/Tst/Erp29/Apoe/Rab21/Jup/Apobr/Rrbp1/Hsp90b1 |
| GO:0031326 | regulation of cellular biosynthetic process | 34 | 0.245 | 0.773 | 0.819 | Atp1a1/Mtdh/Idh1/Apoe/Jup/Rpl10 |
| GO:0032970 | regulation of actin filament-based process | 11 | 0.294 | 0.718 | 0.820 | Atp1a1/Jup |
| GO:0043066 | negative regulation of apoptotic process | 25 | 0.250 | 0.738 | 0.820 | Mtdh/Apoe/Hyou1/Rpl10/Hsp90b1 |
| GO:0043069 | negative regulation of programmed cell death | 25 | 0.250 | 0.738 | 0.820 | Mtdh/Apoe/Hyou1/Rpl10/Hsp90b1 |
| GO:0009056 | catabolic process | 57 | 0.226 | 0.783 | 0.820 | Cltc/Hadha/Mtdh/Idh1/Apoe/Acox1 |
| GO:0000902 | cell morphogenesis | 23 | -0.247 | -0.752 | 0.821 | Cfl1/Lrp1/Myh9/Dnm2/Actn1/Clic4/Cyfip1/Actn4 |
| GO:0051603 | proteolysis involved in cellular protein catabolic process | 10 | 0.303 | 0.722 | 0.821 | Apoe/Hsp90b1/Uggt1 |
| GO:0050896 | response to stimulus | 99 | 0.221 | 0.825 | 0.824 | Hadha/Atp1a1/P4hb/Atp6v1a/Mtdh/Idh1/Erp29/Apoe/Erp44/Hyou1 |
| GO:0030162 | regulation of proteolysis | 19 | -0.254 | -0.745 | 0.824 | Pacsin3/Picalm/Serpina3k/Tmed10/Hsp90ab1/Park7/Cfl1/Bcap31/Myh9/Vcp/Cycs |
| GO:1901576 | organic substance biosynthetic process | 73 | 0.222 | 0.797 | 0.825 | Cltc/Atp1a1/Mtdh/Idh1/Apoe/Dlat/Mthfd1/Atp5h/Jup/Rpl10 |
| GO:0051270 | regulation of cellular component movement | 16 | -0.260 | -0.737 | 0.825 | Actn1/Clic4/Actn4 |
| GO:0009058 | biosynthetic process | 78 | 0.219 | 0.795 | 0.825 | Cltc/Atp1a1/Mtdh/Idh1/Apoe/Dlat/Acox1/Mthfd1/Atp5h/Jup/Rpl10 |
| GO:0060548 | negative regulation of cell death | 27 | -0.233 | -0.752 | 0.826 | Hsp90ab1/Park7/Prdx5/Hspa9/Lrp1/Gstp1/Pa2g4/Tmbim6/Alb/Acaa2 |
| GO:0044260 | cellular macromolecule metabolic process | 80 | 0.222 | 0.807 | 0.827 | Ap2b1/Cltc/P4hb/Mtdh/Erp29/Apoe/Erp44 |
| GO:0044237 | cellular metabolic process | 150 | 0.221 | 0.833 | 0.827 | Ap2b1/Cltc/Hadha/Atp1a1/Tst/P4hb/Mtdh/Idh1/Erp29/Apoe/Dlat/Erp44/Ndufs7/Acox1/Cox4i1/Mthfd1/Atp5h/Jup/Rpl10/Apobr |
| GO:0030163 | protein catabolic process | 18 | 0.258 | 0.701 | 0.828 | Apoe/Hsp90b1/Uggt1/Ndufa13/Msn |
| GO:0042325 | regulation of phosphorylation | 14 | 0.277 | 0.718 | 0.828 | Erp29/Apoe |
| GO:0045184 | establishment of protein localization | 46 | 0.237 | 0.782 | 0.829 | Ap2b1/Cltc/Erp29/Apoe/Rab21/Jup/Rrbp1/Hsp90b1 |
| GO:1902532 | negative regulation of intracellular signal transduction | 12 | 0.284 | 0.706 | 0.831 | Apoe/Hyou1 |
| GO:0006468 | protein phosphorylation | 17 | 0.261 | 0.704 | 0.835 | Erp29/Apoe |
| GO:0006575 | cellular modified amino acid metabolic process | 10 | -0.291 | -0.706 | 0.836 | Ass1/Aldh9a1/Aldh1l1 |
| GO:0045333 | cellular respiration | 25 | 0.247 | 0.729 | 0.837 | Idh1/Dlat/Ndufs7/Cox4i1 |
| GO:0060255 | regulation of macromolecule metabolic process | 54 | -0.216 | -0.791 | 0.842 | Bcap31/Lrp1/Gstp1/Myh9/Rab1b/Vcp/Pa2g4/Cycs/Stoml2/Prdx6/Cct8/Cyfip1/Tmbim6/Atp1b1/Actn4 |
| GO:0044093 | positive regulation of molecular function | 30 | 0.234 | 0.719 | 0.844 | Mtdh/Erp29/Apoe |
| GO:0009968 | negative regulation of signal transduction | 21 | -0.238 | -0.711 | 0.844 | Lrp1/Gstp1/Vcp/Dnm2/Tmbim6/Acaa2 |
| GO:0010648 | negative regulation of cell communication | 21 | -0.238 | -0.711 | 0.844 | Lrp1/Gstp1/Vcp/Dnm2/Tmbim6/Acaa2 |
| GO:0023057 | negative regulation of signaling | 21 | -0.238 | -0.711 | 0.844 | Lrp1/Gstp1/Vcp/Dnm2/Tmbim6/Acaa2 |
| GO:0022904 | respiratory electron transport chain | 15 | -0.261 | -0.727 | 0.845 | Sdhb/Cox5a/Sdha/Uqcrfs1/Park7/Ndufb6/Slc25a12/Cycs/Uqcrc1 |
| GO:0009059 | macromolecule biosynthetic process | 48 | -0.213 | -0.778 | 0.846 | Pa2g4/Stoml2/Rplp2/Cct8/Eef1a1/Cyfip1/Tmbim6/Actn4 |
| GO:0009792 | embryo development ending in birth or egg hatching | 11 | -0.285 | -0.713 | 0.847 | Gna13/Itgb1/Acsl4/Prkcsh/Cfl1/Myh9/Atp1b1 |
| GO:0048638 | regulation of developmental growth | 11 | -0.284 | -0.710 | 0.847 | Gnas/Rpl4/Picalm/Afg3l2/Cfl1/Lrp1/Dnm2/Ppib/Cyfip1 |
| GO:1901135 | carbohydrate derivative metabolic process | 31 | 0.235 | 0.724 | 0.848 | Cltc/Dlat/Erp44/Atp5h/Uggt1/Acat1/Ahcy/Mccc2/Mfn1/Eno1/Rpn1 |
| GO:0044283 | small molecule biosynthetic process | 18 | -0.247 | -0.718 | 0.848 | Got2/Erlin2/Park7/Scd1/Gpd2/Abcd3/Pgk1/Vcp/Ndufa9/Ass1 |
| GO:0070925 | organelle assembly | 15 | 0.262 | 0.689 | 0.850 | Cltc |
| GO:1901701 | cellular response to oxygen-containing compound | 24 | -0.232 | -0.719 | 0.852 | Park7/Prdx5/Cfl1/Pdia3/Lrp1/Gstp1/Sigmar1/Vcp/Dnm2/Cyfip1 |
| GO:0008150 | biological_process | 200 | -0.278 | -0.761 | 0.853 | Actn1/Rplp2/Gnb1/Samm50/Ass1/Ppib/Aldh9a1/Clic4/Prdx6/Decr1/Rab32/Cct8/Slc25a1/Maob/Eef1a1/Bdh1/Uqcrc1/Aco2/Cyfip1/Tmbim6/Lap3/Alb/Cs/Atp1b1/Acaa2/Aldh1l1/Aldh6a1/Actn4 |
| GO:0044238 | primary metabolic process | 132 | 0.216 | 0.819 | 0.853 | Cltc/Hadha/Atp1a1/Tst/P4hb/Mtdh/Idh1/Erp29/Apoe/Dlat/Erp44/Acox1/Pgm1/Mthfd1/Atp5h/Jup/Rpl10/Apobr |
| GO:0048513 | animal organ development | 31 | -0.219 | -0.733 | 0.854 | Rps14/Itgb1/Eef2/Hsp90ab1/Prkcsh/Scd1/Gpd2/Ap2a2/Cfl1/Hspa9/Lrp1/Myh9/Dnm2/Actn1/Gnb1/Ppib/Clic4 |
| GO:0071704 | organic substance metabolic process | 141 | 0.215 | 0.817 | 0.854 | Ap2b1/Cltc/Hadha/Atp1a1/Tst/P4hb/Mtdh/Idh1/Erp29/Apoe/Dlat/Erp44/Acox1/Pgm1/Mthfd1/Atp5h/Jup/Rpl10/Apobr |
| GO:0043603 | cellular amide metabolic process | 37 | -0.220 | -0.760 | 0.856 | Pa2g4/Rplp2/Ass1/Eef1a1/Cyfip1/Cs/Acaa2 |
| GO:0015980 | energy derivation by oxidation of organic compounds | 27 | 0.239 | 0.718 | 0.858 | Idh1/Dlat/Ndufs7/Cox4i1 |
| GO:0046903 | secretion | 17 | 0.252 | 0.679 | 0.861 | Erp29/Apoe/Rab21 |
| GO:0030182 | neuron differentiation | 23 | -0.237 | -0.724 | 0.862 | Rpl4/Picalm/Afg3l2/Itgb1/Acsl4/Hsp90ab1/Prkcsh/Cfl1/Lrp1/Vapa/Dnm2/Eef1a1/Cyfip1 |
| GO:0048699 | generation of neurons | 23 | -0.237 | -0.724 | 0.862 | Rpl4/Picalm/Afg3l2/Itgb1/Acsl4/Hsp90ab1/Prkcsh/Cfl1/Lrp1/Vapa/Dnm2/Eef1a1/Cyfip1 |
| GO:0048856 | anatomical structure development | 65 | 0.218 | 0.767 | 0.864 | Ap2b1/Tst/P4hb/Mtdh/Apoe/Mthfd1/Rab21/Jup/Rpl10 |
| GO:0043170 | macromolecule metabolic process | 89 | -0.200 | -0.809 | 0.867 | Pacsin3/Rpl4/Picalm/Afg3l2/Pcyox1/Serpina3k/Rps14/Rplp0/Uqcrc2/Itgb1/Eef2/Tmed10/Rpl18/Erlin2/Acsl1/Hsp90ab1/Prkcsh/Park7/Prdx5/Ddost/Cfl1/Cdc42bpb/Hspa9/Bcap31/Lrp1/Gstp1/Myh9/Pgk1/Rab1b/Apoc1/Vcp/Ganab/Dnm2/Pa2g4/Cycs/Stoml2/Rplp2/Ppib/Prdx6/Cct8/Eef1a1/Cyfip1/Tmbim6/Lap3/Atp1b1/Actn4 |
| GO:1903829 | positive regulation of cellular protein localization | 12 | -0.276 | -0.711 | 0.868 | Itgb1/Hsp90ab1/Park7/Cfl1/Lrp1/Cct8 |
| GO:0044257 | cellular protein catabolic process | 14 | 0.260 | 0.672 | 0.873 | Apoe/Hsp90b1/Uggt1/Msn |
| GO:0043269 | regulation of ion transport | 30 | -0.214 | -0.718 | 0.873 | Itgb1/Tmed10/Acsl4/Acsl1/Hsp90ab1/Park7/Cfl1/Bcap31/Lrp1/Apoc1/Dnm2/Clic4/Maob/Tmbim6/Atp1b1 |
| GO:1903827 | regulation of cellular protein localization | 17 | 0.248 | 0.668 | 0.877 | Cltc |
| GO:0048585 | negative regulation of response to stimulus | 22 | -0.231 | -0.698 | 0.881 | Lrp1/Gstp1/Vcp/Dnm2/Tmbim6/Acaa2 |
| GO:0022008 | neurogenesis | 24 | -0.223 | -0.691 | 0.883 | Rpl4/Picalm/Afg3l2/Itgb1/Acsl4/Hsp90ab1/Prkcsh/Cfl1/Lrp1/Vapa/Dnm2/Eef1a1/Cyfip1 |
| GO:0034613 | cellular protein localization | 41 | 0.230 | 0.744 | 0.886 | Ap2b1/Cltc/Apoe/Rab21/Jup/Hsp90b1/Ndufa13/Msn/Calr |
| GO:0070727 | cellular macromolecule localization | 41 | 0.230 | 0.744 | 0.886 | Ap2b1/Cltc/Apoe/Rab21/Jup/Hsp90b1/Ndufa13/Msn/Calr |
| GO:0006807 | nitrogen compound metabolic process | 115 | -0.191 | -0.788 | 0.886 | Acadl/Pacsin3/Rpl4/Picalm/Afg3l2/Pcyox1/Serpina3k/Rps14/Rplp0/Uqcrc2/Got2/Itgb1/Ptdss1/Eef2/Suclg1/Tmed10/Acsl4/Rpl18/Erlin2/Acsl1/Hsp90ab1/Prkcsh/Park7/Prdx5/Ddost/Cfl1/Cdc42bpb/Hspa9/Bcap31/Lrp1/Gstp1/Myh9/Pgk1/Rab1b/Apoc1/Vcp/Ganab/Dnm2/Pa2g4/Cycs/Stoml2/Rplp2/Ass1/Ppib/Aldh9a1/Prdx6/Cct8/Maob/Eef1a1/Cyfip1/Tmbim6/Lap3/Cs/Acaa2/Aldh1l1/Aldh6a1/Actn4 |
| GO:0022900 | electron transport chain | 16 | -0.237 | -0.672 | 0.887 | Sdhb/Cox5a/Sdha/Uqcrfs1/Park7/Ndufb6/Slc25a12/Cycs/Uqcrc1 |
| GO:0060341 | regulation of cellular localization | 19 | 0.236 | 0.651 | 0.888 | Cltc/Rab21/Jup |
| GO:0048519 | negative regulation of biological process | 73 | -0.195 | -0.758 | 0.889 | Gstp1/Myh9/Pgk1/Apoc1/Vcp/Dnm2/Pa2g4/Actn1/Ass1/Clic4/Maob/Eef1a1/Cyfip1/Tmbim6/Alb/Acaa2/Actn4 |
| GO:1901566 | organonitrogen compound biosynthetic process | 47 | 0.222 | 0.732 | 0.890 | Cltc/Apoe/Dlat/Mthfd1/Atp5h/Rpl10/Uggt1/Rpl9/Calr/Acat1 |
| GO:0043254 | regulation of protein-containing complex assembly | 12 | -0.266 | -0.684 | 0.893 | Park7/Cfl1/Arpc4/Vcp/Cyfip1 |
| GO:0060560 | developmental growth involved in morphogenesis | 10 | -0.268 | -0.651 | 0.897 | Rpl4/Picalm/Itgb1/Hsp90ab1/Cfl1/Lrp1/Dnm2/Cyfip1 |
| GO:0009057 | macromolecule catabolic process | 20 | 0.234 | 0.655 | 0.897 | Apoe/Hsp90b1/Uggt1/Ndufa13/Msn |
| GO:1903793 | positive regulation of anion transport | 12 | 0.256 | 0.638 | 0.901 | Apoe/Jup/Cd81/Glud1/Tmem30a/Itgb1/Tmed10/Acsl4/Acsl1/Hsp90ab1/Bcap31/Lrp1 |
| GO:0032502 | developmental process | 71 | 0.207 | 0.740 | 0.904 | Ap2b1/Tst/P4hb/Mtdh/Apoe/Acox1/Mthfd1/Rab21/Jup/Rpl10 |
| GO:0007268 | chemical synaptic transmission | 10 | -0.265 | -0.642 | 0.906 | Atp2a2/Itgb1/Park7/Cfl1/Vdac3/Cyfip1 |
| GO:0098916 | anterograde trans-synaptic signaling | 10 | -0.265 | -0.642 | 0.906 | Atp2a2/Itgb1/Park7/Cfl1/Vdac3/Cyfip1 |
| GO:0099536 | synaptic signaling | 10 | -0.265 | -0.642 | 0.906 | Atp2a2/Itgb1/Park7/Cfl1/Vdac3/Cyfip1 |
| GO:0099537 | trans-synaptic signaling | 10 | -0.265 | -0.642 | 0.906 | Atp2a2/Itgb1/Park7/Cfl1/Vdac3/Cyfip1 |
| GO:0006163 | purine nucleotide metabolic process | 19 | -0.228 | -0.668 | 0.911 | Pgk1/Vcp/Stoml2/Cs/Acaa2 |
| GO:0051649 | establishment of localization in cell | 54 | 0.210 | 0.723 | 0.915 | Ap2b1/Cltc/Erp29/Hyou1/Atp5h/Rab21/Jup/Hsp90b1/Canx/Ndufa13/Msn/Calr |
| GO:0043009 | chordate embryonic development | 10 | -0.258 | -0.626 | 0.916 | Gnas/Gna13/Itgb1/Prkcsh/Cfl1/Myh9/Atp1b1 |
| GO:0044249 | cellular biosynthetic process | 71 | 0.204 | 0.731 | 0.916 | Atp1a1/Mtdh/Idh1/Apoe/Dlat/Acox1/Mthfd1/Atp5h/Jup/Rpl10 |
| GO:0006631 | fatty acid metabolic process | 16 | 0.238 | 0.637 | 0.917 | Hadha/Acox1 |
| GO:0008104 | protein localization | 50 | 0.207 | 0.698 | 0.918 | Ap2b1/Cltc/Erp29/Apoe/Rab21/Jup/Rrbp1/Hsp90b1/Ndufa13/Msn/Calr |
| GO:0010975 | regulation of neuron projection development | 15 | -0.236 | -0.658 | 0.919 | Ptprf/Itpr1/Tmem30a/Rpl4/Picalm/Itgb1/Prkcsh/Cfl1/Lrp1/Eef1a1/Cyfip1 |
| GO:0072521 | purine-containing compound metabolic process | 20 | -0.220 | -0.648 | 0.922 | Pgk1/Vcp/Stoml2/Cs/Acaa2 |
| GO:1903362 | regulation of cellular protein catabolic process | 10 | -0.255 | -0.619 | 0.922 | Hsp90ab1/Park7/Bcap31/Lrp1/Vcp |
| GO:0045862 | positive regulation of proteolysis | 14 | -0.235 | -0.634 | 0.923 | Cfl1/Bcap31/Myh9/Vcp/Cycs |
| GO:0006952 | defense response | 15 | -0.234 | -0.653 | 0.923 | Itgb1/Park7/Scd1/Gstp1/Vdac3/Rab14 |
| GO:0010562 | positive regulation of phosphorus metabolic process | 13 | -0.244 | -0.644 | 0.924 | Itgb1/Acsl1/Hsp90ab1/Park7/Vcp/Stoml2 |
| GO:0045937 | positive regulation of phosphate metabolic process | 13 | -0.244 | -0.644 | 0.924 | Itgb1/Acsl1/Hsp90ab1/Park7/Vcp/Stoml2 |
| GO:0050808 | synapse organization | 12 | -0.254 | -0.653 | 0.925 | Cfl1/Vcp/Actn1/Cyfip1 |
| GO:0006753 | nucleoside phosphate metabolic process | 20 | -0.217 | -0.641 | 0.930 | Suclg1/Acsl4/Acsl1/Park7/Pgk1/Vcp/Stoml2/Cs/Acaa2 |
| GO:0009117 | nucleotide metabolic process | 20 | -0.217 | -0.641 | 0.930 | Suclg1/Acsl4/Acsl1/Park7/Pgk1/Vcp/Stoml2/Cs/Acaa2 |
| GO:0007409 | axonogenesis | 11 | -0.243 | -0.609 | 0.932 | Rpl4/Picalm/Afg3l2/Itgb1/Hsp90ab1/Lrp1/Dnm2/Cyfip1 |
| GO:1901700 | response to oxygen-containing compound | 32 | -0.197 | -0.653 | 0.934 | Park7/Prdx5/Scd1/Cfl1/Pdia3/Lrp1/Gstp1/Sigmar1/Vcp/Dnm2/Prdx6/Cyfip1/Tmbim6 |
| GO:0035967 | cellular response to topologically incorrect protein | 10 | 0.252 | 0.601 | 0.934 | Canx/Uggt1/Pdia6/Calr |
| GO:0044265 | cellular macromolecule catabolic process | 16 | 0.232 | 0.620 | 0.935 | Apoe/Hsp90b1/Uggt1/Msn |
| GO:0009790 | embryo development | 14 | -0.230 | -0.620 | 0.936 | Tpi1/Gnas/Gna13/Itgb1/Acsl4/Prkcsh/Cfl1/Myh9/Rab14/Atp1b1 |
| GO:0044070 | regulation of anion transport | 18 | 0.219 | 0.595 | 0.938 | Erp29/Apoe/Jup |
| GO:0006119 | oxidative phosphorylation | 18 | -0.217 | -0.630 | 0.938 | Atp5c1/Cox5a/Sdha/Uqcrfs1/Park7/Ndufb6/Vcp/Cycs/Stoml2/Uqcrc1 |
| GO:0010468 | regulation of gene expression | 35 | 0.201 | 0.634 | 0.940 | Mtdh/Erp29/Apoe/Jup/Rpl10/Ndufa13/Msn/Calr |
| GO:0032501 | multicellular organismal process | 84 | 0.189 | 0.695 | 0.941 | Ap2b1/Cltc/Atp1a1/Mtdh/Apoe/Acox1/Mthfd1/Rab21/Jup/Rpl10 |
| GO:0031344 | regulation of cell projection organization | 19 | -0.213 | -0.625 | 0.944 | Rpl4/Picalm/Itgb1/Prkcsh/Cfl1/Lrp1/Dnm2/Vdac3/Eef1a1/Cyfip1 |
| GO:0120035 | regulation of plasma membrane bounded cell projection organization | 19 | -0.213 | -0.625 | 0.944 | Rpl4/Picalm/Itgb1/Prkcsh/Cfl1/Lrp1/Dnm2/Vdac3/Eef1a1/Cyfip1 |
| GO:0051248 | negative regulation of protein metabolic process | 12 | 0.236 | 0.587 | 0.946 | Apoe/Calr/Phb/Rpl5/Gnai2/Rps3/Picalm/Serpina3k/Tmed10/Hsp90ab1/Park7/Gstp1 |
| GO:0031346 | positive regulation of cell projection organization | 16 | -0.215 | -0.609 | 0.946 | Cfl1/Lrp1/Dnm2/Eef1a1/Cyfip1 |
| GO:0040008 | regulation of growth | 16 | -0.217 | -0.613 | 0.946 | Gnas/Rpl4/Picalm/Afg3l2/Acsl4/Cfl1/Lrp1/Dnm2/Ppib/Cyfip1 |
| GO:0033365 | protein localization to organelle | 22 | 0.211 | 0.600 | 0.950 | Ap2b1/Jup/Ndufa13/Msn/Calr |
| GO:0046034 | ATP metabolic process | 25 | -0.203 | -0.633 | 0.950 | Pgk1/Vcp/Cycs/Stoml2/Uqcrc1/Atp1b1 |
| GO:0032535 | regulation of cellular component size | 13 | -0.227 | -0.598 | 0.950 | Rpl4/Picalm/Hsp90ab1/Cfl1/Lrp1/Arpc4/Dnm2/Cyfip1 |
| GO:0090066 | regulation of anatomical structure size | 13 | -0.227 | -0.598 | 0.950 | Rpl4/Picalm/Hsp90ab1/Cfl1/Lrp1/Arpc4/Dnm2/Cyfip1 |
| GO:0010942 | positive regulation of cell death | 14 | -0.220 | -0.593 | 0.951 | Picalm/Itgb1/Park7/Pdia3/Bcap31/Lrp1/Eef1a1 |
| GO:0016049 | cell growth | 15 | -0.218 | -0.607 | 0.951 | Rpl4/Picalm/Itgb1/Acsl4/Hsp90ab1/Cfl1/Lrp1/Dnm2/Cyfip1 |
| GO:0032269 | negative regulation of cellular protein metabolic process | 11 | 0.235 | 0.573 | 0.951 | Apoe/Calr/Rpl5/Gnai2/Rps3/Picalm/Serpina3k/Tmed10/Hsp90ab1/Park7/Gstp1 |
| GO:0098609 | cell-cell adhesion | 13 | -0.225 | -0.595 | 0.952 | Gstp1/Myh9/Ass1 |
| GO:0010604 | positive regulation of macromolecule metabolic process | 36 | -0.181 | -0.628 | 0.955 | Itgb1/Eef2/Acsl1/Hsp90ab1/Park7/Cfl1/Bcap31/Lrp1/Myh9/Rab1b/Vcp/Cycs/Stoml2/Prdx6/Cct8 |
| GO:0051239 | regulation of multicellular organismal process | 39 | 0.190 | 0.610 | 0.956 | Atp1a1/Mtdh/Apoe/Rab21/Jup |
| GO:0032989 | cellular component morphogenesis | 14 | -0.217 | -0.586 | 0.957 | Rpl4/Picalm/Afg3l2/Itgb1/Hsp90ab1/Cfl1/Lrp1/Dnm2/Cyfip1 |
| GO:0032990 | cell part morphogenesis | 14 | -0.217 | -0.586 | 0.957 | Rpl4/Picalm/Afg3l2/Itgb1/Hsp90ab1/Cfl1/Lrp1/Dnm2/Cyfip1 |
| GO:0048667 | cell morphogenesis involved in neuron differentiation | 14 | -0.217 | -0.586 | 0.957 | Rpl4/Picalm/Afg3l2/Itgb1/Hsp90ab1/Cfl1/Lrp1/Dnm2/Cyfip1 |
| GO:0048812 | neuron projection morphogenesis | 14 | -0.217 | -0.586 | 0.957 | Rpl4/Picalm/Afg3l2/Itgb1/Hsp90ab1/Cfl1/Lrp1/Dnm2/Cyfip1 |
| GO:0048858 | cell projection morphogenesis | 14 | -0.217 | -0.586 | 0.957 | Rpl4/Picalm/Afg3l2/Itgb1/Hsp90ab1/Cfl1/Lrp1/Dnm2/Cyfip1 |
| GO:0120039 | plasma membrane bounded cell projection morphogenesis | 14 | -0.217 | -0.586 | 0.957 | Rpl4/Picalm/Afg3l2/Itgb1/Hsp90ab1/Cfl1/Lrp1/Dnm2/Cyfip1 |
| GO:1901575 | organic substance catabolic process | 46 | 0.190 | 0.626 | 0.958 | Hadha/Idh1/Apoe/Acox1/Hsp90b1/Uggt1/Ndufa13/Msn/Acat1/Echs1/Ahcy/Mccc2 |
| GO:0016310 | phosphorylation | 38 | -0.190 | -0.659 | 0.959 | Itgb1/Uqcrfs1/Acsl1/Hsp90ab1/Park7/Ndufb6/Cfl1/Cdc42bpb/Hspa9/Gstp1/Pgk1/Vcp/Cycs/Stoml2/Eef1a1/Uqcrc1 |
| GO:0042773 | ATP synthesis coupled electron transport | 13 | -0.220 | -0.581 | 0.961 | Cox5a/Sdha/Uqcrfs1/Park7/Ndufb6/Cycs/Uqcrc1 |
| GO:0048731 | system development | 55 | 0.184 | 0.634 | 0.962 | Ap2b1/Mtdh/Apoe/Mthfd1/Rab21/Jup/Rpl10 |
| GO:2000112 | regulation of cellular macromolecule biosynthetic process | 26 | 0.206 | 0.609 | 0.963 | Mtdh/Apoe/Jup/Rpl10/Ndufa13/Calr |
| GO:0034645 | cellular macromolecule biosynthetic process | 44 | -0.171 | -0.618 | 0.965 | Pa2g4/Stoml2/Rplp2/Cct8/Eef1a1/Cyfip1/Tmbim6 |
| GO:0009987 | cellular process | 193 | 0.195 | 0.631 | 0.965 | Ap2b1/Cltc/Hadha/Atp1a1/Tst/P4hb/Atp6v1a/Mtdh/Idh1/Abcb7/Erp29/Apoe/Dlat/Erp44/Ndufs7/Hyou1/Acox1/Cox4i1 |
| GO:0042775 | mitochondrial ATP synthesis coupled electron transport | 12 | -0.224 | -0.577 | 0.966 | Cox5a/Sdha/Uqcrfs1/Park7/Ndufb6/Cycs/Uqcrc1 |
| GO:0031401 | positive regulation of protein modification process | 11 | 0.228 | 0.556 | 0.966 | Erp29 |
| GO:0042327 | positive regulation of phosphorylation | 11 | 0.228 | 0.556 | 0.966 | Erp29 |
| GO:0045935 | positive regulation of nucleobase-containing compound metabolic process | 12 | -0.223 | -0.573 | 0.968 | Hsp90ab1/Park7/Vcp/Prdx6/Cct8 |
| GO:0061564 | axon development | 12 | -0.222 | -0.571 | 0.968 | Rpl4/Picalm/Afg3l2/Itgb1/Hsp90ab1/Lrp1/Dnm2/Cyfip1 |
| GO:0065009 | regulation of molecular function | 41 | -0.173 | -0.612 | 0.968 | Serpina3k/Gna13/Itgb1/Tmed10/Acsl1/Hsp90ab1/Park7/Prdx5/Cfl1/Bcap31/Lrp1/Gstp1/Apoc1/Vcp/Dnm2/Cycs/Eef1a1/Tmbim6/Atp1b1 |
| GO:0048523 | negative regulation of cellular process | 66 | 0.180 | 0.638 | 0.970 | Cltc/Atp1a1/Mtdh/Erp29/Apoe/Hyou1 |
| GO:0007610 | behavior | 12 | -0.220 | -0.566 | 0.970 | Picalm/Itgb1/Park7/Vdac3/Alb |
| GO:0010557 | positive regulation of macromolecule biosynthetic process | 13 | 0.210 | 0.536 | 0.972 | Apoe/Jup |
| GO:0010467 | gene expression | 53 | -0.161 | -0.592 | 0.973 | Lrp1/Gstp1/Myh9/Pgk1/Pa2g4/Stoml2/Rplp2/Prdx6/Eef1a1/Cyfip1/Tmbim6/Atp1b1 |
| GO:0034248 | regulation of cellular amide metabolic process | 11 | 0.219 | 0.534 | 0.976 | Apoe/Rpl10/Calr |
| GO:0042176 | regulation of protein catabolic process | 13 | 0.204 | 0.521 | 0.978 | Apoe/Ndufa13/Msn |
| GO:0051129 | negative regulation of cellular component organization | 17 | -0.185 | -0.531 | 0.980 | Vdac2/Ptprf/Hspa8/Sptbn1/Pacsin3/Picalm/Itgb1/Prkcsh/Cfl1/Lrp1/Dnm2/Acaa2 |
| GO:0007275 | multicellular organism development | 60 | -0.162 | -0.610 | 0.981 | Rpl4/Picalm/Afg3l2/Gna13/Rps14/Itgb1/Eef2/Acsl4/Hsp90ab1/Rab18/Prkcsh/Scd1/Gpd2/Ap2a2/Cfl1/Hspa9/Lrp1/Myh9/Sigmar1/Pgk1/Vapa/Dnm2/Rab14/Actn1/Gnb1/Ppib/Clic4/Eef1a1/Cyfip1/Atp1b1 |
| GO:0034622 | cellular protein-containing complex assembly | 28 | 0.180 | 0.547 | 0.981 | Cltc/Ndufs7/Rpl10/Ndufa13/Calr |
| GO:0001558 | regulation of cell growth | 13 | 0.198 | 0.506 | 0.981 | Apoe/Rab21/Ndufa13 |
| GO:0051247 | positive regulation of protein metabolic process | 30 | 0.175 | 0.539 | 0.983 | Erp29/Apoe/Ndufa13/Msn/Aifm1/Rdx/Phb/Phb2/Rpl5/Hspd1/Cd81/Rps3/Ncstn/Hspa8/Rps9/Gnas/Pacsin3/Picalm/Itgb1/Eef2/Acsl1/Hsp90ab1/Park7/Cfl1/Bcap31/Lrp1/Myh9/Rab1b/Vcp/Cycs |
| GO:0071495 | cellular response to endogenous stimulus | 23 | -0.184 | -0.563 | 0.984 | Acsl1/Hsp90ab1/Park7/Cfl1/Lrp1/Dnm2/Rab14/Eef1a1/Cyfip1 |
| GO:0002682 | regulation of immune system process | 13 | -0.197 | -0.519 | 0.985 | Park7/Hspa9/Stoml2/Tmbim6 |
| GO:0008283 | cell population proliferation | 22 | 0.193 | 0.549 | 0.986 | Apoe/Jup/Msn/Calr |
| GO:0033108 | mitochondrial respiratory chain complex assembly | 10 | 0.208 | 0.497 | 0.986 | Ndufs7/Ndufa13/Ndufb5/Aifm1 |
| GO:0051246 | regulation of protein metabolic process | 39 | -0.167 | -0.582 | 0.988 | Pacsin3/Picalm/Serpina3k/Itgb1/Eef2/Tmed10/Acsl1/Hsp90ab1/Park7/Cfl1/Bcap31/Lrp1/Gstp1/Myh9/Rab1b/Vcp/Pa2g4/Cycs/Cyfip1 |
| GO:0065003 | protein-containing complex assembly | 39 | -0.161 | -0.562 | 0.988 | Picalm/Afg3l2/Rps14/Rplp0/Uqcrfs1/Hsp90ab1/Park7/Ndufb6/Cfl1/Arpc4/Sigmar1/Vcp/Dnm2/Stoml2/Ndufa9/Samm50/Aldh9a1/Cct8/Cyfip1 |
| GO:0043085 | positive regulation of catalytic activity | 25 | -0.167 | -0.521 | 0.988 | Picalm/Gna13/Itgb1/Acsl1/Hsp90ab1/Park7/Bcap31/Vcp/Dnm2/Cycs/Atp1b1 |
| GO:0006955 | immune response | 14 | 0.189 | 0.488 | 0.989 | Apoe |
| GO:0044267 | cellular protein metabolic process | 67 | -0.156 | -0.600 | 0.989 | Pacsin3/Rpl4/Picalm/Afg3l2/Pcyox1/Serpina3k/Rps14/Rplp0/Itgb1/Eef2/Tmed10/Rpl18/Erlin2/Acsl1/Hsp90ab1/Park7/Ddost/Cfl1/Cdc42bpb/Hspa9/Bcap31/Lrp1/Gstp1/Myh9/Vcp/Pa2g4/Cycs/Stoml2/Rplp2/Ppib/Eef1a1/Cyfip1 |
| GO:0032270 | positive regulation of cellular protein metabolic process | 29 | 0.174 | 0.531 | 0.990 | Erp29/Apoe/Ndufa13/Msn/Aifm1/Rdx/Phb/Phb2/Rpl5/Hspd1/Cd81/Rps3/Ncstn/Hspa8/Rps9/Gnas/Pacsin3/Picalm/Itgb1/Eef2/Acsl1/Hsp90ab1/Park7/Cfl1/Bcap31/Lrp1/Myh9/Vcp/Cycs |
| GO:0043933 | protein-containing complex subunit organization | 40 | -0.155 | -0.547 | 0.993 | Hsp90ab1/Park7/Ndufb6/Cfl1/Arpc4/Sigmar1/Vcp/Dnm2/Stoml2/Ndufa9/Samm50/Aldh9a1/Cct8/Cyfip1 |
| GO:0007399 | nervous system development | 30 | -0.138 | -0.462 | 0.993 | Ptprf/Ncstn/Itpr1/Tmem30a/Sdha/Rpl4/Picalm/Afg3l2/Itgb1/Acsl4/Hsp90ab1/Prkcsh/Cfl1/Lrp1/Sigmar1/Vapa/Dnm2/Eef1a1/Cyfip1 |
| GO:0032268 | regulation of cellular protein metabolic process | 38 | -0.149 | -0.514 | 0.995 | Pacsin3/Picalm/Serpina3k/Itgb1/Eef2/Tmed10/Acsl1/Hsp90ab1/Park7/Cfl1/Bcap31/Lrp1/Gstp1/Myh9/Vcp/Pa2g4/Cycs/Cyfip1 |
| GO:0042254 | ribosome biogenesis | 10 | 0.171 | 0.408 | 0.996 | Rpl10/Rps8/Rpl6/Rpl5/Rps24/Rpl10a/Rpl7/Rps14/Rplp0 |
| GO:0001775 | cell activation | 15 | 0.160 | 0.421 | 0.996 | Apoe |
| GO:0006518 | peptide metabolic process | 28 | 0.156 | 0.475 | 0.997 | Idh1/Apoe |
| GO:1901564 | organonitrogen compound metabolic process | 106 | 0.158 | 0.594 | 0.997 | Cltc/Tst/P4hb/Idh1/Erp29/Apoe/Dlat/Erp44 |
| GO:0010628 | positive regulation of gene expression | 17 | 0.146 | 0.393 | 1.000 | Erp29/Msn/Calr |
| GO:0022613 | ribonucleoprotein complex biogenesis | 11 | 0.163 | 0.399 | 1.000 | Rpl10/Rps8/Rpl6/Rpl5/Rps24/Rpl10a/Rpl7/Rps14/Rplp0/Hsp90ab1/Pa2g4 |
| MF Terms | | | | | | |
| GO:0033218 | amide binding | 12 | 0.621 | 1.505 | 0.079 | Cltc/Hadha/Apoe |
| GO:0008324 | cation transmembrane transporter activity | 15 | 0.549 | 1.401 | 0.112 | Atp1a1/Atp6v1a/Cox4i1/Atp5h |
| GO:0022890 | inorganic cation transmembrane transporter activity | 14 | 0.555 | 1.381 | 0.130 | Atp1a1/Atp6v1a/Cox4i1/Atp5h |
| GO:0031625 | ubiquitin protein ligase binding | 15 | -0.489 | -1.318 | 0.135 | Erlin2/Hsp90ab1/Hspa9/Vcp/Pa2g4/Prdx6/Uqcrc1/Tmbim6 |
| GO:0044389 | ubiquitin-like protein ligase binding | 15 | -0.489 | -1.318 | 0.135 | Erlin2/Hsp90ab1/Hspa9/Vcp/Pa2g4/Prdx6/Uqcrc1/Tmbim6 |
| GO:0030554 | adenyl nucleotide binding | 30 | 0.430 | 1.289 | 0.162 | Hadha/Atp1a1/Atp6v1a/Abcb7/Hyou1/Mthfd1/Hsp90b1/Ndufa13/Acat1/Ahcy/Mccc2 |
| GO:0016853 | isomerase activity | 10 | 0.566 | 1.293 | 0.165 | P4hb/Erp44/Pgm1/Pdia6 |
| GO:0032559 | adenyl ribonucleotide binding | 29 | 0.428 | 1.281 | 0.172 | Hadha/Atp1a1/Atp6v1a/Abcb7/Hyou1/Mthfd1 |
| GO:0051287 | NAD binding | 10 | 0.557 | 1.273 | 0.177 | Hadha/Idh1 |
| GO:0005215 | transporter activity | 30 | 0.422 | 1.266 | 0.185 | Atp1a1/Atp6v1a/Abcb7/Apoe/Cox4i1/Atp5h |
| GO:0046983 | protein dimerization activity | 23 | 0.434 | 1.220 | 0.210 | Atp1a1/P4hb/Idh1/Erp29/Apoe/Acox1 |
| GO:0044877 | protein-containing complex binding | 54 | -0.301 | -1.135 | 0.215 | Uqcrc2/Itgb1/Uqcrfs1/Eef2/Suclg1/Tmed10/Prkcsh/Park7/Ap2a2/Cfl1/Cdc42bpb/Bcap31/Lrp1/Hadhb/Arpc4/Myh9/Ndufa4/Vcp/Vapa/Dnm2/Ndufa9/Actn1/Gnb1/Clic4/Rab32/Uqcrc1/Aldh1l1/Actn4 |
| GO:0001882 | nucleoside binding | 20 | -0.411 | -1.206 | 0.223 | Rab1b/Dnm2/Rab14/Rab32/Eef1a1/Actn4 |
| GO:0016887 | ATPase activity | 16 | 0.472 | 1.219 | 0.242 | Atp1a1/Atp6v1a/Abcb7 |
| GO:0005509 | calcium ion binding | 12 | -0.454 | -1.159 | 0.255 | Prkcsh/Gpd2/Lrp1/Slc25a12/Actn1/Actn4 |
| GO:0042802 | identical protein binding | 46 | -0.303 | -1.109 | 0.275 | Slc25a12/Myh9/Sigmar1/Vcp/Vapa/Actn1/Ass1/Aldh9a1/Prdx6/Decr1/Maob/Eef1a1/Alb/Actn4 |
| GO:0022857 | transmembrane transporter activity | 27 | 0.394 | 1.159 | 0.286 | Atp1a1/Atp6v1a/Abcb7/Cox4i1/Atp5h |
| GO:0030234 | enzyme regulator activity | 14 | -0.422 | -1.121 | 0.291 | Hsp90ab1/Park7/Prdx5/Ddost/Gstp1/Apoc1/Vcp/Tmbim6/Atp1b1 |
| GO:0003674 | molecular_function | 201 | -0.421 | -1.102 | 0.292 | Cdc42bpb/Hspa9/Pdia3/Bcap31/Lrp1/Hadhb/Arpc4/Slc25a12/Gstp1/Myh9/Sigmar1/Pgk1/Ndufa4/Rab1b/Apoc1/Vcp/Ganab/Vapa/Dnm2/Vdac3/Rab14/Mdh1/Pa2g4/Cycs/Stoml2/Ndufa9/Ndufb4/Actn1/Rplp2/Gnb1/Samm50/Ass1/Ppib/Aldh9a1/Clic4/Prdx6/Decr1/Rab32/Cct8/Slc25a1/Maob/Eef1a1/Bdh1/Uqcrc1/Aco2/Cyfip1/Tmbim6/Lap3/Alb/Cs/Atp1b1/Acaa2/Aldh1l1/Aldh6a1/Actn4 |
| GO:0005524 | ATP binding | 26 | 0.382 | 1.109 | 0.333 | Atp1a1/Atp6v1a/Abcb7/Hyou1/Mthfd1/Hsp90b1 |
| GO:0015318 | inorganic molecular entity transmembrane transporter activity | 23 | 0.395 | 1.109 | 0.334 | Atp1a1/Atp6v1a/Cox4i1/Atp5h |
| GO:0003924 | GTPase activity | 16 | -0.397 | -1.094 | 0.344 | Rab1b/Dnm2/Rab14/Gnb1/Rab32/Eef1a1 |
| GO:0015075 | ion transmembrane transporter activity | 23 | 0.384 | 1.079 | 0.361 | Atp1a1/Atp6v1a/Cox4i1/Atp5h |
| GO:0098772 | molecular function regulator | 22 | -0.354 | -1.065 | 0.372 | Hsp90ab1/Park7/Prdx5/Ddost/Gstp1/Apoc1/Vcp/Pa2g4/Actn1/Tmbim6/Atp1b1/Actn4 |
| GO:0019904 | protein domain specific binding | 15 | 0.423 | 1.079 | 0.379 | Cltc/Atp1a1/Acox1 |
| GO:0005515 | protein binding | 139 | -0.256 | -1.040 | 0.379 | Etfa/Abcd3/Ap2a2/Cfl1/Cdc42bpb/Hspa9/Pdia3/Bcap31/Lrp1/Arpc4/Slc25a12/Gstp1/Myh9/Sigmar1/Pgk1/Ndufa4/Vcp/Ganab/Vapa/Dnm2/Rab14/Pa2g4/Cycs/Stoml2/Ndufa9/Actn1/Gnb1/Samm50/Ass1/Ppib/Aldh9a1/Clic4/Prdx6/Decr1/Rab32/Cct8/Maob/Eef1a1/Uqcrc1/Cyfip1/Tmbim6/Alb/Atp1b1/Aldh1l1/Actn4 |
| GO:0019843 | rRNA binding | 12 | 0.428 | 1.037 | 0.404 | Tst |
| GO:0003723 | RNA binding | 30 | 0.342 | 1.026 | 0.425 | Cltc/Tst/Mtdh |
| GO:0017076 | purine nucleotide binding | 47 | 0.300 | 0.990 | 0.448 | Hadha/Atp1a1/Atp6v1a/Abcb7/Hyou1/Mthfd1/Rab21/Hsp90b1/Ndufa13/Acat1/Rala/Ahcy/Mccc2/Mfn1 |
| GO:0032553 | ribonucleotide binding | 47 | 0.300 | 0.991 | 0.448 | Hadha/Atp1a1/Atp6v1a/Abcb7/Hyou1/Mthfd1/Rab21/Hsp90b1/Ndufa13/Acat1/Rala/Ndufv1/Mccc2/Mfn1 |
| GO:0019899 | enzyme binding | 57 | -0.261 | -1.001 | 0.463 | Ap2a2/Cfl1/Cdc42bpb/Hspa9/Lrp1/Arpc4/Gstp1/Vcp/Dnm2/Pa2g4/Cycs/Stoml2/Gnb1/Ppib/Prdx6/Eef1a1/Uqcrc1/Cyfip1/Tmbim6/Alb/Atp1b1 |
| GO:0005488 | binding | 180 | 0.287 | 1.018 | 0.477 | Ap2b1/Cltc/Hadha/Atp1a1/Tst/P4hb/Atp6v1a/Mtdh/Idh1/Abcb7/Erp29/Apoe/Dlat/Erp44/Ndufs7/Hyou1/Acox1/Cox4i1/Pgm1/Mthfd1/Atp5h/Rab21/Jup |
| GO:0140096 | catalytic activity, acting on a protein | 14 | 0.389 | 0.968 | 0.490 | P4hb/Dlat/Erp44 |
| GO:0032555 | purine ribonucleotide binding | 46 | 0.296 | 0.976 | 0.492 | Hadha/Atp1a1/Atp6v1a/Abcb7/Hyou1/Mthfd1/Rab21 |
| GO:0016491 | oxidoreductase activity | 50 | 0.290 | 0.967 | 0.497 | Hadha/P4hb/Idh1/Dlat/Ndufs7/Acox1/Cox4i1/Mthfd1 |
| GO:0000166 | nucleotide binding | 63 | 0.276 | 0.968 | 0.507 | Hadha/Atp1a1/Atp6v1a/Idh1/Abcb7/Hyou1/Acox1/Mthfd1/Rab21 |
| GO:1901265 | nucleoside phosphate binding | 63 | 0.276 | 0.968 | 0.507 | Hadha/Atp1a1/Atp6v1a/Idh1/Abcb7/Hyou1/Acox1/Mthfd1/Rab21 |
| GO:0019901 | protein kinase binding | 17 | 0.366 | 0.965 | 0.520 | Cltc/Atp1a1 |
| GO:0046872 | metal ion binding | 45 | -0.265 | -0.966 | 0.538 | Uqcrc1/Aco2/Lap3/Alb/Actn4 |
| GO:0008092 | cytoskeletal protein binding | 28 | 0.319 | 0.949 | 0.538 | Cltc/Atp1a1/P4hb/Apoe |
| GO:0043169 | cation binding | 47 | -0.261 | -0.963 | 0.544 | Uqcrc1/Aco2/Lap3/Alb/Actn4 |
| GO:1901363 | heterocyclic compound binding | 95 | 0.257 | 0.961 | 0.556 | Cltc/Hadha/Atp1a1/Tst/Atp6v1a/Mtdh/Idh1/Abcb7 |
| GO:0003779 | actin binding | 15 | -0.347 | -0.934 | 0.557 | Cfl1/Arpc4/Myh9/Actn1/Cyfip1/Actn4 |
| GO:0042803 | protein homodimerization activity | 15 | 0.361 | 0.920 | 0.561 | Idh1/Erp29/Apoe/Acox1 |
| GO:0019900 | kinase binding | 18 | 0.352 | 0.939 | 0.562 | Cltc/Atp1a1 |
| GO:0097367 | carbohydrate derivative binding | 50 | 0.278 | 0.924 | 0.564 | Hadha/Atp1a1/Atp6v1a/Abcb7/Apoe/Hyou1/Mthfd1/Rab21 |
| GO:0097159 | organic cyclic compound binding | 99 | 0.253 | 0.954 | 0.580 | Cltc/Hadha/Atp1a1/Tst/Atp6v1a/Mtdh/Idh1/Abcb7 |
| GO:0003824 | catalytic activity | 116 | 0.252 | 0.952 | 0.583 | Hadha/Atp1a1/Tst/P4hb/Atp6v1a/Idh1/Abcb7/Dlat/Erp44/Ndufs7/Acox1/Cox4i1/Pgm1/Mthfd1/Atp5h/Rab21 |
| GO:0009055 | electron transfer activity | 17 | 0.332 | 0.874 | 0.637 | Ndufs7/Cox4i1/Ndufa13/Ndufv1/Aifm1/Cyc1/Etfdh |
| GO:0016787 | hydrolase activity | 48 | -0.246 | -0.904 | 0.658 | Hspa9/Pdia3/Myh9/Rab1b/Vcp/Ganab/Dnm2/Rab14/Gnb1/Prdx6/Rab32/Eef1a1/Lap3/Atp1b1/Acaa2/Aldh6a1 |
| GO:0001883 | purine nucleoside binding | 19 | -0.298 | -0.859 | 0.683 | Gna13/Eef2/Suclg1/Hsp90ab1/Rab18/Rab1b/Dnm2/Rab14/Rab32/Eef1a1 |
| GO:0019001 | guanyl nucleotide binding | 19 | -0.298 | -0.859 | 0.683 | Gna13/Eef2/Suclg1/Hsp90ab1/Rab18/Rab1b/Dnm2/Rab14/Rab32/Eef1a1 |
| GO:0032549 | ribonucleoside binding | 19 | -0.298 | -0.859 | 0.683 | Gna13/Eef2/Suclg1/Hsp90ab1/Rab18/Rab1b/Dnm2/Rab14/Rab32/Eef1a1 |
| GO:0032550 | purine ribonucleoside binding | 19 | -0.298 | -0.859 | 0.683 | Gna13/Eef2/Suclg1/Hsp90ab1/Rab18/Rab1b/Dnm2/Rab14/Rab32/Eef1a1 |
| GO:0032561 | guanyl ribonucleotide binding | 19 | -0.298 | -0.859 | 0.683 | Gna13/Eef2/Suclg1/Hsp90ab1/Rab18/Rab1b/Dnm2/Rab14/Rab32/Eef1a1 |
| GO:0016740 | transferase activity | 16 | 0.322 | 0.831 | 0.686 | Hadha/Tst/Dlat |
| GO:0005198 | structural molecule activity | 21 | 0.294 | 0.809 | 0.709 | Cltc/Jup/Rpl10/Rpl9 |
| GO:0005102 | signaling receptor binding | 32 | 0.269 | 0.822 | 0.720 | Cltc/P4hb/Apoe/Jup/Hsp90b1/Canx/Msn/Calr/Rala |
| GO:0005525 | GTP binding | 18 | -0.288 | -0.822 | 0.720 | Gna13/Eef2/Hsp90ab1/Rab18/Rab1b/Dnm2/Rab14/Rab32/Eef1a1 |
| GO:0035639 | purine ribonucleoside triphosphate binding | 42 | 0.255 | 0.814 | 0.773 | Atp1a1/Atp6v1a/Abcb7/Hyou1/Mthfd1/Rab21/Hsp90b1 |
| GO:0003676 | nucleic acid binding | 34 | 0.255 | 0.792 | 0.774 | Cltc/Tst/Mtdh |
| GO:0016651 | oxidoreductase activity, acting on NAD(P)H | 10 | 0.335 | 0.765 | 0.780 | Ndufs7/Ndufa13/Ndufv1/Aifm1 |
| GO:0016462 | pyrophosphatase activity | 32 | 0.256 | 0.781 | 0.788 | Atp1a1/Atp6v1a/Abcb7 |
| GO:0016817 | hydrolase activity, acting on acid anhydrides | 32 | 0.256 | 0.781 | 0.788 | Atp1a1/Atp6v1a/Abcb7 |
| GO:0016818 | hydrolase activity, acting on acid anhydrides, in phosphorus-containing anhydrides | 32 | 0.256 | 0.781 | 0.788 | Atp1a1/Atp6v1a/Abcb7 |
| GO:0017111 | nucleoside-triphosphatase activity | 32 | 0.256 | 0.781 | 0.788 | Atp1a1/Atp6v1a/Abcb7 |
| GO:0008289 | lipid binding | 29 | 0.245 | 0.732 | 0.828 | Atp1a1/Apoe/Acox1 |
| GO:0036094 | small molecule binding | 71 | 0.219 | 0.791 | 0.830 | Hadha/Atp1a1/Atp6v1a/Idh1/Abcb7/Hyou1/Acox1/Mthfd1/Rab21 |
| GO:0016874 | ligase activity | 10 | 0.296 | 0.676 | 0.891 | Mthfd1/Atp5h |
| GO:0005216 | ion channel activity | 10 | -0.272 | -0.654 | 0.914 | Vdac3/Clic4 |
| GO:0015267 | channel activity | 10 | -0.272 | -0.654 | 0.914 | Vdac3/Clic4 |
| GO:0022803 | passive transmembrane transporter activity | 10 | -0.272 | -0.654 | 0.914 | Vdac3/Clic4 |
| GO:0005543 | phospholipid binding | 16 | -0.226 | -0.624 | 0.916 | Vdac2/Myo1b/Hspa8/Itpr1/Sptbn1/Pacsin3/Picalm/Got2/Ap2a2/Cfl1/Stoml2/Bdh1 |
| GO:0051536 | iron-sulfur cluster binding | 10 | -0.261 | -0.627 | 0.932 | Sdhb/Cisd1/Uqcrfs1/Aco2 |
| GO:0051540 | metal cluster binding | 10 | -0.261 | -0.627 | 0.932 | Sdhb/Cisd1/Uqcrfs1/Aco2 |
| GO:0043168 | anion binding | 63 | 0.171 | 0.602 | 0.976 | Atp1a1/Atp6v1a/Abcb7/Hyou1/Acox1/Mthfd1/Rab21 |
| GO:0043167 | ion binding | 96 | -0.173 | -0.706 | 0.980 | Uqcrc2/Got2/Itgb1/Uqcrfs1/Eef2/Suclg1/Acsl4/Acsl1/Hsp90ab1/Rab18/Prkcsh/Park7/Scd1/Gpd2/Etfa/Abcd3/Cdc42bpb/Hspa9/Lrp1/Slc25a12/Gstp1/Myh9/Pgk1/Rab1b/Apoc1/Vcp/Dnm2/Rab14/Stoml2/Actn1/Ass1/Decr1/Rab32/Cct8/Maob/Eef1a1/Uqcrc1/Aco2/Lap3/Alb/Actn4 |
| GO:0003735 | structural constituent of ribosome | 16 | 0.199 | 0.514 | 0.987 | Rpl10/Rpl9/Rps8/Rpl6/Rpl8/Rpl5/Rps24/Rps3/Rpl10a/Rps9/Rpl7/Rpl4/Rps14/Rplp0/Rpl18 |
| CC Terms | | | | | | |
| GO:0015629 | actin cytoskeleton | 13 | -0.590 | -1.527 | 0.030 | Cfl1/Cdc42bpb/Arpc4/Myh9/Stoml2/Actn1/Eef1a1/Actn4 |
| GO:0031981 | nuclear lumen | 39 | -0.411 | -1.445 | 0.054 | Pa2g4/Ndufa9/Ndufb4/Ppib/Clic4/Decr1/Cct8/Eef1a1/Lap3/Aldh6a1/Actn4 |
| GO:0005938 | cell cortex | 10 | -0.597 | -1.437 | 0.061 | Cfl1/Myh9/Actn1/Eef1a1/Actn4 |
| GO:0005634 | nucleus | 72 | -0.326 | -1.262 | 0.090 | Park7/Prdx5/Scd1/Ndufb6/Cfl1/Hspa9/Pdia3/Lrp1/Arpc4/Gstp1/Myh9/Sigmar1/Vcp/Vapa/Dnm2/Pa2g4/Cycs/Stoml2/Ndufa9/Ndufb4/Actn1/Ass1/Ppib/Clic4/Prdx6/Decr1/Cct8/Eef1a1/Lap3/Aldh6a1/Actn4 |
| GO:0005654 | nucleoplasm | 25 | -0.433 | -1.363 | 0.096 | Ndufa9/Ndufb4/Ppib/Decr1/Cct8/Lap3/Aldh6a1/Actn4 |
| GO:0005790 | smooth endoplasmic reticulum | 10 | 0.606 | 1.415 | 0.099 | Erp29/Hyou1/Rpl10/Hsp90b1/Canx/Pdia6/Calr |
| GO:0016324 | apical plasma membrane | 13 | 0.566 | 1.400 | 0.110 | Atp1a1/Atp6v1a/Mtdh |
| GO:0098797 | plasma membrane protein complex | 17 | 0.519 | 1.376 | 0.111 | Ap2b1/Cltc/Atp1a1 |
| GO:0098590 | plasma membrane region | 40 | 0.400 | 1.280 | 0.135 | Ap2b1/Cltc/Atp1a1/Atp6v1a/Mtdh/Ndufs7 |
| GO:0048471 | perinuclear region of cytoplasm | 27 | -0.386 | -1.245 | 0.172 | Hsp90ab1/Park7/Prdx5/Bcap31/Lrp1/Vcp/Vapa/Dnm2/Rab14/Ppib/Clic4/Prdx6/Cyfip1/Actn4 |
| GO:0030135 | coated vesicle | 12 | 0.540 | 1.309 | 0.176 | Ap2b1/Cltc |
| GO:0005739 | mitochondrion | 105 | -0.284 | -1.170 | 0.178 | Dnm2/Vdac3/Mdh1/Cycs/Stoml2/Ndufa9/Ndufb4/Samm50/Ass1/Aldh9a1/Clic4/Prdx6/Decr1/Rab32/Slc25a1/Maob/Bdh1/Uqcrc1/Aco2/Tmbim6/Lap3/Cs/Acaa2/Aldh1l1/Aldh6a1 |
| GO:0042383 | sarcolemma | 10 | 0.562 | 1.313 | 0.181 | Cltc/Atp1a1 |
| GO:0009986 | cell surface | 21 | 0.443 | 1.240 | 0.192 | P4hb/Erp29/Apoe/Erp44 |
| GO:0045177 | apical part of cell | 16 | 0.487 | 1.270 | 0.194 | Atp1a1/Atp6v1a/Mtdh |
| GO:0098552 | side of membrane | 17 | 0.472 | 1.250 | 0.204 | P4hb/Apoe/Rab21/Jup/Canx/Msn/Calr |
| GO:0005741 | mitochondrial outer membrane | 16 | -0.452 | -1.234 | 0.214 | Acsl4/Acsl1/Hadhb/Vdac3/Samm50/Ass1/Rab32/Maob |
| GO:0019867 | outer membrane | 16 | -0.452 | -1.234 | 0.214 | Acsl4/Acsl1/Hadhb/Vdac3/Samm50/Ass1/Rab32/Maob |
| GO:0031968 | organelle outer membrane | 16 | -0.452 | -1.234 | 0.214 | Acsl4/Acsl1/Hadhb/Vdac3/Samm50/Ass1/Rab32/Maob |
| GO:0005768 | endosome | 20 | 0.447 | 1.224 | 0.215 | Cltc/Atp1a1/Apoe/Rab21 |
| GO:0030659 | cytoplasmic vesicle membrane | 15 | 0.479 | 1.231 | 0.225 | Ap2b1/Cltc |
| GO:0030139 | endocytic vesicle | 11 | 0.524 | 1.253 | 0.229 | Ap2b1/Cltc |
| GO:0031410 | cytoplasmic vesicle | 41 | 0.360 | 1.159 | 0.263 | Ap2b1/Cltc/Atp1a1/Erp29/Apoe |
| GO:0097708 | intracellular vesicle | 41 | 0.360 | 1.159 | 0.263 | Ap2b1/Cltc/Atp1a1/Erp29/Apoe |
| GO:0005856 | cytoskeleton | 28 | -0.354 | -1.153 | 0.264 | Cfl1/Cdc42bpb/Lrp1/Arpc4/Myh9/Vapa/Dnm2/Mdh1/Stoml2/Actn1/Clic4/Cct8/Eef1a1/Actn4 |
| GO:0005886 | plasma membrane | 76 | 0.314 | 1.127 | 0.267 | Ap2b1/Cltc/Atp1a1/P4hb/Atp6v1a/Mtdh/Apoe/Ndufs7/Rab21/Jup/Apobr/Hsp90b1/Canx |
| GO:0098791 | Golgi apparatus subcompartment | 11 | -0.472 | -1.172 | 0.290 | Bcap31/Dnm2/Rab14/Rab32/Lap3 |
| GO:0012506 | vesicle membrane | 17 | 0.431 | 1.142 | 0.327 | Ap2b1/Cltc |
| GO:0012505 | endomembrane system | 86 | 0.293 | 1.068 | 0.342 | Ap2b1/Cltc/Atp1a1/P4hb/Mtdh/Erp29/Apoe/Erp44/Hyou1/Rab21/Rpl10/Rrbp1/Hsp90b1/Canx/Uggt1/Pdia6/Nomo1/Msn/Calr |
| GO:0030133 | transport vesicle | 13 | 0.459 | 1.133 | 0.344 | Cltc/Erp29 |
| GO:0005783 | endoplasmic reticulum | 63 | 0.309 | 1.071 | 0.351 | Atp1a1/P4hb/Mtdh/Erp29/Apoe/Erp44/Hyou1/Rab21/Rpl10/Rrbp1/Hsp90b1/Canx/Uggt1/Pdia6/Nomo1 |
| GO:0031974 | membrane-enclosed lumen | 74 | -0.269 | -1.049 | 0.357 | Pa2g4/Cycs/Stoml2/Ndufa9/Ndufb4/Ppib/Clic4/Decr1/Cct8/Eef1a1/Lap3/Cs/Acaa2/Aldh6a1/Actn4 |
| GO:0043233 | organelle lumen | 74 | -0.269 | -1.049 | 0.357 | Pa2g4/Cycs/Stoml2/Ndufa9/Ndufb4/Ppib/Clic4/Decr1/Cct8/Eef1a1/Lap3/Cs/Acaa2/Aldh6a1/Actn4 |
| GO:0070013 | intracellular organelle lumen | 74 | -0.269 | -1.049 | 0.357 | Pa2g4/Cycs/Stoml2/Ndufa9/Ndufb4/Ppib/Clic4/Decr1/Cct8/Eef1a1/Lap3/Cs/Acaa2/Aldh6a1/Actn4 |
| GO:0019898 | extrinsic component of membrane | 12 | 0.454 | 1.100 | 0.361 | Cltc/Apoe/Jup |
| GO:0031982 | vesicle | 46 | 0.320 | 1.052 | 0.392 | Ap2b1/Cltc/Atp1a1/Erp29/Apoe |
| GO:0000323 | lytic vacuole | 10 | 0.427 | 0.996 | 0.449 | Cltc/Apoe |
| GO:0005764 | lysosome | 10 | 0.427 | 0.996 | 0.449 | Cltc/Apoe |
| GO:0005773 | vacuole | 10 | 0.427 | 0.996 | 0.449 | Cltc/Apoe |
| GO:0008021 | synaptic vesicle | 11 | 0.419 | 1.001 | 0.455 | Cltc |
| GO:0070382 | exocytic vesicle | 11 | 0.419 | 1.001 | 0.455 | Cltc |
| GO:0097060 | synaptic membrane | 14 | 0.386 | 0.975 | 0.472 | Cltc/Ndufs7/Canx |
| GO:0098978 | glutamatergic synapse | 13 | 0.388 | 0.958 | 0.478 | Ap2b1/Apoe |
| GO:1990904 | ribonucleoprotein complex | 22 | -0.317 | -0.979 | 0.479 | Pa2g4/Rplp2/Actn4 |
| GO:0030141 | secretory granule | 14 | -0.372 | -0.977 | 0.494 | Itgb1/Tmed10/Ap2a2/Pdia3/Lrp1/Rab14/Actn1 |
| GO:0098796 | membrane protein complex | 59 | 0.286 | 0.983 | 0.507 | Ap2b1/Cltc/Atp1a1/Atp6v1a/Ndufs7/Cox4i1/Atp5h/Jup |
| GO:0031253 | cell projection membrane | 10 | -0.403 | -0.968 | 0.525 | Gna13/Itgb1/Hsp90ab1/Dnm2/Gnb1/Eef1a1 |
| GO:0005829 | cytosol | 71 | -0.252 | -0.974 | 0.529 | Gstp1/Myh9/Pgk1/Vcp/Dnm2/Rab14/Mdh1/Cycs/Rplp2/Ass1/Aldh9a1/Clic4/Prdx6/Decr1/Cct8/Eef1a1/Aco2/Lap3/Aldh1l1/Actn4 |
| GO:0030424 | axon | 14 | -0.356 | -0.936 | 0.551 | Hsp90ab1/Park7/Cfl1/Lrp1/Dnm2/Cyfip1 |
| GO:0140534 | endoplasmic reticulum protein-containing complex | 13 | 0.368 | 0.909 | 0.560 | P4hb/Hyou1/Hsp90b1/Pdia6/Calr |
| GO:0045202 | synapse | 53 | 0.281 | 0.944 | 0.562 | Ap2b1/Cltc/Atp1a1/Apoe/Ndufs7/Rab21/Rpl10/Canx/Rpl9 |
| GO:0071944 | cell periphery | 80 | 0.265 | 0.961 | 0.575 | Ap2b1/Cltc/Atp1a1/P4hb/Atp6v1a/Mtdh/Apoe/Ndufs7/Rab21/Jup/Apobr/Hsp90b1/Canx |
| GO:0005575 | cellular_component | 203 | -0.419 | -0.963 | 0.581 | Cdc42bpb/Hspa9/Pdia3/Bcap31/Lrp1/Hadhb/Arpc4/Slc25a12/Gstp1/Myh9/Sigmar1/Pgk1/Ndufa4/Rab1b/Apoc1/Vcp/Ganab/Vapa/Dnm2/Vdac3/Rab14/Mdh1/Pa2g4/Cycs/Stoml2/Ndufa9/Ndufb4/Actn1/Rplp2/Gnb1/Samm50/Ass1/Ppib/Aldh9a1/Clic4/Prdx6/Decr1/Rab32/Cct8/Slc25a1/Maob/Eef1a1/Bdh1/Uqcrc1/Aco2/Cyfip1/Tmbim6/Lap3/Alb/Cs/Atp1b1/Acaa2/Aldh1l1/Aldh6a1/Actn4 |
| GO:0110165 | cellular anatomical entity | 203 | -0.419 | -0.963 | 0.581 | Cdc42bpb/Hspa9/Pdia3/Bcap31/Lrp1/Hadhb/Arpc4/Slc25a12/Gstp1/Myh9/Sigmar1/Pgk1/Ndufa4/Rab1b/Apoc1/Vcp/Ganab/Vapa/Dnm2/Vdac3/Rab14/Mdh1/Pa2g4/Cycs/Stoml2/Ndufa9/Ndufb4/Actn1/Rplp2/Gnb1/Samm50/Ass1/Ppib/Aldh9a1/Clic4/Prdx6/Decr1/Rab32/Cct8/Slc25a1/Maob/Eef1a1/Bdh1/Uqcrc1/Aco2/Cyfip1/Tmbim6/Lap3/Alb/Cs/Atp1b1/Acaa2/Aldh1l1/Aldh6a1/Actn4 |
| GO:0043005 | neuron projection | 33 | -0.268 | -0.911 | 0.616 | Dnm2/Actn1/Gnb1/Ass1/Cyfip1/Actn4 |
| GO:0015630 | microtubule cytoskeleton | 12 | 0.357 | 0.866 | 0.630 | Cltc/Apoe |
| GO:0120025 | plasma membrane bounded cell projection | 48 | -0.253 | -0.903 | 0.649 | Actn1/Gnb1/Ass1/Clic4/Cct8/Eef1a1/Cyfip1/Atp1b1/Actn4 |
| GO:0014069 | postsynaptic density | 19 | 0.322 | 0.871 | 0.651 | Atp1a1 |
| GO:0032279 | asymmetric synapse | 19 | 0.322 | 0.871 | 0.651 | Atp1a1 |
| GO:0098984 | neuron to neuron synapse | 19 | 0.322 | 0.871 | 0.651 | Atp1a1 |
| GO:0099572 | postsynaptic specialization | 19 | 0.322 | 0.871 | 0.651 | Atp1a1 |
| GO:0005911 | cell-cell junction | 14 | -0.325 | -0.854 | 0.665 | Myh9/Vapa/Actn1/Clic4/Atp1b1/Actn4 |
| GO:0031252 | cell leading edge | 12 | -0.330 | -0.842 | 0.669 | Cfl1/Myh9/Dnm2/Actn1/Eef1a1/Cyfip1 |
| GO:0005794 | Golgi apparatus | 25 | 0.294 | 0.860 | 0.686 | Cltc/Atp1a1/Apoe |
| GO:0005887 | integral component of plasma membrane | 11 | 0.337 | 0.806 | 0.700 | Atp1a1 |
| GO:0099081 | supramolecular polymer | 15 | 0.309 | 0.794 | 0.706 | Cltc/Apoe/Pgm1/Jup |
| GO:0099512 | supramolecular fiber | 15 | 0.309 | 0.794 | 0.706 | Cltc/Apoe/Pgm1/Jup |
| GO:0043227 | membrane-bounded organelle | 186 | -0.257 | -0.883 | 0.710 | Vcp/Ganab/Vapa/Dnm2/Vdac3/Rab14/Mdh1/Pa2g4/Cycs/Stoml2/Ndufa9/Ndufb4/Actn1/Gnb1/Samm50/Ass1/Ppib/Aldh9a1/Clic4/Prdx6/Decr1/Rab32/Cct8/Slc25a1/Maob/Eef1a1/Bdh1/Uqcrc1/Aco2/Tmbim6/Lap3/Alb/Cs/Atp1b1/Acaa2/Aldh1l1/Aldh6a1/Actn4 |
| GO:0005789 | endoplasmic reticulum membrane | 23 | 0.295 | 0.844 | 0.710 | Mtdh/Erp44/Rrbp1/Hsp90b1/Canx/Nomo1/Calr |
| GO:0031226 | intrinsic component of plasma membrane | 12 | 0.331 | 0.802 | 0.715 | Atp1a1 |
| GO:0098798 | mitochondrial protein-containing complex | 38 | 0.261 | 0.833 | 0.726 | Hadha/Dlat/Ndufs7/Cox4i1/Atp5h |
| GO:0030425 | dendrite | 17 | -0.284 | -0.794 | 0.726 | Rplp0/Itgb1/Hsp90ab1/Cfl1/Lrp1/Dnm2/Actn1/Gnb1/Cyfip1 |
| GO:0097447 | dendritic tree | 17 | -0.284 | -0.794 | 0.726 | Rplp0/Itgb1/Hsp90ab1/Cfl1/Lrp1/Dnm2/Actn1/Gnb1/Cyfip1 |
| GO:0099080 | supramolecular complex | 17 | -0.284 | -0.795 | 0.726 | Myh9/Vcp/Dnm2/Actn1/Cct8/Actn4 |
| GO:0070161 | anchoring junction | 16 | -0.294 | -0.801 | 0.737 | Myh9/Vapa/Actn1/Clic4/Atp1b1/Actn4 |
| GO:1990204 | oxidoreductase complex | 24 | 0.282 | 0.814 | 0.746 | P4hb/Dlat/Ndufs7 |
| GO:0098793 | presynapse | 16 | 0.292 | 0.761 | 0.755 | Cltc |
| GO:0031090 | organelle membrane | 112 | 0.231 | 0.867 | 0.758 | Ap2b1/Cltc/Hadha/Atp1a1/Tst/Mtdh/Abcb7/Erp44/Ndufs7/Acox1/Cox4i1/Atp5h/Rab21/Rrbp1/Hsp90b1/Canx/Nomo1/Ndufa13/Msn/Calr/Acat1/Rala/Ndufv1 |
| GO:0043231 | intracellular membrane-bounded organelle | 176 | -0.227 | -0.848 | 0.758 | Hspa9/Pdia3/Bcap31/Lrp1/Hadhb/Arpc4/Slc25a12/Gstp1/Myh9/Sigmar1/Ndufa4/Rab1b/Vcp/Ganab/Vapa/Dnm2/Vdac3/Rab14/Mdh1/Pa2g4/Cycs/Stoml2/Ndufa9/Ndufb4/Actn1/Samm50/Ass1/Ppib/Aldh9a1/Clic4/Prdx6/Decr1/Rab32/Cct8/Slc25a1/Maob/Eef1a1/Bdh1/Uqcrc1/Aco2/Tmbim6/Lap3/Alb/Cs/Acaa2/Aldh1l1/Aldh6a1/Actn4 |
| GO:0030054 | cell junction | 64 | 0.240 | 0.833 | 0.759 | Ap2b1/Cltc/Atp1a1/Mtdh/Apoe/Ndufs7/Rab21/Jup/Rpl10 |
| GO:0098827 | endoplasmic reticulum subcompartment | 24 | 0.278 | 0.802 | 0.763 | Mtdh/Erp44/Rrbp1/Hsp90b1/Canx/Nomo1/Calr |
| GO:0016020 | membrane | 148 | 0.229 | 0.868 | 0.784 | Ap2b1/Cltc/Hadha/Atp1a1/Tst/P4hb/Atp6v1a/Mtdh/Abcb7/Apoe/Erp44/Ndufs7/Acox1/Cox4i1/Atp5h/Rab21/Jup/Apobr/Rrbp1/Hsp90b1/Canx |
| GO:0042175 | nuclear outer membrane-endoplasmic reticulum membrane network | 24 | 0.271 | 0.781 | 0.796 | Mtdh/Erp44/Rrbp1/Hsp90b1/Canx/Nomo1/Calr |
| GO:0005576 | extracellular region | 19 | 0.277 | 0.748 | 0.801 | Apoe/Hyou1/Apobr/Hsp90b1/Pdia6/Calr |
| GO:0042995 | cell projection | 52 | -0.227 | -0.823 | 0.802 | Actn1/Gnb1/Ass1/Clic4/Cct8/Eef1a1/Cyfip1/Atp1b1/Actn4 |
| GO:0005740 | mitochondrial envelope | 71 | -0.216 | -0.835 | 0.806 | Immt/Ndufb6/Gpd2/Abcd3/Cfl1/Pdia3/Hadhb/Slc25a12/Ndufa4/Dnm2/Vdac3/Cycs/Stoml2/Ndufa9/Ndufb4/Samm50/Ass1/Rab32/Slc25a1/Maob/Bdh1/Uqcrc1/Tmbim6/Acaa2 |
| GO:0031966 | mitochondrial membrane | 68 | -0.211 | -0.809 | 0.832 | Dnm2/Vdac3/Stoml2/Ndufa9/Ndufb4/Samm50/Ass1/Rab32/Slc25a1/Maob/Bdh1/Uqcrc1/Tmbim6/Acaa2 |
| GO:0045121 | membrane raft | 15 | 0.271 | 0.697 | 0.833 | Atp1a1 |
| GO:0098857 | membrane microdomain | 15 | 0.271 | 0.697 | 0.833 | Atp1a1 |
| GO:0031967 | organelle envelope | 76 | -0.211 | -0.830 | 0.836 | Immt/Ndufb6/Gpd2/Abcd3/Cfl1/Pdia3/Hadhb/Slc25a12/Sigmar1/Ndufa4/Vapa/Dnm2/Vdac3/Cycs/Stoml2/Ndufa9/Ndufb4/Samm50/Ass1/Rab32/Slc25a1/Maob/Bdh1/Uqcrc1/Tmbim6/Acaa2 |
| GO:0031975 | envelope | 76 | -0.211 | -0.830 | 0.836 | Immt/Ndufb6/Gpd2/Abcd3/Cfl1/Pdia3/Hadhb/Slc25a12/Sigmar1/Ndufa4/Vapa/Dnm2/Vdac3/Cycs/Stoml2/Ndufa9/Ndufb4/Samm50/Ass1/Rab32/Slc25a1/Maob/Bdh1/Uqcrc1/Tmbim6/Acaa2 |
| GO:0043226 | organelle | 195 | -0.264 | -0.807 | 0.836 | Vcp/Ganab/Vapa/Dnm2/Vdac3/Rab14/Mdh1/Pa2g4/Cycs/Stoml2/Ndufa9/Ndufb4/Actn1/Rplp2/Gnb1/Samm50/Ass1/Ppib/Aldh9a1/Clic4/Prdx6/Decr1/Rab32/Cct8/Slc25a1/Maob/Eef1a1/Bdh1/Uqcrc1/Aco2/Tmbim6/Lap3/Alb/Cs/Atp1b1/Acaa2/Aldh1l1/Aldh6a1/Actn4 |
| GO:1902494 | catalytic complex | 41 | 0.235 | 0.756 | 0.836 | Atp1a1/P4hb/Dlat/Ndufs7 |
| GO:0044297 | cell body | 22 | -0.232 | -0.717 | 0.850 | Got2/Acsl4/Hsp90ab1/Park7/Cfl1/Lrp1/Gnb1/Ass1/Cct8/Cyfip1 |
| GO:0043209 | myelin sheath | 44 | 0.231 | 0.751 | 0.860 | Cltc/Atp1a1/Atp6v1a/Dlat |
| GO:0098858 | actin-based cell projection | 10 | 0.288 | 0.674 | 0.874 | Atp6v1a |
| GO:0036477 | somatodendritic compartment | 26 | -0.224 | -0.712 | 0.883 | Rplp0/Got2/Itgb1/Acsl4/Hsp90ab1/Cfl1/Lrp1/Dnm2/Actn1/Gnb1/Ass1/Cyfip1 |
| GO:0099503 | secretory vesicle | 22 | 0.241 | 0.681 | 0.886 | Cltc |
| GO:0032991 | protein-containing complex | 125 | 0.205 | 0.780 | 0.888 | Ap2b1/Cltc/Hadha/Atp1a1/P4hb/Atp6v1a/Apoe/Dlat/Ndufs7/Hyou1/Cox4i1/Atp5h/Jup/Rpl10/Apobr/Hsp90b1/Canx/Uggt1/Rpl9/Pdia6/Nomo1/Ndufa13 |
| GO:0031301 | integral component of organelle membrane | 13 | 0.268 | 0.662 | 0.888 | Rrbp1/Canx/Calr/Mfn1/Etfdh/Slc25a3/Sfxn1/Ero1a/Itpr1/Afg3l2/Immt/Scd1/Pdia3 |
| GO:0005622 | intracellular anatomical structure | 197 | -0.243 | -0.715 | 0.892 | Vcp/Ganab/Vapa/Dnm2/Vdac3/Rab14/Mdh1/Pa2g4/Cycs/Stoml2/Ndufa9/Ndufb4/Actn1/Rplp2/Gnb1/Samm50/Ass1/Ppib/Aldh9a1/Clic4/Prdx6/Decr1/Rab32/Cct8/Slc25a1/Maob/Eef1a1/Bdh1/Uqcrc1/Aco2/Cyfip1/Tmbim6/Lap3/Alb/Cs/Acaa2/Aldh1l1/Aldh6a1/Actn4 |
| GO:0005737 | cytoplasm | 197 | -0.243 | -0.715 | 0.892 | Vcp/Ganab/Vapa/Dnm2/Vdac3/Rab14/Mdh1/Pa2g4/Cycs/Stoml2/Ndufa9/Ndufb4/Actn1/Rplp2/Gnb1/Samm50/Ass1/Ppib/Aldh9a1/Clic4/Prdx6/Decr1/Rab32/Cct8/Slc25a1/Maob/Eef1a1/Bdh1/Uqcrc1/Aco2/Cyfip1/Tmbim6/Lap3/Alb/Cs/Acaa2/Aldh1l1/Aldh6a1/Actn4 |
| GO:0005769 | early endosome | 11 | 0.270 | 0.644 | 0.902 | Apoe/Rab21 |
| GO:0098588 | bounding membrane of organelle | 42 | 0.212 | 0.684 | 0.908 | Ap2b1/Cltc |
| GO:0005759 | mitochondrial matrix | 23 | 0.230 | 0.656 | 0.910 | Hadha/Dlat |
| GO:0005743 | mitochondrial inner membrane | 58 | 0.205 | 0.702 | 0.921 | Hadha/Tst/Abcb7/Ndufs7/Cox4i1/Atp5h |
| GO:0005730 | nucleolus | 14 | 0.249 | 0.631 | 0.923 | Mtdh |
| GO:0005840 | ribosome | 18 | 0.234 | 0.620 | 0.924 | Rpl10/Canx/Rpl9/Rps8/Rpl6/Rpl8/Rpl5/Rps24/Rps3/Rpl10a/Rps9/Rpl7/Rpl4/Rps14/Rplp0/Eef2/Rpl18 |
| GO:0019866 | organelle inner membrane | 59 | 0.202 | 0.695 | 0.925 | Hadha/Tst/Abcb7/Ndufs7/Cox4i1/Atp5h |
| GO:0019897 | extrinsic component of plasma membrane | 11 | 0.251 | 0.600 | 0.935 | Apoe/Jup |
| GO:0043228 | non-membrane-bounded organelle | 63 | 0.205 | 0.709 | 0.937 | Cltc/Hadha/P4hb/Mtdh/Apoe |
| GO:0043232 | intracellular non-membrane-bounded organelle | 63 | 0.205 | 0.709 | 0.937 | Cltc/Hadha/P4hb/Mtdh/Apoe |
| GO:0098562 | cytoplasmic side of membrane | 10 | 0.251 | 0.587 | 0.946 | Rab21/Jup/Msn |
| GO:0005747 | mitochondrial respiratory chain complex I | 13 | 0.223 | 0.551 | 0.953 | Ndufs7/Ndufa13/Ndufv1/Ndufb5 |
| GO:0030964 | NADH dehydrogenase complex | 13 | 0.223 | 0.551 | 0.953 | Ndufs7/Ndufa13/Ndufv1/Ndufb5 |
| GO:0045271 | respiratory chain complex I | 13 | 0.223 | 0.551 | 0.953 | Ndufs7/Ndufa13/Ndufv1/Ndufb5 |
| GO:0031984 | organelle subcompartment | 33 | 0.193 | 0.602 | 0.957 | Mtdh/Erp44/Rab21/Rrbp1/Hsp90b1/Canx/Nomo1/Calr |
| GO:0043229 | intracellular organelle | 187 | 0.199 | 0.678 | 0.962 | Cltc/Hadha/Atp1a1/Tst/P4hb/Atp6v1a/Mtdh/Idh1/Abcb7/Erp29/Apoe/Dlat/Erp44/Ndufs7/Hyou1/Acox1/Cox4i1/Pgm1/Mthfd1/Atp5h/Rab21/Jup/Rpl10 |
| GO:0022626 | cytosolic ribosome | 16 | 0.199 | 0.519 | 0.968 | Rpl10/Rpl9/Rps8/Rpl6/Rpl8/Rpl5/Rps24/Rps3/Rpl10a/Rps9/Rpl7/Rpl4/Rps14/Rplp0/Rpl18 |
| GO:0044391 | ribosomal subunit | 16 | 0.199 | 0.519 | 0.968 | Rpl10/Rpl9/Rps8/Rpl6/Rpl8/Rpl5/Rps24/Rps3/Rpl10a/Rps9/Rpl7/Rpl4/Rps14/Rplp0/Rpl18 |
| GO:0098794 | postsynapse | 24 | 0.202 | 0.583 | 0.970 | Atp1a1 |
| GO:0005615 | extracellular space | 14 | 0.213 | 0.540 | 0.974 | Apoe/Apobr/Pdia6/Calr |
| GO:0031300 | intrinsic component of organelle membrane | 16 | 0.187 | 0.489 | 0.976 | Rrbp1/Canx/Calr/Mfn1/Rab5b |
| GO:0043025 | neuronal cell body | 18 | 0.192 | 0.510 | 0.976 | Apoe/Ndufs7/Canx |
| GO:0016021 | integral component of membrane | 62 | -0.166 | -0.627 | 0.985 | Itgb1/Uqcrfs1/Ptdss1/Tmed10/Acsl4/Erlin2/Acsl1/Ddost/Immt/Scd1/Ndufb6/Abcd3/Pdia3/Bcap31/Lrp1/Slc25a12/Sigmar1/Ndufa4/Vapa/Vdac3/Samm50/Clic4/Slc25a1/Maob/Tmbim6/Atp1b1 |
| GO:0005746 | mitochondrial respirasome | 21 | -0.162 | -0.493 | 0.989 | Ndufa4/Ndufa9/Ndufb4/Uqcrc1 |
| GO:0070469 | respirasome | 21 | -0.162 | -0.493 | 0.989 | Ndufa4/Ndufa9/Ndufb4/Uqcrc1 |
| GO:0098803 | respiratory chain complex | 21 | -0.162 | -0.493 | 0.989 | Ndufa4/Ndufa9/Ndufb4/Uqcrc1 |
| GO:0098800 | inner mitochondrial membrane protein complex | 28 | 0.157 | 0.472 | 0.993 | Ndufs7/Cox4i1/Atp5h |
| GO:0031224 | intrinsic component of membrane | 67 | -0.155 | -0.590 | 0.997 | Itgb1/Uqcrfs1/Ptdss1/Tmed10/Acsl4/Erlin2/Acsl1/Ddost/Immt/Scd1/Ndufb6/Abcd3/Pdia3/Bcap31/Lrp1/Slc25a12/Sigmar1/Ndufa4/Vapa/Vdac3/Rab14/Samm50/Clic4/Slc25a1/Maob/Tmbim6/Atp1b1 |
| GO:0015934 | large ribosomal subunit | 11 | 0.153 | 0.367 | 1.000 | Rpl10/Rpl9 |
| GO:0022625 | cytosolic large ribosomal subunit | 11 | 0.153 | 0.367 | 1.000 | Rpl10/Rpl9 |
| KEGG Terms | | | | | | |
| mmu04810 | Regulation of actin cytoskeleton | 10 | -0.577 | -1.382 | 0.107 | Cfl1/Arpc4/Myh9/Actn1/Cyfip1/Actn4 |
| mmu04141 | Protein processing in endoplasmic reticulum | 20 | 0.473 | 1.323 | 0.127 | P4hb/Erp29/Hyou1/Rrbp1/Hsp90b1/Canx/Uggt1/Pdia6/Calr |
| mmu05016 | Huntington disease | 33 | 0.345 | 1.068 | 0.373 | Ap2b1/Cltc/Ndufs7/Cox4i1/Atp5h |
| mmu04022 | cGMP-PKG signaling pathway | 10 | 0.398 | 0.939 | 0.547 | Atp1a1 |
| mmu00280 | Valine, leucine and isoleucine degradation | 10 | -0.393 | -0.940 | 0.564 | Aldh9a1/Acaa2/Aldh6a1 |
| mmu01212 | Fatty acid metabolism | 11 | 0.346 | 0.828 | 0.669 | Hadha/Acox1 |
| mmu01200 | Carbon metabolism | 18 | -0.292 | -0.837 | 0.690 | Aco2/Cs/Aldh6a1 |
| mmu01100 | Metabolic pathways | 72 | 0.249 | 0.876 | 0.704 | Hadha/Tst/Atp6v1a/Idh1/Dlat/Ndufs7/Acox1/Cox4i1/Pgm1/Mthfd1/Atp5h |
| mmu00071 | Fatty acid degradation | 11 | 0.330 | 0.791 | 0.707 | Hadha/Acox1 |
| mmu00190 | Oxidative phosphorylation | 26 | 0.277 | 0.815 | 0.726 | Atp6v1a/Ndufs7/Cox4i1/Atp5h |
| mmu04723 | Retrograde endocannabinoid signaling | 17 | -0.268 | -0.749 | 0.816 | Ndufa4/Ndufa9/Ndufb4/Gnb1 |
| mmu05014 | Amyotrophic lateral sclerosis | 28 | -0.219 | -0.714 | 0.866 | Sigmar1/Ndufa4/Vcp/Cycs/Ndufa9/Ndufb4/Uqcrc1 |
| mmu05022 | Pathways of neurodegeneration - multiple diseases | 35 | -0.198 | -0.672 | 0.933 | Sigmar1/Ndufa4/Vcp/Vdac3/Cycs/Ndufa9/Ndufb4/Uqcrc1 |
| mmu05012 | Parkinson disease | 35 | -0.197 | -0.666 | 0.944 | Ndufa4/Vdac3/Cycs/Ndufa9/Ndufb4/Maob/Uqcrc1 |
| mmu04932 | Non-alcoholic fatty liver disease | 22 | -0.213 | -0.637 | 0.950 | Ndufa4/Cycs/Ndufa9/Ndufb4/Uqcrc1 |
| mmu05010 | Alzheimer disease | 34 | 0.184 | 0.569 | 0.973 | Apoe/Ndufs7/Cox4i1/Atp5h |
| mmu05200 | Pathways in cancer | 11 | -0.211 | -0.520 | 0.976 | Gnas/Gna13/Itgb1/Hsp90ab1/Gstp1/Cycs/Gnb1 |
| mmu05020 | Prion disease | 31 | -0.176 | -0.576 | 0.977 | Ndufa4/Vdac3/Cycs/Ndufa9/Ndufb4/Uqcrc1 |
| mmu03010 | Ribosome | 16 | 0.199 | 0.529 | 0.980 | Rpl10/Rpl9/Rps8/Rpl6/Rpl8/Rpl5/Rps24/Rps3/Rpl10a/Rps9/Rpl7/Rpl4/Rps14/Rplp0/Rpl18 |
| mmu05171 | Coronavirus disease - COVID-19 | 16 | 0.199 | 0.529 | 0.980 | Rpl10/Rpl9/Rps8/Rpl6/Rpl8/Rpl5/Rps24/Rps3/Rpl10a/Rps9/Rpl7/Rpl4/Rps14/Rplp0/Rpl18 |
| mmu05208 | Chemical carcinogenesis - reactive oxygen species | 27 | 0.161 | 0.474 | 0.994 | Ndufs7/Cox4i1/Atp5h |
| mmu04714 | Thermogenesis | 27 | 0.156 | 0.459 | 0.996 | Ndufs7/Cox4i1/Atp5h |
| mmu05415 | Diabetic cardiomyopathy | 28 | 0.154 | 0.455 | 0.998 | Ndufs7/Cox4i1/Atp5h |
