# Supplementary material for: The ultrastructural and proteomic analysis of mitochondria‐associated endoplasmic reticulum membrane in the midbrain of a Parkinson's disease mouse model
Source: Aging Cell. 2024 Nov 29;24(4):e14436. doi: 10.1111/acel.14436 (PMC11984660; doi:10.1111/acel.14436)
Supplement: Supplementary file 21 — Table S15. PPI network information of KEGG pathways for consensus MAM proteins in MAM proteomics. [file ACEL-24-e14436-s016.docx]

### Supplementary Table 15 PPI network information of KEGG pathways for consensus MAM proteins in MAM proteomics

| Name in PPI plot | Term name | Term ID | Adjusted p value | negative log10 of adjusted p value | Term size | Query size | Intersection size | Effective domain size | Intersections |
| --- | --- | --- | --- | --- | --- | --- | --- | --- | --- |
| OXPHOS | Oxidative phosphorylation | KEGG:00190 | 0.000 | 15.813 | 133 | 167 | 25 | 8875 | NDUFB6,COX5A,NDUFA9,UQCRC2,UQCRFS1,NDUFB4,ATP5H,UQCRC1,COX4I1,NDUFB10,NDUFV2,ATP6V1A,SDHB,CYC1,NDUFS3,NDUFA4,ATP5O,ATP5C1,NDUFB5,NDUFV1,NDUFS1,NDUFS7,NDUFA13,SDHA,NDUFS2 |
| TCA cycle | Citrate cycle (TCA cycle) | KEGG:00020 | 0.000 | 7.714 | 32 | 167 | 10 | 8875 | MDH1,MDH2,DLAT,SDHB,IDH1,FH,CS,SUCLG1,SDHA,ACO2 |
| Glycolysis / Gluconeogenesis | Glycolysis / Gluconeogenesis | KEGG:00010 | 0.003 | 2.518 | 66 | 167 | 8 | 8875 | ALDH9A1,ALDH2,PGK1,TPI1,LDHA,DLAT,PGM1,ENO1 |
| Protein processing | Protein processing in endoplasmic reticulum | KEGG:04141 | 0.000 | 9.202 | 171 | 167 | 21 | 8875 | MOGS,CALR,PDIA3,HSPA8,PDIA6,PRKCSH,ERO1B,HSP90B1,P4HB,UGGT1,ERO1A,CANX,GANAB,HYOU1,HSP90AB1,RRBP1,RPN1,ERP29,BCAP31,VCP,DDOST |
| Calcium reabsorption | Endocrine and other factor-regulated calcium reabsorption | KEGG:04961 | 0.002 | 2.773 | 61 | 167 | 8 | 8875 | DNM2,CLTC,GNAS,AP2B1,AP2A2,AP2A1,ATP1A1,ATP1B1 |
| Fatty acid metabolism | Fatty acid metabolism | KEGG:01212 | 0.000 | 5.775 | 62 | 167 | 11 | 8875 | ACOX1,ACSL4,SCD1,HADHB,ACSL1,ACAA2,ACAT1,HADHA,HSD17B4,ECHS1,ACADL |
| Fatty acid metabolism | Propanoate metabolism | KEGG:00640 | 0.002 | 2.692 | 31 | 167 | 6 | 8875 | ACOX1,LDHA,HADHA,SUCLG1,ECHS1,ALDH6A1 |
| Fatty acid metabolism | Fatty acid degradation | KEGG:00071 | 0.000 | 6.629 | 52 | 167 | 11 | 8875 | ACOX1,ALDH9A1,ALDH2,ACSL4,HADHB,ACSL1,ACAA2,ACAT1,HADHA,ECHS1,ACADL |
| Cholesterol metabolism | Cholesterol metabolism | KEGG:04979 | 0.004 | 2.426 | 50 | 167 | 7 | 8875 | APOC1,LRP1,VDAC3,APOE,VDAC2,VAPA,VDAC1 |
| Amino acid metabolism | Biosynthesis of amino acids | KEGG:01230 | 0.010 | 1.990 | 78 | 167 | 8 | 8875 | PGK1,ASS1,TPI1,IDH1,ENO1,CS,GOT2,ACO2 |
| Amino acid metabolism | Valine, leucine and isoleucine degradation | KEGG:00280 | 0.000 | 5.141 | 56 | 167 | 10 | 8875 | MCCC1,ALDH9A1,ALDH2,HADHB,ACAA2,ACAT1,HADHA,ECHS1,ALDH6A1,MCCC2 |
| Amino acid metabolism | beta-Alanine metabolism | KEGG:00410 | 0.002 | 2.608 | 32 | 167 | 6 | 8875 | ACOX1,ALDH9A1,ALDH2,HADHA,ECHS1,ALDH6A1 |
| Amino acid metabolism | Pyruvate metabolism | KEGG:00620 | 0.000 | 3.876 | 44 | 167 | 8 | 8875 | MDH1,ALDH9A1,ALDH2,LDHA,MDH2,DLAT,ACAT1,FH |
| Amino acid metabolism | Cysteine and methionine metabolism | KEGG:00270 | 0.045 | 1.346 | 53 | 167 | 6 | 8875 | MDH1,TST,LDHA,MDH2,AHCY,GOT2 |
| Amino acid metabolism | Tryptophan metabolism | KEGG:00380 | 0.036 | 1.438 | 51 | 167 | 6 | 8875 | MAOB,ALDH9A1,ALDH2,ACAT1,HADHA,ECHS1 |
| ROS | Chemical carcinogenesis - reactive oxygen species | KEGG:05208 | 0.000 | 12.347 | 220 | 167 | 27 | 8875 | NDUFB6,COX5A,NDUFA9,UQCRC2,UQCRFS1,NDUFB4,ATP5H,UQCRC1,COX4I1,VDAC3,NDUFB10,NDUFV2,VDAC2,SDHB,CYC1,NDUFS3,NDUFA4,ATP5O,ATP5C1,NDUFB5,VDAC1,NDUFV1,NDUFS1,NDUFS7,NDUFA13,SDHA,NDUFS2 |
| Peroxisome | Peroxisome | KEGG:04146 | 0.020 | 1.691 | 86 | 167 | 8 | 8875 | ACOX1,ACSL4,PRDX5,PRDX1,ACSL1,IDH1,HSD17B4,ABCD3 |
| Ribosome | Ribosome | KEGG:03010 | 0.000 | 5.796 | 169 | 167 | 17 | 8875 | RPLP0,RPL6,RPL10A,RPL18,RPL9,RPS3A,RPS14,RPL8,RPS3,RPS8,RPL7,RPL4,RPL10,RPL5,RPLP2,RPS9,RPS24 |
