# Supplementary material for: The ultrastructural and proteomic analysis of mitochondria‐associated endoplasmic reticulum membrane in the midbrain of a Parkinson's disease mouse model
Source: Aging Cell. 2024 Nov 29;24(4):e14436. doi: 10.1111/acel.14436 (PMC11984660; doi:10.1111/acel.14436)
Supplement: Supplementary file 22 — Table S16. K‐means functionally clustered information of BP terms for consensus MAM proteins in MAM proteomics. [file ACEL-24-e14436-s001.docx]

### Supplementary Table 16 K-means functionally clustered information of BP terms for consensus MAM proteins in MAM proteomics

| Cluster Name | Term description | Observed gene count | Background gene count | Strength | False discovery rate | Matching proteins in network |
| --- | --- | --- | --- | --- | --- | --- |
| Cytoskeleton regulation | Regulation of localization | 16 | 2806 | 0.760 | 0.000 | Rdx,Rala,Rab21,Gna13,Actn1,Rab14,Cyfip1,Cd81,Lrp1,Gnai2,Actn4,Gnas,Itgb1,Apoc1,Anxa6,Msn |
| Cytoskeleton regulation | Regulation of vesicle-mediated transport | 10 | 600 | 1.220 | 0.000 | Rdx,Rala,Rab21,Cyfip1,Lrp1,Gnai2,Actn4,Itgb1,Apoc1,Msn |
| Cytoskeleton regulation | Regulation of cell morphogenesis | 8 | 528 | 1.180 | 0.000 | Rdx,Myh9,Rab21,Gna13,Cyfip1,Lrp1,Actn4,Msn |
| Cytoskeleton regulation | Regulation of transport | 12 | 1862 | 0.810 | 0.000 | Rdx,Rala,Rab21,Cyfip1,Cd81,Lrp1,Gnai2,Actn4,Gnas,Itgb1,Apoc1,Msn |
| Cytoskeleton regulation | Positive regulation of early endosome to late endosome transport | 3 | 8 | 2.570 | 0.000 | Rdx,Rab21,Msn |
| Cytoskeleton regulation | Regulation of biological quality | 15 | 3822 | 0.590 | 0.000 | Rdx,Myh9,Rab21,Gna13,Rab14,Gnb1,Cyfip1,Cd81,Lrp1,Gnai2,Gnas,Itgb1,Anxa6,Msn,Arpc4 |
| Cytoskeleton regulation | Regulation of cell shape | 5 | 151 | 1.520 | 0.001 | Rdx,Myh9,Gna13,Cyfip1,Msn |
| Cytoskeleton regulation | Anatomical structure morphogenesis | 12 | 2244 | 0.730 | 0.001 | Rala,Myh9,Gna13,Actn1,Alcam,Cyfip1,Cd81,Lrp1,Gnas,Itgb1,Anxa6,Msn |
| Cytoskeleton regulation | Cellular component organization | 16 | 4769 | 0.530 | 0.001 | Rdx,Rala,Myh9,Rab21,Actn1,Alcam,Rab14,Cyfip1,Lrp1,Sigmar1,Actn4,Itgb1,Apoc1,Anxa6,Msn,Arpc4 |
| Cytoskeleton regulation | Regulation of anatomical structure morphogenesis | 9 | 1090 | 0.920 | 0.001 | Rdx,Myh9,Rab21,Gna13,Cyfip1,Lrp1,Actn4,Itgb1,Msn |
| Cytoskeleton regulation | Positive regulation of transport | 9 | 1020 | 0.950 | 0.001 | Rdx,Rab21,Cd81,Lrp1,Gnai2,Actn4,Gnas,Itgb1,Msn |
| Cytoskeleton regulation | Regulation of cellular component movement | 9 | 1048 | 0.930 | 0.001 | Rdx,Gna13,Actn1,Cd81,Lrp1,Gnai2,Actn4,Itgb1,Msn |
| Cytoskeleton regulation | Regulation of cellular component organization | 12 | 2438 | 0.690 | 0.001 | Rdx,Rala,Myh9,Rab21,Cyfip1,Cd81,Lrp1,Actn4,Itgb1,Apoc1,Msn,Arpc4 |
| Cytoskeleton regulation | System development | 15 | 4350 | 0.540 | 0.001 | Rala,Myh9,Rab21,Gna13,Actn1,Alcam,Gnb1,Cyfip1,Cd81,Lrp1,Sigmar1,Gnas,Itgb1,Anxa6,Msn |
| Cytoskeleton regulation | Ras protein signal transduction | 5 | 198 | 1.400 | 0.001 | Rala,Rab21,Gna13,Rab14,Cyfip1 |
| Cytoskeleton regulation | Response to stimulus | 18 | 6908 | 0.420 | 0.001 | Rdx,Rala,Myh9,Rab21,Gna13,Alcam,Rab14,Gnb1,Cyfip1,Cd81,Lrp1,Sigmar1,Gnai2,Actn4,Gnas,Itgb1,Anxa6,Msn |
| Cytoskeleton regulation | Regulation of cell migration | 8 | 915 | 0.940 | 0.001 | Rdx,Gna13,Cd81,Lrp1,Gnai2,Actn4,Itgb1,Msn |
| Cytoskeleton regulation | Anatomical structure development | 16 | 5258 | 0.480 | 0.001 | Rdx,Rala,Myh9,Rab21,Gna13,Actn1,Alcam,Gnb1,Cyfip1,Cd81,Lrp1,Sigmar1,Gnas,Itgb1,Anxa6,Msn |
| Cytoskeleton regulation | Regulation of cell size | 5 | 212 | 1.370 | 0.001 | Rdx,Rab21,Cyfip1,Lrp1,Msn |
| Cytoskeleton regulation | Regulation of cellular component size | 6 | 402 | 1.170 | 0.001 | Rdx,Rab21,Cyfip1,Lrp1,Msn,Arpc4 |
| Cytoskeleton regulation | Regulation of endocytosis | 5 | 228 | 1.340 | 0.002 | Rab21,Lrp1,Actn4,Itgb1,Apoc1 |
| Cytoskeleton regulation | Multicellular organismal process | 17 | 6272 | 0.430 | 0.002 | Rala,Myh9,Rab21,Gna13,Actn1,Alcam,Gnb1,Cyfip1,Cd81,Lrp1,Sigmar1,Gnai2,Gnas,Itgb1,Apoc1,Anxa6,Msn |
| Cytoskeleton regulation | Movement of cell or subcellular component | 9 | 1382 | 0.810 | 0.002 | Myh9,Rab21,Alcam,Cyfip1,Lrp1,Actn4,Itgb1,Anxa6,Msn |
| Cytoskeleton regulation | Cellular response to stimulus | 16 | 5497 | 0.460 | 0.002 | Rdx,Rala,Rab21,Gna13,Rab14,Gnb1,Cyfip1,Cd81,Lrp1,Sigmar1,Gnai2,Actn4,Gnas,Itgb1,Anxa6,Msn |
| Cytoskeleton regulation | Regulation of cellular process | 20 | 9541 | 0.320 | 0.002 | Rdx,Rala,Myh9,Rab21,Gna13,Actn1,Rab14,Gnb1,Cyfip1,Cd81,Lrp1,Sigmar1,Gnai2,Actn4,Gnas,Itgb1,Apoc1,Anxa6,Msn,Arpc4 |
| Cytoskeleton regulation | Developmental growth | 6 | 458 | 1.120 | 0.002 | Alcam,Cyfip1,Cd81,Gnas,Itgb1,Anxa6 |
| Cytoskeleton regulation | Establishment of localization | 13 | 3522 | 0.570 | 0.002 | Rdx,Rala,Myh9,Rab21,Rab14,Pcyox1,Cd81,Lrp1,Sigmar1,Actn4,Itgb1,Apoc1,Anxa6 |
| Cytoskeleton regulation | Regulation of cellular localization | 8 | 1083 | 0.870 | 0.002 | Rdx,Rab21,Cyfip1,Cd81,Lrp1,Itgb1,Anxa6,Msn |
| Cytoskeleton regulation | Actin cytoskeleton organization | 6 | 496 | 1.080 | 0.002 | Rala,Myh9,Actn1,Actn4,Itgb1,Arpc4 |
| Cytoskeleton regulation | Positive regulation of endocytosis | 4 | 125 | 1.510 | 0.002 | Rab21,Lrp1,Actn4,Itgb1 |
| Cytoskeleton regulation | Axon extension | 3 | 35 | 1.930 | 0.002 | Alcam,Cyfip1,Itgb1 |
| Cytoskeleton regulation | Response to chemical | 13 | 3720 | 0.540 | 0.003 | Rdx,Alcam,Rab14,Gnb1,Cyfip1,Cd81,Lrp1,Sigmar1,Gnai2,Actn4,Gnas,Itgb1,Msn |
| Cytoskeleton regulation | Positive regulation of protein catabolic process in the vacuole | 2 | 3 | 2.820 | 0.003 | Cd81,Lrp1 |
| Cytoskeleton regulation | Signal transduction | 13 | 3779 | 0.540 | 0.003 | Rala,Rab21,Gna13,Rab14,Gnb1,Cyfip1,Lrp1,Sigmar1,Gnai2,Actn4,Gnas,Itgb1,Anxa6 |
| Cytoskeleton regulation | Phagocytosis | 4 | 144 | 1.440 | 0.004 | Myh9,Rab14,Lrp1,Itgb1 |
| Cytoskeleton regulation | Positive regulation of cellular protein localization | 5 | 322 | 1.190 | 0.004 | Rdx,Cd81,Lrp1,Itgb1,Msn |
| Cytoskeleton regulation | Cellular process | 22 | 13330 | 0.220 | 0.004 | Rdx,Rala,Myh9,Rab21,Gna13,Actn1,Alcam,Rab14,Gnb1,Pcyox1,Cyfip1,Cd81,Lrp1,Sigmar1,Gnai2,Actn4,Gnas,Itgb1,Apoc1,Anxa6,Msn,Arpc4 |
| Cytoskeleton regulation | Regulation of developmental process | 11 | 2669 | 0.620 | 0.004 | Rdx,Myh9,Rab21,Gna13,Rab14,Cyfip1,Lrp1,Actn4,Gnas,Itgb1,Msn |
| Cytoskeleton regulation | Positive regulation of cellular component organization | 8 | 1236 | 0.810 | 0.004 | Rala,Rab21,Cyfip1,Lrp1,Actn4,Itgb1,Msn,Arpc4 |
| Cytoskeleton regulation | Cell morphogenesis involved in differentiation | 6 | 575 | 1.020 | 0.004 | Myh9,Actn1,Alcam,Cyfip1,Lrp1,Itgb1 |
| Cytoskeleton regulation | Positive regulation of cellular protein catabolic process | 4 | 157 | 1.410 | 0.004 | Rdx,Cd81,Lrp1,Msn |
| Cytoskeleton regulation | Localization | 14 | 4646 | 0.480 | 0.005 | Rdx,Rala,Myh9,Rab21,Rab14,Pcyox1,Cd81,Lrp1,Sigmar1,Actn4,Itgb1,Apoc1,Anxa6,Msn |
| Cytoskeleton regulation | Cell activation | 6 | 599 | 1.000 | 0.005 | Myh9,Gna13,Cd81,Lrp1,Gnas,Msn |
| Cytoskeleton regulation | Cell development | 9 | 1730 | 0.720 | 0.005 | Rdx,Myh9,Actn1,Alcam,Cyfip1,Lrp1,Itgb1,Anxa6,Msn |
| Cytoskeleton regulation | Transport | 12 | 3382 | 0.550 | 0.005 | Rala,Myh9,Rab21,Rab14,Pcyox1,Cd81,Lrp1,Sigmar1,Actn4,Itgb1,Apoc1,Anxa6 |
| Cytoskeleton regulation | Anatomical structure formation involved in morphogenesis | 7 | 952 | 0.870 | 0.006 | Rala,Myh9,Gna13,Actn1,Cd81,Gnas,Itgb1 |
| Cytoskeleton regulation | Regulation of actin filament-based process | 5 | 388 | 1.110 | 0.007 | Rdx,Myh9,Cyfip1,Lrp1,Arpc4 |
| Cytoskeleton regulation | Positive regulation of intracellular transport | 4 | 194 | 1.320 | 0.008 | Rdx,Rab21,Cd81,Msn |
| Cytoskeleton regulation | Cell-cell adhesion | 5 | 398 | 1.100 | 0.008 | Myh9,Alcam,Gnas,Itgb1,Msn |
| Cytoskeleton regulation | Cell differentiation | 12 | 3674 | 0.510 | 0.011 | Rdx,Myh9,Rab21,Gna13,Actn1,Alcam,Cyfip1,Cd81,Lrp1,Itgb1,Anxa6,Msn |
| Cytoskeleton regulation | Positive regulation of protein localization to early endosome | 2 | 10 | 2.300 | 0.012 | Rdx,Msn |
| Cytoskeleton regulation | Cell adhesion | 6 | 744 | 0.910 | 0.012 | Myh9,Actn1,Alcam,Gnas,Itgb1,Msn |
| Cytoskeleton regulation | Regulation of lipase activity | 3 | 78 | 1.590 | 0.012 | Gna13,Lrp1,Apoc1 |
| Cytoskeleton regulation | Cellular response to chemical stimulus | 10 | 2535 | 0.600 | 0.012 | Rdx,Rab14,Gnb1,Cyfip1,Lrp1,Sigmar1,Gnai2,Actn4,Gnas,Msn |
| Cytoskeleton regulation | Plasma membrane repair | 2 | 11 | 2.260 | 0.013 | Myh9,Anxa6 |
| Cytoskeleton regulation | Positive regulation of cell projection organization | 5 | 474 | 1.020 | 0.015 | Rala,Rab21,Cyfip1,Lrp1,Itgb1 |
| Cytoskeleton regulation | Calcium ion homeostasis | 5 | 477 | 1.020 | 0.015 | Gna13,Gnb1,Lrp1,Gnas,Anxa6 |
| Cytoskeleton regulation | Regulation of plasma membrane bounded cell projection organization | 6 | 788 | 0.880 | 0.015 | Rdx,Rala,Rab21,Cyfip1,Lrp1,Itgb1 |
| Cytoskeleton regulation | Positive regulation of cellular protein metabolic process | 8 | 1600 | 0.700 | 0.016 | Rdx,Myh9,Cd81,Lrp1,Gnai2,Gnas,Itgb1,Msn |
| Cytoskeleton regulation | Apical protein localization | 2 | 13 | 2.190 | 0.016 | Rdx,Rab14 |
| Cytoskeleton regulation | Locomotion | 7 | 1183 | 0.770 | 0.017 | Myh9,Alcam,Cyfip1,Lrp1,Itgb1,Anxa6,Msn |
| Cytoskeleton regulation | Regulation of hydrolase activity | 7 | 1184 | 0.770 | 0.017 | Rdx,Gna13,Lrp1,Gnai2,Gnas,Itgb1,Apoc1 |
| Cytoskeleton regulation | Positive regulation of cellular process | 14 | 5407 | 0.410 | 0.017 | Rdx,Rala,Myh9,Rab21,Actn1,Cyfip1,Cd81,Lrp1,Gnai2,Actn4,Gnas,Itgb1,Msn,Arpc4 |
| Cytoskeleton regulation | G protein-coupled receptor signaling pathway | 6 | 824 | 0.860 | 0.018 | Gna13,Gnb1,Lrp1,Sigmar1,Gnai2,Gnas |
| Cytoskeleton regulation | Immunological synapse formation | 2 | 15 | 2.130 | 0.018 | Cd81,Msn |
| Cytoskeleton regulation | Wound healing | 4 | 265 | 1.180 | 0.018 | Myh9,Gna13,Gnas,Anxa6 |
| Cytoskeleton regulation | Response to organic substance | 10 | 2742 | 0.560 | 0.019 | Rdx,Rab14,Cyfip1,Cd81,Lrp1,Gnai2,Actn4,Gnas,Itgb1,Msn |
| Cytoskeleton regulation | Macromolecule localization | 9 | 2198 | 0.610 | 0.019 | Rdx,Myh9,Rab21,Rab14,Cd81,Sigmar1,Actn4,Itgb1,Apoc1 |
| Cytoskeleton regulation | Tissue development | 8 | 1720 | 0.670 | 0.021 | Rdx,Rala,Gna13,Cd81,Gnas,Itgb1,Anxa6,Msn |
| Cytoskeleton regulation | Regulation of catabolic process | 6 | 872 | 0.840 | 0.022 | Rdx,Cd81,Lrp1,Itgb1,Apoc1,Msn |
| Cytoskeleton regulation | Platelet formation | 2 | 19 | 2.020 | 0.025 | Myh9,Actn1 |
| Cytoskeleton regulation | Regulation of axon extension | 3 | 112 | 1.430 | 0.025 | Rab21,Cyfip1,Lrp1 |
| Cytoskeleton regulation | Cellular response to low-density lipoprotein particle stimulus | 2 | 19 | 2.020 | 0.025 | Cd81,Itgb1 |
| Cytoskeleton regulation | Positive regulation of protein localization to membrane | 3 | 112 | 1.430 | 0.025 | Cd81,Lrp1,Itgb1 |
| Cytoskeleton regulation | Positive regulation of cell migration | 5 | 567 | 0.950 | 0.026 | Rdx,Lrp1,Gnai2,Actn4,Itgb1 |
| Cytoskeleton regulation | Response to endogenous stimulus | 7 | 1370 | 0.710 | 0.032 | Rdx,Rab14,Cyfip1,Cd81,Lrp1,Gnas,Itgb1 |
| Cytoskeleton regulation | Adenylate cyclase-activating g protein-coupled receptor signaling pathway | 3 | 128 | 1.370 | 0.033 | Gna13,Gnai2,Gnas |
| Cytoskeleton regulation | Establishment of cell polarity | 3 | 129 | 1.370 | 0.033 | Myh9,Itgb1,Msn |
| Cytoskeleton regulation | Positive regulation of intracellular signal transduction | 6 | 967 | 0.790 | 0.033 | Cd81,Lrp1,Gnai2,Actn4,Gnas,Itgb1 |
| Cytoskeleton regulation | Regulation of protein localization | 6 | 978 | 0.790 | 0.034 | Rdx,Rab14,Cd81,Lrp1,Itgb1,Msn |
| Cytoskeleton regulation | Intracellular signal transduction | 7 | 1402 | 0.700 | 0.034 | Rala,Rab21,Gna13,Rab14,Cyfip1,Gnai2,Gnas |
| Cytoskeleton regulation | Regulation of cellular component biogenesis | 6 | 978 | 0.790 | 0.034 | Rdx,Rala,Cyfip1,Lrp1,Msn,Arpc4 |
| Cytoskeleton regulation | Regulation of cell morphogenesis involved in differentiation | 4 | 343 | 1.070 | 0.037 | Rab21,Cyfip1,Lrp1,Actn4 |
| Cytoskeleton regulation | Regulation of actin cytoskeleton organization | 4 | 344 | 1.070 | 0.037 | Rdx,Cyfip1,Lrp1,Arpc4 |
| Cytoskeleton regulation | Regulation of renal sodium excretion | 2 | 26 | 1.890 | 0.037 | Gnai2,Gnas |
| Cytoskeleton regulation | Regulation of body fluid levels | 4 | 349 | 1.060 | 0.038 | Gna13,Rab14,Gnai2,Gnas |
| Cytoskeleton regulation | Myoblast fusion | 2 | 27 | 1.870 | 0.038 | Myh9,Cd81 |
| Cytoskeleton regulation | Axonogenesis | 4 | 351 | 1.060 | 0.038 | Alcam,Cyfip1,Lrp1,Itgb1 |
| Cytoskeleton regulation | Receptor-mediated endocytosis | 3 | 143 | 1.320 | 0.040 | Cd81,Lrp1,Itgb1 |
| Cytoskeleton regulation | Positive regulation of nitrogen compound metabolic process | 10 | 3119 | 0.510 | 0.040 | Rdx,Myh9,Actn1,Cd81,Lrp1,Gnai2,Actn4,Gnas,Itgb1,Msn |
| Cytoskeleton regulation | Cellular response to oxygen-containing compound | 6 | 1026 | 0.770 | 0.040 | Cyfip1,Lrp1,Sigmar1,Gnai2,Gnas,Msn |
| Cytoskeleton regulation | Cellular component assembly | 8 | 1983 | 0.610 | 0.041 | Rdx,Actn1,Rab14,Cyfip1,Sigmar1,Actn4,Itgb1,Arpc4 |
| Cytoskeleton regulation | Regulation of ruffle assembly | 2 | 29 | 1.840 | 0.041 | Rdx,Cyfip1 |
| Cytoskeleton regulation | Positive regulation of neuron projection development | 4 | 366 | 1.040 | 0.042 | Rab21,Cyfip1,Lrp1,Itgb1 |
| Cytoskeleton regulation | Response to nitrogen compound | 6 | 1064 | 0.750 | 0.045 | Cyfip1,Cd81,Lrp1,Gnai2,Gnas,Itgb1 |
| Cytoskeleton regulation | Regulation of multicellular organismal process | 10 | 3205 | 0.500 | 0.046 | Rab21,Rab14,Cyfip1,Cd81,Lrp1,Gnai2,Gnas,Itgb1,Apoc1,Anxa6 |
| Cytoskeleton regulation | Plasma membrane bounded cell projection organization | 6 | 1072 | 0.750 | 0.046 | Rdx,Myh9,Alcam,Cyfip1,Lrp1,Itgb1 |
| Cytoskeleton regulation | Blood vessel endothelial cell migration | 2 | 32 | 1.800 | 0.047 | Myh9,Itgb1 |
| Cytoskeleton regulation | Positive regulation of signal transduction | 7 | 1538 | 0.660 | 0.048 | Cyfip1,Cd81,Lrp1,Gnai2,Actn4,Gnas,Itgb1 |
| Cytoskeleton regulation | Regulation of cell adhesion | 5 | 705 | 0.850 | 0.048 | Rdx,Cd81,Lrp1,Actn4,Itgb1 |
| Cytoskeleton regulation | Regulation of growth | 5 | 701 | 0.850 | 0.048 | Rab21,Cyfip1,Cd81,Lrp1,Gnas |
| Cytoskeleton regulation | Regulation of developmental growth | 4 | 388 | 1.010 | 0.048 | Rab21,Cyfip1,Lrp1,Gnas |
| Cytoskeleton regulation | Establishment of endothelial barrier | 2 | 33 | 1.780 | 0.048 | Rdx,Msn |
| Cytoskeleton regulation | Regulation of actin filament polymerization | 3 | 165 | 1.260 | 0.050 | Rdx,Cyfip1,Arpc4 |
| Cytoskeleton regulation | Regulation of secretion by cell | 5 | 713 | 0.850 | 0.050 | Rala,Rab21,Lrp1,Gnai2,Gnas |
| OXPHOS | Electron transport chain, and mitochondrial proton-transporting ATP synthase complex | 21 | 108 | 2.110 | 0.000 | Cox5a,Ndufs3,Ndufs2,Cyc1,Ndufb4,Atp5o,Uqcrc1,Ndufs1,Ndufa4,Uqcrc2,Cox4i1,Ndufv1,Ndufb10,Uqcrfs1,Ndufa9,Ndufb6,Ndufs7,Ndufa13,Ndufb5,Ndufv2,Cycs |
| OXPHOS | Respirasome | 20 | 78 | 2.230 | 0.000 | Cox5a,Ndufs3,Ndufs2,Cyc1,Ndufb4,Uqcrc1,Ndufs1,Ndufa4,Uqcrc2,Cox4i1,Ndufv1,Ndufb10,Uqcrfs1,Ndufa9,Ndufb6,Ndufs7,Ndufa13,Ndufb5,Ndufv2,Cycs |
| OXPHOS | NADH dehydrogenase complex | 13 | 37 | 2.370 | 0.000 | Ndufs3,Ndufs2,Ndufb4,Ndufs1,Ndufa4,Ndufv1,Ndufb10,Ndufa9,Ndufb6,Ndufs7,Ndufa13,Ndufb5,Ndufv2 |
| OXPHOS | NADH dehydrogenase complex | 11 | 32 | 2.360 | 0.000 | Ndufs3,Ndufs2,Ndufb4,Ndufs1,Ndufv1,Ndufb10,Ndufa9,Ndufb6,Ndufs7,Ndufa13,Ndufb5 |
| OXPHOS | NADH dehydrogenase complex | 10 | 26 | 2.410 | 0.000 | Ndufs2,Ndufb4,Ndufs1,Ndufv1,Ndufb10,Ndufa9,Ndufb6,Ndufs7,Ndufa13,Ndufb5 |
| OXPHOS | NADH dehydrogenase complex | 7 | 20 | 2.370 | 0.000 | Ndufs2,Ndufs1,Ndufv1,Ndufa9,Ndufb6,Ndufs7,Ndufa13 |
| OXPHOS | Cytochrome complex | 7 | 25 | 2.270 | 0.000 | Cox5a,Cyc1,Uqcrc1,Uqcrc2,Cox4i1,Uqcrfs1,Cycs |
| OXPHOS | Mitochondrial electron transport, nadh to ubiquinone, and nadh dehydrogenase complex | 5 | 15 | 2.350 | 0.000 | Ndufs2,Ndufs1,Ndufv1,Ndufa9,Ndufs7 |
| OXPHOS | Respiratory chain complex iii, and copper centre cu(a) | 5 | 15 | 2.350 | 0.000 | Cyc1,Uqcrc1,Uqcrc2,Uqcrfs1,Cycs |
| OXPHOS | Respiratory chain complex iii | 4 | 10 | 2.430 | 0.000 | Cyc1,Uqcrc1,Uqcrc2,Uqcrfs1 |
| OXPHOS | Mixed, incl. etc complex i subunit conserved region, and nadh-ubiquinone oxidoreductase subunit 10 | 3 | 6 | 2.520 | 0.000 | Ndufb4,Ndufb10,Ndufb5 |
| OXPHOS | Mitochondrial electron transport, nadh to ubiquinone, and molybdopterin oxidoreductase | 3 | 9 | 2.350 | 0.000 | Ndufs2,Ndufs1,Ndufv1 |
| OXPHOS | Citric acid cycle (TCA cycle) | 3 | 10 | 2.300 | 0.000 | Sdhb,Sdha,Suclg1 |
| OXPHOS | Fumarate reductase complex | 2 | 5 | 2.430 | 0.010 | Sdhb,Sdha |
| OXPHOS | Mixed, incl. grim-19 protein, and nadh:ubiquinone oxidoreductase, b18 subunit | 2 | 5 | 2.430 | 0.010 | Ndufb6,Ndufa13 |
| OXPHOS | Respiratory chain complex iii | 2 | 5 | 2.430 | 0.010 | Uqcrc1,Uqcrc2 |
| OXPHOS | Mixed, incl. acyl binding, and ubiquinone-6 metabolic process | 2 | 6 | 2.350 | 0.012 | Ndufa9,Ndufs7 |
| OXPHOS | Mitochondrial respiratory chain complex iv, and cytochrome c oxidase subunit 6c | 2 | 6 | 2.350 | 0.012 | Cox5a,Cox4i1 |
| Protein folding and processing | Protein processing in endoplasmic reticulum, and signal peptide processing | 6 | 123 | 1.620 | 0.000 |  |
| Protein folding and processing | Protein processing in endoplasmic reticulum, and signal peptidase complex | 5 | 73 | 1.760 | 0.000 |  |
| Protein folding and processing | TAP complex, and MHC protein complex assembly | 3 | 6 | 2.630 | 0.000 |  |
| Protein folding and processing | Endoplasmic reticulum chaperone complex, and unfolded protein response | 3 | 29 | 1.940 | 0.009 |  |
| Protein folding and processing | Oligosaccharyltransferase complex | 2 | 5 | 2.530 | 0.017 |  |
| Protein folding and processing | Polycystin complex, and glucosidase ii complex | 2 | 6 | 2.450 | 0.020 |  |
| Protein folding and processing | Endoplasmic reticulum chaperone complex | 2 | 6 | 2.450 | 0.020 |  |
| Protein folding and processing | Mixed, incl. tim44-like, and chaperone dnak | 2 | 6 | 2.450 | 0.020 |  |
| Clathrin-mediated endocytosis | Clathrin coat, and synaptic vesicle recycling | 6 | 75 | 1.800 | 0.000 | Ap2a2,Ap2b1,Picalm,Cltc,Ap2a1,Dnm2 |
| Clathrin-mediated endocytosis | Clathrin coat, and clathrin-dependent endocytosis | 5 | 51 | 1.890 | 0.000 | Ap2a2,Ap2b1,Picalm,Cltc,Ap2a1 |
| Clathrin-mediated endocytosis | AP-2 adaptor complex | 3 | 5 | 2.670 | 0.000 | Ap2a2,Ap2b1,Ap2a1 |
| Clathrin-mediated endocytosis | Mixed, incl. protein localization to mitochondrion, and cristae formation | 4 | 109 | 1.460 | 0.005 | Samm50,Afg3l2,Immt,Mfn1 |
| Clathrin-mediated endocytosis | Glutathione S-transferase, Pi class, and methylarsonate reductase activity | 2 | 5 | 2.500 | 0.013 | Prdx6,Gstp1 |
| Clathrin-mediated endocytosis | Presynaptic endocytic zone membrane, and clathrin interactor 1 | 2 | 6 | 2.420 | 0.016 | Picalm,Cltc |
| Clathrin-mediated endocytosis | Protein maturation by protein folding, and disulphide isomerase | 2 | 6 | 2.420 | 0.016 | Ero1l,Ero1lb |
| Clathrin-mediated endocytosis | Protein targeting to mitochondrion, and cristae formation | 3 | 73 | 1.510 | 0.037 | Samm50,Afg3l2,Immt |
| Clathrin-mediated endocytosis | MICOS complex, and SAM complex | 2 | 12 | 2.120 | 0.040 | Samm50,Immt |
| Clathrin-mediated endocytosis | Detoxification of Reactive Oxygen Species | 2 | 13 | 2.080 | 0.044 | Prdx5,Prdx1 |
| TCA cycle | Carbon metabolism, and Pyruvate metabolism | 12 | 131 | 2.030 | 0.000 | Cs,Mdh2,Aco2,Fh1,Got2,Dlat,Eno1,Pgk1,Idh1,Mdh1,Ldha,Tpi1 |
| TCA cycle | TCA cycle, and Aminotransferase | 8 | 60 | 2.190 | 0.000 | Cs,Mdh2,Aco2,Fh1,Got2,Dlat,Idh1,Mdh1 |
| TCA cycle | Citrate cycle (TCA cycle), and Phenylalanine, tyrosine and tryptophan biosynthesis | 7 | 34 | 2.380 | 0.000 | Cs,Mdh2,Aco2,Fh1,Got2,Idh1,Mdh1 |
| TCA cycle | Oxaloacetate metabolic process, and cysteine-s-conjugate beta-lyase activity | 4 | 15 | 2.490 | 0.000 | Cs,Mdh2,Got2,Mdh1 |
| TCA cycle | Phosphoglycerate kinase, and Triosephosphate isomerase | 3 | 5 | 2.840 | 0.000 | Eno1,Pgk1,Tpi1 |
| TCA cycle | Oxaloacetate metabolic process | 3 | 7 | 2.700 | 0.000 | Cs,Mdh2,Mdh1 |
| TCA cycle | Glycolysis, and fructose metabolic process | 4 | 45 | 2.010 | 0.000 | Eno1,Pgk1,Ldha,Tpi1 |
| TCA cycle | Tricarboxylic acid cycle | 3 | 19 | 2.260 | 0.000 | Aco2,Fh1,Idh1 |
| TCA cycle | Porin, eukaryotic type, and ATP:ADP antiporter activity | 2 | 10 | 2.370 | 0.015 | Vdac2,Vdac1 |
| TCA cycle | Proton-transporting atp synthase activity, rotational mechanism | 2 | 14 | 2.220 | 0.025 | Atp5h,Atp5c1 |
| Fatty acid oxidation | Fatty acid beta-oxidation, and valine, leucine and isoleucine degradation | 14 | 70 | 2.250 | 0.000 | Mccc2,Hsd17b4,Echs1,Acadl,Mccc1,Etfdh,Decr1,Acsl4,Acat1,Acaa2,Acox1,Aldh6a1,Hadhb,Hadha |
| Fatty acid oxidation | Mixed, incl. fatty acid beta-oxidation, and microbody membrane | 15 | 122 | 2.040 | 0.000 | Mccc2,Hsd17b4,Echs1,Acadl,Mccc1,Etfdh,Abcd3,Decr1,Acsl4,Acat1,Acaa2,Acox1,Aldh6a1,Hadhb,Hadha |
| Fatty acid oxidation | Mixed, incl. fatty acid oxidation, and peroxisome | 16 | 185 | 1.880 | 0.000 | Mccc2,Hsd17b4,Echs1,Acadl,Mccc1,Etfdh,Abcd3,Decr1,Acsl4,Acsl1,Acat1,Acaa2,Acox1,Aldh6a1,Hadhb,Hadha |
| Fatty acid oxidation | Fatty acid beta-oxidation, and long-chain fatty acid-coa ligase activity | 10 | 42 | 2.320 | 0.000 | Hsd17b4,Echs1,Acadl,Etfdh,Decr1,Acsl4,Acaa2,Acox1,Hadhb,Hadha |
| Fatty acid oxidation | Fatty acid beta-oxidation | 9 | 31 | 2.410 | 0.000 | Hsd17b4,Echs1,Acadl,Etfdh,Decr1,Acaa2,Acox1,Hadhb,Hadha |
| Fatty acid oxidation | Mitochondrial fatty acid beta-oxidation of saturated fatty acids, and electron transfer flavoprotein complex | 7 | 15 | 2.610 | 0.000 | Echs1,Acadl,Etfdh,Decr1,Acaa2,Hadhb,Hadha |
| Fatty acid oxidation | Mitochondrial fatty acid beta-oxidation of saturated fatty acids | 5 | 10 | 2.640 | 0.000 | Echs1,Acadl,Acaa2,Hadhb,Hadha |
| Fatty acid oxidation | Ketone body metabolism, and branched-chain amino acid catabolic process | 4 | 28 | 2.100 | 0.000 | Mccc2,Mccc1,Acat1,Aldh6a1 |
| Fatty acid oxidation | Beta oxidation of palmitoyl-CoA to myristoyl-CoA, and medium-chain fatty acid catabolic process | 3 | 5 | 2.720 | 0.000 | Acaa2,Hadhb,Hadha |
| Fatty acid oxidation | Mixed, incl. coa carboxylase activity, and acetate-coa ligase activity | 3 | 15 | 2.250 | 0.000 | Mccc2,Mccc1,Aldh6a1 |
| Fatty acid oxidation | Carnitine synthesis, and cellular detoxification of aldehyde | 2 | 6 | 2.470 | 0.013 | Aldh9a1,Aldh2 |
| Fatty acid oxidation | acyl-CoA oxidase activity, and SCP2 sterol-binding domain | 2 | 6 | 2.470 | 0.013 | Hsd17b4,Acox1 |
| Fatty acid oxidation | CoA carboxylase activity, and Isovaleryl-CoA dehydrogenase | 2 | 6 | 2.470 | 0.013 | Mccc2,Mccc1 |
| ER to golgi vesicle-mediated transport | Endoplasmic reticulum to golgi vesicle-mediated transport | 4 | 99 | 1.770 | 0.008 | Vapa,Rab1b,Tmed10,Rab1 |
| ER to golgi vesicle-mediated transport | Golgi vesicle budding | 3 | 28 | 2.200 | 0.008 | Vapa,Tmed10,Rab1 |
| ER to golgi vesicle-mediated transport | Positive regulation of glycoprotein metabolic process | 3 | 27 | 2.210 | 0.008 | Ncstn,Rab1b,Rab1 |
| ER to golgi vesicle-mediated transport | Rab protein signal transduction | 3 | 66 | 1.820 | 0.028 | Rab1b,Rab18,Rab1 |
| ER to golgi vesicle-mediated transport | Symbiotic process | 4 | 247 | 1.380 | 0.035 | Vapa,Rab1b,Ppib,Rab1 |
| ER to golgi vesicle-mediated transport | Positive regulation by host of viral genome replication | 2 | 8 | 2.570 | 0.035 | Vapa,Ppib |
| Protein synthesis | SRP-dependent cotranslational protein targeting to membrane | 18 | 81 | 2.100 | 0.000 | Rpl8,Rps9,Rpl10,Rps14,Rps3a1,Rpl6,Rps3,Rpl4,Eef2,Rpl10a,Rpl7,Rpl5,Rplp2,Rplp0,Rps8,Rpl18,Rpl9,Rps24 |
| Protein synthesis | Mixed, incl. cytosolic ribosome, and elongation factor | 20 | 182 | 1.790 | 0.000 | Rpl8,Rps9,Rpl10,Rps14,Pa2g4,Rps3a1,Rpl6,Rps3,Rpl4,Eef1a1,Eef2,Rpl10a,Rpl7,Rpl5,Rplp2,Rplp0,Rps8,Rpl18,Rpl9,Rps24 |
| Protein synthesis | Cytosolic ribosome, and elongation factor | 19 | 152 | 1.850 | 0.000 | Rpl8,Rps9,Rpl10,Rps14,Rps3a1,Rpl6,Rps3,Rpl4,Eef1a1,Eef2,Rpl10a,Rpl7,Rpl5,Rplp2,Rplp0,Rps8,Rpl18,Rpl9,Rps24 |
| Protein synthesis | Cytosolic large ribosomal subunit | 10 | 44 | 2.110 | 0.000 | Rpl10,Rpl6,Rpl4,Rpl10a,Rpl7,Rpl5,Rplp2,Rplp0,Rpl18,Rpl9 |
| Protein synthesis | Cytosolic large ribosomal subunit | 9 | 38 | 2.130 | 0.000 | Rpl6,Rpl4,Rpl10a,Rpl7,Rpl5,Rplp2,Rplp0,Rpl18,Rpl9 |
| Protein synthesis | Cytosolic small ribosomal subunit | 8 | 34 | 2.120 | 0.000 | Rpl8,Rps9,Rps14,Rps3a1,Rps3,Eef2,Rps8,Rps24 |
| Protein synthesis | Cytosolic small ribosomal subunit | 6 | 27 | 2.100 | 0.000 | Rps9,Rps14,Rps3a1,Rps3,Rps8,Rps24 |
| Protein synthesis | Cytosolic large ribosomal subunit | 6 | 31 | 2.040 | 0.000 | Rpl6,Rpl4,Rpl7,Rpl5,Rpl18,Rpl9 |
| Protein synthesis | Cytosolic small ribosomal subunit | 5 | 17 | 2.220 | 0.000 | Rps9,Rps3a1,Rps3,Rps8,Rps24 |
| Protein synthesis | Cytosolic large ribosomal subunit | 5 | 26 | 2.040 | 0.000 | Rpl6,Rpl4,Rpl7,Rpl18,Rpl9 |
| Protein synthesis | Cytosolic small ribosomal subunit | 4 | 11 | 2.310 | 0.000 | Rps3a1,Rps3,Rps8,Rps24 |
| Protein synthesis | Ribosomal protein L12 family, and Ribosomal protein L30e | 3 | 5 | 2.530 | 0.000 | Rpl10a,Rplp2,Rplp0 |
| Protein synthesis | Mixed, incl. ribosomal protein l28e, and ribosomal protein l18e | 3 | 7 | 2.380 | 0.000 | Rpl6,Rpl4,Rpl18 |
| Protein synthesis | Alpha-amino acid metabolic process, and beta-alanine metabolism | 4 | 109 | 1.320 | 0.007 | Mthfd1,Ahcy,Ass1,Aldh1l1 |
| Protein synthesis | C6 deamination of adenosine, and Protein LYRIC | 2 | 5 | 2.350 | 0.009 | Snd1,Mtdh |
| Protein synthesis | Mixed, incl. folic acid-containing compound metabolic process, and s-adenosylmethionine metabolic process | 3 | 52 | 1.510 | 0.018 | Mthfd1,Ahcy,Aldh1l1 |
| Protein synthesis | One carbon pool by folate | 2 | 12 | 1.970 | 0.037 | Mthfd1,Aldh1l1 |
